# Identification of genes and long non-coding RNAs for intramuscular and subcutaneous fat deposition in ducks by transcriptome analysis

**DOI:** 10.5713/ab.25.0268

**Published:** 2025-08-12

**Authors:** Tingting Zhou, Xunhao Meng, Wenshuang Liang, Min Xue, Tianqi Yang, Yong Jiang, Hao Bai, Guobin Chang, Guohong Chen, Zhixiu Wang

**Affiliations:** 1 Laboratory of Animal Genetics and Breeding and Molecular Design of Jiangsu Province, College of Animal Science and Technology, Yangzhou University, Yangzhou, China

**Keywords:** Cell Differentiation, Duck, Intramuscular Preadipocytes, Long Non-coding RNA, Subcutaneous Preadipocytes, Whole Transcriptome Sequencing

## Abstract

**Objective:**

Fat deposition is an important factor that affects meat production and quality in livestock and poultry. Long non-coding RNAs (lncRNAs) play an important role in duck fat deposition. The purpose of this study was to identify key lncRNAs and mRNAs involved in fat deposition of meat ducks based on whole transcriptome sequencing for intramuscular preadipocyte (IMP-0), intramuscular adipocyte after 4 days of induction (IMP-4), subcutaneous preadipocyte (SCP-0), and subcutaneous adipocyte after 4 days of induction (SCP-4).

**Methods:**

Differentially expressed mRNAs and lncRNAs were identified across groups through differential expression analysis, specific gene screening, and functional enrichment analysis. Subsequently, a lncRNA-mRNA co-expression network was constructed and key nodes were identified. Finally, preliminary expression validation was performed at the mRNA level.

**Results:**

Differential expression analysis revealed 1,419 mRNAs and 697 lncRNAs in the IMP-0-vs-IMP-4 comparison, and 2,307 mRNAs and 1,180 lncRNAs in the SCP-0-vs-SCP-4 comparison. Venn analysis identified unique differentially expressed genes for each group, including *CHKA*, *PNPLA2*, *PLPP1*, *FABP4*, *ACSL5*, *UGT8*, *FAT1*, and *FADS2*. Functional enrichment showed that the IMP-0-vs-IMP-4 group was significantly associated with regulation of the MAPK cascade, lipid binding, and arachidonic acid metabolism. The SCP-0-vs-SCP-4 group was notably enriched in beta-alanine metabolism, the Wnt signaling pathway, and lipid metabolic processes. Co-expression network analysis further constructed a network of 193 nodes and 275 edges for the IMP-0-vs-IMP-4 group, and a larger network of 564 nodes and 3,471 edges for the SCP-0-vs-SCP-4 group. Key lncRNAs, such as MSTRG.8652.4, MSTRG.15586.1, and MSTRG.6393.1, were identified based on their high connectivity degree.

**Conclusion:**

Taken together, the current findings indicated that there are differentially regulated differential genes, lncRNAs, and enrichment pathways in IMP-0-vs-IMP-4 and SCP-0-vs-SCP-4. Because of being differentially regulated, some differential factors were significantly increased in expression in intramuscular adipocyte induction while significantly downregulated in subcutaneous adipocyte induction, such as FABP3, MSTRG.13937.5, and MSTRG.6393.1. Meanwhile, there were also some factors that were specifically regulated, CHKA, PLA2G4A, FADS2, MSTRG.13842.1, MSTRG.16051.2 and MSTRG.13842.1 were significantly downregulated only in subcutaneous adipocytes. This suggests that these lncRNAs and their target genes may play important roles in intramuscular fat and subcutaneous fat deposition.

## INTRODUCTION

The duck industry is a specialty that plays an important role in the agricultural economy. In 2024, 4.220 billion meat ducks were slaughtered in China, promoting the effective supply of high-quality animal protein. The composition of duck meat is different from that of chicken and turkey. The fat percentage, lipid level and oxidative energy metabolism of duck meat is higher than that of other common poultry meat [1]. Modern commercial ducks have undergone extensive genetic selection for their rapid growth. Fat deposition is an important factor affecting meat production and quality in meat duck [2,3]. Intramuscular fat and subcutaneous fat content are economically important characteristics in meat duck production. Excessive accumulation of subcutaneous fat reduces the proportion of lean meat, whereas intramuscular fat quantifies the lipids deposited in the muscle, both between and within muscle fibers, and elevated levels are associated with improved meat quality [4–6].

The smallest units of adipose tissue are fat cells, and the adipose tissues of livestock and poultry are composed of many fat cells. The ability of adipocyte precursors to differentiate into new adipocytes runs through the whole life process, and adipocytes can be distributed in multiple parts of the body [7]. Adipocyte differentiation of duck is a complex biological process, which is induced by a variety of transcription factors [8]. Through various signal transduction pathways, different transcription factors are induced to regulate the expression of adipocyte-specific genes. Nowadays, some genes related to lipid metabolism have been found, including *PPAR*-*γ* [9], *FABP4* [10], *C/EBPα* [11], *ELOVL5* [12], *PLIN2* [13]. What’s more, an increasing number of studies have shown that some non-coding RNAs, such as long non-coding RNAs (lncRNAs) [14], play essential regulatory roles by forming complex and precise post-transcriptional regulatory networks. LncRNAs are nucleotide sequences more than 200 nucleotides in length that cannot encode proteins or can only encode peptides. It regulates gene expression at the epigenetic, transcriptional, and posttranscriptional levels [15]. Among all transcribed regions, lncRNAs are a key element of the transcriptome and its regulation [16]. LncRNAs engage in many post-transcriptional regulations and regulate target genes through antisense, cis, and trans interaction. Some antisense lncRNA may bind to the mRNA of the sense strand, resulting in the regulation of gene silencing, transcription, and mRNA stability.

Whole transcriptome sequencing technologies have been widely utilized in the past decades to reveal the genetic origins of phenotypic traits because they can capture a high-resolution picture of the transcriptomic landscape [17,18]. In recent years, an increasing number of studies have shown that lncRNAs, which were once considered genome noise, can mediate the epigenetic, transcriptional, and post-transcriptional regulation of fat deposition-related genes by participating in the fat deposition regulatory network [19–21], such as lnc-BATE1 [22], lnc-OAD [23] and lnc-ORA [24].

The lower expression of genes regulating lipogenesis and lipolysis in intramuscular adipocytes resulted in a significantly lower lipogenic differentiation capacity of intramuscular adipocytes than that of subcutaneous adipocytes. This suggests that there is site specificity in the deposition of intramuscular and subcutaneous fat in animals [25,26]. Therefore, the aim of this experiment was to compare the differentially expressed genes (DEGs) and lncRNAs in subcutaneous precursor adipocytes (SCP) and intramuscular precursor adipocytes (IMP) of broiler ducks after induced differentiation by transcriptome sequencing, in order to provide new ideas and directions for future in-depth studies on the mechanisms of the regulation of subcutaneous and intramuscular fat deposition, and to reveal the unique modes of regulation of the two parts of the body fat.

## MATERIALS AND METHODS

### Data and sample collection

Cherry Valley ducks used in this study were purchased from Shuyang Zhongke Seed Poultry. Subcutaneous and intramuscular preadipocytes were obtained from subcutaneous fat and muscle tissues of 8-day-old Cherry Valley ducks by trypsin digestion and differential adhesion. Isobutylmethylxanthine (IBMX, 0.5 mM), insulin (1 mg/mL), rosiglitazone (RSG) and Dex (1 mM, Sigma-Aldrich) were used to induce preadipocytes. The subcutaneous fat cells and intramuscular fat cells before induction were recorded as SCP-0 and IMP-0, respectively. Subcutaneous adipocytes and intramuscular adipocytes were induced with an induction system for 4 days, recorded as SCP-4 and IMP-4. All culture systems were per-formed at 37°C in 95% humidity with 5% CO_2_ (Thermo Fisher Scientific).

### RNA template preparation for total RNA extraction

RNA was extracted from four groups: intramuscular preadipocyte (IMP-0), intra-muscular adipocyte after 4 days of induction (IMP-4), subcutaneous preadipocyte (SCP-0), and subcutaneous adipocyte after 4 days of induction (SCP-4). Total RNA was isolated from the cells using the TRIzol Reagent kit (Invitrogen), as described above. The concentration and purity of all RNA samples were determined using a NanoDrop2000 Spectrophotometer (Thermo Fisher Scientific) and RNA integrity was examined using agarose gel electrophoresis. Qualified RNA was stored in a refrigerator at −80°C.

### RNA library preparation and sequencing

Preparation and deep sequencing of the full transcriptome library were performed using Gene Denovo. Using the PrimeScriptTM RT reagent kit with a gDNA Eraser reverse transcription kit (Thermo Fisher Scientific), cDNA was synthesized by reverse transcription according to the manufacturer’s instructions. In accordance with the manufacturer’s instructions, whole-transcriptome libraries were constructed using the TruSeq RNA Sample Preparation Kit (Illumina). qPCR-ABI 7500 was used for quantification and an Agilent2100 was used to detect the invariant size of the library. After library inspection, the cDNA library was constructed and sequenced on an Illumina HiSeqTM 4000.

### Construction and analysis of coding and non-coding genes

Primary intramuscular and subcutaneous preadipocytes were isolated from ducks and cultured in 6-well plates. When the cells grew to a confluence of approximately 90%, they were washed twice with PBS containing 1% double antibodies. After 4 days of induction of primary cell differentiation with induction medium, they were washed twice with PBS containing 1% double antibodies. The TRIzol reagent (1 mL) was added to each well to lyse the cells. After cell lysis, the liquid was collected, packed into a 1.5 mL centrifuge tube and placed in a refrigerator at 80°C for cryopreservation. Each cell had three replicates, which were transported on dry ice at low temperature and sent to Gideon Biotechnology, where the library was constructed by whole transcriptome sequencing. Two libraries were constructed for each cell, namely Illumina HSeq4000 (for mRNA and lncRNA).

### Sequencing data analysis

Genes were counted using HTSeq, and the number of transcripts (TPMs) per thousand million reads was calculated to evaluate the gene expression levels. The edgeR package in the R software (ver. 3.5.2) was used to screen for differentially expressed mRNA and lncRNA (DE mRNA and DE lncRNA) in subcutaneous and intramuscular duck adipocytes. Statistical significance was defined as log2 | (fold change)|≥1 and p-adjusted<0.05. The volcano plot and heatmap of DE RNA were plotted using ggplot and heatmap packages.

### Gene function and pathway enrichment analysis

Gene Ontology (GO) is widely used in bioinformatics to analyze gene function from three aspects: cellular components, molecular functions, and biological processes (BP). The Kyoto Encyclopedia of Genes and Genomes (KEGG) is a database used for analyzing gene function and genomic information, allowing the study of genes and gene expression information as a whole network [27]. GO and KEGG pathway enrichment analyses of DEGs, were performed using OmicStudio (https://www.omicstudio.cn/index). In this study, GO annotation and KEGG functional enrichment analyses of the DE mRNA and DE lncRNA were performed.

### Long non-coding RNA-mRNA co-expression network

A differentially expressed lncRNA-mRNA co-expression network was constructed to explore the interaction between intramuscular adipocytes and subcutaneous adipocytes before and after induction. The Pearson correlation coefficient (r) of lncRNAs and mRNAs was calculated to obtain the correlation between their expression levels. The lncRNA-mRNA co-expression network was constructed by setting |r|>0.9 and p<0.01 and then imported into the Omicsmart platform (https://www.omicsmart.com/#/) for visualization. Connectivity is a metric used to evaluate the importance of lncRNA-mRNA in a network. The greater the degree, the greater its moderating effect on the network. By calculation, the top 10 differentially expressed lncRNAs were listed.

### Validation of differentially expressed mRNA and differentially expressed long non-coding RNA using real-time quantitative polymerase chain reaction

PowerUp SYBR Green Master Mix (A25742; Thermo Fisher Scientific) and the LightCycler 96 Real-Time polymerase chain reaction (PCR) Detection System (Roche) were used for quantitative reverse transcription-PCR (qRT-PCR). A 20 μL reaction volume consisting of 10 μL PowerUp SYBR Green Master Mix (2X), 0.8 μL Forward Primer, 0.8 μL Reverse Primer, 2 μL cNDA template, and 6.4 μL ddH2O was used. The following qRT-PCR conditions were used: denaturation at 95°C for 10 min, followed by 40 cycles of denaturation at 95°C for 3 s, annealing at 50°C–60°C for 30 s, and elongation at 72°C for 20 s. Each sample was analyzed in triplicate. GAPDH was used as an internal control for mRNA, and all RT-qPCR reactions were performed at least three times independently (Table 1).

### Statistical analysis

SPSS software was used for data analysis, and results are presented as the mean ± standard error of the mean (SEM). Gene expression was calculated using the relative quantification (2^−ΔΔCT^) method. A t-test was used for pairwise analysis using SPSS (ver. 22.0; IBM). Differences were considered statistically significant at p≤0.05 (* p≤0.05 and ** p≤0.01). GraphPad Prism 9.0 (GraphPad Software) was used for statistical analysis and graph creation.

## RESULTS

### *In vitro* cell culture and results of oil red O staining

**Figure 1:**
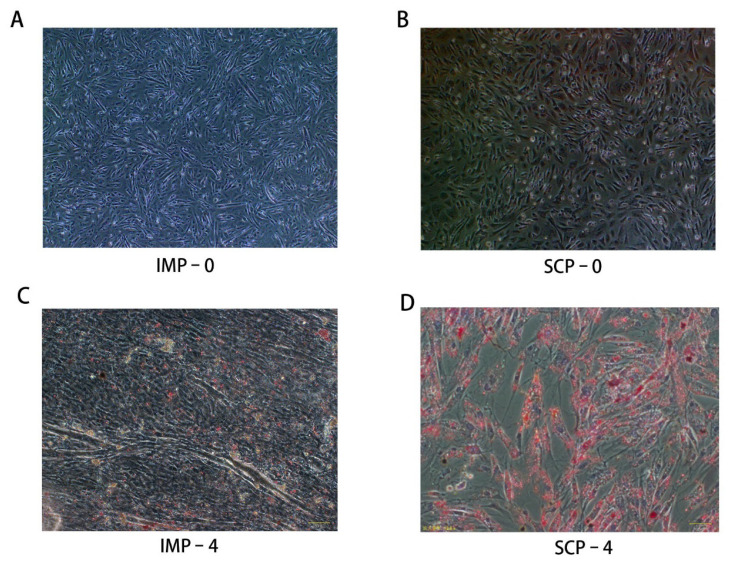
Oil red O staining of intramuscular adipocytes and subcutaneous adipocytes at different time after differentiation. (A) Before IMP induction (Oil red O staining, ×4). (B) Before SCP induction (Oil red O staining, ×4). (C) After IMP induction (Oil red O staining, ×10). (D) After SCP induction (Oil red O staining, ×10). IMP, intramuscular preadipocytes; SCP, subcutaneous preadipocytes.

In cell culture in vitro, it was found that the phenotype of differentiated and undifferentiated preadipocytes changed. Oil Red O staining revealed that the amount of visible and sporadic lipid droplets increased starting from four days after differentiation (Figure 1). Precursor adipocytes do not have lipid droplet aggregation before induction, and after induction of differentiation, the number of lipid droplets gradually increases, the morphology gradually becomes rounded, and a large number of small lipid droplets will be aggregated together and become large lipid droplets, which are stained red. However, it can be clearly seen that after 4 days of induction, there were more red lipid droplets in subcutaneous fat cells than in intramuscular fat cells.

### Quality control of sequencing data

To guarantee the data, we filtered low-quality RNA-sequencing data using fastp and obtained clean reads of approximately 99.80% of the original reads from the four evaluated cell treatment groups (Table 2). In addition, among the four groups, the Q20 values ranged from 97.55%–97.79%, and the Q30 values ranged from 92.96% to 93.47% (Supplement 1). The percentage of clean reads mapped to the ribosomal RNA database ranged from 0.25% to 0.36% (Supplement 2). Remove the reads from the upper ribosome in case of mismatches, and retain the unmapped reads for subsequent transcriptome analysis. Other reads were analyzed using CNCI, CPC, CPAT, LGC, and PfamScan.

### Global responses of mRNA to fat deposition

**Figure 2:**
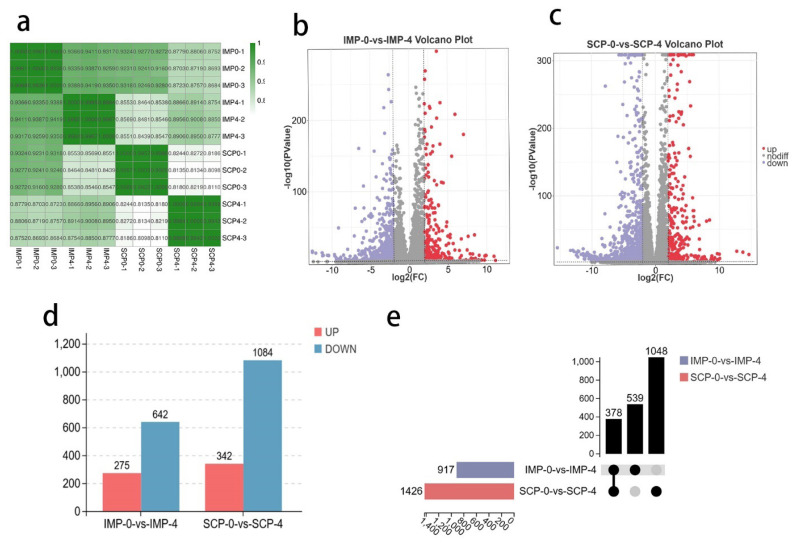
Identification of differentially expressed genes (DEGs). (a) Statistical diagram of the different genes. Volcano plot for the identified DEGs in IMP-0-vs-IMP-4 (b), SCP-0-vs-SCP-4 (c), based on the criteria of p<0.01 and |log2fold change (FC)|>2. Red and purple dots indicate the upregulated and downregulated mRNAs, respectively. (d) Venn diagrams of mRNAs expressed in two groups. (e) Heatmap of all DE lncRNA expression in each sample. IMP, intramuscular preadipocytes; SCP, subcutaneous preadipocytes; DE, differentially expressed; lncRNA, long non-coding RNA.

The heat map shows the DE mRNA expression profiles of the four groups, the correlation results are excellent for the next analysis (Figure 2A). At a cut-off with an absolute value of |log2FC|>2 and p-value<0.01, 917 DEGs were identified in the IMP-0 group compared with the IMP-4 group, including 275 upregulated and 642 downregulated DEGs (Figure 2B, Supplement 3). Based on these standards, there were 1426 DEGs (342 upregulated and 1084 downregulated) between the SCP-0 and SCP-4 groups (Figure 2C, Supplement 4). The bar chart for the differential fundamental analysis is shown in Figure 2d. In addition, 539 genes and 1,048 genes were specifically expressed in IMP-0-vs-IMP-4 and SCP-0-vs-SCP-4, respectively (Figure 2E).

### Functional analysis of the identified differentially expressed genes

**Figure 3:**
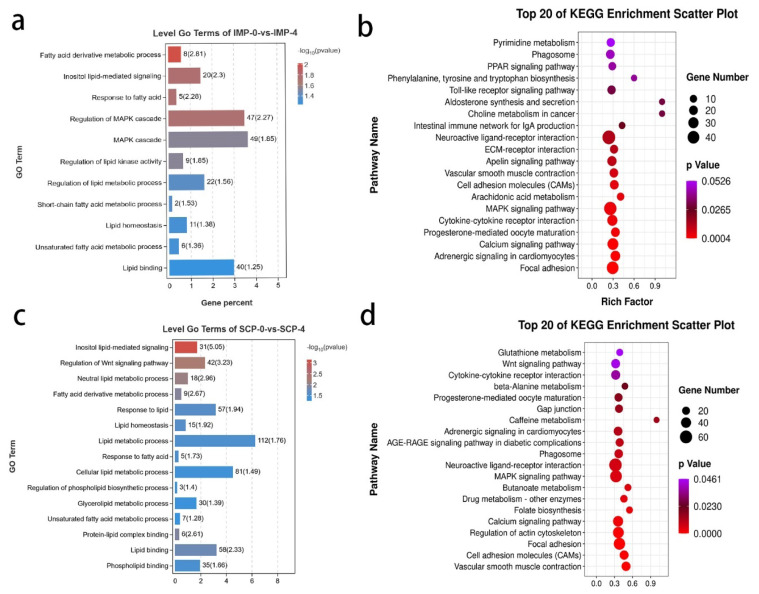
Functional analysis of identified differentially expressed genes (DEGs) Gene Ontology (GO) Terms and Kyoto Encyclopedia of Genes and Genomes (KEGG) pathway. (a, b) GO and KEGG in IMP-0-vs-IMP-4. (c, d) SCP-0-vs-SCP-4. IMP, intramuscular preadipocytes; SCP, subcutaneous preadipocytes.

To further analyze the function of differentially expressed mRNAs in adipocytes from different adipose tissue sources (IMP and SCP), GO and KEGG pathway analyses were carried out. In IMP-0-vs-IMP-4, significant enriched GO terms were regulation of MAPK cascade, lipid binding, etc (Figure 3A). KEGG pathways involved in lipid metabolism-related pathways, including Arachidonic acid metabolism, Aldosterone synthesis and secretion, PPAR signaling pathway and so on (Figure 3B). In SCP-0-vs-SCP-4, the most KEGG significant differences in biological functions were mainly in the MAPK signaling pathway, beta-alanine metabolism, Wnt signaling pathway, etc (Figure 3C). GO analysis showed that the BP related to lipid metabolism in differentially expressed mRNAs were mainly concentrated in lipid metabolic process, cellular lipid metabolic process, etc (Figure 3D).

### Protein-protein interaction network analysis for differentially expressed genes

For differentially expressed mRNAs, protein-protein interaction (PPI) enrichment analysis was carried out through String (https://cn.string-db.org/). MCODE (Cytoscape, Cytoscape Consortium, CA, USA) was also used to identify closely connected network components. In the PPI network, combine-score≥0.6 and degree≥10 were used as thresholds to screen key nodes. In order to investigate the mutual interaction of the identified DE-mRNAs, PPI networks were constructed for SCP-0-vs-SCP-4 and IMP-0-vs-IMP-4 DE-mRNAs, respectively.

**Figure 4:**
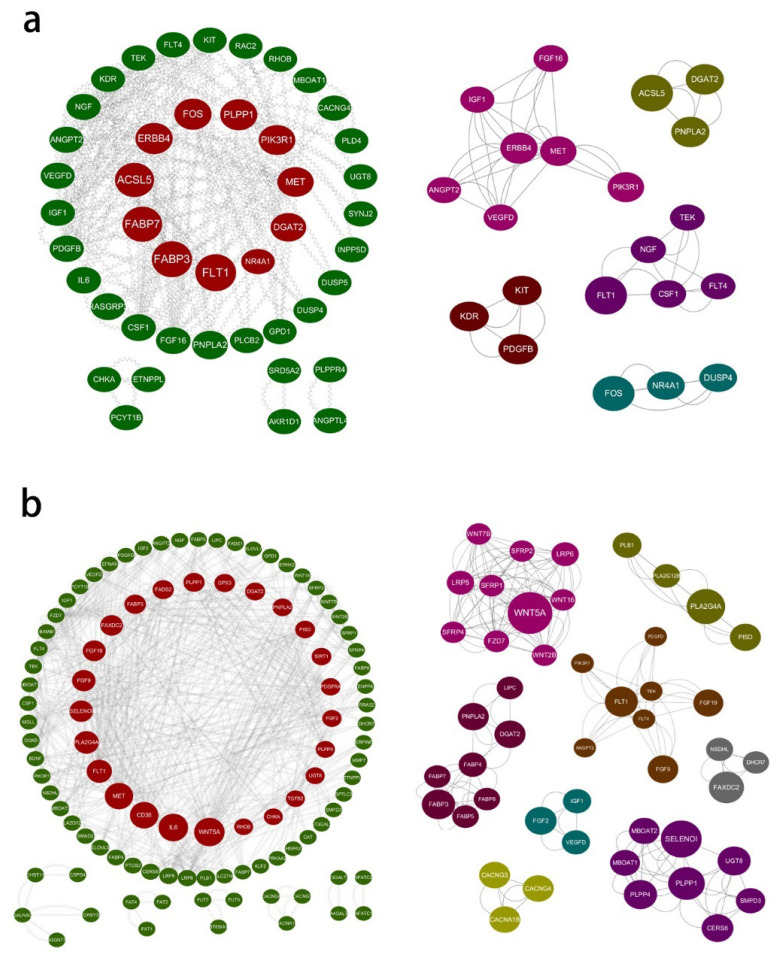
All protein-protein interactions among differentially expressed mRNAs were extracted from PPI data source and MCODE algorithm was applied to identify neighborhoods where proteins were densely connected. (a) A PPI network of IMP-0-vs-IMP-4 and five MCODE modules of IMP-0-vs-IMP-4 were enriched. (b) A PPI network of SCP-0-vs-SCP-4 and Eight MCODE modules of SCP-0-vs-SCP-4 were enriched. PPI, protein-protein interaction; IMP, intramuscular preadipocytes; SCP, subcutaneous preadipocytes.

In IMP-0-vs-IMP-4, The PPI network of IMP-0 and IMP-4 have 72 nodes and 152 edges. In addition, five MCODE modules were enriched (Figure 4A), among which MET, ERBB4, KDR, PDGFB, PNPLA2 and other encoded proteins were at the key node positions. This has an important regulatory role in intramuscular fat metabolism (Supplement 5).

In SCP-0-vs-SCP-4, the PPI included 130 nodes and 273 edges (Figure 4B). According to the annotation of MCODE, PPI enrichment analysis showed that 8 MCODE modules were enriched. Among them, WNT5A, SELENO1, FABP3, PLT1, PLA2G4A and other encoded proteins were at the key node positions, which may be involved in subcutaneous fat metabolism (Supplement 6).

### Global responses of long non-coding RNA to fat deposition

**Figure 5:**
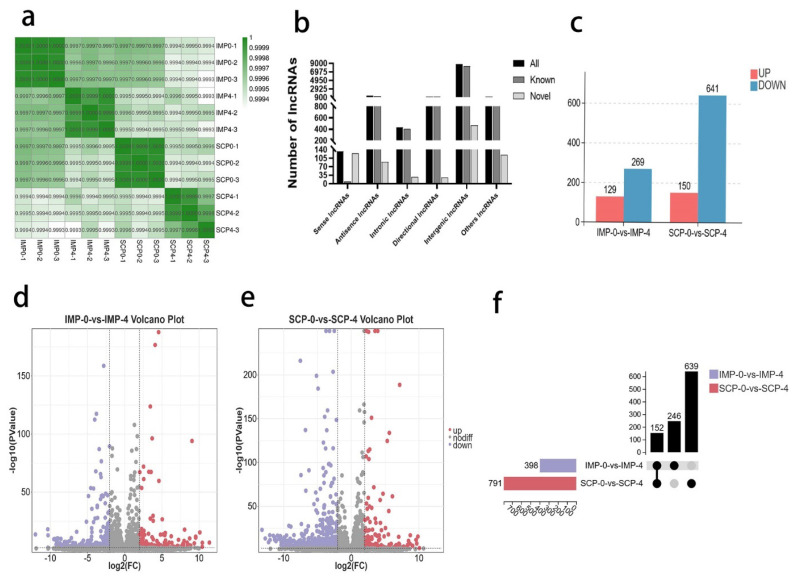
Identification of differentially expressed lncRNA (DE lncRNAs). (a) Heatmap of all DE lncRNA expression in each sample. (b) Statistical diagram of lncRNA types. (c) Statistical diagram of the different lncRNAs. Volcano plot for the identified DELs in IMP-0-vs-IMP-4 (d), SCP-0-vs-SCP-4 (e), based on the criteria of p<0.01 and |log2fold change (FC)|>2. Red and purple dots indicate the upregulated and downregulated lncRNAs, respectively. (f) Venn diagrams of lncRNAs expressed in two groups. IMP, intramuscular preadipocytes; SCP, subcutaneous preadipocytes; lncRNA, long non-coding RNA.

A heat map of these DE lncRNA is shown in Figure 5A. Through prediction of the lncRNA type, the new lncRNAs were divided into five categories (Figure 5B). A total of 1189 lncRNAs were identified (Figure 5C) by examining the intersection of the five tools included in the subsequent analysis. There were 398 lncRNA in IMP-0-vs-IMP-4, including 129 upregulated and 269 downregulated lncRNA (Figure 5D, Supplement 7). There were 791 lncRNA in SCP-0-vs-SCP-4, including 150 upregulated and 641 downregulated lncRNA (Figure 5E, Supplement 8). Moreover, 246 genes and 630 genes were specifically expressed in IMP-0-vs-IMP-4 and SCP-0-vs-SCP-4, respectively (Figure 5F). In this part of the study, the differentially expressed lncRNA were screened. Combined with the DE mRNA, candidate functional mRNA can be further screened. These lncRNA may be closely related to genetic backgrounds.

### Combined analysis of differential long non-coding RNA and mRNA unique to IMP-0-vs-IMP-4 and SCP-0-vs-SCP-4

**Figure 6:**
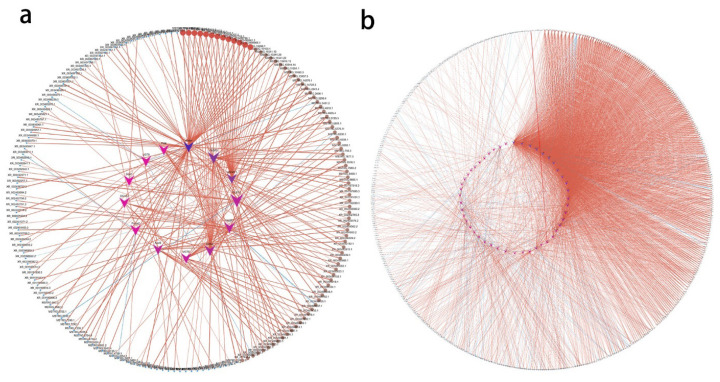
A lncRNA-mRNA co-expression network of IMP-0-vs-IMP-4 and SCP-0-vs-SCP-4. (a) IMP-0-vs-IMP-4 (b) SCP-0-vs-SCP-4. The triangles in the nodes represent the mRNA, the circles represent the lncRNA, and the edges represent the interactions between the lncRNA and the mRNA. In addition, the thickness of the line indicates the magnitude of the correlation coefficient, with red representing a positive correlation and blue representing a negative correlation. lncRNA, long non-coding RNA; IMP, intramuscular preadipocytes; SCP, subcutaneous preadipocytes.

In order to explore the interaction between differentially expressed lncRNAs and mRNAs, the transtarget genes of differentially expressed lncRNAs in intramuscular adipose tissue and subcutaneous adipose tissue were predicted, where lncRNAs may correspond to multiple mRNAs and one mRNA may correspond to multiple lncRNAs. According to |r|>0.9 and p<0.01, combined with GO and KEGG analysis, the target genes related to lipid metabolism, adipocyte differentiation, fatty acid biosynthesis and metabolism were screened, and the lncRNA-mRNA networks of IMP-0-vs-IMP-4 and SCP-0-vs-SCP-4 were constructed. Among them, the results of PPI analysis intersected, and there were 12 and 46 DEGs in the IMP-0-vs-IMP-4 group (Figure 6A) and the SCP-0-vs-SCP-4 group (Figure 6B), respectively.

The IMP-0-vs-IMP-4 group constituted a lncRNA-mRNA co-expression network with 193 nodes and 275 edges (Supplement 9). MSTRG.7877.3, MSTRG.6393.1, MSTRG.13937.5, MSTRG.2868.1, XR_003492841.1 (LOC113840110), MSTRG.10341.22, MSTRG.10341.20, MSTRG.5135.3, MSTRG.7978.1, MSTRG.3461.1 are key lncRNAs (Table 3). The SCP-0-vs-SCP-4 group constitutes a lncRNA-mRNA co-expression network with 395 nodes and 1,362 edges (Supplement 10). Moreover, we sorted the differentially expressed lncRNAs by connectivity degree, and the top 10 lncRNAs were listed in Table 4, such as MSTRG.8652.4, XR_003499406.1 (LOC113844692), for further study.

### Quantitative reverse transcription-polymerase chain reaction validation of differentially expressed mRNAs and differentially expressed long non-coding RNAs

**Figure 7:**
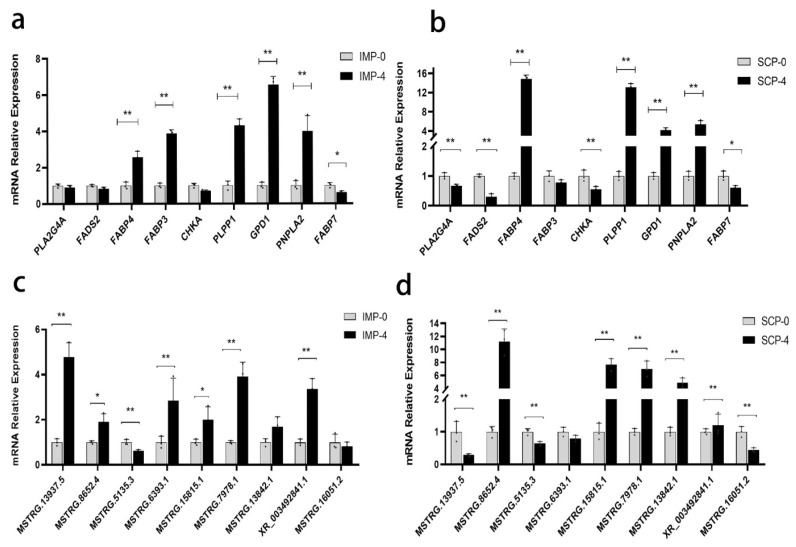
Verification of mRNA and lncRNA expression levels for total transcriptome differences in IMP and SCP. (a) Verification of mRNA expression levels before and after IMP induction. (b) Verification of mRNA expression levels before and after SCP induction. (c) Verification of lncRNA expression levels before and after IMP induction. (d) Verification of lncRNA expression levels before and after SCP induction. (n = 3, * p≤0.05 and ** p≤0.01). IMP, intramuscular preadipocytes; SCP, subcutaneous preadipocytes; lncRNA, long non-coding RNA.

**Figure 8:**
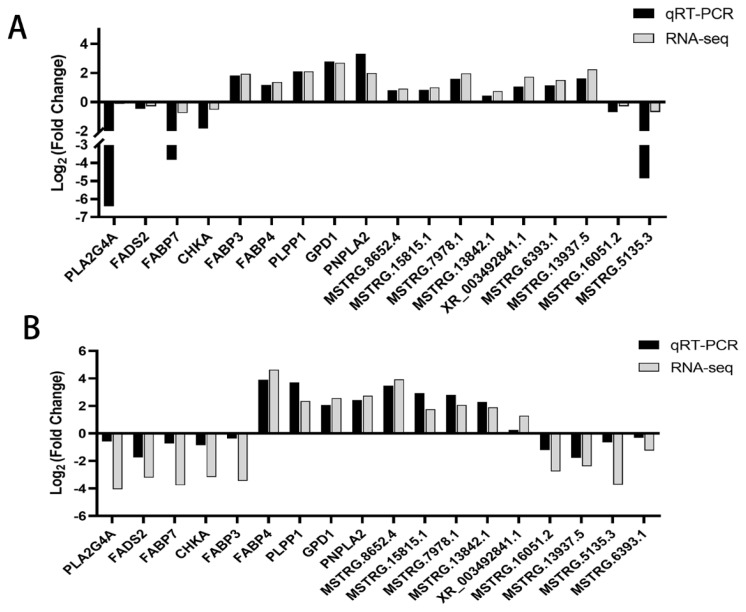
Expressions of 9 genes and 9 lncRNAs were validated by qRT-PCR or RNA-seq. (A) In IMP. (B) In SCP. qRT-PCR, quantitative reverse transcription-polymerase chain reaction; lncRNA, long non-coding RNA; IMP, intramuscular preadipocytes; SCP, subcutaneous preadipocytes.

In order to validate the DEGs obtained from RNA-seq, nine genes (Figures 7A, 7B) and nine lncRNAs (Figures 7C, 7D) were randomly selected for RT-qPCR to verify differential expression. The expression trends of these genes were consistent with the transcriptome sequencing results, indirectly indicating the confidence of the transcriptome sequencing results (Figures 8A, 8B).

## DISCUSSION

In the last century, the focus of livestock and poultry breeding has been on improving economic traits and achieving remarkable results. However, when the growth rate is increased, it often causes obesity in the body, reduces feed conversion efficiency, and reduces carcass quality, resulting in adverse effects [18,26]. This has created major drawbacks and reduced the economic value of animal husbandry. Therefore, excessive fat deposition in ducks is the primary research focus of the duck industry [28]. The fat traits of meat ducks are affected by genetic, nutritional and environmental factors, and the specific effects that selection can have on lipid distribution [27].

Specific regulatory mechanisms exist for fat deposition in different adipose tissuees [27]. Intramuscular preadipocyte, the fat deposited in muscle, is preferred because it improves meat quality [29,30]. On the contrary, excessive SCP reduces the proportion of lean meat and affects the overall meat quality. In this study, muscle and subcutaneous adipocytes were cultured in differentiation medium for 4 days, and lipid droplets gradually accumulated, but there were differences in fat-forming capacity. There were more lipid droplets in subcutaneous adipocytes than in intramuscular adipocytes. Therefore, the collected IMP and SCP were used as research objects, and adipoblast models before and 4 days after induction were constructed in vitro, and the genes of SCP-0, SCP-4, IMP-0, and IMP-4 were identified by whole transcriptome sequencing technology, and the differentially expressed lncRNAs and mRNAs of precursor adipocytes from the two sites before and after induction were detected, and their biological functions were statistically analyzed. It is hoped that by comparative analysis of mRNA and non-coding RNA from preadipocytes at different sites before and after induction, key genes and regulatory pathways that are re-associated with lipid metabolism can be screened.

To better understand the regulatory network controlling intramuscular adipocytes and subcutaneous adipocytes, we analyzed the expression profiles of lncRNAs and mrna in SCP-0, SCP-4, IMP-0, and IMP-4 for the first time by using RNA-seq. A total of 274 up-regulated DEGs and 642 down-regulated DEGs were identified between the IMP-0 and IMP-4 groups. 1,426 DEGs (342 up-regulated and 1,084 down-regulated) were found between the SCP-0 and SCP-4 groups. In both IMP-0-vs-IMP-4 and SCP-0-vs-SCP-4 groups, far more genes were down-regulated than up-regulated. But the differential genes screened in the SCP-0-vs-SCP-4 group were about six times more than in IMP-0-vs-IMP-4. This may be due to the slower differentiation of intramuscular fat precursor cells, which may preferentially repress proliferation-related genes early on in preparation for subsequent lipid accumulation. Subcutaneous fat differentiates more rapidly and initiates lipid synthesis early (e.g., *FASN*, *ACACA* upregulation). Intramuscular adipocytes are more dependent on inflammation-related signaling (arachidonic acid) and slow PPAR pathway activation before and after induction, possibly reflecting their dual metabolic-endocrine role. In contrast, subcutaneous adipocytes, before and after induction, preferentially establish lipid storage functions by rapidly switching off the Wnt pathway and activating energy metabolism. These differences may stem from the different developmental origins of the cells at the two sites, such as IMP derived from MSCs and SCP from perivascular progenitor cells. It may also be due to or differences in microenvironmental signaling.

In addition, through PPI enrichment analysis, we found that the IMP-0-vs-IMP-4 group network was sparser, suggesting that the regulation of the early stage of intramuscular adipose differentiation is relatively specific and more dependent on a few key genes, related to growth factor signaling (MET/PDGFB) and lipolytic inhibition (PNPLA2) that work together to promote adipose precursor cell colonization and early differentiation. In contrast, the SCP-0-vs-SCP-4 group network is broadly involved, suggesting that subcutaneous adipose differentiation involves more complex synergistic regulation of multiple pathways, such as metabolism and signaling crossover (Wnt inhibition, lipid metabolism activation, and inflammatory signaling), to accommodate rapid subcutaneous adipose expansion. Downregulation of WNT5A deregulates the inhibition of differentiation, whereas FABP3 and PLA2G4A synergistically enhance lipid synthesis and local signaling.

Furthermore, the lncRNA-mRNA co-expression network can directly show interactions at the entire transcriptional level. Based on the location of genes in the co-expression network, key genes that may play an important role in lipid metabolism can be effectively inferred and screened. In this study, a series of key genes and lncRNAs, such as *FABP7*, *PNPLA2*, MSTRG.6393.1, and MSTRG.13937.5, were listed by analyzing the differences in co-expression networks and the changes in ligation abundance.

Fatty acid binding proteins (FABPs) play an important role in coordinating lipid transport, metabolism and responses in different tissues and organs of different species. They promote fatty acid solubilization, transport and metabolism, and regulate tissue- and cell-specific lipid responses [31]. *FABP3* is known as muscle-cardiac FABP and is primarily involved in muscle lipid uptake and oxidation [31]. *FABP4* is known as adipocyte FABP and is found in abundance in adipocytes [31]. *FABP7* is known as brain FABP [31] and can affect gene expression through activation of the peroxisome proliferation-activated receptor, which influences transcription and fatty acid metabolism [32]. *PLA2G4A*, also known as cPLA2 or cPLA2α. It is a positive regulator of adipogenesis that promotes the proliferation of precursor adipocytes and facilitates adipogenesis, and also participates in the expansion of adipose tissue and deposition of neutral lipids [33]. Fatty acid desaturase 2 (*FADS2*) is an endoplasmic reticulum membrane-bound protein that is involved in the biosynthesis of PUFAs [34] and also plays an important role in adipogenesis [35,36]. Fatty triglyceride (TG) lipase (*PNPLA2*, also known as *ATGL*) is a novel TG lipase that specifically removes the first fatty acid from the TG molecule, thereby making a significant difference in the production of free fatty acids and diglycerides [37]. *PNPLA2* is a key enzyme for the intracellular hydrolysis of stored TGs and determines fatty acid signaling through *PPARα*, which is key to lipolysis [38,39].

Finally, we randomly selected 9 differentially expressed mRNAs and lncRNAs to verify their expression levels by qRT-PCR. And the quantitative results were consistent with the sequencing results. Interestingly, these key genes and lncRNAs were differentially regulated before and after induction of adipocytes from the two sites. Some factors were differentially regulated, such as *FABP3*, MSTRG.13937.5, and MSTRG.6393.1, which significantly increased expression in intramuscular adipocytes but were significantly downregulated in subcutaneous adipocytes after 4 days of induction. Some factors were specifically regulated, *CHKA*, *PLA2G4A*, *FADS2*, MSTRG.13842.1, MSTRG.16051.2 and MSTRG.13842.1 were significantly down-regulated only in subcutaneous adipocytes.

These key genes and lncRNAs can provide potential targets for fat deposition and metabolism-related functions in adipose tissue at two different sites, and then guide the production of intramuscular fat and subcutaneous fat in meat ducks, and improve meat quality in a targeted manner. These findings lay a theoretical foundation for the subsequent study of fat deposition differences in different parts of ducks. These findings can provide potential targets for fat deposition and metabolism in two different parts of adipose tissue, and then purposefully guide the growth of intramuscular fat and subcutaneous fat in meat ducks, and improve meat quality according to needs. Next, on the basis of bioinformatics analysis, the functions of these key factors will be further verified in broiler duck experiments, and the role of lncRNA-mRNA in the development of intramuscular fat and subcutaneous fat will be further studied, in order to provide new ideas and scientific basis for the targeted development of different adipose tissues.

## CONCLUSION

In this study, we focused on lipid droplets in intramuscular and subcutaneous adipocytes, and after 4 days of incubation in differentiation medium, we found that adipocytes from the two sites had differences in their lipogenic capacity. Subsequently, we explored the differences between intramuscular adipose precursor cells (IMP) and subcutaneous adipose precursor cells (SCP) before and after induction of differentiation by whole transcriptome sequencing. The BP and signaling pathways of some differentially expressed lncRNAs and mRNAs were related to fat synthesis and metabolism. The results showed that different lncRNAs occupied a central position in the co-expression network in the IMP-0-vs-IMP-4 and SCP-0-vs-SCP-4 groups, respectively. Because of being differentially regulated, some differential factors were significantly increased in expression in intramuscular adipocyte induction while significantly downregulated in subcutaneous adipocyte induction, such as *FABP3*, MSTRG.13937.5, and MSTRG.6393.1. Meanwhile, there were also some factors that were specifically regulated, *CHKA*, *PLA2G4A*, *FADS2*, MSTRG.13842.1, MSTRG.16051.2 and MSTRG.13842.1 were significantly downregulated only in subcutaneous adipocytes. This suggests that these lncRNAs and their target genes may play important roles in intramuscular fat and subcutaneous fat deposition.

## Figures and Tables

**Table 1:** Primer sequences of qRT-PCR

Gene	Genebank ID	Product (bp)	Annealing temperature (°C)
CHKA	F: TGGTTCTGGAGAGCGTTATGT R: CATTTTCTCGGCGATTTCTGC	151	60
FABP4	F: ACTGGGCCAGGAATTTGACG R: CTCGTGGAAGTGACGCCTT	165	59
FABP3	F: TGGAGTTCGATGAGACAACAGC R: CTCTTGCCCGTCCCATTTCTG	60	60
FABP7	F: GCACATTCAAGAACACGGAGA R: CACATCACCAAAAGTAAGGGTCA	181	59
PLPP1	F: GGCAGGTTGTCCTTCTATTCAG R: CAGTGTGGGGCGTAAGAGT	151	60
PNPLA2	F: ATGGTGGCATTTCAGACAACC R: CGGACAGATGTCACTCTCGC	70	60
PLA2G4A	F: ATGGATGAAACTCTAGGGACAGC R: CTGGGCATGAGCAAACTTCAA	116	60
FADS2	F: TGACCGCAAGGTTTACAACAT R: AGGCATCCGTTGCATCTTCTC	140	61
GPD1	F: CCAGGGACAACTCCTGAAAGA R: TTGGCGAAGGCTATCATCTCC	116	60
GAPDH	F: AGATGCTGGTGCTGAATACG R: CGGAGATGATGACACGCTTA	116	60
MSTRG.13937.5	F: AAACCCAGCACGGAAGGAAA R: GGCTTCGTTAGGTGGGAACA	90	60
MSTRG.8652.4	F: TGTGGTGGCAAGGGTTTAGG R: CTGGCACCAATCTCTCTCCC	125	60
MSTRG.5135.3	F: GAGAAGGGGAGCACTGACAC R: CAATGAGACCACCCCACCTC	121	60
MSTRG.6393.1	F: AAACCCAGCACGGAAGGAAA R: GGCTTCGTTAGGTGGGAACA	144	60
MSTRG.15815.1	F: GGCCCTGGTCTCATGTTTTCT R: GGGTACGAGTGCAAGGTTAGT	81	60
MSTRG.7978.1	F: TTGGAGCGGGAGATTGTGTC R: GTGTGTCTCATTCTGCTGGC	141	59
MSTRG.13842.1	F: GTTGCTGCGAGTGGTTCAAG R: GCTGCTGGAGGATCAGTTGT	114	60
XR_003492841.1	F: CAAATGCGACCTTGCCTCAC R: AGCCCACGGTTTCCTGTTTT	163	60
MSTRG.16051.2	F: CGGGGGCTTTTGTCTATCCT R: TCGGCTGGTTGGGAAGATTT	127	59

qRT-PCR, quantitative reverse transcription-polymerase chain reaction.

**Table 2:** Sequencing data filtering statistics

Sample	Raw datas	Clean data (%)	Average rate
IMP-0			
IMP-0-1	71224750	71108066 (99.84%)	99.80%
IMP-0-2	94537958	94350452 (99.80%)	
IMP-0-3	87050342	86858322 (99.78%)	
IMP-4			
IMP-4-1	80808624	80632426 (99.78%)	99.81%
IMP-4 2	80270898	80126204 (99.82%)	
IMP-4-3	70239768	70117364 (99.83%)	
SCP-0			
SCP-0-1	74805746	74630234 (99.77%)	99.78%
SCP-0-2	81719228	81545898 (99.79%)	
SCP-0-3	108349258	108112258 (99.78%)	
SCP-4			
SCP-4-1	85468506	85298604 (99.80%)	99.80%
SCP-4-2	100597660	100379024 (99.78%)	
SCP-4-3	78018698	77872136 (99.81%)	

IMP, intramuscular preadipocytes; SCP, subcutaneous preadipocytes.

**Table 3:** The top 10 lncRNAs of IMP-0-vs-IMP-4 were sorted by connectivity degree

lncRNA id of IMP-0-vs-IMP-4	Content
MSTRG.7877.3	170
MSTRG.6393.1	168
MSTRG.13937.5	167
MSTRG.2868.1	162
XR_003492841.1(LOC113840110)	144
MSTRG.10341.22	135
MSTRG.10341.20	111
MSTRG.5135.3	91
MSTRG.7978.1	61
MSTRG.3461.1	28

lncRNA, long non-coding RNA; IMP, intramuscular preadipocytes.

**Table 4:** The top 10 lncRNAs of SCP-0-vs-SCP-4 were sorted by connectivity degree

lncRNA id of SCP-0-vs-SCP-4	Content
MSTRG.8652.4	293
XR_003499406.1 (LOC113844692)	290
MSTRG.15586.1	181
MSTRG.6393.1	175
MSTRG.16051.2	108
MSTRG.13937.5	67
MSTRG.13842.1	62
XR_003493886.1 (LOC113841287)	57
XR_217450.4 (LOC101790536)	51
MSTRG.15815.1	49

lncRNA, long non-coding RNA; SCP, subcutaneous preadipocytes.

## SUPPLEMENTARY MATERIAL

**Supplement 1**. Base Information Statistics.

**Supplement 2**. Comparison of Ribosome Statistics.

**Supplement 3**. Table of differential mRNAs analysis in the IMP-0-vs-IMP-4 group results.

**Supplement 4**. DemRNAs of SCP-0-vs-SCP-4-.

**Supplement 5**. MCODE information for PPI networks of the IMP-0-vs-IMP-4 group.

**Supplement 6**. MCODE information for PPI networks of the SCP-0-vs-SCP-4 group.

**Supplement 7**. Table of differential lncRNAs analysis in the IMP-0-vs-IMP-4 group results.

**Supplement 8**. Table of differential lncRNAs analysis in the SCP-0-vs-SCP-4 group results.

**Supplement 9**. A lncRNA-mRNA co-expression network of the IMP-0-vs-IMP-4 group.

**Supplement 10**. A lncRNA-mRNA co-expression network of the SCP-0-vs-SCP-4 group.
