# Supplementary material for: Identification of genes and long non-coding RNAs for intramuscular and subcutaneous fat deposition in ducks by transcriptome analysis
Source: Anim Biosci. 2025 Aug 12;39(1):250268. doi: 10.5713/ab.25.0268 (PMC12754461; doi:10.5713/ab.25.0268)
Supplement: Supplementary file 1 [file ab-25-0268-Supplementary-1.pdf]

Supplement 1. Base Information Statistics

Sample	RawData(bp)	BF_Q20(%)	BF_Q30(%)	BF_GC(%)	CleanData(bp)	AF_Q20(%)	AF_Q20 Average(%)	AF_Q30(%)	AF_Q30 Average(%)	AF_N(%)	AF_GC(%)
IMP0-1	10683712500	10440043298 (97.72%)	9966078578 (93.28%)	4643089457 (43.46%)	10629353451	10394489678 (97.79%)	97.78%	9925229730 (93.38%)	93.41	37940 (0.00%)	4613822396 (43.41%)
IMP0-2	14180693700	13858899678 (97.73%)	13244694022 (93.40%)	6176931119 (43.56%)	14113607619	13804358115 (97.81%)		13195736990 (93.50%)		344259 (0.00%)	6141587239 (43.52%)
IMP0-3	13057551300	12749874590 (97.64%)	12173961056 (93.23%)	5707076568 (43.71%)	12981339458	12687097053 (97.73%)		12117672963 (93.35%)		47835 (0.00%)	5666364264 (43.65%)
IMP4-1	12121293600	11814377911 (97.47%)	11247577249 (92.79%)	5280756102 (43.57%)	12051134487	11756609677 (97.56%)	97.79%	11195822286 (92.90%)	93.46666667	43958 (0.00%)	5243395038 (43.51%)
IMP4-2	12040634700	11784230639 (97.87%)	11292722744 (93.79%)	5224078765 (43.39%)	11974760527	11728870851 (97.95%)		11242727394 (93.89%)		43213 (0.00%)	5189972073 (43.34%)
IMP4-3	10535965200	10303479198 (97.79%)	9853828016 (93.53%)	4579410739 (43.46%)	10487765058	10263656437 (97.86%)		9818090980 (93.61%)		38147 (0.00%)	4553871409 (43.42%)
SCP0-1	11220861900	10923516243 (97.35%)	10402100916 (92.70%)	4953329684 (44.14%)	11158628035	10873131920 (97.44%)	97.55%	10357090893 (92.82%)	92.97666667	82053 (0.00%)	4920973996 (44.10%)
SCP0-2	12257884200	11979015334 (97.72%)	11450776047 (93.42%)	5396840128 (44.03%)	12200728737	11932952490 (97.81%)		11409783133 (93.52%)		44096 (0.00%)	5366522149 (43.99%)
SCP0-3	16252388700	15814294635 (97.30%)	15031757354 (92.49%)	7152926282 (44.01%)	16172141960	15749524198 (97.39%)		14974418735 (92.59%)		216562 (0.00%)	7110540813 (43.97%)
SCP4-1	12820275900	12503222077 (97.53%)	11918964999 (92.97%)	5633199999 (43.94%)	12765158875	12458938674 (97.60%)	97.56%	11879555220 (93.06%)	92.95666667	180654 (0.00%)	5603874411 (43.90%)
SCP4-2	15089649000	14664781439 (97.18%)	13915783448 (92.22%)	6615271790 (43.84%)	15025502683	14614726291 (97.27%)		13872047355 (92.32%)		216206 (0.00%)	6581092545 (43.80%)
SCP4-3	11702804700	11437529337 (97.73%)	10929338667 (93.39%)	5214850015 (44.56%)	11644524149	11389275080 (97.81%)		10886115860 (93.49%)		43129 (0.00%)	5183594479 (44.52%)

Description: Sample: name of the sample; RawData(bp): total number of bases in the downlinked data (in bp); BF: Before Filter, information about the bases in the sample

before filtering; BF_Q20(%): number of sequenced bases with quality value of Q20 or above and its percentage in RawData; BF_Q30(%): number of bases with sequencing base quality value of Q30 or above and its percentage in RawData; CleanData(bp): total number of bases with high quality data after filtering (in bp); AF: After Filter, filtered sample base information; AF_Q20(%): number of bases with sequencing base quality value of Q20 or above and its percentage in CleanData: AF_Q30(%): Number of sequenced bases with quality value of Q30 or above and percentage of CleanData; AF_N(%): Number of single-end reads containing N bases and percentage of CleanData; AF_GC(%): GC ratio of sequenced bases after filter.
