# Supplementary material for: Identification of genes and long non-coding RNAs for intramuscular and subcutaneous fat deposition in ducks by transcriptome analysis
Source: Anim Biosci. 2025 Aug 12;39(1):250268. doi: 10.5713/ab.25.0268 (PMC12754461; doi:10.5713/ab.25.0268)
Supplement: Supplementary file 2 [file ab-25-0268-Supplementary-2.pdf]

Supplement 2. Comparison of Ribosome Statistics

Sample	clean_reads	Mapped_Reads(%)	Averae	Unmapped_Reads(%)
IMP0-1	71224750	190772 (0.27%)	0.26%	70917294 (99.73%)
IMP0-2	94537958	226704 (0.24%)		94123748 (99.76%)
IMP0-3	87050342	235250 (0.27%)		86623072 (99.73%)
IMP4-1	80808624	277234 (0.34%)	0.36%	80355192 (99.66%)
IMP4-2	80270898	296206 (0.37%)		79829998 (99.63%)
IMP4-3	70239768	255032 (0.36%)		69862332 (99.64%)
SCP0-1	74805746	198786 (0.27%)	0.25%	74431448 (99.73%)
SCP0-2	81719228	174702 (0.21%)		81371196 (99.79%)
SCP0-3	108349258	285020 (0.26%)		107827238 (99.74%)
SCP4-1	85468506	231638 (0.27%)	0.33%	85066966 (99.73%)
SCP4-2	100597660	304316 (0.30%)		100074708 (99.70%)
SCP4-3	78018698	324500 (0.42%)		77547636 (99.58%)

Description: Sample: sample name; clean_reads: number of high-quality reads; Mapped_Reads(%): number of reads that matched the ribosomes of the species and the percentage of reads (based on cleand reads); Unmapped_Reads(%): number of reads that did not match the ribosomes and the percentage of reads (based on cleand reads).
