# Supplementary material for: Identification of genes and long non-coding RNAs for intramuscular and subcutaneous fat deposition in ducks by transcriptome analysis
Source: Anim Biosci. 2025 Aug 12;39(1):250268. doi: 10.5713/ab.25.0268 (PMC12754461; doi:10.5713/ab.25.0268)
Supplement: Supplementary file 3 [file ab-25-0268-Supplementary-3.pdf]

Supplement 3. Table of differential mRNAs analysis in the IMP-0-vs-IMP-4 group results

id	IMP-0_fpkm_mean	IMP-4_fpkm_mean	log2(fc)	PValue	FDR	Symbol
ncbi_101790688	88.23	1.176666667	-6.228491697	0	0	CLDN5
ncbi_101792633	1.163333333	17.85666667	3.940126355	0	0	SLC15A2
ncbi_101792740	3.546666667	83.95	4.564994675	0	0	ACKR3
ncbi_101793407	0.95	18.27666667	4.265931649	0	0	TENT5C
ncbi_101793749	277.8866667	6.706666667	-5.372756904	0	0	Mmp7
ncbi_101793837	23.17666667	253.37	3.45050072	0	0	TNNT3
ncbi_101793840	35.61666667	227.68	2.67638316	0	0	ZNF106
ncbi_101793981	133.24	1341.153333	3.331375025	0	0	GSN
ncbi_101794671	2.116666667	20.83	3.298796843	0	0	adam22
ncbi_101795399	42.53333333	330.99	2.960121802	0	0	EFEMP1
ncbi_101796141	3.536666667	77.16333333	4.447453311	0	0	Pdk4
ncbi_101797391	2.813333333	32.56666667	3.533043658	0	0	SLC37A1
ncbi_101799662	6.966666667	104.05	3.900664618	0	0	COL4A5
ncbi_101799687	10.12	58.02666667	2.519506766	0	0	COL14A1
ncbi_101799717	23.07666667	95.3	2.046041367	0	0	PIK3R1
ncbi_101800228	3.276666667	54.76	4.062821626	0	0	--
ncbi_101801623	10.36666667	79.93666667	2.946905334	0	0	TEX2
ncbi_101801733	4.923333333	32.05	2.702617031	0	0	Stab1
ncbi_101802755	9.4	267.2466667	4.829367386	0	0	ACTA1
ncbi_101803155	8.05	61.63666667	2.936728154	0	0	SLC40A1
ncbi_101804140	0.44	22.65666667	5.686288288	0	0	Igfn1
ncbi_110353953	0.406666667	30.86333333	6.245903334	0	0	MYH1B
ncbi_101801672	3.873333333	48.55	3.647823728	1.01E-296	5.07E-294	STC2
ncbi_101794506	3.606666667	16.40333333	2.185251017	3.69E-269	1.78E-266	KLF15
ncbi_101796074	74.06666667	12.23666667	-2.597613797	4.59E-264	2.14E-261	FLNB
ncbi_101799557	38.33333333	165.4666667	2.109869254	1.51E-257	6.80E-255	PLPP1
ncbi_101795823	28.23333333	4.85	-2.541342816	2.49E-243	1.05E-240	ANLN
ncbi_101791516	121.5	25.61666667	-2.24580165	3.29E-226	1.19E-223	SLC2A3
ncbi_101795892	1.173333333	19.80333333	4.077058455	1.86E-224	6.55E-222	MYOM2

ncbi_101800546	3.466666667	14.34333333	2.048759312	4.81E-223	1.65E-220	SLC16A7
ncbi_101803788	12.93666667	84.55333333	2.708395699	4.00E-219	1.30E-216	rab18b
ncbi_101794773	41.32333333	4.53	-3.189373678	6.04E-218	1.92E-215	FBLN1
ncbi_101800897	10.59333333	72.11	2.767042718	7.55E-217	2.34E-214	DHRS7
ncbi_101800675	8.606666667	38.19666667	2.149920243	1.07E-209	3.24E-207	HACD1
ncbi_101795165	0.56	35.86	6.000804851	5.36E-208	1.59E-205	MYOT
ncbi_101803718	6.8	84.46	3.634661596	7.89E-203	2.29E-200	Sln
ncbi_101802030	10.03333333	77.67333333	2.952618394	2.13E-190	5.55E-188	MYOZ1
ncbi_101795864	21.18666667	89.77333333	2.08313034	5.75E-187	1.39E-184	Kcne4
ncbi_101801901	16.25	70.75666667	2.122426366	3.98E-184	9.43E-182	SRL
ncbi_101800940	37.6	4.336666667	-3.116074201	5.66E-182	1.29E-179	CCR8
MSTRG.3206	0.026666667	3.636666667	7.091435386	1.14E-179	2.51E-177	--
ncbi_101795161	20.44333333	84.4	2.045612549	3.73E-176	7.97E-174	EEF1A2
ncbi_101790839	5.443333333	28.03666667	2.364752545	2.05E-166	3.92E-164	Itih5
ncbi_101797300	1.96	32.19333333	4.037836403	2.48E-166	4.69E-164	CKMT2
ncbi_101797741	2.45	21.57	3.138172522	7.91E-164	1.43E-161	CLDN1
ncbi_101796901	31.59666667	0.356666667	-6.469052166	6.54E-161	1.15E-158	ASPN
ncbi_101802260	5.546666667	25.50666667	2.20118144	3.97E-160	6.82E-158	SMOC2
ncbi_101798493	49.92333333	11.60333333	-2.105174951	1.63E-158	2.65E-156	Sdc1
ncbi_101793597	6.283333333	0.283333333	-4.470957872	4.77E-157	7.67E-155	IGF1
ncbi_101803905	2.676666667	22.34	3.061119794	9.89E-156	1.57E-153	SLC25A12
ncbi_101796257	4.9	34.23333333	2.804548121	9.80E-153	1.50E-150	GATM
ncbi_101791751	34.12333333	7.403333333	-2.204511691	1.22E-152	1.85E-150	UHRF1
ncbi_101790786	11.2	54.21333333	2.275148982	8.22E-152	1.22E-149	PLBD1
ncbi_101802143	0.39	18.18	5.542734265	3.36E-149	4.87E-147	ZBTB16
ncbi_101789696	39.63333333	6.963333333	-2.508864318	1.60E-147	2.26E-145	CD200R1A
ncbi_101792370	193.72	36.87333333	-2.393323162	1.14E-146	1.59E-144	LY6E
ncbi_101799485	25.2	4.696666667	-2.423714623	4.12E-145	5.59E-143	ITGA4
ncbi_101793083	35.64	3.366666667	-3.404104545	2.71E-139	3.56E-137	NRSN1
ncbi_113843234	15.03333333	84.5	2.490786409	1.62E-137	2.01E-135	LAMA2
ncbi_101794165	27.32333333	6.43	-2.087242855	1.04E-136	1.28E-134	FAM129A
ncbi_101794665	53.96333333	9.94	-2.44066171	8.99E-133	1.07E-130	RRM2

ncbi_101803448	6.566666667	29.53	2.168948228	1.01E-132	1.18E-130	mmp11
ncbi_101805178	52.53	10.19	-2.365987533	8.77E-131	9.94E-129	KPNA2
ncbi_101794326	1.886666667	10.61333333	2.491966378	7.14E-127	7.69E-125	Homer2
ncbi_101795125	191.6133333	37.34666667	-2.359146566	3.07E-126	3.28E-124	RIHB
ncbi_101801129	13.96333333	1.913333333	-2.867483242	9.53E-125	9.94E-123	FAM198B
ncbi_101793310	35.92	7.8	-2.203241321	9.58E-124	9.91E-122	Vcp
ncbi_101792047	1.436666667	6.703333333	2.222153307	6.26E-123	6.43E-121	CAMK1D
ncbi_101793940	11.41	0.603333333	-4.24119969	7.13E-121	7.04E-119	Hcls1
ncbi_101791912	3.913333333	22.97666667	2.553699607	2.77E-120	2.69E-118	Egfl6
ncbi_101803645	3.233333333	13.16333333	2.025430715	5.97E-118	5.56E-116	ADGRL3
ncbi_101801927	9.57	1.03	-3.215874587	9.28E-118	8.58E-116	GAS2L3
ncbi_113841128	122.95	23.17666667	-2.407326741	2.05E-117	1.88E-115	--
ncbi_101796805	0.726666667	7.08	3.284383726	3.26E-115	2.91E-113	MTUS2
ncbi_101800593	16.30666667	2.133333333	-2.934280594	3.48E-115	3.09E-113	PTPRC
ncbi_101790561	0.176666667	3.246666667	4.199857508	6.61E-113	5.59E-111	CLIC5
ncbi_113842028	2.773333333	60.05666667	4.43663147	5.73E-112	4.76E-110	Myh8
ncbi_101796345	25.74	5.643333333	-2.189392581	8.81E-112	7.27E-110	EPAS1
ncbi_101800132	0.56	6.716666667	3.584246701	3.08E-109	2.51E-107	RNF152
ncbi_110351183	104.3933333	1.766666667	-5.884855914	3.81E-108	3.09E-106	CCL4
ncbi_101800657	12.80333333	1.203333333	-3.411411222	1.19E-104	9.44E-103	TEC
ncbi_101804467	3.756666667	0.886666667	-2.082989365	5.07E-104	3.96E-102	SYNE1
ncbi_101803881	34.25	8.436666667	-2.021358981	1.71E-103	1.32E-101	Lyn
ncbi_101803192	21.96666667	3.703333333	-2.568419649	1.05E-102	7.94E-101	SMC4
ncbi_101790193	110.0433333	22.30333333	-2.302740498	3.24E-102	2.43E-100	CTGF
ncbi_101796234	1.766666667	10.00333333	2.501379054	1.01E-100	7.33E-99	Avpr1a
ncbi_101800870	6.096666667	32.91333333	2.43257957	7.54E-99	5.43E-97	SYNPO2L
ncbi_101803383	1.413333333	6.116666667	2.113643893	2.14E-95	1.46E-93	IGSF10
ncbi_101800480	2.91	13.08333333	2.168639095	3.87E-92	2.54E-90	ST8SIA2
ncbi_101795842	8.446666667	0.49	-4.107528464	1.12E-91	7.26E-90	QNR-71
ncbi_101804335	10.53666667	1.236666667	-3.090889943	1.14E-91	7.30E-90	PTPRE
ncbi_101799119	80.70666667	12.48333333	-2.692684632	1.50E-91	9.55E-90	SERPINB2
ncbi_101792368	26.04	5.85	-2.154220919	4.57E-90	2.89E-88	CCNA2

ncbi_101795349	2.796666667	20.16	2.849715424	5.82E-89	3.66E-87	KCNJ8
ncbi_101793285	51.90333333	3.676666667	-3.819356904	2.78E-88	1.72E-86	FABP7
ncbi_101792309	1.38	8.156666667	2.563311429	1.20E-87	7.40E-86	Il13ra2
ncbi_101798494	16.11	3.82	-2.076311951	1.05E-86	6.42E-85	DIO2
ncbi_101799428	0.816666667	6.376666667	2.964983219	3.42E-84	2.01E-82	SLITRK1
ncbi_101800942	9.386666667	1.363333333	-2.783474586	1.49E-83	8.70E-82	RPS6KA
ncbi_113839853	83.69666667	14.16333333	-2.563009324	6.30E-83	3.64E-81	TAGLN
ncbi_101794431	18.94666667	4.176666667	-2.18152014	7.93E-83	4.56E-81	RGS10
ncbi_101792719	12.01	2.946666667	-2.027080377	6.19E-81	3.48E-79	WDFY4
ncbi_101791356	1.606666667	22.30333333	3.795116793	6.50E-81	3.64E-79	GHOX-7
ncbi_101801298	0.776666667	5.26	2.759695345	2.15E-80	1.19E-78	NEB
ncbi_101802187	38.1	5.59	-2.76887081	2.24E-80	1.24E-78	Tubb2b
ncbi_101796353	6.793333333	1.21	-2.489112598	2.41E-80	1.33E-78	NUP210
ncbi_101802218	0.35	4.93	3.816160819	3.29E-80	1.80E-78	COL20A1
ncbi_101800607	13.01	52.73666667	2.019185423	6.30E-79	3.42E-77	LY86
ncbi_101792607	3.716666667	18.54666667	2.31907871	4.52E-78	2.42E-76	NFKBIZ
ncbi_101802858	0.97	3.9	2.007417472	1.07E-77	5.64E-76	FREM2
ncbi_101804721	33.52666667	0.586666667	-5.836624219	1.75E-77	9.10E-76	LYZ
ncbi_101802167	26.28	0.21	-6.967432138	3.31E-77	1.70E-75	ATP6V0D2
ncbi_101794181	2.72	21.57333333	2.98757055	8.16E-77	4.15E-75	MMP27
ncbi_101796497	3.643333333	36.37333333	3.319550243	1.25E-76	6.32E-75	Pnpla2
ncbi_101794917	5.39	0.703333333	-2.938004775	1.30E-76	6.58E-75	FGD5
ncbi_113842416	11.49666667	0.52	-4.466560195	1.54E-76	7.77E-75	CSTB
ncbi_101804723	0.52	2.92	2.489384841	1.87E-76	9.38E-75	Irs2
ncbi_101792362	82.24666667	14.96333333	-2.458525614	3.71E-75	1.85E-73	SPP1
ncbi_101803695	13.86666667	2.743333333	-2.337619193	3.50E-73	1.73E-71	BUB1
ncbi_101803686	37.81	7.51	-2.331883036	1.98E-72	9.67E-71	CDK1
ncbi_101793887	9.93	1.403333333	-2.822935985	3.79E-71	1.81E-69	CSF2RB
ncbi_101800857	0.523333333	5.353333333	3.354635429	4.06E-70	1.90E-68	ADRB2
ncbi_101804547	0.346666667	3.033333333	3.129283017	1.05E-69	4.87E-68	SFMBT2
ncbi_101796813	18.85333333	3.49	-2.433520678	1.39E-69	6.42E-68	MPP6
ncbi_101796982	59.26	14.74333333	-2.006995885	1.94E-69	8.90E-68	CYGB

ncbi_101794359	3.286666667	0.106666667	-4.945443836	3.76E-69	1.72E-67	Il1r1
ncbi_101799814	6.4	0.45	-3.830074999	5.99E-69	2.71E-67	TMEM121
ncbi_101799520	8.233333333	0.813333333	-3.339557989	1.87E-68	8.35E-67	Myo1f
ncbi_101792171	14.36333333	1.713333333	-3.067512834	4.49E-68	2.00E-66	CYBB
ncbi_101800659	14.71	3.583333333	-2.037423087	9.81E-68	4.35E-66	PTPN5
ncbi_101800981	5.45	26.07333333	2.2582469	1.39E-67	6.13E-66	Limd2
ncbi_101790286	8.083333333	1.373333333	-2.557268505	2.81E-67	1.22E-65	BUB1B
ncbi_101796531	4.393333333	27.58	2.650234587	1.16E-66	5.05E-65	SH3BGR
ncbi_101798214	4.596666667	0.123333333	-5.219953376	4.51E-66	1.92E-64	MMRN2
ncbi_101804889	10.95	0.403333333	-4.762814418	8.68E-66	3.69E-64	MATK
ncbi_101802443	1.68	8.496666667	2.338435735	2.58E-64	1.07E-62	SLC6A2
ncbi_101800956	4.413333333	0.143333333	-4.944422652	4.65E-64	1.92E-62	lrmp
ncbi_101792564	34.10666667	3.016666667	-3.499026567	5.66E-63	2.29E-61	LY6E
ncbi_101790142	11.58666667	0.213333333	-5.763212367	9.13E-62	3.63E-60	--
ncbi_101802409	4.35	0.406666667	-3.419096754	6.39E-61	2.49E-59	TFEC
ncbi_101796109	0.576666667	3.436666667	2.57520039	6.93E-61	2.70E-59	COL24A1
ncbi_101789862	24.25	5.756666667	-2.074679165	1.19E-60	4.60E-59	LCP1
ncbi_101792619	5.233333333	0.363333333	-3.848364519	1.23E-60	4.75E-59	TIMP4
ncbi_101805182	2.583333333	26.07333333	3.335269321	3.07E-60	1.17E-58	Rasl11b
ncbi_101804445	4.853333333	0.07	-6.115477217	1.67E-58	6.28E-57	Cd93
ncbi_101794178	0.73	4.233333333	2.535825722	2.04E-58	7.64E-57	JADE1
ncbi_113845671	22.28666667	4.086666667	-2.447184376	3.79E-58	1.41E-56	CNN2
ncbi_101798749	4.603333333	0.48	-3.261572603	4.26E-58	1.58E-56	CARD11
ncbi_101804995	1.523333333	9.13	2.583383196	1.45E-57	5.34E-56	FGFR3
ncbi_101804514	27.63333333	4.63	-2.577325502	4.55E-57	1.65E-55	CYTL1
ncbi_101793775	0.096666667	2.84	4.876728625	6.78E-57	2.45E-55	Pah
ncbi_101796476	11	2.393333333	-2.200410275	1.74E-56	6.24E-55	NDC80
ncbi_101803859	2.886666667	0.17	-4.085797873	8.28E-56	2.96E-54	Tmc3
ncbi_101803182	2.773333333	0.646666667	-2.100526876	2.00E-55	7.03E-54	FGL1
ncbi_101796639	3.796666667	21.82	2.522845855	2.03E-55	7.10E-54	TM6SF1
ncbi_101804447	3.793333333	0.333333333	-3.508428653	8.17E-54	2.80E-52	Plcb2
ncbi_106016586	0.516666667	5.71	3.466185031	3.44E-53	1.17E-51	--

ncbi_101793000	9.546666667	1.21	-2.979990039	2.28E-52	7.63E-51	MAOA
ncbi_101800187	8.333333333	1.57	-2.40812913	5.66E-52	1.88E-50	--
ncbi_101792609	0.256666667	3.146666667	3.615856509	1.71E-51	5.58E-50	GFRA4
ncbi_101800682	6.803333333	0.72	-3.240172965	3.52E-51	1.14E-49	TMEM26
ncbi_101800116	11.18666667	1.746666667	-2.679103999	4.14E-51	1.34E-49	PLK1
ncbi_101801016	0.19	5.336666667	4.811867578	7.40E-51	2.38E-49	LYPD1
ncbi_106020344	1.183333333	8.183333333	2.789832095	2.69E-50	8.58E-49	PFKM
ncbi_101803634	8.33	1.786666667	-2.221045996	2.77E-50	8.81E-49	INCENP
ncbi_101797866	6.89	1.293333333	-2.413409831	2.79E-50	8.85E-49	INPP5D
ncbi_101800620	62.07666667	0.11	-9.140403757	1.20E-49	3.76E-48	Ccl5
ncbi_101792773	21.09666667	4.666666667	-2.176550741	4.68E-49	1.45E-47	--
ncbi_101797409	7.923333333	1.563333333	-2.341482075	5.17E-49	1.59E-47	NCAPG
ncbi_101790910	11.89666667	1.413333333	-3.073383731	1.62E-48	4.88E-47	Atp6v1c2
ncbi_101797296	7.656666667	1.55	-2.304448235	3.35E-48	1.00E-46	ECT2
ncbi_101801575	0.473333333	6.693333333	3.821796434	5.63E-48	1.68E-46	Ccdc85a
ncbi_101790794	24.32333333	3.446666667	-2.819067268	7.09E-48	2.11E-46	RAC2
ncbi_101793169	3.233333333	0.236666667	-3.772093818	1.12E-47	3.31E-46	LAMP3
ncbi_101796947	6.396666667	0.46	-3.797614539	1.95E-47	5.69E-46	SYNGR3
ncbi_101794855	10.03333333	1.77	-2.502979721	2.94E-47	8.54E-46	CKAP2
ncbi_101802738	3.65	0.456666667	-2.998683072	1.21E-46	3.47E-45	CENPF
ncbi_101790659	0.386666667	3.42	3.14483402	2.91E-46	8.18E-45	NTRK3
ncbi_101803713	0.383333333	2.273333333	2.568137878	5.28E-46	1.48E-44	Cfh
ncbi_101797250	11.91	2.72	-2.130494857	5.54E-46	1.54E-44	APBB1IP
ncbi_101795367	3.13	0.576666667	-2.44035312	6.72E-46	1.87E-44	DEPDC1B
ncbi_101799127	0.663333333	3.34	2.332042173	1.31E-45	3.62E-44	PEX5L
ncbi_101790941	8.253333333	1.63	-2.340104944	1.46E-45	4.01E-44	ENTPD2
ncbi_101800379	6.143333333	1.13	-2.442698892	2.17E-45	5.95E-44	ALS2CL
ncbi_101803955	7.11	1.033333333	-2.782543845	3.09E-45	8.41E-44	ADRA2A
ncbi_101791304	11.48	2.596666667	-2.144389909	5.70E-45	1.53E-43	tor4a-a
ncbi_101791683	7.216666667	0.153333333	-5.556589354	7.42E-45	1.99E-43	TYR
ncbi_101804091	0.07	1.366666667	4.287162677	9.32E-45	2.48E-43	TRIM67
ncbi_106020632	74.32666667	15.11333333	-2.298058018	1.54E-44	4.09E-43	CSTA

ncbi_101794222	0.74	10.72666667	3.857532744	6.50E-44	1.70E-42	STIMATE
ncbi_101796233	1.463333333	6.616666667	2.176846162	2.58E-43	6.62E-42	LVRN
ncbi_101793609	0.786666667	3.17	2.010658481	2.69E-43	6.89E-42	Slc12a5
ncbi_101790483	4.91	0.236666667	-4.374794596	4.67E-43	1.19E-41	RASGRP3
ncbi_101804021	1.043333333	5.21	2.320083216	9.93E-43	2.50E-41	TMEM132C
ncbi_101790984	13.84333333	3.033333333	-2.190215421	3.23E-42	8.06E-41	ENO2
ncbi_101802521	19.56333333	2.076666667	-3.23581064	4.78E-42	1.19E-40	IL15
ncbi_101796029	4.75	0.626666667	-2.922157352	7.07E-42	1.74E-40	--
ncbi_101790919	7.32	1.596666667	-2.196780493	1.89E-41	4.63E-40	TRIM27
ncbi_101801774	0.146666667	1.626666667	3.471305719	2.78E-41	6.75E-40	Adamts20
ncbi_101799841	3.093333333	0.226666667	-3.770518154	6.16E-41	1.48E-39	SLC9A2
ncbi_101804915	1.996666667	0.18	-3.471524691	8.24E-41	1.98E-39	DSP
ncbi_101803920	6.276666667	0.743333333	-3.077917385	4.72E-40	1.11E-38	SUCNR1
ncbi_101797452	4.46	0.88	-2.341468281	5.22E-40	1.23E-38	STK10
ncbi_101804741	0.416666667	16.36333333	5.295429167	1.98E-39	4.60E-38	SLC2A5
ncbi_101802347	4.39	0.453333333	-3.275576789	3.10E-39	7.14E-38	EPCAM
ncbi_101790002	1.843333333	9.106666667	2.304606098	4.09E-39	9.36E-38	SCG5
ncbi_101799811	0.02	6.96	8.442943496	1.08E-38	2.45E-37	CDH20
ncbi_101790293	12.00666667	2.666666667	-2.170726276	2.32E-38	5.20E-37	RACGAP1
ncbi_101794037	12.81333333	0.093333333	-7.101037699	2.95E-38	6.62E-37	Acod1
ncbi_101796588	5.606666667	0.473333333	-3.566214871	4.27E-38	9.52E-37	slc51a
ncbi_101801463	7.023333333	1.05	-2.74176658	7.78E-38	1.73E-36	DEPDC1
ncbi_101802278	1.223333333	7.31	2.579053844	2.09E-37	4.58E-36	Hebp2
ncbi_101796456	0.843333333	4.336666667	2.362411672	2.10E-37	4.61E-36	Fsd2
ncbi_101794600	0.263333333	8.33	4.983354438	3.35E-37	7.28E-36	CCK
ncbi_101790001	10.99	1.716666667	-2.67850955	3.89E-37	8.40E-36	MOXD1
ncbi_101792759	6.196666667	0.486666667	-3.670486496	7.59E-37	1.63E-35	TRPC3
ncbi_101790005	0.03	5.91	7.622051819	9.64E-37	2.06E-35	PROKR1
ncbi_101805205	3.563333333	0.19	-4.229156124	1.14E-36	2.42E-35	GPR183
ncbi_101797308	5.173333333	1.18	-2.132307292	1.32E-36	2.79E-35	Spata13
ncbi_101800393	2.29	0.35	-2.709920771	1.42E-36	3.00E-35	CIT
ncbi_101800778	0.423333333	2.383333333	2.493114745	3.40E-36	7.11E-35	TENM2

ncbi_101791569	34.44	0.573333333	-5.908567173	1.04E-35	2.15E-34	CCL3
ncbi_101795751	14.17666667	2.296666667	-2.625904967	1.14E-35	2.35E-34	Cdc20
ncbi_101795495	6.453333333	0.846666667	-2.930178551	1.43E-35	2.94E-34	HEY1
ncbi_101791824	3.19	0.053333333	-5.902375114	5.29E-35	1.06E-33	TMEM71
ncbi_101803053	0.42	16.40333333	5.287455878	5.94E-35	1.19E-33	MYBPC1
ncbi_101802583	4.386666667	0.666666667	-2.718087584	2.15E-34	4.26E-33	KNL1
ncbi_101799380	3.61	0.52	-2.795415309	2.65E-34	5.21E-33	TBXAS1
ncbi_101804360	5.08	0.216666667	-4.551279374	6.78E-34	1.31E-32	Cd34
ncbi_113839738	11.25666667	1.47	-2.936891618	8.93E-34	1.72E-32	--
ncbi_113840077	1.883333333	11.41666667	2.599781215	1.34E-33	2.55E-32	ELN
ncbi_101795500	0.286666667	3.943333333	3.781969604	2.99E-33	5.62E-32	LRRC3B
ncbi_113839637	0.036666667	3.876666667	6.724203763	3.56E-33	6.70E-32	MYH1
ncbi_101794395	0.67	3.433333333	2.357376931	6.64E-33	1.23E-31	RIC3
ncbi_101793831	9.836666667	1.303333333	-2.915963408	8.52E-33	1.57E-31	RAB10
ncbi_101790494	4.993333333	1.206666667	-2.048976021	1.54E-32	2.82E-31	PIK3R6
ncbi_101801305	10.74333333	2.106666667	-2.350407725	1.78E-32	3.25E-31	SEC23A
ncbi_101793164	5.09	0.706666667	-2.848563892	2.63E-32	4.76E-31	EXO1
ncbi_101798327	0.13	2.02	3.957771765	2.87E-32	5.19E-31	WNT11
MSTRG.15966	0.326666667	2.51	2.941796211	3.26E-32	5.86E-31	ADAMTS19
ncbi_101791102	0.096666667	0.706666667	2.869939459	4.16E-32	7.44E-31	kcnk9
ncbi_101801217	7.626666667	1.793333333	-2.088408974	6.21E-32	1.10E-30	LCP2
ncbi_101801170	3.846666667	0.923333333	-2.058685343	6.64E-32	1.17E-30	KIF14
ncbi_101797742	2.173333333	0.063333333	-5.100800641	7.89E-32	1.39E-30	Drd4
ncbi_101804717	4.623333333	0.88	-2.393357953	1.06E-31	1.86E-30	Sytl2
ncbi_101791827	7.643333333	0.54	-3.823170637	2.34E-31	4.03E-30	LMCD1
ncbi_101793097	0.443333333	2.61	2.557586062	3.02E-31	5.18E-30	gpr146
ncbi_101791864	1.166666667	5.85	2.326044203	3.38E-31	5.80E-30	lgr5-a
ncbi_101792698	3.033333333	0.103333333	-4.875526425	4.21E-31	7.19E-30	Sytl5
ncbi_106014253	3.126666667	0.12	-4.703519111	5.23E-31	8.89E-30	MYCT1
ncbi_101799672	0.713333333	3.38	2.244374951	1.84E-30	3.08E-29	IL20RA
ncbi_113841866	8.923333333	1.566666667	-2.509884477	2.11E-30	3.53E-29	pol
ncbi_101795348	0.276666667	1.41	2.349474422	2.39E-30	3.98E-29	KCTD16

ncbi_101798978	0.16	1.75	3.451211112	4.93E-30	8.14E-29	TSHR
ncbi_101799162	2.106666667	0.173333333	-3.60334103	6.83E-30	1.12E-28	COLEC11
ncbi_101794194	6.893333333	0.533333333	-3.692092375	7.33E-30	1.20E-28	IL2RG
ncbi_101795738	1.44	6.253333333	2.11855661	1.38E-29	2.24E-28	FAM13B
ncbi_101797344	3.083333333	0.423333333	-2.864624869	1.65E-29	2.68E-28	PARP8
ncbi_101803200	3.856666667	0.513333333	-2.909386608	1.74E-29	2.82E-28	ANKRD29
ncbi_101792194	7.97	0.913333333	-3.125366332	2.20E-29	3.54E-28	PDLIM4
ncbi_101790484	5.986666667	1.346666667	-2.152360152	6.05E-29	9.59E-28	CDC45
ncbi_101789620	0.173333333	1.576666667	3.185256655	6.57E-29	1.04E-27	--
ncbi_101797579	2.263333333	0.033333333	-6.085339669	1.09E-28	1.71E-27	KDR
ncbi_101793568	2.306666667	0.283333333	-3.025237291	1.42E-28	2.23E-27	PTPRZ1
ncbi_101801281	4.246666667	0.566666667	-2.905758626	1.81E-28	2.80E-27	P2RX7
ncbi_101801378	0.866666667	3.91	2.173619485	2.02E-28	3.12E-27	MRC1
ncbi_101793812	10.11	1.5	-2.752748591	2.12E-28	3.28E-27	Il5ra
ncbi_101791353	5.306666667	1.226666667	-2.113062664	2.33E-28	3.59E-27	MCM10
ncbi_113843947	5.08	1.133333333	-2.164256251	2.39E-28	3.68E-27	Kif20b
ncbi_101795255	0.686666667	3.76	2.453050825	7.17E-28	1.09E-26	tmem38b-b
ncbi_101800417	0.773333333	3.34	2.110685798	8.11E-28	1.23E-26	ANKRD6
ncbi_101794612	3.56	0.733333333	-2.279336218	8.97E-28	1.36E-26	CHL1
ncbi_101802330	4.253333333	1.06	-2.004529658	9.62E-28	1.45E-26	CHST11
ncbi_101800113	7.026666667	1.35	-2.379881054	1.01E-27	1.53E-26	Gpa33
ncbi_110351750	5.14	1.06	-2.277704095	3.25E-27	4.85E-26	--
ncbi_101793032	2.156666667	0.28	-2.945304479	3.96E-27	5.91E-26	PCDH1
MSTRG.16539	0.173333333	5.943333333	5.099651269	5.38E-27	7.99E-26	MYH1B
ncbi_101794826	0.026666667	1.363333333	5.675957033	8.11E-27	1.20E-25	ACVR1C
ncbi_101792323	1.943333333	0.173333333	-3.486912355	9.17E-27	1.35E-25	ARHGEF9
ncbi_101793511	2.14	0.31	-2.787270676	1.15E-26	1.69E-25	SAMD9
ncbi_101804919	3.24	0.273333333	-3.567260499	1.16E-26	1.69E-25	HEYL
ncbi_101797713	7.33	1.476666667	-2.311469	1.34E-26	1.96E-25	TNFSF10
ncbi_101791444	4.656666667	0.54	-3.108266303	1.97E-26	2.87E-25	SLA
ncbi_101798969	0.526666667	2.553333333	2.277419834	2.06E-26	3.00E-25	ZFPM2
ncbi_101794139	5.61	1.13	-2.311677998	2.15E-26	3.11E-25	PARPBP

ncbi_101795310	0.22	2.22	3.334984248	3.44E-26	4.95E-25	SLC16A12
ncbi_101796955	1.593333333	0.14	-3.508549385	4.18E-26	6.00E-25	ARAP3
ncbi_110352128	6.81	0.616666667	-3.465092028	5.51E-26	7.87E-25	WISP2
ncbi_101803463	6.27	1.326666667	-2.240659513	2.28E-25	3.19E-24	CDCA8
ncbi_101791715	1.4	5.86	2.065473837	2.77E-25	3.85E-24	VEGFD
ncbi_101800022	8.17	1.61	-2.34327539	9.00E-25	1.23E-23	RGS18
ncbi_101794592	0.143333333	1.51	3.397102485	1.08E-24	1.48E-23	ESRRB
ncbi_101790320	5.043333333	1.16	-2.120252776	1.09E-24	1.48E-23	RFFL
ncbi_101790164	1.846666667	0.183333333	-3.332382453	1.23E-24	1.66E-23	FLT4
ncbi_101791895	2.99	0.3	-3.317111079	1.33E-24	1.81E-23	Nf2
ncbi_101804745	33.81333333	7.13	-2.245618263	1.44E-24	1.95E-23	LAMTOR2
ncbi_101799932	10.46666667	2.04	-2.359161001	1.65E-24	2.23E-23	RGCC
ncbi_113845717	0.26	7.12	4.775293713	2.01E-24	2.70E-23	TFPI2
ncbi_101799307	2.876666667	0.616666667	-2.221835289	6.83E-24	8.99E-23	IQGAP3
ncbi_101803184	2.276666667	25.16	3.466136939	7.17E-24	9.42E-23	FKBP5
ncbi_113840986	0.213333333	2.716666667	3.670656249	1.99E-23	2.57E-22	ASZ1
ncbi_101803781	6.02	1.29	-2.222392421	2.05E-23	2.65E-22	Hjurp
ncbi_101790640	1.943333333	0.236666667	-3.037604954	3.04E-23	3.92E-22	DOCK8
ncbi_101802803	11.47	2.296666667	-2.320252004	3.04E-23	3.92E-22	Map3k7cl
ncbi_101798404	5.94	0.97	-2.614406279	3.10E-23	3.99E-22	CD1D
ncbi_101796737	1.09	5.95	2.448561533	5.67E-23	7.19E-22	CA8
ncbi_101804840	4.256666667	0.98	-2.118870465	8.35E-23	1.05E-21	TP53I11
ncbi_101789662	6.93	1.143333333	-2.599609277	1.09E-22	1.37E-21	Mfsd12
ncbi_110351393	11.86666667	0.943333333	-3.653003283	1.18E-22	1.47E-21	Srgn
ncbi_101795189	1.136666667	0.166666667	-2.769771739	1.93E-22	2.39E-21	CNTNAP2
ncbi_101795684	2.793333333	0.083333333	-5.066950244	2.33E-22	2.88E-21	4-Sep
ncbi_101794895	4.39	0.666666667	-2.71918344	2.41E-22	2.98E-21	Pdgfb
ncbi_101800531	0.676666667	3.983333333	2.557458986	2.81E-22	3.46E-21	nr4a1
ncbi_101793250	5.383333333	1.32	-2.02796183	3.20E-22	3.93E-21	UBQLN1
ncbi_101804573	7.976666667	1.213333333	-2.716810041	3.30E-22	4.05E-21	DCK
ncbi_101794457	3.61	0.163333333	-4.466107683	4.26E-22	5.19E-21	CD1D
ncbi_101802918	2.396666667	0.283333333	-3.080457024	4.38E-22	5.33E-21	PECAM1

ncbi_101800349	3.543333333	0.31	-3.51476707	5.77E-22	7.00E-21	RGS4
ncbi_101804073	0.96	0.126666667	-2.921997488	8.25E-22	9.95E-21	ADCY1
ncbi_101804835	4.02	0.596666667	-2.752198415	9.95E-22	1.20E-20	CORO2B
ncbi_101801144	0.326666667	2.536666667	2.957042799	1.02E-21	1.22E-20	ERBB4
ncbi_101804747	10.65333333	2.616666667	-2.025502849	1.02E-21	1.23E-20	CD47
ncbi_101791887	0.206666667	1.753333333	3.084722679	1.47E-21	1.75E-20	B3GALT5
ncbi_101797012	3.38	0.43	-2.974614682	1.57E-21	1.87E-20	BNC1
ncbi_101798590	2.046666667	9.413333333	2.201429528	3.39E-21	3.98E-20	csgA
ncbi_101795145	1.383333333	0.286666667	-2.270702772	3.44E-21	4.04E-20	DCHS2
MSTRG.12650	70.67333333	15.13333333	-2.223436159	4.07E-21	4.75E-20	--
ncbi_101796718	3.83	0.873333333	-2.132740081	4.64E-21	5.41E-20	Arhgap45
ncbi_101795383	1.676666667	0.05	-5.067523994	5.50E-21	6.38E-20	IL18R1
ncbi_101796599	4.826666667	0.996666667	-2.275844213	7.37E-21	8.51E-20	DHRS12
ncbi_101791466	10.54333333	2.063333333	-2.353282241	8.34E-21	9.60E-20	SBSPON
ncbi_101800967	2.856666667	0.11	-4.698757275	8.69E-21	9.99E-20	Esm1
ncbi_101800011	82.70333333	14.48666667	-2.513219804	8.94E-21	1.03E-19	COL12A1
ncbi_106020629	12.63	2.663333333	-2.245549732	1.23E-20	1.40E-19	GLIPR1
ncbi_101800934	2.253333333	0.51	-2.143491594	1.32E-20	1.50E-19	ARHGEF4
ncbi_101801495	13.54666667	2.013333333	-2.750279947	1.35E-20	1.53E-19	Rgs1
ncbi_110351193	3.256666667	0.396666667	-3.037396989	2.10E-20	2.37E-19	CXorf21
ncbi_101790838	13.33666667	1.93	-2.788725375	2.48E-20	2.78E-19	BTF3
MSTRG.17782	7.59	1.153333333	-2.718290348	3.73E-20	4.15E-19	VLDLR
ncbi_101802913	3.37	0.226666667	-3.894104441	4.86E-20	5.38E-19	GABRD
ncbi_101802342	0.106666667	1.586666667	3.894817763	5.73E-20	6.33E-19	HRH3
ncbi_101793216	7.926666667	0.16	-5.630570499	6.91E-20	7.59E-19	CDH5
ncbi_101802909	19.37666667	0.076666667	-7.981505237	1.24E-19	1.35E-18	CCL4
ncbi_101800806	1.44	0.163333333	-3.140177658	2.03E-19	2.19E-18	VWF
ncbi_113839925	0.636666667	2.723333333	2.09676344	2.55E-19	2.75E-18	KIAA1324
ncbi_101798066	0.976666667	0.076666667	-3.671194898	2.87E-19	3.08E-18	F8
ncbi_101798219	6.123333333	1.413333333	-2.115215457	3.05E-19	3.27E-18	KIF2C
ncbi_101795463	6.993333333	1.503333333	-2.217815339	3.72E-19	3.98E-18	PIMREG
ncbi_101800757	1.623333333	0.04	-5.342815461	3.75E-19	4.01E-18	CCR5

ncbi_101804508	3.423333333	0.293333333	-3.544788848	9.52E-19	1.00E-17	AKR1B10
ncbi_101795655	2.25	0.46	-2.290219235	1.14E-18	1.19E-17	PKP2
ncbi_101801363	0.1	0.86	3.10433666	1.85E-18	1.91E-17	NTSR1
MSTRG.1123	2.92	0.483333333	-2.59487797	1.95E-18	2.01E-17	TTMP
ncbi_101796943	1.516666667	0.366666667	-2.048363022	1.97E-18	2.03E-17	NLRC5
ncbi_101794066	0.39	2	2.358453971	2.72E-18	2.79E-17	IGSF11
ncbi_101805421	0.283333333	2.726666667	3.266566097	3.82E-18	3.88E-17	CTGF
ncbi_113840909	0.686666667	6.47	3.236083876	3.84E-18	3.90E-17	COL14A1
MSTRG.16836	19.57333333	2.936666667	-2.736638044	4.25E-18	4.32E-17	env
MSTRG.15967	0.38	2.27	2.578620974	5.38E-18	5.44E-17	ADAMTS19
MSTRG.1135	7.07	1.553333333	-2.186342761	5.73E-18	5.79E-17	HHLA2
ncbi_101796665	1.596666667	0.016666667	-6.581953751	8.58E-18	8.62E-17	DCSTAMP
ncbi_101795659	0.026666667	1.58	5.888743249	1.17E-17	1.16E-16	Smtnl2
ncbi_101802181	0.02	3.58	7.483815777	1.20E-17	1.19E-16	ANGPTL7
ncbi_101799192	8.92	40.02333333	2.165725711	1.42E-17	1.41E-16	XDH
ncbi_101802032	0.71	2.87	2.015159807	1.89E-17	1.85E-16	ABLIM2
ncbi_101800021	1.48	0.106666667	-3.794415866	1.94E-17	1.89E-16	UNC13D
ncbi_101803352	16.97	3.656666667	-2.21438554	2.28E-17	2.22E-16	CD74
ncbi_101800038	0.043333333	0.433333333	3.321928095	2.77E-17	2.68E-16	CACNA1G
ncbi_101803437	3.703333333	0.796666667	-2.216776293	2.81E-17	2.72E-16	CDCA7
ncbi_101793673	2.943333333	0.156666667	-4.231680776	2.89E-17	2.80E-16	TM4SF1
ncbi_101805216	1.233333333	0.12	-3.361456459	3.02E-17	2.92E-16	ITGA11
ncbi_101801322	0.056666667	0.76	3.745427173	3.13E-17	3.02E-16	--
ncbi_101795668	5.956666667	0.001	-12.54028951	3.51E-17	3.37E-16	MDM4
ncbi_101799544	2.9	0.146666667	-4.305439972	4.18E-17	4.00E-16	snai1
ncbi_113842321	0.023333333	2.083333333	6.480357457	5.34E-17	5.10E-16	ARX
ncbi_101800635	2.103333333	0.123333333	-4.092042829	5.41E-17	5.16E-16	Sema4a
ncbi_101791767	1.296666667	0.313333333	-2.049037493	6.53E-17	6.20E-16	RAB11FIP4
ncbi_101803808	1.613333333	0.026666667	-5.918863237	6.87E-17	6.52E-16	ADGRL4
ncbi_101794085	4.406666667	0.063333333	-6.120578948	7.66E-17	7.22E-16	--
ncbi_101796083	1.073333333	0.053333333	-4.330916878	7.75E-17	7.30E-16	KIT
ncbi_101801059	2.74	0.5	-2.454175893	8.46E-17	7.95E-16	MOV10L1

ncbi_101803834	4.07	0.95	-2.099029376	1.44E-16	1.34E-15	TMEM40
ncbi_101801698	0.84	3.616666667	2.106199404	1.51E-16	1.41E-15	FOS
ncbi_101790981	5.62	1.333333333	-2.075532631	1.76E-16	1.63E-15	Slbp
ncbi_101803492	0.89	0.103333333	-3.106499621	1.76E-16	1.63E-15	Fat4
ncbi_101791519	1.92	0.36	-2.415037499	2.31E-16	2.12E-15	MAP3K15
ncbi_101800331	1.953333333	0.206666667	-3.240560544	2.41E-16	2.21E-15	SPTY2D1
ncbi_113839921	5.256666667	0.923333333	-2.509224779	2.83E-16	2.58E-15	--
ncbi_101798416	9.723333333	2.196666667	-2.146135016	4.26E-16	3.86E-15	ANGPT2
ncbi_101802230	1.92	0.056666667	-5.08246216	4.46E-16	4.04E-15	sox18-a
ncbi_101798515	2.316666667	0.483333333	-2.260960078	6.43E-16	5.78E-15	KIF18A
ncbi_101790967	3.773333333	0.483333333	-2.964749153	9.45E-16	8.42E-15	KAZALD1
ncbi_101792044	1.046666667	0.25	-2.065802058	1.28E-15	1.13E-14	SAMD12
ncbi_101794097	5.94	0.001	-12.53624722	1.82E-15	1.60E-14	Tex261
ncbi_101797804	1.586666667	0.166666667	-3.250961574	1.88E-15	1.65E-14	PRDM1
ncbi_101802715	4.993333333	0.203333333	-4.618084571	2.13E-15	1.86E-14	IL18
ncbi_101792080	1.59	0.17	-3.225420114	3.02E-15	2.61E-14	KCNK13
ncbi_101799388	3.486666667	0.87	-2.00276114	3.05E-15	2.65E-14	TLR4
ncbi_101794041	1.053333333	0.02	-5.718818247	3.43E-15	2.97E-14	Tie1
ncbi_101803726	1.59	0.25	-2.669026766	3.48E-15	3.01E-14	CNK3/IPCEF1
ncbi_101800865	0.816666667	4.453333333	2.447066353	3.98E-15	3.41E-14	WNT2B
ncbi_113842385	2.663333333	0.393333333	-2.759408644	5.45E-15	4.62E-14	--
ncbi_101796896	2.5	0.001	-11.28771238	6.31E-15	5.35E-14	Slco4c1
ncbi_101803831	0.183333333	0.843333333	2.201633861	7.59E-15	6.38E-14	TMEM132B
ncbi_101795460	0.056666667	0.68	3.584962501	8.04E-15	6.75E-14	DPP10
ncbi_101789475	1.206666667	0.176666667	-2.771925433	8.90E-15	7.46E-14	DACH1
ncbi_101799695	2.33	0.383333333	-2.603658594	9.27E-15	7.75E-14	Rgs14
ncbi_101802124	0.016666667	0.55	5.044394119	9.89E-15	8.24E-14	GPR12
ncbi_101799683	3.32	0.383333333	-3.114511881	9.94E-15	8.28E-14	cfaD
ncbi_101800569	3.25	0.74	-2.134842542	1.61E-14	1.32E-13	SGK3
ncbi_101799041	3.116666667	0.753333333	-2.048643592	1.71E-14	1.40E-13	PRKCH
ncbi_101792678	1.753333333	0.09	-4.284031487	2.01E-14	1.63E-13	ANGPT4
ncbi_101790909	0.19	1.32	2.796466606	2.01E-14	1.63E-13	Myh8

ncbi_101802211	0.196666667	2.553333333	3.698557533	2.04E-14	1.66E-13	Timd4
MSTRG.14722	4.39	0.001	-12.10000522	2.12E-14	1.72E-13	--
ncbi_101800961	1.243333333	0.12	-3.373106819	2.14E-14	1.74E-13	PHACTR1
ncbi_101791125	2.076666667	0.186666667	-3.475733431	2.35E-14	1.90E-13	--
ncbi_101798215	2.016666667	0.5	-2.011972642	4.22E-14	3.37E-13	STIL
ncbi_101802627	2.786666667	0.253333333	-3.459431619	4.79E-14	3.81E-13	EVI2A
ncbi_106016818	1.226666667	0.02	-5.938599455	6.43E-14	5.09E-13	SMIM32
ncbi_101803164	2.17	0.286666667	-2.920248978	6.75E-14	5.34E-13	RET
ncbi_101799384	1.51	0.206666667	-2.86917093	7.22E-14	5.70E-13	ATP8A2
ncbi_101792727	1.576666667	0.206666667	-2.931500063	7.45E-14	5.87E-13	Mcf2l
ncbi_101802428	12.11	3.003333333	-2.011562355	1.28E-13	9.94E-13	CLEC19A
ncbi_101802668	1.466666667	0.05	-4.874469118	1.64E-13	1.27E-12	BANK1
ncbi_101803904	1.34	0.193333333	-2.793070696	1.70E-13	1.31E-12	FBLN7
ncbi_101797637	1.596666667	0.2	-2.99699125	2.01E-13	1.54E-12	Mapk8ip1
ncbi_101794676	0.546666667	0.05	-3.450661409	2.14E-13	1.64E-12	GPRIN3
ncbi_101797832	3.016666667	0.69	-2.128287024	2.17E-13	1.66E-12	TLR5
ncbi_101802020	1.47	0.053333333	-4.784634846	2.28E-13	1.74E-12	SLC2A6
ncbi_101799158	0.07	0.846666667	3.596367264	2.47E-13	1.89E-12	--
ncbi_101803931	0.043333333	0.826666667	4.253756592	2.67E-13	2.04E-12	MYLK2
ncbi_101796112	4.07	0.123333333	-5.044394119	2.78E-13	2.11E-12	TM4SF18
ncbi_101802995	0.856666667	0.1	-3.098733954	3.80E-13	2.88E-12	CDH12
ncbi_101804552	3.296666667	0.57	-2.531974196	4.90E-13	3.68E-12	PLAU
ncbi_101794396	1.673333333	0.02	-6.386581053	5.79E-13	4.32E-12	PLA2G4E
MSTRG.17164	8.41	1.63	-2.367233836	6.12E-13	4.56E-12	LRP8
ncbi_101799400	1.473333333	0.286666667	-2.361637805	6.38E-13	4.74E-12	HTR2A
ncbi_101804018	1.92	0.446666667	-2.103835811	6.74E-13	5.00E-12	1-Mar
ncbi_101803806	1.15	0.07	-4.038135129	6.94E-13	5.14E-12	USP18
ncbi_101793858	6.63	1.443333333	-2.199604346	7.29E-13	5.39E-12	Ankrd37
ncbi_101801376	1.526666667	0.023333333	-6.031848866	7.33E-13	5.41E-12	Otop3
ncbi_101793632	3.916666667	0.63	-2.636202617	7.71E-13	5.68E-12	KIF23
ncbi_101797917	0.02	0.703333333	5.136136688	8.15E-13	5.99E-12	UNC5C
ncbi_101789408	1.41	0.286666667	-2.298249098	8.59E-13	6.30E-12	--

ncbi_106018497	0.18	0.983333333	2.449683642	1.04E-12	7.63E-12	--
ncbi_101804095	2.696666667	0.03	-6.490070891	1.78E-12	1.28E-11	Serpina3m
ncbi_101797498	2.876666667	0.606666667	-2.245422109	2.06E-12	1.48E-11	RAD54L
ncbi_101804405	0.033333333	0.726666667	4.44625623	2.29E-12	1.64E-11	OPRM1
ncbi_101800943	0.013333333	3.113333333	7.86727874	2.60E-12	1.85E-11	Uts2r
ncbi_113843594	0.14	1.373333333	3.294183104	2.87E-12	2.04E-11	PRDM8
ncbi_113844025	0.263333333	1.793333333	2.767681614	2.99E-12	2.12E-11	Ina
ncbi_101801364	0.823333333	0.001	-9.685332826	3.20E-12	2.27E-11	Ovos
ncbi_101795752	0.466666667	0.04	-3.544320516	3.91E-12	2.76E-11	Greb1
ncbi_101799797	0.473333333	0.083333333	-2.50589093	3.98E-12	2.81E-11	Fut9
ncbi_101801610	2.79	0.54	-2.36923381	4.47E-12	3.14E-11	fam162b
ncbi_101799353	5.346666667	1.03	-2.375995399	4.76E-12	3.34E-11	MGAT4C
ncbi_101790322	0.38	0.066666667	-2.510961919	5.29E-12	3.70E-11	CR1
ncbi_101790768	1.5	6.01	2.00240249	5.43E-12	3.80E-11	SYPL2
ncbi_101796929	0.003333333	0.98	8.199672345	6.75E-12	4.71E-11	EDIL3
ncbi_113844293	3.346666667	0.656666667	-2.349491734	6.81E-12	4.75E-11	Lurap1
ncbi_101803604	0.983333333	0.06	-4.034646143	6.95E-12	4.84E-11	RGS5
ncbi_101797384	0.733333333	0.023333333	-4.974004791	7.36E-12	5.12E-11	Traf5
ncbi_101798429	1.976666667	0.43	-2.200661039	9.06E-12	6.28E-11	EPHA2
ncbi_101789958	3.026666667	0.656666667	-2.204496668	9.57E-12	6.61E-11	NEK2
ncbi_101802306	3.906666667	0.723333333	-2.433205622	9.80E-12	6.76E-11	EVI2B
ncbi_101795331	0.696666667	0.043333333	-4.006919414	9.83E-12	6.78E-11	FLT1
ncbi_101796964	4.033333333	0.446666667	-3.174702142	1.07E-11	7.39E-11	ABI3BP
ncbi_101802220	2.86	0.656666667	-2.122782018	1.09E-11	7.47E-11	CENPE
ncbi_101804825	6.78	1.516666667	-2.160381229	1.34E-11	9.16E-11	CCNB3
ncbi_101795208	2.426666667	0.006666667	-8.50779464	1.42E-11	9.70E-11	STEAP4
ncbi_101794184	7.24	1.223333333	-2.565172135	1.59E-11	1.08E-10	HIST2H2AC
ncbi_101804985	1.26	0.016666667	-6.240314329	1.77E-11	1.20E-10	GPR82
ncbi_101804544	1.42	0.256666667	-2.46792308	1.78E-11	1.21E-10	--
ncbi_101792566	2.336666667	0.58	-2.010327138	1.84E-11	1.24E-10	Hip1r
ncbi_101804317	6.453333333	1.14	-2.501010722	1.94E-11	1.31E-10	CSF1
ncbi_101801803	1.4	0.001	-10.45121111	1.98E-11	1.33E-10	OIT3

ncbi_101791385	8.88	1.026666667	-3.112591826	2.12E-11	1.42E-10	CCL5
ncbi_101803106	1.563333333	0.001	-10.61040971	2.28E-11	1.53E-10	Rbfox2
ncbi_101797444	1.976666667	0.273333333	-2.85433629	2.36E-11	1.58E-10	Abhd6
ncbi_113843962	2.233333333	0.001	-11.12498288	2.77E-11	1.84E-10	VWA2
ncbi_101797163	2.85	0.056666667	-5.652317769	2.87E-11	1.90E-10	ALOX5AP
ncbi_101799500	0.05	0.57	3.510961919	3.08E-11	2.03E-10	Klhl35
ncbi_101797775	2.326666667	0.376666667	-2.626904264	3.71E-11	2.44E-10	CENPN
ncbi_113844808	1.97	0.333333333	-2.56315813	4.04E-11	2.65E-10	V-FPS
ncbi_101801621	1.543333333	0.34	-2.182443041	4.28E-11	2.81E-10	LRRK1
ncbi_101802660	1.433333333	0.116666667	-3.618909833	4.43E-11	2.90E-10	Adgrg2
ncbi_101792331	0.206666667	0.866666667	2.068171503	4.49E-11	2.94E-10	COL8A2
ncbi_101791637	0.106666667	0.973333333	3.189824559	5.29E-11	3.45E-10	GRM7
ncbi_113839804	2.336666667	0.163333333	-3.83856079	5.31E-11	3.47E-10	Tinagl1
ncbi_101799397	0.22	1.943333333	3.142957954	7.28E-11	4.69E-10	SRD5A2
ncbi_101797649	5.176666667	0.823333333	-2.652474883	8.31E-11	5.32E-10	SPC25
ncbi_101794483	1.38	0.001	-10.43045255	8.92E-11	5.69E-10	--
ncbi_101800435	3.726666667	0.736666667	-2.338801913	1.03E-10	6.57E-10	C1qtnf4
ncbi_101790749	2.2	0.23	-3.257797757	1.05E-10	6.65E-10	Cnrip1
ncbi_101790524	1.21	0.063333333	-4.255898225	1.18E-10	7.43E-10	Mat2a
ncbi_113842153	2.406666667	0.001	-11.23282062	1.18E-10	7.45E-10	PLAU
ncbi_110354688	2.476666667	0.346666667	-2.836778682	1.23E-10	7.77E-10	Rspo4
ncbi_101799102	1.816666667	0.083333333	-4.44625623	1.38E-10	8.69E-10	ST6GALNAC2
ncbi_101794229	0.866666667	0.09	-3.267480311	1.48E-10	9.27E-10	RASGRP1
ncbi_101791432	1.55	0.066666667	-4.539158811	1.76E-10	1.10E-09	Krt23
ncbi_106015064	0.053333333	0.55	3.366322214	1.98E-10	1.23E-09	MRC1
ncbi_101793514	1.023333333	0.08	-3.677132345	2.17E-10	1.34E-09	CCDC68
ncbi_101799817	0.103333333	0.683333333	2.725283789	2.27E-10	1.40E-09	CNTN3
ncbi_101789855	0.18	1.24	2.784271309	2.44E-10	1.50E-09	Gpd1
ncbi_101798538	0.596666667	0.033333333	-4.161887682	2.63E-10	1.61E-09	AQP4
ncbi_101792845	0.506666667	0.05	-3.341036918	2.71E-10	1.66E-09	SPAG17
ncbi_101800454	3.433333333	23.98	2.804149822	2.83E-10	1.73E-09	DEPTOR
ncbi_113840727	3.563333333	0.86	-2.050818882	2.89E-10	1.76E-09	SKA3

ncbi_101791398	0.02	0.276666667	3.790076931	3.02E-10	1.84E-09	ASB11
ncbi_101791456	0.803333333	0.001	-9.64985493	3.04E-10	1.85E-09	CDCP1
ncbi_101792742	6.073333333	1.143333333	-2.409242478	3.45E-10	2.10E-09	RAMP2
ncbi_101802365	4.406666667	0.813333333	-2.437769124	3.62E-10	2.19E-09	NCF1
ncbi_101799219	1.34	0.216666667	-2.628683878	3.78E-10	2.29E-09	SLC22A4
ncbi_101791320	1.86	0.393333333	-2.241478262	3.89E-10	2.35E-09	Kif20b
ncbi_101792227	1.063333333	4.3	2.015742737	4.02E-10	2.43E-09	CAV3
ncbi_101789957	0.001	0.526666667	9.040746342	4.13E-10	2.49E-09	Pcdh19
ncbi_101797234	1.733333333	0.336666667	-2.36415633	4.94E-10	2.95E-09	KIF18B
ncbi_113843792	1.84	8.096666667	2.137622318	5.09E-10	3.04E-09	Cdkn1c
ncbi_113842446	1.613333333	0.25	-2.690044547	5.21E-10	3.10E-09	--
ncbi_101791376	2.153333333	0.016666667	-7.01346226	5.32E-10	3.17E-09	Upp1
ncbi_101795858	0.943333333	0.2	-2.237767647	5.58E-10	3.32E-09	NTN4
ncbi_101801979	6.693333333	0.89	-2.910847622	6.13E-10	3.64E-09	PREX2
ncbi_101804934	0.96	0.186666667	-2.362570079	6.53E-10	3.87E-09	Gabrb2
ncbi_101795149	1.9	0.436666667	-2.121395108	6.81E-10	4.03E-09	Hectd3
ncbi_101795631	0.001	0.926666667	9.855906667	7.03E-10	4.15E-09	RASA4
ncbi_101800737	7.493333333	0.16	-5.549463819	7.69E-10	4.52E-09	NCF2
ncbi_101790752	1.876666667	0.253333333	-2.889063599	8.04E-10	4.72E-09	RND2
ncbi_101797939	0.01	1.056666667	6.723376529	8.13E-10	4.77E-09	Pde4d
ncbi_101800588	1.353333333	0.006666667	-7.665335917	8.81E-10	5.16E-09	Gpr132
ncbi_101799470	0.001	0.973333333	9.926790153	9.19E-10	5.37E-09	SNTG2
ncbi_101800190	0.106666667	0.536666667	2.330916878	9.21E-10	5.38E-09	Grid1
ncbi_101793391	0.923333333	0.126666667	-2.865814653	1.02E-09	5.93E-09	Shank3
ncbi_101796803	21.97333333	1.88	-3.546949175	1.07E-09	6.26E-09	ITGB2
ncbi_113845381	5	1.193333333	-2.066931008	1.15E-09	6.68E-09	Clec2e
ncbi_101790896	0.333333333	4.556666667	3.772941338	1.28E-09	7.38E-09	AFAP1L1
ncbi_101800184	2.183333333	0.296666667	-2.879617665	1.40E-09	8.02E-09	MYO1G
ncbi_101798015	1.693333333	0.193333333	-3.130703692	1.40E-09	8.04E-09	CBS
ncbi_101804397	1.89	0.066666667	-4.82527683	1.45E-09	8.34E-09	LTC4S
ncbi_113839971	1.263333333	0.123333333	-3.356600673	1.50E-09	8.61E-09	ARHGAP9
ncbi_101789711	0.176666667	1.01	2.515253529	1.60E-09	9.16E-09	Kcnc1

ncbi_101793081	2.703333333	0.666666667	-2.019701914	1.99E-09	1.13E-08	MBOAT1
ncbi_101796013	0.046666667	0.766666667	4.038135129	2.54E-09	1.44E-08	NXPH2
ncbi_101797173	0.216666667	0.026666667	-3.022367813	2.74E-09	1.54E-08	DNAH5
ncbi_101794727	0.573333333	0.126666667	-2.178337241	3.12E-09	1.74E-08	CUBN
ncbi_113843973	0.666666667	0.15	-2.152003093	3.19E-09	1.78E-08	MKI67
ncbi_101796754	3.02	0.456666667	-2.725335157	3.42E-09	1.91E-08	ARG2
ncbi_113842175	0.001	0.886666667	9.79224803	3.49E-09	1.95E-08	Sntg2
ncbi_101794616	0.613333333	0.03	-4.353636955	3.59E-09	2.00E-08	Sall3
ncbi_101790989	2.82	0.546666667	-2.366961849	3.85E-09	2.14E-08	Ctxn1
ncbi_101794597	3.21	0.496666667	-2.692223467	3.97E-09	2.20E-08	NCF4
ncbi_101804105	1.906666667	0.186666667	-3.352516415	4.10E-09	2.27E-08	F9
ncbi_101799871	0.001	0.863333333	9.753773882	4.81E-09	2.64E-08	NOV
ncbi_101789777	1.973333333	0.326666667	-2.594743522	4.82E-09	2.65E-08	CARD9
ncbi_101795505	2.04	0.376666667	-2.43720888	5.05E-09	2.77E-08	Kcnj15
ncbi_101793931	0.01	0.246666667	4.624490865	5.05E-09	2.77E-08	DCC
ncbi_101791863	2.183333333	0.38	-2.522461082	6.48E-09	3.52E-08	Cndp1
ncbi_106020454	2.66	0.27	-3.300394933	7.32E-09	3.96E-08	K-CAM
ncbi_113841024	3.866666667	0.706666667	-2.451988635	8.12E-09	4.37E-08	--
ncbi_101792490	0.49	0.03	-4.029747343	1.19E-08	6.29E-08	TRPC6
ncbi_101805376	0.466666667	0.001	-8.866248611	1.19E-08	6.31E-08	RIPOR1
ncbi_101799183	0.043333333	0.753333333	4.119739244	1.50E-08	7.93E-08	KIRREL3
ncbi_101795675	0.06	0.836666667	3.801618553	1.56E-08	8.24E-08	SCG2
ncbi_101796783	0.633333333	0.106666667	-2.569855608	1.60E-08	8.42E-08	SH3TC2
ncbi_101789391	0.22	0.026666667	-3.044394119	1.71E-08	8.99E-08	HMCN2
ncbi_113841161	0.001	1.973333333	10.94641896	1.76E-08	9.22E-08	CYC
ncbi_101793344	1.743333333	0.386666667	-2.172686141	1.87E-08	9.78E-08	etnppl
ncbi_101805449	2.106666667	0.236666667	-3.154033629	1.89E-08	9.84E-08	CYSLTR2
ncbi_101796090	2.336666667	0.45	-2.376455037	2.18E-08	1.13E-07	ALDOB
ncbi_101797743	3.466666667	0.743333333	-2.221467913	2.42E-08	1.25E-07	KIF2A
ncbi_101793227	0.323333333	0.033333333	-3.277984747	2.79E-08	1.43E-07	PTPRB
ncbi_101803018	0.103333333	1.346666667	3.704015172	3.39E-08	1.72E-07	METTL6
ncbi_113839837	0.096666667	0.803333333	3.054908341	3.42E-08	1.74E-07	MFRP

ncbi_101793979	0.49	0.083333333	-2.555816155	3.65E-08	1.85E-07	Apc2
ncbi_101798726	1.283333333	0.143333333	-3.162449881	3.78E-08	1.91E-07	APOLD1
ncbi_101791342	4.01	0.913333333	-2.134388844	3.92E-08	1.98E-07	DUSP5
ncbi_101793630	0.956666667	0.076666667	-3.641344971	4.44E-08	2.23E-07	RHOH
ncbi_101803313	1.51	0.28	-2.431049817	4.84E-08	2.42E-07	SASH3
ncbi_113840905	1.923333333	0.286666667	-2.746162754	4.84E-08	2.42E-07	WDYHV1
ncbi_113839755	0.713333333	0.001	-9.478432581	4.88E-08	2.44E-07	SORBS3
ncbi_101794111	1.633333333	0.23	-2.828113482	5.12E-08	2.56E-07	CSF2RA
ncbi_113845183	1.58	0.193333333	-3.030762254	5.21E-08	2.60E-07	--
ncbi_101789505	0.096666667	0.85	3.136372442	5.41E-08	2.69E-07	NAALAD2
ncbi_101789875	0.376666667	0.06	-2.650253961	5.71E-08	2.84E-07	ALK
ncbi_101798767	0.723333333	0.023333333	-4.95419631	6.23E-08	3.08E-07	SELP
ncbi_101799296	0.043333333	0.21	2.276840205	6.73E-08	3.31E-07	DNAH10
ncbi_101790262	0.88	0.17	-2.371968777	6.75E-08	3.32E-07	EDNRB
ncbi_101797588	0.353333333	1.62	2.196892049	6.83E-08	3.36E-07	PLA2G6
ncbi_101790022	0.79	0.12	-2.718818247	6.99E-08	3.43E-07	Mfng
ncbi_113845763	2.976666667	0.526666667	-2.498735617	7.17E-08	3.52E-07	--
ncbi_101790274	1.52	0.193333333	-2.974909019	8.06E-08	3.94E-07	Tmem51
MSTRG.17522	1.93	0.273333333	-2.819867533	8.19E-08	4.00E-07	--
ncbi_101789382	1.576666667	0.093333333	-4.078341451	8.79E-08	4.28E-07	SCEL
ncbi_101795121	2.943333333	0.313333333	-3.231680776	8.82E-08	4.29E-07	HIST2H2AC
ncbi_101801460	1.46	0.03	-5.604862058	9.34E-08	4.54E-07	Esam
ncbi_101801834	1.59	0.313333333	-2.343256604	9.76E-08	4.73E-07	Acsl5
ncbi_101795667	1.15	0.183333333	-2.649092838	1.20E-07	5.77E-07	COL9A2
ncbi_101793567	2.66	0.58	-2.19730144	1.31E-07	6.25E-07	Nasp
ncbi_113839922	1.42	0.126666667	-3.486782107	1.42E-07	6.74E-07	CD300E
ncbi_101804865	1.143333333	0.01	-6.837102265	1.55E-07	7.34E-07	SPRY3
ncbi_101795211	1.71	0.293333333	-2.543383397	1.55E-07	7.36E-07	NMES1
ncbi_101794846	3.87	0.193333333	-4.323171262	1.63E-07	7.69E-07	ARHGAP15
ncbi_101797166	0.473333333	0.033333333	-3.827819025	1.73E-07	8.16E-07	PRAG1
ncbi_101790547	0.17	0.993333333	2.546743178	1.84E-07	8.67E-07	ELOVL4
ncbi_101801992	1.443333333	0.27	-2.418373212	2.04E-07	9.57E-07	SERPINB12

ncbi_101796643	2.546666667	0.103333333	-4.623232518	2.20E-07	1.02E-06	CLEC2D
ncbi_101790680	1.553333333	0.253333333	-2.616258631	2.42E-07	1.12E-06	TMEM268
ncbi_101804057	0.01	0.096666667	3.273018494	2.47E-07	1.14E-06	FCGBP
ncbi_101804875	1.293333333	0.1	-3.693022247	2.59E-07	1.20E-06	Trim39
ncbi_101793109	1.516666667	0.15	-3.337869639	2.85E-07	1.31E-06	EGFL7
ncbi_113844859	0.103333333	3.79	5.196820229	3.16E-07	1.45E-06	--
ncbi_101798784	0.816666667	0.14	-2.544320516	3.40E-07	1.55E-06	--
ncbi_101799365	2.53	0.606666667	-2.060161435	3.70E-07	1.68E-06	REM1
ncbi_101791855	0.716666667	0.04	-4.163230349	3.75E-07	1.70E-06	--
ncbi_106019563	2.293333333	0.35	-2.712019237	3.79E-07	1.72E-06	HAPLN3
ncbi_101802569	0.48	0.01	-5.584962501	3.81E-07	1.73E-06	PCDH12
ncbi_101805380	0.326666667	0.073333333	-2.155278225	3.81E-07	1.73E-06	OTOG
ncbi_113842399	0.31	0.013333333	-4.539158811	4.16E-07	1.88E-06	GAP43
ncbi_101803986	0.043333333	1.193333333	4.783376059	4.80E-07	2.15E-06	CFAP97D1
ncbi_101796113	0.506666667	0.001	-8.984893108	5.05E-07	2.26E-06	DEK
ncbi_101797008	0.99	0.016666667	-5.892391026	5.32E-07	2.38E-06	Gja4
ncbi_101803162	2.33	0.303333333	-2.941354005	5.34E-07	2.39E-06	TMEM196
ncbi_101794378	0.123333333	0.773333333	2.648527629	5.38E-07	2.40E-06	NPY2R
ncbi_101793607	1.376666667	0.086666667	-3.989558253	6.07E-07	2.71E-06	Dele
ncbi_101794893	0.87	0.016666667	-5.705977902	6.19E-07	2.76E-06	P2RY2
ncbi_101802759	0.433333333	0.016666667	-4.700439718	6.24E-07	2.78E-06	SLC6A4
ncbi_101795448	0.596666667	0.09	-2.728928275	6.56E-07	2.91E-06	INHBA
ncbi_106014712	1.273333333	0.3	-2.085575732	6.64E-07	2.94E-06	COL2A1
ncbi_101790773	1.94	0.363333333	-2.416691018	6.76E-07	2.99E-06	DUSP4
ncbi_106019854	0.316666667	0.001	-8.306821202	6.83E-07	3.02E-06	DOK2
ncbi_101803503	0.9	0.166666667	-2.432959407	6.83E-07	3.02E-06	DLL1
ncbi_101792285	1.843333333	0.433333333	-2.088767857	6.84E-07	3.02E-06	PTK2B
ncbi_101795658	2.443333333	0.573333333	-2.091404633	7.25E-07	3.19E-06	HAUS1
ncbi_101798100	0.9	0.063333333	-3.828888084	7.70E-07	3.38E-06	CD1D
ncbi_101797569	0.88	0.06	-3.874469118	8.11E-07	3.54E-06	IFIT5
ncbi_101798732	0.81	0.086666667	-3.224372785	8.29E-07	3.61E-06	BLNK
ncbi_101795890	0.433333333	0.02	-4.437405312	1.02E-06	4.41E-06	KLHL4

ncbi_101803870	1.68	0.173333333	-3.276840205	1.06E-06	4.60E-06	--
ncbi_101803579	1.893333333	0.37	-2.355331253	1.19E-06	5.12E-06	CENPO
ncbi_101800554	0.04	0.276666667	2.790076931	1.22E-06	5.23E-06	FGF16
ncbi_101804010	1.96	0.213333333	-3.199672345	1.29E-06	5.49E-06	CXCL8
ncbi_101796880	1.706666667	0.293333333	-2.540568381	1.34E-06	5.73E-06	PLCD3
ncbi_113844815	0.03	2.8	6.544320516	1.48E-06	6.31E-06	Pde2a
ncbi_101800153	1.313333333	0.32	-2.037089319	1.50E-06	6.38E-06	IKBKE
ncbi_101797770	0.636666667	5.51	3.113442181	1.54E-06	6.53E-06	ANOS1
ncbi_101798346	1.6	0.346666667	-2.206450877	1.63E-06	6.87E-06	Creb3l3
ncbi_101790424	11.93	2.67	-2.159682396	1.66E-06	6.95E-06	tspan36
ncbi_101793135	0.393333333	0.013333333	-4.882643049	1.97E-06	8.19E-06	--
ncbi_101802412	0.796666667	0.126666667	-2.652939295	1.99E-06	8.28E-06	Pigr
ncbi_113842861	0.146666667	0.001	-7.196397213	2.04E-06	8.49E-06	SOX17
ncbi_101794186	0.666666667	0.001	-9.380821784	2.48E-06	1.02E-05	ASS1
ncbi_101790665	0.233333333	0.02	-3.544320516	2.55E-06	1.05E-05	RASSF9
ncbi_113845633	0.16	1.22	2.930737338	2.68E-06	1.10E-05	ADAMTSL5
ncbi_101804270	0.483333333	0.001	-8.916874684	2.77E-06	1.14E-05	FAAH2
ncbi_101802676	0.99	0.23	-2.105794664	2.84E-06	1.16E-05	Slc25a47
ncbi_101789759	0.126666667	0.51	2.009460329	2.87E-06	1.18E-05	Myh7b
ncbi_106019463	0.21	0.033333333	-2.655351829	2.96E-06	1.21E-05	--
ncbi_101798906	1.593333333	0.12	-3.730941807	3.25E-06	1.32E-05	Cd274
ncbi_101799313	0.006666667	0.3	5.491853096	4.03E-06	1.62E-05	NDST4
ncbi_101793362	0.966666667	0.076666667	-3.656347134	4.04E-06	1.62E-05	TRIM14
ncbi_101796057	0.236666667	1.216666667	2.362005534	4.23E-06	1.70E-05	ABRA
ncbi_101794966	0.001	0.256666667	8.003752135	4.43E-06	1.77E-05	MDGA1
ncbi_101793998	0.633333333	0.03	-4.399930607	4.45E-06	1.78E-05	SLC22A3
ncbi_101805460	1.316666667	0.206666667	-2.671512533	4.87E-06	1.94E-05	Frmd4a
ncbi_101794585	1.72	0.333333333	-2.367371066	5.06E-06	2.00E-05	NINJ2
ncbi_101804947	0.393333333	0.056666667	-2.795180208	5.30E-06	2.09E-05	Rab38
ncbi_101796604	0.843333333	0.113333333	-2.895530733	5.59E-06	2.20E-05	EPN3
ncbi_101801239	0.293333333	0.01	-4.874469118	5.90E-06	2.31E-05	B4GALT6
ncbi_101790338	0.703333333	0.033333333	-4.399171094	6.36E-06	2.49E-05	CMPK2

ncbi_101800662	0.92	0.17	-2.436099115	6.68E-06	2.61E-05	SOX13
ncbi_101798027	1.52	0.326666667	-2.21818017	7.06E-06	2.75E-05	NCBP3
ncbi_113843416	0.126666667	1.353333333	3.417408404	7.45E-06	2.90E-05	hmx3b
ncbi_101796085	0.69	0.03	-4.523561956	7.56E-06	2.94E-05	TNIP3
ncbi_101794408	0.001	0.373333333	8.544320516	7.58E-06	2.94E-05	BPIFB4
ncbi_101792322	1.516666667	0.346666667	-2.129283017	7.70E-06	2.99E-05	SAMD13
ncbi_101794441	0.913333333	0.106666667	-3.098032083	8.36E-06	3.23E-05	Themis2
ncbi_101790940	0.85	0.143333333	-2.568088682	8.48E-06	3.27E-05	Spns3
ncbi_101800384	1.1	0.273333333	-2.00877021	8.88E-06	3.42E-05	RFT1
ncbi_101791486	0.48	0.06	-3	9.54E-06	3.66E-05	ABCG4
ncbi_101795243	0.21	0.003333333	-5.977279923	9.78E-06	3.75E-05	RHCG
ncbi_101789673	0.563333333	0.02	-4.815916936	9.95E-06	3.81E-05	TRAF3IP3
ncbi_113840127	0.653333333	0.09	-2.859822342	1.00E-05	3.84E-05	--
ncbi_101801763	0.456666667	0.106666667	-2.098032083	1.07E-05	4.07E-05	LEKR1
ncbi_101805361	0.613333333	0.001	-9.26052755	1.09E-05	4.14E-05	PLVAP
ncbi_101793811	1.86	0.33	-2.494764692	1.10E-05	4.19E-05	PTHLH
ncbi_101793624	0.023333333	0.343333333	3.879145605	1.12E-05	4.24E-05	Asb10
ncbi_101798159	0.286666667	0.02	-3.841302254	1.29E-05	4.84E-05	DLEC1
ncbi_101797264	0.226666667	0.003333333	-6.087462841	1.32E-05	4.95E-05	TLR7
ncbi_101801276	0.376666667	0.006666667	-5.820178962	1.32E-05	4.98E-05	SLC17A8
ncbi_113840568	0.403333333	2.736666667	2.762375175	1.35E-05	5.07E-05	WDYHV1
ncbi_113841255	0.403333333	2.736666667	2.762375175	1.35E-05	5.07E-05	WDYHV1
ncbi_101796858	1.86	0.043333333	-5.423681594	1.36E-05	5.10E-05	Bcl2l15
ncbi_101793226	1.006666667	0.213333333	-2.238404739	1.57E-05	5.85E-05	CD83
ncbi_101790845	3.06	0.74	-2.047934477	1.66E-05	6.18E-05	D10Jhu81e
ncbi_101789452	1.756666667	0.32	-2.456696651	1.71E-05	6.34E-05	PENK
ncbi_101790968	0.073333333	0.44	2.584962501	1.94E-05	7.15E-05	FAM19A2
ncbi_101789823	0.25	1.77	2.82374936	2.03E-05	7.44E-05	FKBP1B
ncbi_101797507	0.63	0.15	-2.070389328	2.07E-05	7.59E-05	SYT4
ncbi_101801046	0.023333333	0.273333333	3.550197083	2.10E-05	7.67E-05	DSC2
ncbi_101800028	1.546666667	0.2	-2.9510904	2.11E-05	7.72E-05	ARHGEF5
ncbi_101797665	0.94	0.103333333	-3.185355042	2.21E-05	8.04E-05	ACKR2

ncbi_113843933	0.093333333	0.636666667	2.770073906	2.24E-05	8.16E-05	GRID1
ncbi_101803273	0.343333333	0.053333333	-2.686500527	2.25E-05	8.18E-05	OR5AS1
ncbi_101802747	0.236666667	0.013333333	-4.14974712	2.40E-05	8.71E-05	ASAP1
ncbi_101800424	2.3	0.056666667	-5.34298971	2.55E-05	9.22E-05	--
ncbi_101800605	0.943333333	0.096666667	-3.286677248	2.87E-05	0.000102994	--
ncbi_101795665	1.116666667	0.226666667	-2.300554444	2.94E-05	0.000105359	ASPA
ncbi_101801843	0.623333333	0.001	-9.283860054	2.97E-05	0.000106312	Lipm
ncbi_113845533	0.016666667	0.266666667	4	3.27E-05	0.00011675	HS3ST3A1
ncbi_101792805	0.66	0.096666667	-2.771375625	3.45E-05	0.000122697	NGF
ncbi_106020611	0.053333333	0.6	3.491853096	3.84E-05	0.0001354	BTN2A1
ncbi_101792469	0.45	0.04	-3.491853096	3.99E-05	0.000140463	Elavl4
ncbi_101803545	0.603333333	0.001	-9.236811481	4.05E-05	0.000142123	CXorf36
ncbi_101799140	0.053333333	0.5	3.22881869	4.05E-05	0.000142274	Pkd2l2
ncbi_101794754	0.39	0.016666667	-4.548436625	4.31E-05	0.000150362	MYO1A
ncbi_101791551	1.466666667	0.076666667	-4.257797757	4.38E-05	0.00015279	TSPAN8
ncbi_101794511	1.27	0.293333333	-2.114215569	4.54E-05	0.000158166	Ttc25
ncbi_106017781	0.68	0.126666667	-2.424497829	4.75E-05	0.000164821	Lypd6
ncbi_101792348	0.166666667	0.703333333	2.077242999	4.80E-05	0.000166657	c1qtnf12
ncbi_101800713	0.096666667	1.26	3.704261429	4.92E-05	0.000170219	HBZ
ncbi_110351189	0.001	0.296666667	8.212699025	5.09E-05	0.000175888	Btn1a1
ncbi_101794930	3.36	0.583333333	-2.526068812	5.22E-05	0.000180121	Cdca3
ncbi_101795407	0.4	0.013333333	-4.906890596	5.25E-05	0.000181178	hnmt
ncbi_101799578	0.001	0.296666667	8.212699025	5.40E-05	0.000185829	unc93a
ncbi_101801710	0.053333333	0.26	2.285402219	5.66E-05	0.000194567	Rusc2
MSTRG.15727	0.303333333	1.316666667	2.117914203	5.73E-05	0.000196806	--
ncbi_101798928	1.326666667	0.306666667	-2.113062664	5.77E-05	0.000197976	LDLRAD1
ncbi_101798824	0.001	0.35	8.451211112	5.88E-05	0.00020128	OIH
ncbi_101793067	0.286666667	0.001	-8.163230349	5.99E-05	0.000204492	ANGPTL5
ncbi_101803961	0.236666667	0.001	-7.886712714	6.18E-05	0.000210295	Mlxipl
ncbi_101805181	0.493333333	0.066666667	-2.887525271	6.45E-05	0.000218613	SLC7A9
ncbi_101791820	1.446666667	0.11	-3.717157113	6.56E-05	0.000222001	MYL4
ncbi_101803135	3.083333333	0.726666667	-2.085125231	6.74E-05	0.000228175	tpx2-a

ncbi_101801400	0.353333333	0.013333333	-4.727920455	7.05E-05	0.000238031	Pld4
MSTRG.16250	2.36	0.463333333	-2.348664477	7.45E-05	0.000250964	melk
ncbi_113845031	0.073333333	0.53	2.853451337	7.48E-05	0.000251734	--
ncbi_113843843	0.076666667	0.443333333	2.531720479	7.60E-05	0.000255634	VWCE
ncbi_101798663	0.923333333	0.183333333	-2.332382453	7.64E-05	0.000256664	Rhob
ncbi_101800679	0.37	0.076666667	-2.27085391	8.12E-05	0.000272092	ANO3
ncbi_101799868	0.243333333	1.476666667	2.60133833	8.19E-05	0.000274232	NCOR1
ncbi_101790071	0.006666667	0.106666667	4	8.23E-05	0.000275311	Col14a1
ncbi_113842608	0.18	0.003333333	-5.754887502	8.29E-05	0.000277306	Tmie
ncbi_101800982	0.92	0.103333333	-3.154328146	8.55E-05	0.00028561	slc7a6
ncbi_101802933	0.783333333	0.016666667	-5.554588852	8.58E-05	0.00028637	Ca3
ncbi_101791678	0.316666667	0.02	-3.984893108	8.63E-05	0.000287953	Il12rb2
ncbi_101800820	0.59	2.85	2.27217506	8.98E-05	0.000298692	RALY
MSTRG.17682	1.23	0.02	-5.942514505	9.03E-05	0.00030006	--
ncbi_101797846	0.21	0.001	-7.714245518	9.32E-05	0.000308767	CCDC177
ncbi_101798037	0.306666667	0.013333333	-4.523561956	9.71E-05	0.000321151	CD200
ncbi_101798911	0.223333333	0.001	-7.803054785	0.000101996	0.000336585	rhbdf2
ncbi_113841371	0.07	0.63	3.169925001	0.000103564	0.000341503	ASZ1
ncbi_101794819	0.576666667	0.093333333	-2.627273306	0.000107317	0.000353074	STAT4
ncbi_101798162	0.001	0.36	8.491853096	0.000108601	0.000357026	OIH
ncbi_101804651	0.37	0.083333333	-2.150559677	0.000113259	0.000370846	Fbxo16
ncbi_101798158	0.153333333	0.966666667	2.656347134	0.000119151	0.000389256	TMOD4
ncbi_106014279	0.18	0.026666667	-2.754887502	0.000123426	0.000402316	COL7A1
ncbi_101800766	0.363333333	0.07	-2.375866902	0.000125186	0.000407645	ILDR1
ncbi_101802499	0.586666667	0.001	-9.196397213	0.000126793	0.000412464	VIP
ncbi_113845590	0.503333333	0.073333333	-2.778973121	0.00012978	0.000421027	CBARP
ncbi_101797082	0.18	1.163333333	2.692195724	0.000133155	0.000431437	TMEFF2
ncbi_101789465	0.576666667	0.133333333	-2.112700133	0.000135049	0.000436922	SH3BP1
ncbi_101796098	0.193333333	0.013333333	-3.857980995	0.000139155	0.000449538	GPR65
ncbi_101801371	0.153333333	0.003333333	-5.523561956	0.000139373	0.000450132	PTPRQ
ncbi_101794586	0.16	0.001	-7.321928095	0.000146168	0.000470678	ZNF366
ncbi_101803274	0.396666667	0.093333333	-2.087462841	0.00014914	0.000478946	PRAG1

ncbi_101797924	1.53	0.17	-3.169925001	0.000154756	0.000496247	KIF20A
ncbi_113844342	0.056666667	0.816666667	3.849175098	0.000160595	0.000513708	C4bpa
MSTRG.17732	3.633333333	0.646666667	-2.490199577	0.000161164	0.000515402	MKI67
ncbi_101801074	0.17	0.001	-7.409390936	0.000175979	0.000559967	ROBO4
ncbi_101799891	0.303333333	0.043333333	-2.807354922	0.000182267	0.000578487	Plekha6
ncbi_101799948	0.76	0.126666667	-2.584962501	0.00018893	0.000597453	BIN2
ncbi_101794572	0.52	0.093333333	-2.478047297	0.00019168	0.000605414	CDH1
ncbi_101795272	0.006666667	0.096666667	3.857980995	0.000194225	0.000612859	RAB3C
ncbi_101803226	0.086666667	0.626666667	2.854149134	0.000195636	0.000617014	CTLA4
ncbi_101798142	0.35	0.066666667	-2.392317423	0.000198149	0.000624486	MAP3K19
ncbi_101799944	0.41	2.25	2.456229187	0.000199055	0.000626803	CENPT
ncbi_110354560	0.443333333	0.001	-8.79224803	0.000199077	0.000626803	Krt17
ncbi_101792197	0.59	0.03	-4.297680549	0.000200097	0.000629407	--
ncbi_101798338	0.046666667	0.226666667	2.280107919	0.000214423	0.000671231	FRMPD4
ncbi_101799077	0.71	0.001	-9.471675214	0.000219964	0.000686761	Atp6v1g3
ncbi_101798459	0.056666667	0.263333333	2.216317907	0.000222641	0.000694122	GRID2
ncbi_101802855	0.62	0.053333333	-3.539158811	0.000223803	0.000697577	CAMK1G
ncbi_101797491	0.31	0.036666667	-3.079727192	0.000227104	0.000706855	--
ncbi_110354301	1.46	0.153333333	-3.251225104	0.00022786	0.000709035	CCL8
ncbi_101802309	0.166666667	0.01	-4.058893689	0.00023033	0.000716039	RGS6
ncbi_101798321	0.096666667	0.003333333	-4.857980995	0.000233828	0.000725876	IL6
ncbi_113842230	0.553333333	0.12	-2.20511443	0.000239681	0.000742454	--
ncbi_101804261	0.023333333	0.526666667	4.496425826	0.000250681	0.000773951	FBP2
ncbi_101790269	0.34	0.046666667	-2.86507042	0.000258934	0.000796978	CWH43
ncbi_101792921	0.103333333	0.013333333	-2.95419631	0.000303122	0.000925338	DNAH12
ncbi_101801718	0.13	0.016666667	-2.963474124	0.000305552	0.000932537	UNC80
ncbi_110351185	0.953333333	0.16	-2.574908836	0.00032513	0.000988812	TLR2-1
ncbi_101794283	0.076666667	0.41	2.418952549	0.000339755	0.001029215	HK1
ncbi_101790240	0.613333333	0.076666667	-3	0.00035174	0.001063831	ANKRD13B
ncbi_101799488	0.05	0.323333333	2.693022247	0.00035367	0.001069377	SLC35D3
ncbi_101793057	0.566666667	0.02	-4.824428435	0.00039861	0.00119776	KRT14
ncbi_101789750	0.14	0.673333333	2.26589406	0.000400824	0.001203303	FBXO15

ncbi_101803829	0.296666667	0.06	-2.30580843	0.000415226	0.001242247	LAMB3
ncbi_101800748	0.16	0.01	-4	0.000418352	0.001251311	ST18
ncbi_101791057	0.24	0.033333333	-2.847996907	0.000445565	0.00132844	CEP350
ncbi_101791758	0.633333333	0.056666667	-3.482392767	0.000445912	0.001328971	KRT19
ncbi_101796391	0.973333333	0.14	-2.797507136	0.000450458	0.00134088	Dnase1l3
ncbi_101796494	1.283333333	0.246666667	-2.37926127	0.000452087	0.001344899	--
ncbi_101796299	0.001	0.34	8.409390936	0.000455212	0.001353797	Syndig1
ncbi_101800947	0.16	0.001	-7.321928095	0.000470256	0.001394718	CAPN9
ncbi_101803082	0.533333333	0.11	-2.277533976	0.000493386	0.001457019	GANAB
ncbi_101789851	0.146666667	0.001	-7.196397213	0.000495029	0.001461541	GPR183
ncbi_101803777	0.996666667	0.14	-2.831684251	0.000529851	0.001556595	TNFSF13B
ncbi_101796333	0.333333333	0.023333333	-3.836501268	0.000555389	0.001629048	OSTN
ncbi_101796606	0.966666667	0.16	-2.594946589	0.00056215	0.001647768	KMO
ncbi_101799881	0.11	0.001	-6.781359714	0.000566611	0.001659724	SLC34A1
ncbi_101797413	0.423333333	0.003333333	-6.988684687	0.000573437	0.001676706	KIAA0825
ncbi_101795771	0.186666667	0.01	-4.222392421	0.000574247	0.001678698	GCNT2
ncbi_101794779	1.27	0.103333333	-3.619450877	0.000595825	0.001735159	ptcd2
ncbi_101798763	1.176666667	0.103333333	-3.509328063	0.000596917	0.001737566	CDCA4
ncbi_101800572	0.006666667	0.22	5.044394119	0.000605451	0.001760439	LRRC2
ncbi_101804796	2.463333333	0.023333333	-6.722075632	0.000610143	0.001772503	SUFU
ncbi_101796407	0.146666667	0.001	-7.196397213	0.000621064	0.001803825	--
ncbi_113843852	0.186666667	0.001	-7.544320516	0.00068153	0.001966736	Mrgprd
ncbi_101799253	0.376666667	0.053333333	-2.820178962	0.000716179	0.002059428	WT1
ncbi_101795437	0.083333333	0.001	-6.380821784	0.000721772	0.002075055	A2ML1
ncbi_101795080	0.393333333	0.043333333	-3.182203331	0.00072746	0.002088643	OR51E2
ncbi_101797799	0.306666667	0.026666667	-3.523561956	0.000782026	0.002235953	Hoxc5
ncbi_113845664	0.663333333	0.063333333	-3.388697107	0.000813981	0.002317659	Traf4
ncbi_101794316	0.143333333	0.033333333	-2.10433666	0.000818151	0.002328515	TH
ncbi_101790806	0.16	0.013333333	-3.584962501	0.000847656	0.00240828	CACNA2D3
MSTRG.1984	1.296666667	0.146666667	-3.144194726	0.000916112	0.00259035	Ppfia2
ncbi_101798651	0.086666667	0.4	2.206450877	0.000943844	0.002662989	VNN1
ncbi_101789714	0.146666667	0.013333333	-3.459431619	0.000951797	0.002683762	TEK

ncbi_101798916	0.436666667	0.093333333	-2.226068079	0.000957437	0.002697837	SLC13A3
ncbi_101796922	0.15	0.82	2.450661409	0.000984726	0.002768148	DLX5
ncbi_106016036	0.06	0.7	3.544320516	0.000989378	0.002779428	dusp22a
ncbi_101801396	0.493333333	0.056666667	-3.121990524	0.000993991	0.002789982	Nqo2
ncbi_113843266	0.196666667	0.01	-4.297680549	0.001002819	0.002812943	--
ncbi_101792097	0.226666667	0.001	-7.824428435	0.001018815	0.002853514	ARSD
ncbi_110351440	0.693333333	0.001	-9.437405312	0.001065331	0.002976122	HIST2H2AC
ncbi_101802190	0.026666667	0.293333333	3.459431619	0.001081759	0.003021369	selenbp1
ncbi_101801549	0.26	0.006666667	-5.285402219	0.00109614	0.003056949	Nipal1
ncbi_101803487	0.13	0.013333333	-3.285402219	0.001134901	0.003158966	Cnih3
ncbi_101799471	0.001	0.16	7.321928095	0.001147427	0.003189743	GPR37
ncbi_101795620	1.003333333	0.143333333	-2.807354922	0.001159957	0.003222514	SLC25A21
ncbi_101795011	0.596666667	0.143333333	-2.057551023	0.001169749	0.003247642	GYS2
ncbi_101797733	0.266666667	1.263333333	2.244125943	0.001188182	0.003296712	drd1
ncbi_101805432	0.446666667	0.033333333	-3.744161096	0.001196586	0.003317205	CYP2C19
ncbi_101799169	0.676666667	0.1	-2.758445322	0.001205941	0.003341719	TSPAN13
ncbi_101791292	0.193333333	0.001	-7.594946589	0.001217668	0.003373495	TGM3
ncbi_113843868	0.653333333	0.001	-9.351675438	0.0012818	0.003535392	gacHH
ncbi_101791537	0.076666667	0.32	2.061400545	0.001302939	0.00359074	trabd2a
ncbi_101793004	0.023333333	0.123333333	2.402098444	0.00132795	0.003651091	Kcnd3
ncbi_101804072	0.233333333	0.053333333	-2.129283017	0.001338114	0.00367516	KIAA1211L
ncbi_101789607	0.043333333	0.396666667	3.194378045	0.001352933	0.003712844	HTR5A
ncbi_101790234	0.136666667	0.593333333	2.118181426	0.001352973	0.003712844	CHGA
ncbi_101792807	1.903333333	0.413333333	-2.203150625	0.001428632	0.003901575	ZDHHC20
ncbi_101793581	2.346666667	0.496666667	-2.240263098	0.001429818	0.003903995	Pkia
ncbi_101790233	1.673333333	0.156666667	-3.416954702	0.001430358	0.003904653	SKA1
ncbi_101791894	0.17	0.036666667	-2.212993723	0.001478538	0.004028583	CALN1
ncbi_101794331	0.223333333	0.001	-7.803054785	0.001483319	0.004039076	--
ncbi_106017129	1.023333333	0.196666667	-2.379451796	0.001531253	0.004158309	TIRAP
ncbi_101801254	1.19	0.203333333	-2.549042926	0.001536807	0.004171652	--
ncbi_113844954	0.001	0.496666667	8.956134115	0.001544935	0.004191974	--
ncbi_113843045	2.64	0.606666667	-2.12156198	0.00162294	0.00438537	Hist1h3b

ncbi_101790257	0.123333333	0.693333333	2.490986353	0.001677084	0.004521364	cckar
ncbi_101790047	0.75	0.146666667	-2.354349573	0.001677861	0.004522523	--
ncbi_101799598	0.42	0.09	-2.222392421	0.001696868	0.004568088	Card10
ncbi_113840906	0.836666667	0.043333333	-4.271103836	0.001749889	0.004698208	ATAD2
ncbi_101794613	0.083333333	0.013333333	-2.64385619	0.001790073	0.004800166	CFAP54
ncbi_101796578	0.27	0.006666667	-5.339850003	0.001797209	0.004816327	gsg1l
ncbi_101792199	0.753333333	0.16	-2.235216462	0.001798365	0.004818437	SLC17A9
ncbi_101797179	0.11	0.016666667	-2.722466024	0.001805159	0.004834651	MUC5B
ncbi_113844391	0.726666667	0.13	-2.482782106	0.001833589	0.004906762	Egr2
ncbi_101797959	0.383333333	0.026666667	-3.845490051	0.001885121	0.005031235	--
ncbi_101792419	0.346666667	0.033333333	-3.378511623	0.001963971	0.005227766	TSPAN2
ncbi_101803191	0.773333333	0.1	-2.9510904	0.002071703	0.005493216	CFAP161
ncbi_113845289	0.001	0.646666667	9.336878436	0.00207303	0.005494499	Msrb1
ncbi_101802740	0.206666667	0.013333333	-3.95419631	0.002078918	0.005507866	DDB_G0282555
ncbi_101791567	0.023333333	0.2	3.099535674	0.002131093	0.005638076	Igsf5
ncbi_101797192	0.043333333	0.67	3.950611973	0.002201104	0.005806798	ERCC4
ncbi_101793229	0.12	0.49	2.029747343	0.002252588	0.005933015	RASL12
ncbi_101790069	0.966666667	0.163333333	-2.565199246	0.002263766	0.005960047	LYG2
ncbi_113842418	1.066666667	0.03	-5.152003093	0.002286852	0.00601354	CSTA
ncbi_101793261	0.283333333	0.006666667	-5.409390936	0.002290858	0.006022858	MPZL2
ncbi_101802165	0.583333333	16.94333333	4.860253404	0.002352394	0.006163509	Myh8
ncbi_101797759	0.093333333	0.001	-6.544320516	0.002391353	0.006259295	--
ncbi_101795194	0.123333333	0.023333333	-2.402098444	0.002395824	0.006269738	Dlgap2
ncbi_101799156	0.613333333	0.096666667	-2.665580961	0.002396491	0.006270224	TMEM173
ncbi_101789461	0.426666667	0.096666667	-2.142019005	0.002438652	0.006374139	Ush1c
ncbi_110353607	0.443333333	0.053333333	-3.055282436	0.002449636	0.006399	LIF
ncbi_101801420	0.686666667	0.16	-2.101538026	0.002455498	0.00641027	ABI3BP
ncbi_113839600	0.773333333	0.076666667	-3.334419039	0.002526886	0.006582325	Dnajb13
ncbi_113841056	0.001	0.836666667	9.708509148	0.002530022	0.006589179	NEL
ncbi_101802273	0.62	0.133333333	-2.217230716	0.00253677	0.006605432	Tex12
ncbi_101793727	0.653333333	0.1	-2.707819249	0.002664676	0.00691225	--
ncbi_101798540	3.79	0.756666667	-2.324468052	0.002677332	0.006939556	TK1

ncbi_101805481	0.001	0.223333333	7.803054785	0.002679292	0.006941874	Nipal2
ncbi_110353045	0.373333333	1.85	2.308989039	0.002710749	0.007016406	Commd4
ncbi_101800905	0.05	0.001	-5.64385619	0.002747233	0.007103786	MYO7B
ncbi_106020544	0.286666667	0.016666667	-4.10433666	0.002822833	0.007286263	RUFY4
ncbi_101796414	0.08	0.323333333	2.014950341	0.002898367	0.007470875	LMOD3
ncbi_101796159	0.356666667	0.033333333	-3.419538892	0.002912301	0.007505307	P2RY1
ncbi_113845380	1.716666667	0.343333333	-2.321928095	0.002993547	0.007691875	CLEC2D
ncbi_101792912	0.273333333	0.013333333	-4.357552005	0.00303291	0.007785344	CYP2J2
ncbi_101801266	0.283333333	0.05	-2.502500341	0.003065269	0.007860669	AGT
ncbi_101794421	0.21	0.001	-7.714245518	0.003086759	0.007906699	CX3CR1
ncbi_101792815	1.143333333	0.146666667	-2.962633148	0.003251393	0.008285807	PMP2
ncbi_101803518	0.033333333	1.226666667	5.201633861	0.003274315	0.00833933	SAP30L
ncbi_113841212	0.076666667	0.543333333	2.825166198	0.003275332	0.00834029	--
ncbi_101802338	0.2	0.013333333	-3.906890596	0.003326797	0.008453178	Syt12
ncbi_101802138	0.336666667	0.073333333	-2.198779864	0.003430123	0.008692	HTR1D
ncbi_101797172	0.001	0.126666667	6.984893108	0.003476325	0.008803943	FBXO33
ncbi_101800543	0.003333333	0.106666667	5	0.003498179	0.00885241	KCNJ3
ncbi_101804545	0.596666667	0.073333333	-3.024384159	0.003519793	0.008901921	Azin2
ncbi_101791407	0.143333333	0.003333333	-5.426264755	0.003535679	0.008940363	COLEC10
ncbi_113843417	0.006666667	0.15	4.491853096	0.003623879	0.009145646	HMX1
ncbi_101797473	0.333333333	0.001	-8.380821784	0.003811866	0.009579269	CMTM8
ncbi_101796684	0.11	0.003333333	-5.044394119	0.003939838	0.009881815	TRPC7
ncbi_101802566	0.353333333	0.066666667	-2.40599236	0.003941625	0.009882494	GUCA1A
ncbi_101804593	0.003333333	0.05	3.906890596	0.003961045	0.009925457	ZMAT4
ncbi_101794945	0.453333333	0.07	-2.695145418	0.004053424	0.010135597	ARHGAP8
ncbi_113840301	0.783333333	0.026666667	-4.876516947	0.004124943	0.010298532	GLRX
ncbi_101802474	0.096666667	0.636666667	2.719447833	0.00415023	0.010353733	C12orf65
ncbi_101790323	0.183333333	0.02	-3.196397213	0.004302628	0.010711374	HEPHL1
ncbi_101801414	0.14	0.003333333	-5.392317423	0.004322644	0.010757096	PRLHR
ncbi_101802491	0.203333333	0.026666667	-2.930737338	0.004397135	0.010927864	C10orf90
ncbi_101799781	2.59	0.453333333	-2.514307947	0.004411592	0.010961705	TST
ncbi_101795741	0.001	0.14	7.129283017	0.004428503	0.01099953	PLIN1

ncbi_101802374	0.316666667	0.036666667	-3.11042399	0.004525229	0.011220536	SPTLC3
ncbi_101795086	0.196666667	0.01	-4.297680549	0.004575176	0.011335756	KCNQ4
ncbi_101793286	0.176666667	0.013333333	-3.727920455	0.004623434	0.011438243	MAEL
ncbi_101799392	0.63	0.156666667	-2.007653573	0.004668986	0.011548431	P2RY3
ncbi_101800584	0.803333333	0.096666667	-3.054908341	0.00467179	0.011553176	KCNMB2
ncbi_101802540	0.26	0.001	-8.022367813	0.004733799	0.011686566	TECTB
ncbi_101795292	0.11	0.001	-6.781359714	0.00483349	0.011916878	AHR
ncbi_101792812	0.253333333	0.01	-4.662965013	0.004949329	0.012161074	TMEM269
ncbi_101797191	0.033333333	0.203333333	2.608809243	0.004960651	0.012184789	PCDHA2
ncbi_101798059	0.1	0.003333333	-4.906890596	0.004968078	0.012197586	SLC26A9
ncbi_101803666	1.41	0.123333333	-3.515060487	0.004981915	0.012225013	ITGB3
ncbi_101803729	0.306666667	0.013333333	-4.523561956	0.005087364	0.012453293	C2orf54
ncbi_106016138	0.3	0.01	-4.906890596	0.005118209	0.012512352	--
ncbi_110354421	0.25	0.05	-2.321928095	0.005309978	0.012954439	--
ncbi_101803962	0.45	0.07	-2.684498174	0.005368135	0.013084077	Nme1
ncbi_101804950	0.24	0.033333333	-2.847996907	0.005567507	0.013519459	PODXL
ncbi_113840807	0.336666667	0.083333333	-2.014355293	0.005619454	0.013630366	--
ncbi_101794789	0.233333333	0.01	-4.544320516	0.00567126	0.013743236	PVALB
ncbi_101790654	0.043333333	1.24	4.838719093	0.005674822	0.013749313	MEGF11
ncbi_113839978	0.226666667	0.001	-7.824428435	0.005790973	0.014007299	KRT8
ncbi_101794280	0.093333333	0.006666667	-3.807354922	0.005800485	0.014027704	CHRNA9
ncbi_101805398	0.503333333	0.073333333	-2.778973121	0.005861227	0.014158838	Ubap1
ncbi_101796528	0.433333333	0.046666667	-3.215012891	0.00592314	0.014297802	rasl11b
ncbi_101791306	0.406666667	0.05	-3.023846742	0.005986399	0.014442479	C2cd4cC2CD4
ncbi_101791808	0.363333333	0.063333333	-2.520256811	0.006194318	0.014891726	CIB3
ncbi_101804930	0.076666667	0.006666667	-3.523561956	0.006299994	0.015102855	Gabrr3
ncbi_101800542	0.176666667	0.013333333	-3.727920455	0.006343088	0.01519334	ENPP3
ncbi_113839714	0.486666667	0.06	-3.019899557	0.006370322	0.015255768	AADACL4
ncbi_101800088	0.1	0.506666667	2.341036918	0.006534726	0.015615061	Rsph1
ncbi_101791361	0.61	0.056666667	-3.428236997	0.0065807	0.015713395	GSC
ncbi_101800039	0.166666667	0.013333333	-3.64385619	0.00676233	0.016105788	Hgfac
ncbi_101798764	0.396666667	0.073333333	-2.435386145	0.006809872	0.016216055	Dnaaf1

ncbi_101800307	0.09	0.013333333	-2.754887502	0.006959217	0.016526398	CNTNAP5
ncbi_101802353	0.001	0.2	7.64385619	0.007283675	0.017230981	TCF21
ncbi_101795930	0.25	0.033333333	-2.906890596	0.00738005	0.017433662	TMEM72
ncbi_101796210	0.06	0.001	-5.906890596	0.007479386	0.017652327	GRID2IP
ncbi_101796632	0.516666667	0.106666667	-2.276124405	0.00760043	0.0179153	NEIL3
ncbi_101802200	0.03	0.001	-4.906890596	0.007685105	0.018095258	Dnah1
ncbi_101802936	0.693333333	0.156666667	-2.145850866	0.007723039	0.018168168	St3gal2
ncbi_101794244	0.013333333	0.086666667	2.700439718	0.007836533	0.018405263	NPY6R
ncbi_101792707	0.023333333	0.16	2.777607579	0.007877864	0.018485472	SCNN1A
ncbi_101793245	0.05	0.27	2.432959407	0.007879193	0.018485472	--
ncbi_101798576	0.11	0.001	-6.781359714	0.007896101	0.018515144	SCARF1
ncbi_101794833	0.026666667	0.13	2.285402219	0.007920916	0.018566649	COL22A1
ncbi_101795396	0.55	0.136666667	-2.00877021	0.007957363	0.018638675	POLR3G
ncbi_113844894	0.096666667	0.596666667	2.625834782	0.008090641	0.018930444	IL34
ncbi_101802629	0.236666667	0.001	-7.886712714	0.008188165	0.019131159	COQ3
ncbi_101789652	0.216666667	0.016666667	-3.700439718	0.008266205	0.019299657	--
ncbi_101794038	0.413333333	0.046666667	-3.146841388	0.008295554	0.01936471	--
ncbi_101801133	0.253333333	0.001	-7.984893108	0.008324383	0.019414624	Sla2
MSTRG.2660	0.39	1.713333333	2.13525983	0.008419121	0.019621535	--
ncbi_101789878	0.136666667	0.013333333	-3.357552005	0.008692003	0.02017814	CCR7
ncbi_101802176	0.003333333	0.113333333	5.087462841	0.008786235	0.020364255	CCKAR
ncbi_101802695	0.086666667	0.42	2.276840205	0.009040326	0.020915984	RXFP3
ncbi_101800816	0.001	2.26	11.14210706	0.009080067	0.020996749	PRICKLE1
ncbi_101797068	0.273333333	0.06	-2.187627003	0.009212693	0.021269475	Slc39a10
ncbi_101792276	0.203333333	0.01	-4.345774837	0.00925829	0.021359613	3-Sep
ncbi_101797613	0.116666667	0.02	-2.544320516	0.009361755	0.021567777	Col4a3
ncbi_101795032	0.136666667	0.016666667	-3.03562391	0.009424001	0.021699673	C4B
ncbi_101797962	0.593333333	0.063333333	-3.227805918	0.009580166	0.022024245	Creb5
ncbi_101803956	0.21	0.03	-2.807354922	0.009657626	0.022182758	SYT5
ncbi_101798626	0.073333333	0.001	-6.196397213	0.009883925	0.022646679	ESR2
ncbi_101798705	0.03	0.183333333	2.611434712	0.009903626	0.022683847	KCNH1

Description: Sample: id: gene id; IMP0-1_count: gene count value for IMP0-1; IMP0-4_count: gene count value for IMP0-4; log2(FC): logarithmic value of the multiplicity of difference in FPKM between samples IMP0 and IMP4, bottomed by 2; P_value: significance P-value; FDR: P-value after BH correction; Symbol: gene name; Symbol. Gene name; KEGG_A_class KEGG: first level annotation; KEGG_B_class: KEGG second level annotation; K_ID: KEGG pathway ID.
