# Supplementary material for: Identification of genes and long non-coding RNAs for intramuscular and subcutaneous fat deposition in ducks by transcriptome analysis
Source: Anim Biosci. 2025 Aug 12;39(1):250268. doi: 10.5713/ab.25.0268 (PMC12754461; doi:10.5713/ab.25.0268)
Supplement: Supplementary file 4 [file ab-25-0268-Supplementary-4.pdf]

Supplement 4. DemRNAs of SCP-0-vs-SCP-4-

id	SCP-0_fpkm_mean	SCP-4_fpkm_mean	log2(fc)	PValue	FDR	Symbol
ncbi_101789686	986.0366667	150.82	-2.708813541	0	0	--
ncbi_101790193	327.17	26.58666667	-3.621265652	0	0	CTGF
ncbi_101790783	12.23333333	65.34333333	2.41722249	0	0	Adamts1
ncbi_101790965	41.74333333	216.08	2.371947831	0	0	Ndrg1
ncbi_101791542	40.78	2.8	-3.864363043	0	0	CLMN
ncbi_101792143	4.65	28.53	2.617177126	0	0	TSPAN4
ncbi_101792626	13.88	345.65	4.638232453	0	0	FABP4
ncbi_101792633	0.246666667	13.04666667	5.724974675	0	0	SLC15A2
ncbi_101792965	36.02	168.5066667	2.225935584	0	0	RDH10
ncbi_101793407	0.603333333	24.3	5.331857212	0	0	TENT5C
ncbi_101793497	643.2866667	44.64666667	-3.848837414	0	0	ACTA2
ncbi_101793749	645.89	12.45666667	-5.696298516	0	0	Mmp7
ncbi_101793891	12.75666667	170.5366667	3.740758657	0	0	EPHB1
ncbi_101793981	126.2966667	687.9	2.445382293	0	0	GSN
ncbi_101794506	3.63	20.52	2.498989278	0	0	KLF15
ncbi_101794773	37.70666667	2.41	-3.967714568	0	0	FBLN1
ncbi_101795371	648.61	97.29	-2.736987837	0	0	Rlc-a
ncbi_101795664	4.903333333	62.62666667	3.674942347	0	0	ABCC9
ncbi_101795864	5.206666667	78.08666667	3.906644276	0	0	Kcne4
ncbi_101795897	18.63666667	76.44	2.036183934	0	0	Capn5
ncbi_101795981	47.14666667	2.82	-4.063388706	0	0	Gpc3
ncbi_101796074	161.67	23.31333333	-2.793824792	0	0	FLNB
ncbi_101796345	38.25	4.6	-3.055753982	0	0	EPAS1

ncbi_101796701	89.37666667	534.24	2.579517853	0	0	Serpinf1
ncbi_101797476	157.5333333	24.24666667	-2.699798798	0	0	St14
ncbi_101798095	64.00333333	334.06	2.383888298	0	0	SFXN1
ncbi_101798249	19.84	91.92	2.211966772	0	0	Acss1
ncbi_101798353	2.223333333	30.66333333	3.785718371	0	0	CACNB4
ncbi_101798493	186.3966667	4.516666667	-5.366973805	0	0	Sdc1
ncbi_101799001	138.7666667	9.296666667	-3.899803719	0	0	CYR61
ncbi_101799119	343.5	9.373333333	-5.1956041	0	0	SERPINB2
ncbi_101799167	8.836666667	46.55333333	2.397310303	0	0	PDE7B
ncbi_101799393	169.8766667	33.05666667	-2.361474545	0	0	COL11A1
ncbi_101799557	56.5	288.9333333	2.35441388	0	0	PLPP1
ncbi_101799687	25.78	107.3733333	2.058311569	0	0	COL14A1
ncbi_101799717	7.04	137.5933333	4.288691331	0	0	PIK3R1
ncbi_101799854	41.68666667	206.9766667	2.311810214	0	0	Ifngr1
ncbi_101800131	12.97666667	275.3933333	4.407501886	0	0	Rbp7
ncbi_101800175	915.7	99.37333333	-3.203944359	0	0	Fn1
ncbi_101800352	18.88666667	91.79333333	2.281021278	0	0	FAM174B
ncbi_101800454	1.71	40.28	4.557995453	0	0	DEPTOR
ncbi_101800506	15.57	96.53333333	2.632258252	0	0	YPEL1
ncbi_101800608	66.53666667	4.62	-3.848184836	0	0	Dner
ncbi_101800839	167.06	32.39666667	-2.366449058	0	0	Mfge8
ncbi_101801449	86.13333333	19.18333333	-2.166718237	0	0	Vgll3
ncbi_101801527	129.2266667	557.1933333	2.108274187	0	0	FSTL1
ncbi_101801623	18.63	145.1666667	2.962010639	0	0	TEX2
ncbi_101801672	0.91	58.91333333	6.016583828	0	0	STC2

ncbi_101802129	113.1966667	2.87	-5.301636928	0	0	CEMIP
ncbi_101802143	3.52	41.69666667	3.566284722	0	0	ZBTB16
ncbi_101802361	69.87333333	2.87	-4.605619324	0	0	LRRN4
ncbi_101802643	32.84	428.0833333	3.704365636	0	0	C2orf40
ncbi_101802795	237.14	58.68333333	-2.014716306	0	0	MYH9
ncbi_101803155	2.56	30.53	3.576011874	0	0	SLC40A1
ncbi_101803411	15.83	229.84	3.859896737	0	0	IGFBP5
ncbi_101804284	549.9033333	97.65	-2.493486083	0	0	CCDC80
ncbi_101804475	87.50666667	2.073333333	-5.399368948	0	0	TMEM2
ncbi_101804496	2.37	49.58666667	4.386993283	0	0	CYP2D20
ncbi_101804683	1.123333333	16.23666667	3.853397487	0	0	PIEZO2
ncbi_101804896	566.8133333	112.9	-2.32782821	0	0	TNC
ncbi_113839853	354.4533333	13.08333333	-4.759793639	0	0	TAGLN
ncbi_113840895	1127.99	107.2433333	-3.394794406	0	0	FN1
ncbi_113841361	184.1133333	1091.463333	2.567597651	0	0	BTG1
ncbi_113841776	15.84666667	79.52666667	2.3272593	0	0	SH3D19
ncbi_113842398	29.55	4.543333333	-2.701335069	0	0	OCC1
ncbi_101789694	7.28	91.34333333	3.649289082	5.53E-308	8.22E-306	IGFBP4
ncbi_101793994	60.82666667	6.743333333	-3.173170127	1.09E-307	1.60E-305	PPP1R3C
ncbi_101796495	0.686666667	19.87333333	4.855080133	4.15E-307	6.02E-305	CD36
ncbi_101795655	16.91666667	0.913333333	-4.211160024	1.00E-304	1.44E-302	PKP2
ncbi_101792740	7.82	55.11	2.817073615	1.24E-301	1.72E-299	ACKR3
ncbi_101799368	83.81666667	5.83	-3.845669359	6.81E-297	9.38E-295	MYL9
ncbi_101799386	41.43333333	1.6	-4.69464808	6.12E-296	8.33E-294	LINGO1
ncbi_101798279	14.33666667	77.58333333	2.436037131	1.61E-294	2.15E-292	narfl

ncbi_101797140	5.913333333	70.77333333	3.581162361	1.68E-293	2.22E-291	APOA1
ncbi_101804310	170.3233333	30.17	-2.497089488	5.62E-284	7.27E-282	TIMP3
ncbi_101794356	2.36	40.19666667	4.090217105	7.20E-284	9.23E-282	Tmem100
ncbi_101804763	41.95666667	7.686666667	-2.448470052	1.74E-283	2.21E-281	Kcnk1
ncbi_101802853	2.743333333	14.91	2.442278423	8.56E-279	1.07E-276	SORCS2
ncbi_101805263	57.30666667	9.846666667	-2.540995657	1.56E-276	1.93E-274	Tmem47
MSTRG.886	4.483333333	0.343333333	-3.70688993	4.05E-275	4.90E-273	RTase
ncbi_101795823	25.88333333	3.57	-2.858027445	8.94E-271	1.06E-268	ANLN
ncbi_101792348	0.51	21.70333333	5.41127558	3.85E-269	4.49E-267	c1qtnf12
ncbi_101801129	17.84666667	0.633333333	-4.816545815	6.25E-269	7.23E-267	FAM198B
MSTRG.3206	0.49	5.503333333	3.48945206	1.51E-265	1.71E-263	--
ncbi_101792619	40.76	0.18	-7.823013335	8.80E-263	9.92E-261	TIMP4
ncbi_101792694	79.80333333	8.136666667	-3.293939214	2.37E-261	2.65E-259	MFAP5
ncbi_101791868	51.11666667	7.476666667	-2.773326641	2.61E-258	2.87E-256	MYO1D
ncbi_101796233	2.096666667	22.83333333	3.444972066	4.27E-257	4.65E-255	LVRN
ncbi_101794003	16.22	72.65	2.163188978	2.18E-256	2.35E-254	MYLIP
ncbi_101803099	117.38	18.93333333	-2.632186279	8.83E-256	9.47E-254	TUBB6
ncbi_101802641	46.29333333	210.4366667	2.184509751	2.16E-247	2.28E-245	DPT
ncbi_101798843	8.72	74.75	3.099673539	7.22E-245	7.56E-243	PDGFD
ncbi_101800386	23.7	3.456666667	-2.777433666	1.16E-244	1.20E-242	Jag2
ncbi_101796751	13.53333333	2.61	-2.374395515	8.63E-242	8.89E-240	DST
ncbi_110352128	53.54	1.58	-5.082620675	1.12E-235	1.13E-233	WISP2
ncbi_101804995	0.356666667	16.76666667	5.554875698	2.77E-233	2.74E-231	FGFR3
ncbi_101799485	75.00333333	9.643333333	-2.959350891	7.69E-233	7.56E-231	ITGA4
ncbi_101798758	60.58333333	6.53	-3.213766061	2.29E-228	2.17E-226	FADS2

ncbi_101800607	3.99	52.31	3.712626118	1.32E-224	1.22E-222	LY86
ncbi_101794662	1.116666667	19.84333333	4.151383894	2.76E-224	2.55E-222	NT5E
ncbi_101802983	34.80333333	4.37	-2.993520304	5.86E-224	5.36E-222	MGLL
ncbi_101803839	46.42666667	7.213333333	-2.686215704	6.63E-223	5.98E-221	--
ncbi_101792229	1.15	19.24	4.064403033	5.81E-221	5.20E-219	ICOSLG
ncbi_101792773	93.70666667	12.21	-2.94008849	4.32E-220	3.84E-218	--
ncbi_101798031	15.68333333	0.733333333	-4.418619294	3.43E-219	3.01E-217	NTN1
ncbi_101804308	2.903333333	20.18333333	2.797382336	7.39E-219	6.44E-217	MMP23
ncbi_101799708	12.58	62.79666667	2.319556059	5.43E-218	4.70E-216	LHFPL6
ncbi_101794926	8.363333333	107.4433333	3.683354094	5.97E-215	5.11E-213	PLAC9
ncbi_101800011	136.6433333	28.76666667	-2.24794511	3.73E-214	3.17E-212	COL12A1
ncbi_101800553	29.16333333	7.283333333	-2.001484848	2.49E-213	2.10E-211	SLC5A3
ncbi_101795217	229.0433333	36.84	-2.636275605	1.52E-212	1.27E-210	ACTN1
ncbi_101804791	9.416666667	43.98666667	2.22377791	1.33E-211	1.10E-209	SPRY2
ncbi_101803812	40.85666667	7.103333333	-2.524003413	1.25E-210	1.02E-208	SEMA3D
ncbi_101799004	1.816666667	17.72666667	3.286555642	8.88E-210	7.22E-208	Ggt5
ncbi_101799123	0.853333333	18.20333333	4.414949441	2.67E-205	2.10E-203	ENPP2
ncbi_101790851	28.63	3.193333333	-3.16439261	5.23E-204	4.09E-202	CHKA
ncbi_101797405	193.34	37.23666667	-2.376344309	4.89E-203	3.81E-201	CD44
ncbi_101801872	21.74666667	3.856666667	-2.495367918	2.87E-195	2.17E-193	MYO10
ncbi_101797297	22.41	1.39	-4.010985861	5.39E-194	3.96E-192	PERP
ncbi_101793568	8.133333333	0.14	-5.86034801	2.04E-188	1.47E-186	PTPRZ1
ncbi_101797220	7.12	0.22	-5.016301812	6.43E-187	4.62E-185	--
ncbi_101796901	31.77	3.426666667	-3.212787326	3.14E-185	2.23E-183	ASPN
ncbi_101803192	35.93666667	3.096666667	-3.536668596	2.10E-183	1.48E-181	SMC4

ncbi_101794671	1.066666667	7.076666667	2.729960561	3.61E-179	2.53E-177	adam22
ncbi_101791510	12.49666667	0.663333333	-4.23566549	1.27E-178	8.84E-177	PARD6B
ncbi_101803448	6.84	48.08666667	2.813568693	1.60E-175	1.08E-173	mmp11
ncbi_101800480	5.13	24.53	2.257516503	1.12E-174	7.53E-173	ST8SIA2
ncbi_101791702	66.07333333	7.91	-3.062318529	4.71E-174	3.16E-172	MXRA5
ncbi_101792888	14.26666667	3.106666667	-2.199208937	4.09E-171	2.73E-169	NSD2
ncbi_101800981	0.903333333	34.20333333	5.242734676	2.67E-170	1.76E-168	Limd2
ncbi_101802443	0.166666667	6.25	5.22881869	5.39E-170	3.54E-168	SLC6A2
ncbi_101793573	26.44333333	4.87	-2.440910371	3.43E-167	2.20E-165	STK17A
ncbi_101797574	108.27	14.60666667	-2.88993466	1.25E-164	7.86E-163	CTHRC1
ncbi_101790132	70.44666667	10.92	-2.689558588	1.63E-164	1.02E-162	ODC1
ncbi_113843234	5.05	65.44666667	3.695964422	7.79E-164	4.85E-162	LAMA2
ncbi_101799058	1.17	9.556666667	3.029998969	2.38E-163	1.47E-161	Tril
ncbi_101796514	31.76333333	136.2233333	2.100539614	4.34E-161	2.65E-159	OGN
ncbi_101801342	33.44666667	7.903333333	-2.081329275	1.17E-160	7.09E-159	SPECC1
ncbi_101800212	7.94	34.26	2.109314239	2.74E-160	1.65E-158	ENPP1
ncbi_101795759	17.53333333	3.853333333	-2.185921402	7.16E-160	4.27E-158	Pik3r1
ncbi_101796234	0.163333333	7.373333333	5.496425826	1.53E-158	9.04E-157	Avpr1a
ncbi_101796303	20.58333333	3.06	-2.749873078	8.34E-158	4.87E-156	GJC1
ncbi_101789635	14.80666667	61.34	2.050581274	2.33E-157	1.35E-155	HIST1H1C
ncbi_101798327	0.923333333	8.276666667	3.16412638	7.68E-157	4.42E-155	WNT11
ncbi_101796014	5.736666667	27.39666667	2.255715776	3.59E-156	2.06E-154	SRGAP3
ncbi_101799522	16.47333333	3.933333333	-2.066308151	4.56E-156	2.61E-154	Scube1
ncbi_101803645	0.706666667	7.493333333	3.406505866	3.90E-152	2.14E-150	ADGRL3
ncbi_101803788	20.36	82.03333333	2.010472691	5.55E-152	3.04E-150	rab18b

ncbi_101790142	26.58666667	1.88	-3.821898342	1.72E-150	9.27E-149	--
ncbi_101795474	9.86	0.15	-6.038553241	5.86E-150	3.15E-148	PAK1
ncbi_101796169	13.00666667	0.803333333	-4.017108726	2.07E-149	1.11E-147	Ntn4
ncbi_101797658	151.7733333	21.94666667	-2.78984459	3.47E-147	1.84E-145	Hspa2
ncbi_101791715	2.26	21.69	3.262635375	5.16E-146	2.70E-144	VEGFD
ncbi_101796141	2.956666667	16.74	2.501256019	9.91E-145	5.10E-143	Pdk4
ncbi_101791841	19.95666667	1.396666667	-3.836811121	5.56E-144	2.82E-142	TNFRSF11B
ncbi_101795038	2.76	20.51333333	2.893821771	7.79E-143	3.87E-141	--
ncbi_101794475	13.24333333	0.89	-3.895317146	6.53E-142	3.22E-140	HRH1
ncbi_101799604	38.15	8.23	-2.212718721	1.85E-141	9.11E-140	ALCAM
ncbi_101800897	11.03333333	54.53666667	2.305357808	7.56E-139	3.66E-137	DHRS7
ncbi_106019618	114.2366667	15.88666667	-2.846137433	8.17E-139	3.94E-137	TMSB15B
ncbi_101790288	27.76666667	6.803333333	-2.029040313	2.38E-136	1.13E-134	wtip
ncbi_101793330	17.63666667	2.033333333	-3.11665927	3.01E-136	1.42E-134	MKX
ncbi_101799950	12.81	0.293333333	-5.448585642	5.82E-136	2.72E-134	CTNNA3
ncbi_101789503	1.506666667	11.20666667	2.894925047	1.94E-133	8.93E-132	PRG4
ncbi_101792430	37.93	7.456666667	-2.346736616	2.00E-133	9.19E-132	PQLC3
ncbi_101802908	0.896666667	5.47	2.608897161	2.44E-133	1.12E-131	Nrf1
ncbi_101789578	22.78666667	2.053333333	-3.472150141	5.09E-132	2.31E-130	FAM72A
ncbi_101789841	6.993333333	0.503333333	-3.796394223	5.43E-132	2.45E-130	NCAM2
ncbi_101804696	75.78666667	15.38333333	-2.300575907	9.72E-132	4.37E-130	CALD1
ncbi_101801432	14.94666667	70.11333333	2.229865048	4.87E-131	2.15E-129	KERA
ncbi_101804514	83.47333333	11.94	-2.805512546	1.10E-130	4.84E-129	CYTL1
ncbi_101794860	5.386666667	24.58333333	2.190215851	7.62E-129	3.30E-127	Psat1
ncbi_101791751	23.31666667	4.533333333	-2.362717406	8.54E-129	3.68E-127	UHRF1

ncbi_101790276	0.363333333	8.776666667	4.594307481	3.90E-128	1.68E-126	ADAM19
ncbi_101798549	63.13333333	5.953333333	-3.406632345	9.61E-127	4.09E-125	CSRP1
ncbi_101804021	0.726666667	6.596666667	3.182371572	1.19E-125	5.05E-124	TMEM132C
ncbi_101799795	23.09333333	0.883333333	-4.708374665	1.75E-125	7.37E-124	Cyp3a9
ncbi_101801559	8.013333333	0.68	-3.558795839	3.24E-125	1.33E-123	MET
ncbi_101792025	44.03	6.996666667	-2.653747178	8.87E-124	3.61E-122	ACTC1
ncbi_101800417	0.573333333	6.753333333	3.558153704	1.88E-121	7.53E-120	ANKRD6
ncbi_101800028	14.12666667	1.393333333	-3.34180874	5.61E-121	2.24E-119	ARHGEF5
ncbi_101793307	31.13	5.136666667	-2.599401216	6.77E-121	2.69E-119	Filip1l
ncbi_101800097	20.37	3.35	-2.60421298	9.76E-120	3.85E-118	Pawr
ncbi_101800586	1.45	7.05	2.281570357	4.94E-118	1.92E-116	SFMBT1
ncbi_101803651	27.16666667	3.863333333	-2.813919493	2.21E-117	8.57E-116	STARD5
ncbi_101798376	1.213333333	24.47666667	4.334359244	1.21E-115	4.61E-114	Mc5r
ncbi_101801360	25.64333333	5.343333333	-2.262771883	6.83E-115	2.57E-113	MLF1
ncbi_101791864	3.44	34.6	3.330291568	6.80E-114	2.53E-112	lgr5-a
ncbi_101802046	2.62	10.56666667	2.011881623	3.33E-113	1.23E-111	Mid2
ncbi_101794936	4.933333333	27.28	2.467208969	5.25E-112	1.92E-110	PTGFRN
ncbi_101801433	12.42666667	81.80333333	2.718720272	1.39E-111	5.05E-110	Selenop
ncbi_101799131	1.776666667	11.68666667	2.717618558	2.95E-111	1.07E-109	Csgalnact1
ncbi_101790844	29.60333333	5.616666667	-2.397973542	1.32E-110	4.72E-109	Rflnb
ncbi_101800494	21.23	5.023333333	-2.079387455	3.31E-110	1.17E-108	ARAP2
ncbi_101800796	0.713333333	11.82666667	4.051323308	1.67E-109	5.87E-108	nkain4
ncbi_110351750	11.56666667	0.923333333	-3.646977781	5.32E-106	1.82E-104	--
ncbi_101803955	10.89666667	1.033333333	-3.398509256	8.93E-106	3.05E-104	ADRA2A
ncbi_101790670	14.07	0.376666667	-5.223190152	1.49E-105	5.05E-104	CES1

ncbi_101803187	5.493333333	0.456666667	-3.588468444	2.43E-105	8.21E-104	SPHKAP
ncbi_101802080	1.87	8.496666667	2.183858697	2.50E-105	8.43E-104	Kif21a
ncbi_101795900	2.206666667	10.65	2.270912809	3.82E-105	1.28E-103	CBX7
ncbi_101797374	1.873333333	0.066666667	-4.812498225	5.25E-104	1.75E-102	MUC5AC
ncbi_101799364	0.553333333	11.48333333	4.375248836	5.16E-103	1.70E-101	C1QTNF3
ncbi_101799111	10.55333333	58.14333333	2.461915028	1.00E-102	3.29E-101	IGF2
ncbi_101799400	6.16	0.323333333	-4.251836199	1.65E-102	5.41E-101	HTR2A
MSTRG.4892	8.193333333	0.18	-5.508381698	2.00E-102	6.54E-101	--
ncbi_101804028	2.606666667	11.32	2.118595946	4.44E-102	1.44E-100	ccdc85c
ncbi_101803244	42.99333333	4.753333333	-3.177101488	9.73E-101	3.10E-99	Tubb2b
ncbi_101791827	14.73333333	0.406666667	-5.179093317	4.14E-99	1.31E-97	LMCD1
ncbi_101802576	14.02	0.463333333	-4.919292062	5.67E-99	1.79E-97	Iqcj-Schip1
ncbi_101803713	0.376666667	3.103333333	3.042458395	6.00E-98	1.88E-96	Cfh
ncbi_101792399	0.103333333	5.91	5.837780511	3.00E-97	9.39E-96	DLL4
ncbi_101803685	3.696666667	15.11333333	2.031525026	4.47E-97	1.40E-95	IGFBP3
ncbi_101803015	3.26	0.23	-3.825166198	7.67E-97	2.38E-95	EXPH5
ncbi_101791004	24.73666667	2.323333333	-3.412383046	4.05E-96	1.24E-94	Fhl1
ncbi_101804010	32.68666667	0.853333333	-5.259449046	4.93E-96	1.51E-94	CXCL8
ncbi_101796641	0.37	6.98	4.237629861	5.84E-96	1.78E-94	VIL1
ncbi_101794341	0.503333333	6.213333333	3.625781405	4.42E-95	1.34E-93	TDH
ncbi_101804467	5.253333333	1.306666667	-2.007341975	7.61E-94	2.29E-92	SYNE1
ncbi_101800302	52.84666667	9.546666667	-2.468743484	4.63E-93	1.38E-91	PDLIM1
ncbi_101794262	20.09	3.783333333	-2.408747767	1.77E-92	5.25E-91	MYO1B
ncbi_113845671	38.82	7.843333333	-2.307261298	2.28E-92	6.74E-91	CNN2
ncbi_101799556	15.39	2.09	-2.880418384	1.03E-91	3.03E-90	ADAMTS8

ncbi_101803675	10.26666667	0.936666667	-3.454288315	2.03E-91	5.92E-90	EDN3
ncbi_101802225	33.65	6.69	-2.330528389	2.14E-91	6.23E-90	TES
MSTRG.1411	2.51	0.326666667	-2.941796211	2.00E-89	5.71E-88	--
ncbi_101794665	24.20666667	3.543333333	-2.772225333	8.62E-88	2.42E-86	RRM2
ncbi_101800219	5.73	1.12	-2.355036407	2.29E-87	6.40E-86	SOX11
ncbi_101794291	8.073333333	1.3	-2.634652836	1.91E-86	5.24E-85	EPHA5
ncbi_101790006	20.7	3.186666667	-2.699510745	7.37E-86	2.02E-84	MFSD13A
ncbi_101802012	0.49	13.65666667	4.800679833	7.67E-86	2.09E-84	DEPTOR
ncbi_101790269	6.403333333	0.15	-5.415788707	8.56E-86	2.33E-84	CWH43
ncbi_101804741	3.75	16.13666667	2.105380093	1.48E-85	4.02E-84	SLC2A5
ncbi_101799514	15.08666667	2.253333333	-2.743141433	2.06E-85	5.56E-84	Amotl2
ncbi_101805216	10.86666667	1.796666667	-2.596514786	3.81E-85	1.02E-83	ITGA11
ncbi_101796109	1.3	5.993333333	2.204846992	1.21E-84	3.22E-83	COL24A1
ncbi_101800151	3.566666667	0.143333333	-4.637130327	2.55E-84	6.78E-83	Plch2
ncbi_101799848	14.37666667	1.813333333	-2.987013159	8.80E-84	2.32E-82	--
ncbi_101795448	6.613333333	0.123333333	-5.744742945	1.79E-83	4.69E-82	INHBA
ncbi_113842446	10.94333333	1.416666667	-2.949480004	5.72E-83	1.49E-81	--
ncbi_101805178	30.03666667	6.873333333	-2.127642886	7.51E-83	1.95E-81	KPNA2
ncbi_101790291	2.233333333	16.58666667	2.892753485	1.54E-82	3.98E-81	TRIM2
ncbi_101794141	4.486666667	0.566666667	-2.985071758	2.80E-82	7.22E-81	FGD4
ncbi_101802187	31.63666667	6.113333333	-2.371566463	3.21E-82	8.26E-81	Tubb2b
ncbi_101798185	1.593333333	15.72333333	3.302787078	3.96E-82	1.02E-80	RFLNA
ncbi_101798786	11.49666667	2.77	-2.053257747	4.13E-81	1.05E-79	NFATC2
ncbi_101789745	3.05	16.88333333	2.468718621	8.94E-81	2.27E-79	Me3
ncbi_101800857	0.32	5.556666667	4.118075888	1.31E-80	3.33E-79	ADRB2

ncbi_101799332	0.63	5.316666667	3.077098284	1.06E-79	2.67E-78	ASTN2
ncbi_101792211	2.716666667	15.40333333	2.503333125	1.46E-79	3.65E-78	CHAC1
ncbi_101798471	1.11	8.35	2.911216521	1.81E-79	4.52E-78	S1PR1
ncbi_101795859	0.283333333	4.733333333	4.062284278	3.50E-79	8.70E-78	CHRNA7
ncbi_101791912	0.16	10.38333333	6.020053947	2.87E-78	7.10E-77	Egfl6
ncbi_101802488	0.306666667	18.45666667	5.91132685	3.16E-78	7.79E-77	AKR1D1
ncbi_101804057	0.043333333	1.036666667	4.580331052	4.57E-78	1.12E-76	FCGBP
MSTRG.12221	2.296666667	0.486666667	-2.238535614	1.02E-77	2.47E-76	--
ncbi_101802195	2.886666667	0.143333333	-4.33195846	1.26E-77	3.04E-76	NRG3
ncbi_101798487	5.276666667	0.32	-4.043483039	1.68E-77	4.06E-76	SALL4
ncbi_101800165	18.2	2.18	-3.06153841	8.02E-77	1.91E-75	SRPX2
ncbi_101802392	12.85	2.61	-2.299646648	1.52E-76	3.60E-75	Tmem8a
ncbi_101803495	22.44666667	3.72	-2.593126694	6.57E-76	1.55E-74	LOXL3
ncbi_101794710	2.196666667	12.90666667	2.554728582	7.20E-76	1.69E-74	TLL1
ncbi_101793475	38.02	9.06	-2.069175577	1.69E-75	3.96E-74	F3
ncbi_101800269	0.916666667	10.38	3.501265421	3.14E-75	7.30E-74	PTGER4
ncbi_101803200	11.26666667	1.863333333	-2.596103058	5.43E-75	1.26E-73	ANKRD29
ncbi_101798449	39.06	7.93	-2.300299178	5.52E-75	1.28E-73	LRRC42
ncbi_101798550	27.23333333	6.63	-2.038292802	6.45E-75	1.49E-73	HSPH1
ncbi_101803859	12.21333333	0.05	-7.932313192	7.35E-74	1.67E-72	Tmc3
ncbi_101802925	8.06	0.686666667	-3.553098002	9.92E-74	2.26E-72	Lrrc15
ncbi_101795623	2.473333333	0.22	-3.490881257	4.68E-72	1.05E-70	Cend1
ncbi_101803551	3.376666667	0.373333333	-3.177063537	6.44E-72	1.44E-70	PROX1
ncbi_101794923	2.403333333	10.15666667	2.079318335	1.17E-71	2.61E-70	CHRM4
ncbi_101792841	5.213333333	21.51666667	2.045176583	2.67E-71	5.91E-70	Pdcd7

ncbi_101804833	6.043333333	0.376666667	-4.003984247	3.42E-71	7.55E-70	ACPP
ncbi_101793805	3.26	0.166666667	-4.289834465	3.91E-71	8.57E-70	NIPBL
ncbi_101793261	11.13	0.496666667	-4.486031858	5.31E-71	1.16E-69	MPZL2
ncbi_101804616	0.37	7.136666667	4.269653214	2.39E-70	5.17E-69	PHYHD1
ncbi_101804816	6.893333333	1.613333333	-2.095157233	4.90E-70	1.05E-68	CERS6
ncbi_101791667	6.673333333	31.04	2.217649084	8.11E-70	1.74E-68	TPST1
ncbi_101795348	0.203333333	1.67	3.037929456	1.36E-69	2.91E-68	KCTD16
ncbi_101793344	7.346666667	0.373333333	-4.298553587	1.58E-69	3.38E-68	etnppl
ncbi_101798492	0.056666667	3.613333333	5.9946862	1.99E-69	4.24E-68	GABRR2
ncbi_101800582	20.26	3.913333333	-2.372164266	2.29E-69	4.85E-68	TGL2
ncbi_101790685	15.75666667	2.956666667	-2.413918855	1.04E-68	2.19E-67	CCND3
ncbi_101798086	29.44666667	4.526666667	-2.701583353	1.34E-68	2.81E-67	NPM3
ncbi_101795460	0.106666667	3.213333333	4.912889336	7.37E-68	1.53E-66	DPP10
ncbi_101795788	1.4	7.713333333	2.461927631	9.50E-67	1.95E-65	CP
ncbi_101794231	3.376666667	16.85	2.319076918	1.30E-66	2.65E-65	TFPI2
ncbi_101797616	18.97666667	4.203333333	-2.174620824	9.04E-65	1.80E-63	RIPK2
ncbi_101799811	0.026666667	9.54	8.482807957	2.23E-64	4.41E-63	CDH20
ncbi_101795335	1.266666667	6.25	2.302819272	6.64E-64	1.30E-62	Adgra2
ncbi_101800276	2.686666667	0.33	-3.025279408	1.44E-63	2.79E-62	prr5
ncbi_101797555	11.56333333	0.883333333	-3.710455576	5.48E-63	1.06E-61	KLF2
ncbi_101798657	0.516666667	3.263333333	2.659040644	7.16E-63	1.38E-61	NCALD
ncbi_101790005	0.04	10.28333333	8.006092273	1.31E-62	2.51E-61	PROKR1
ncbi_101794826	0.043333333	2.973333333	6.100460182	1.53E-62	2.93E-61	ACVR1C
ncbi_101802919	1.8	10.36	2.524955191	5.93E-62	1.13E-60	--
ncbi_101794528	0.206666667	5.756666667	4.799856057	9.15E-62	1.74E-60	MMP13

ncbi_101800132	0.15	2.846666667	4.246239163	1.17E-61	2.22E-60	RNF152
ncbi_101793140	3.126666667	0.07	-5.48112669	3.24E-61	6.09E-60	ARHGEF28
ncbi_101791104	9.33	1.62	-2.525883268	1.51E-60	2.80E-59	ITGA6
ncbi_101802160	0.126666667	2.646666667	4.385067684	2.88E-60	5.32E-59	Pde1c
ncbi_101792566	6.783333333	0.893333333	-2.924723889	6.14E-60	1.13E-58	Hip1r
ncbi_101799239	22.59666667	5.33	-2.083902532	1.18E-59	2.16E-58	slc2a10
ncbi_101804447	4.64	0.736666667	-2.655040936	2.54E-59	4.64E-58	Plcb2
ncbi_101800984	14.37333333	3.04	-2.241251449	3.26E-59	5.94E-58	Aspg
ncbi_101794935	46.44666667	10.40666667	-2.158067026	3.60E-59	6.55E-58	Mylk
ncbi_101796553	0.61	4.506666667	2.885179598	3.82E-59	6.94E-58	COL21A1
ncbi_110351183	34.76333333	2.103333333	-4.046817014	6.39E-59	1.16E-57	CCL4
ncbi_101803709	1.65	9.42	2.513261035	7.71E-59	1.39E-57	AADAC
ncbi_101789631	23.05333333	3.593333333	-2.681580691	3.86E-57	6.82E-56	ACOT7
ncbi_101801151	2.966666667	0.106666667	-4.797661526	5.52E-57	9.71E-56	ANO1
ncbi_101796737	0.093333333	10.60666667	6.828363198	9.81E-57	1.71E-55	CA8
ncbi_101795180	1.956666667	0.106666667	-4.197216693	1.51E-56	2.62E-55	PCDH10
ncbi_101801277	6.79	36.57	2.429177147	7.82E-56	1.35E-54	C16orf70
ncbi_101793352	1.56	0.19	-3.037474705	8.55E-56	1.47E-54	MAP1LC3C
ncbi_101804887	13.29333333	3.03	-2.13331321	2.95E-55	5.02E-54	CRMP1
ncbi_101794651	4.713333333	0.15	-4.973713308	6.07E-55	1.03E-53	RIMS1
ncbi_101804319	2.49	0.22	-3.500570313	7.53E-55	1.27E-53	CRYBG1
ncbi_101792508	3.083333333	0.14	-4.460992133	1.84E-54	3.09E-53	HTR2C
ncbi_101803765	4.616666667	0.27	-4.095820258	3.71E-54	6.19E-53	SLC6A15
ncbi_101794037	14.89333333	0.106666667	-7.12541347	3.76E-54	6.28E-53	Acod1
ncbi_101801774	0.033333333	1.953333333	5.87282876	4.18E-54	6.96E-53	Adamts20

ncbi_101800939	6.586666667	0.816666667	-3.011729293	5.96E-54	9.91E-53	GRIA2
ncbi_101790513	3.093333333	0.133333333	-4.5360529	8.21E-54	1.36E-52	MYOCD
ncbi_101795601	65.93	2.553333333	-4.690481285	8.98E-54	1.49E-52	Ddah1
ncbi_101799372	7.7	1.863333333	-2.046972663	2.06E-53	3.40E-52	CHSY3
ncbi_101802393	31.73333333	7.693333333	-2.04431835	2.25E-53	3.70E-52	DUSP1
ncbi_101796497	3.55	23.76666667	2.743048646	3.91E-53	6.36E-52	Pnpla2
ncbi_101793984	0.193333333	2.146666667	3.472935883	7.42E-53	1.20E-51	LAYN
ncbi_101799740	8.723333333	0.676666667	-3.688362292	1.13E-52	1.83E-51	CACNG3
ncbi_101804305	31.30666667	7.773333333	-2.00986462	1.18E-52	1.90E-51	RSPO3
ncbi_101799829	15.82	2.87	-2.462626958	1.30E-52	2.10E-51	Lama3
ncbi_101789743	0.173333333	7.656666667	5.465095423	1.44E-52	2.32E-51	CPM
ncbi_101790600	0.106666667	2.866666667	4.74819285	1.51E-52	2.43E-51	Prima1
ncbi_101801610	10.66333333	1.17	-3.188078057	2.18E-52	3.50E-51	fam162b
ncbi_101804721	19.39	0.38	-5.673169575	2.32E-52	3.72E-51	LYZ
ncbi_101800860	4.693333333	0.896666667	-2.387969256	4.41E-52	7.03E-51	SAMD9L
ncbi_101791898	7.793333333	1.923333333	-2.018631706	6.85E-52	1.09E-50	NPR2
ncbi_101799480	21.04666667	2.576666667	-3.030013941	1.34E-51	2.13E-50	UCHL1
ncbi_101798797	3.106666667	0.16	-4.279223644	8.79E-51	1.39E-49	Cds1
ncbi_101789661	3.636666667	0.07	-5.699117964	2.93E-50	4.58E-49	OPN1LW
ncbi_101799465	10.77666667	1.343333333	-3.004021764	3.10E-50	4.84E-49	PCOLCE2
ncbi_101801776	4.15	0.83	-2.321928095	8.41E-50	1.30E-48	FAM84B
ncbi_101800934	3.07	0.343333333	-3.160556819	1.74E-49	2.67E-48	ARHGEF4
ncbi_101796588	5.503333333	0.183333333	-4.907764691	5.32E-49	8.12E-48	slc51a
ncbi_101797402	11.03333333	1.936666667	-2.510221148	5.75E-49	8.77E-48	GYG2
ncbi_101802885	11.12	1.81	-2.619095186	1.08E-48	1.64E-47	FST

ncbi_101790237	4.866666667	0.226666667	-4.424289813	1.22E-48	1.85E-47	--
ncbi_101805182	4.063333333	18.35666667	2.175568483	2.54E-48	3.83E-47	Rasl11b
ncbi_101795145	2.27	0.373333333	-2.604156066	2.83E-48	4.26E-47	DCHS2
ncbi_101793392	5.226666667	21.62333333	2.048625879	1.74E-47	2.60E-46	FABP5
ncbi_101793482	22.86	5.163333333	-2.14645076	1.83E-47	2.73E-46	AGTRAP
ncbi_101791029	33.65	5.086666667	-2.725814043	2.45E-47	3.63E-46	DUSP14
MSTRG.890	2.126666667	0.173333333	-3.616972896	3.70E-47	5.46E-46	--
ncbi_101791596	22.64333333	4.763333333	-2.249042938	7.90E-47	1.16E-45	SLC25A43
ncbi_101795133	29.60666667	5.27	-2.490047204	8.95E-47	1.31E-45	Nedd9
ncbi_101796090	8.25	0.353333333	-4.545292355	1.05E-46	1.53E-45	ALDOB
ncbi_101793285	32.79	2.41	-3.76615085	3.86E-46	5.58E-45	FABP7
ncbi_101792194	10.45666667	0.413333333	-4.660973503	5.17E-46	7.43E-45	PDLIM4
ncbi_101795087	45.53	8.523333333	-2.417327799	5.90E-46	8.46E-45	Pdlim5
ncbi_101805003	8.17	1.723333333	-2.245134299	1.97E-45	2.81E-44	Stk39
ncbi_101797990	4.026666667	0.333333333	-3.59454855	2.88E-45	4.09E-44	HTR2B
ncbi_101804547	0.813333333	3.62	2.15407105	5.11E-45	7.21E-44	SFMBT2
ncbi_101793081	5.173333333	0.67	-2.948861151	7.70E-45	1.08E-43	MBOAT1
ncbi_113845377	4.15	20.28333333	2.289111521	1.11E-44	1.56E-43	CLEC2D
ncbi_101801570	9.673333333	1.52	-2.66994179	1.34E-44	1.87E-43	STMN2
ncbi_101802211	0.563333333	6.48	3.523933067	5.18E-44	7.18E-43	Timd4
ncbi_101792044	2.016666667	0.246666667	-3.031337967	7.70E-44	1.07E-42	SAMD12
ncbi_101803084	8.276666667	1.953333333	-2.083111692	1.66E-43	2.29E-42	NCAM1
ncbi_101794917	2.563333333	0.343333333	-2.900339261	2.11E-43	2.90E-42	FGD5
ncbi_101800101	0.463333333	4.816666667	3.377912705	2.53E-43	3.47E-42	C7
ncbi_101795511	6.643333333	0.236666667	-4.810978875	3.80E-43	5.19E-42	HAPLN1

MSTRG.17367	2.493333333	0.103333333	-4.59269815	4.11E-43	5.60E-42	UBAP2
ncbi_101802009	5.666666667	0.85	-2.736965594	6.02E-43	8.19E-42	CAPRIN2
ncbi_101796425	5.266666667	0.93	-2.501587531	6.59E-43	8.95E-42	Smtn
ncbi_101796048	2.773333333	0.443333333	-2.645157283	1.90E-42	2.56E-41	PATJ
ncbi_101797885	3.47	0.75	-2.209973162	2.24E-42	3.02E-41	ALK
ncbi_106014264	11.72	47.25666667	2.0115453	2.31E-42	3.11E-41	IFITM5
ncbi_101793001	2.893333333	0.13	-4.476149014	5.75E-42	7.70E-41	PCDH20
ncbi_101801641	0.713333333	6.303333333	3.143466662	1.58E-41	2.09E-40	TGFBR2
ncbi_101801126	1.906666667	0.253333333	-2.911943823	1.62E-41	2.14E-40	CABCOCO1
ncbi_101802330	3.013333333	0.34	-3.14775362	3.22E-41	4.23E-40	CHST11
ncbi_101800667	8.416666667	2.036666667	-2.047039103	3.99E-41	5.23E-40	CDH2
ncbi_101793654	0.14	1.923333333	3.780110086	5.72E-41	7.48E-40	KLHL14
ncbi_101801992	5.966666667	0.063333333	-6.557816359	1.03E-40	1.33E-39	SERPINB12
ncbi_101793237	0.626666667	5.183333333	3.048110013	1.48E-40	1.91E-39	HS3ST1
ncbi_101798389	36	6.873333333	-2.388915075	1.56E-40	2.01E-39	SGCG
ncbi_101793782	5.786666667	0.15	-5.269698136	2.39E-40	3.07E-39	MMP9
ncbi_101800229	6.3	1.55	-2.023083613	3.76E-40	4.81E-39	RHOBTB1
ncbi_101798937	1.323333333	0.04	-5.048032696	5.90E-40	7.51E-39	PCDH17
ncbi_101802347	3.58	0.21	-4.091498354	7.99E-40	1.01E-38	EPCAM
ncbi_101790238	6.116666667	0.306666667	-4.318002392	1.53E-39	1.94E-38	Tppp
ncbi_101794613	1.296666667	0.013333333	-6.603626345	2.52E-39	3.16E-38	CFAP54
ncbi_101793936	0.293333333	1.533333333	2.386058432	3.92E-39	4.92E-38	RPGR
ncbi_101794144	15.11666667	3.02	-2.323519595	4.28E-39	5.37E-38	SDC4
ncbi_113843594	0.116666667	3.59	4.943519518	4.33E-39	5.42E-38	PRDM8
ncbi_101803905	0.293333333	4.726666667	4.010210199	7.61E-39	9.46E-38	SLC25A12

ncbi_101797625	3.783333333	0.77	-2.296727541	1.30E-38	1.61E-37	LRRC8B
ncbi_101799672	0.803333333	4.816666667	2.583964441	1.42E-38	1.75E-37	IL20RA
ncbi_101804465	0.593333333	2.563333333	2.111106357	1.48E-38	1.83E-37	cyp26b1
ncbi_106014693	1.74	10.83333333	2.638318006	3.86E-38	4.71E-37	KCTD12
ncbi_101801632	4.133333333	0.54	-2.936274402	4.04E-38	4.92E-37	DCLK2
ncbi_101798283	4.943333333	0.19	-4.701412868	5.18E-38	6.30E-37	ZNF326
ncbi_101798679	0.08	1.273333333	3.992466327	5.42E-38	6.58E-37	FREM1
ncbi_113840195	0.64	2.89	2.174925683	1.12E-37	1.34E-36	--
ncbi_101795189	1.496666667	0.17	-3.138146293	1.62E-37	1.94E-36	CNTNAP2
ncbi_101799803	9.42	1.896666667	-2.312260908	1.99E-37	2.37E-36	BPI
ncbi_101795169	1.98	0.063333333	-4.966391607	2.54E-37	3.02E-36	MYRIP
ncbi_101791893	1.57	0.02	-6.294620749	2.59E-37	3.09E-36	SLC4A11
MSTRG.17368	1.146666667	0.003333333	-8.426264755	2.82E-37	3.34E-36	Ubap2
ncbi_101802614	17.80666667	4.126666667	-2.109368661	3.90E-37	4.62E-36	PRKG1
ncbi_101802040	0.506666667	2.973333333	2.552972386	4.65E-37	5.49E-36	Cntln
ncbi_101802374	7.233333333	1.06	-2.770596372	5.63E-37	6.63E-36	SPTLC3
ncbi_110354688	7.013333333	0.48	-3.868993988	6.99E-37	8.20E-36	Rspo4
ncbi_101793216	2.48	0.24	-3.36923381	2.40E-36	2.79E-35	CDH5
ncbi_101801769	22.90333333	5.32	-2.106059431	2.46E-36	2.86E-35	CDK2AP1
ncbi_101791544	1.5	0.04	-5.22881869	2.82E-36	3.27E-35	CEFIP
ncbi_101803383	0.246666667	6.553333333	4.731594241	2.95E-36	3.42E-35	IGSF10
ncbi_106016586	0.33	4.156666667	3.65488913	3.66E-36	4.24E-35	--
ncbi_101798901	1.503333333	0.113333333	-3.729520782	3.83E-36	4.42E-35	ITGB4
ncbi_101792363	4.38	0.916666667	-2.256461752	4.16E-36	4.81E-35	DIAPH3
ncbi_101793350	4.616666667	0.533333333	-3.113742166	4.26E-36	4.92E-35	Cacna2d1

ncbi_101800038	0.01	1.183333333	6.886712714	4.33E-36	4.99E-35	CACNA1G
ncbi_113843792	0.946666667	14.01666667	3.888142966	5.04E-36	5.81E-35	Cdkn1c
ncbi_101795349	0.613333333	23.77	5.276326733	6.27E-36	7.22E-35	KCNJ8
ncbi_101802559	3.58	0.103333333	-5.114581968	6.66E-36	7.65E-35	RHPN2
ncbi_101801215	2.02	8.4	2.056034035	7.27E-36	8.34E-35	DGAT2
ncbi_101793347	2.5	0.13	-4.265344567	7.64E-36	8.75E-35	Trpa1
ncbi_101799545	0.513333333	3.396666667	2.726151795	1.37E-35	1.56E-34	KCNS3
ncbi_101795141	5.4	1.233333333	-2.130396637	1.78E-35	2.01E-34	EFNB2
ncbi_101800555	0.066666667	1.643333333	4.623515741	2.02E-35	2.28E-34	Kcng3
MSTRG.884	2.203333333	0.17	-3.69608112	2.09E-35	2.35E-34	RTase
ncbi_101803214	2.83	0.033333333	-6.407692649	3.03E-35	3.41E-34	RASSF6
ncbi_101804325	9.953333333	1.466666667	-2.762638737	5.41E-35	6.02E-34	NSG1
ncbi_101794732	7.47	122.84	4.039528364	8.27E-35	9.16E-34	FNDC1
ncbi_101791300	3.423333333	0.176666667	-4.276300012	1.02E-34	1.13E-33	Mcam
ncbi_101801927	4.336666667	1.033333333	-2.069280841	1.25E-34	1.38E-33	GAS2L3
ncbi_101797179	1.08	0.053333333	-4.339850003	2.13E-34	2.34E-33	MUC5B
ncbi_101795251	1.17	0.08	-3.87036472	2.29E-34	2.51E-33	P2RY8
ncbi_101804426	1.16	0.05	-4.5360529	3.46E-34	3.79E-33	SNTG1
ncbi_101797308	4.45	1.06	-2.069741071	5.57E-34	6.09E-33	Spata13
MSTRG.15966	0.446666667	2.853333333	2.675377796	8.27E-34	9.00E-33	ADAMTS19
ncbi_101795486	1.03	6.626666667	2.685639014	9.49E-34	1.03E-32	HPGD
ncbi_101795713	3.05	0.69	-2.144140976	1.57E-33	1.70E-32	KALRN
ncbi_101796917	0.053333333	1.056666667	4.30833903	4.55E-33	4.88E-32	NWD2
ncbi_101802098	3.883333333	0.106666667	-5.18611424	7.64E-33	8.16E-32	RERGL
ncbi_101797816	2.083333333	0.16	-3.702749879	7.87E-33	8.40E-32	Proser2

ncbi_101790972	6.153333333	0.84	-2.872906914	1.43E-32	1.52E-31	TOX
ncbi_101790688	5.693333333	1.03	-2.466629232	1.87E-32	1.97E-31	CLDN5
ncbi_101805390	2.46	12.46333333	2.34095975	1.89E-32	1.99E-31	LRR1
ncbi_101793940	4.983333333	0.943333333	-2.401271526	2.58E-32	2.71E-31	Hcls1
ncbi_101796157	2.996666667	0.506666667	-2.564249792	3.20E-32	3.34E-31	PCSK1
ncbi_101802729	5.146666667	0.746666667	-2.785102115	5.76E-32	5.97E-31	KCNK12
ncbi_101799515	7.263333333	0.513333333	-3.82266394	9.72E-32	1.00E-30	UEVLD
ncbi_101790623	2.193333333	0.18	-3.607056272	1.03E-31	1.07E-30	EML5
ncbi_101802218	0.323333333	3.046666667	3.236137513	1.26E-31	1.29E-30	COL20A1
ncbi_113839804	6.916666667	0.12	-5.84897062	4.23E-31	4.29E-30	Tinagl1
ncbi_101798916	2.876666667	0.2	-3.846326154	5.52E-31	5.59E-30	SLC13A3
MSTRG.1123	3.176666667	0.28	-3.504014981	6.00E-31	6.07E-30	TTMP
ncbi_101793626	3.64	14.87	2.030394292	6.84E-31	6.91E-30	GEM
ncbi_101803855	3.366666667	18.32	2.444026711	8.00E-31	8.07E-30	VPS11
ncbi_101800093	1.95	0.046666667	-5.384937892	8.04E-31	8.11E-30	BRINP3
ncbi_101799158	0.683333333	3.92	2.520192245	8.76E-31	8.82E-30	--
ncbi_101800652	0.88	0.006666667	-7.044394119	1.26E-30	1.26E-29	XKR4
ncbi_101800930	8.543333333	1.683333333	-2.343478186	1.34E-30	1.34E-29	KCNAB1
ncbi_101804573	9.58	1.006666667	-3.250439607	2.54E-30	2.52E-29	DCK
ncbi_101803844	2.09	0.4	-2.385431037	2.91E-30	2.88E-29	AKAP6
ncbi_101801378	0.43	2.223333333	2.370315696	5.39E-30	5.29E-29	MRC1
ncbi_101793811	5.626666667	0.28	-4.328781766	5.85E-30	5.74E-29	PTHLH
ncbi_101795337	3.56	0.313333333	-3.50610708	7.53E-30	7.37E-29	LRRC7
ncbi_101801122	7.013333333	1.366666667	-2.35943889	9.51E-30	9.29E-29	Pim1
ncbi_101804622	5.173333333	1.236666667	-2.064637466	9.92E-30	9.68E-29	MRVI1

ncbi_101795310	0.143333333	2.176666667	3.924674427	1.42E-29	1.38E-28	SLC16A12
ncbi_101800379	5.28	1.12	-2.237039197	1.89E-29	1.83E-28	ALS2CL
ncbi_101793979	1.06	0.056666667	-4.225420114	1.96E-29	1.90E-28	Apc2
ncbi_101803818	2.31	0.25	-3.207892852	2.02E-29	1.95E-28	ENDOD1
ncbi_113843257	6.336666667	40.46333333	2.674819141	2.02E-29	1.95E-28	MARCKS
ncbi_101800620	11.72	0.13	-6.494317136	2.32E-29	2.24E-28	Ccl5
ncbi_101795171	25.18666667	5.393333333	-2.223411094	3.58E-29	3.42E-28	--
ncbi_101795752	2.126666667	0.023333333	-6.510057692	5.20E-29	4.94E-28	Greb1
ncbi_101804309	0.203333333	1.67	3.037929456	6.37E-29	6.03E-28	FSTL4
ncbi_101794246	0.563333333	2.68	2.250172255	7.03E-29	6.64E-28	--
ncbi_101791342	6.146666667	0.31	-4.309464129	7.34E-29	6.94E-28	DUSP5
ncbi_101803929	12.24333333	2.83	-2.113122437	7.63E-29	7.20E-28	ELMOD1
ncbi_101794654	1.506666667	0.223333333	-2.754089772	9.51E-29	8.95E-28	FAM84A
ncbi_101792063	0.25	4.14	4.049630768	1.18E-28	1.10E-27	FAM69C
ncbi_101793775	0.056666667	1.346666667	4.570748642	1.30E-28	1.22E-27	Pah
ncbi_101798429	3.766666667	0.51	-2.884719215	1.38E-28	1.30E-27	EPHA2
ncbi_110351557	1.926666667	0.306666667	-2.651363726	1.75E-28	1.63E-27	Gdf6
ncbi_101801170	1.92	0.373333333	-2.362570079	2.40E-28	2.24E-27	KIF14
ncbi_101790286	3.563333333	0.866666667	-2.039678325	2.43E-28	2.26E-27	BUB1B
ncbi_101804600	8.126666667	1.76	-2.207088291	3.52E-28	3.26E-27	SPINT1
ncbi_106019410	3.276666667	0.023333333	-7.133692684	3.65E-28	3.37E-27	Ctxnd1
ncbi_113843461	10.45333333	0.033333333	-8.292781749	4.15E-28	3.83E-27	--
ncbi_101801727	1.193333333	7.883333333	2.723808691	1.36E-27	1.24E-26	selenbp1-a
ncbi_101791844	0.056666667	1.93	5.089956697	1.80E-27	1.64E-26	Ccdc3
ncbi_101803341	0.57	3.36	2.559427409	2.24E-27	2.03E-26	RUNX2

ncbi_101804711	3.453333333	0.503333333	-2.778403548	3.25E-27	2.93E-26	MME
ncbi_101797897	7.983333333	1.703333333	-2.22863046	8.13E-27	7.29E-26	Adarb1
ncbi_101795544	3.296666667	0.013333333	-7.949826711	9.06E-27	8.10E-26	GABRB3
ncbi_101793483	1.346666667	6.326666667	2.232052794	1.35E-26	1.20E-25	NKD1
ncbi_101791444	6.013333333	1.39	-2.11308005	1.36E-26	1.21E-25	SLA
ncbi_113845381	0.426666667	5.81	3.767356854	1.53E-26	1.36E-25	Clec2e
ncbi_101795773	0.876666667	0.143333333	-2.612654235	1.81E-26	1.60E-25	GPR98
ncbi_101794727	1.356666667	0.253333333	-2.420957471	5.95E-26	5.21E-25	CUBN
ncbi_101791733	0.876666667	0.063333333	-3.790991476	6.43E-26	5.61E-25	TACC2
ncbi_101798262	1.353333333	0.2	-2.758445322	7.87E-26	6.85E-25	TENM1
ncbi_101801967	3.973333333	0.73	-2.444381461	2.59E-25	2.23E-24	SLC2A12
ncbi_101790665	1.663333333	0.006666667	-7.962896005	2.61E-25	2.24E-24	RASSF9
ncbi_101796556	1.133333333	0.103333333	-3.455194626	2.74E-25	2.35E-24	PCDH9
ncbi_101804197	1.593333333	0.04	-5.315904307	2.84E-25	2.44E-24	TOX3
ncbi_101803012	6.93	0.026666667	-8.021674043	4.55E-25	3.88E-24	Edn1
ncbi_101796687	0.553333333	4.34	2.971474302	4.77E-25	4.07E-24	LHX8
ncbi_101790350	1.466666667	0.07	-4.389042291	6.05E-25	5.13E-24	SHROOM3
ncbi_101798722	1.543333333	0.103333333	-3.900672073	7.49E-25	6.33E-24	MMEL1
ncbi_101804482	1.853333333	0.32	-2.533978572	8.54E-25	7.21E-24	Spag6
ncbi_101793351	1.006666667	0.01	-6.653442239	1.75E-24	1.47E-23	GPR26
ncbi_101800579	1.98	0.25	-2.98550043	2.64E-24	2.20E-23	ADORA2B
ncbi_101792124	1.016666667	6.82	2.745924997	5.86E-24	4.85E-23	PKDCC
ncbi_101804812	8.706666667	1.633333333	-2.414301243	6.12E-24	5.06E-23	GOLM1
ncbi_113840909	1.83	11.07	2.596739668	6.90E-24	5.68E-23	COL14A1
ncbi_113841128	43.55333333	0.001	-15.41049552	7.44E-24	6.12E-23	--

ncbi_101802755	4.95	0.253333333	-4.288319702	7.45E-24	6.12E-23	ACTA1
ncbi_101803787	2.656666667	0.036666667	-6.179004295	9.12E-24	7.47E-23	SMAD2
ncbi_101804933	3.666666667	0.75	-2.289506617	1.10E-23	8.94E-23	PALLD
ncbi_101800374	1.05	0.053333333	-4.299208018	1.32E-23	1.07E-22	ANKRD9
ncbi_101795488	3.44	0.596666667	-2.527411478	1.57E-23	1.28E-22	trabd2b
ncbi_101792609	0.046666667	0.963333333	4.36757076	1.91E-23	1.54E-22	GFRA4
ncbi_101796882	1.076666667	0.123333333	-3.125936989	3.90E-23	3.12E-22	EML6
ncbi_101803171	1.266666667	0.05	-4.662965013	5.25E-23	4.18E-22	KCNJ12
ncbi_101797047	0.85	3.573333333	2.071735754	6.67E-23	5.30E-22	BDH1
ncbi_101792582	0.57	0.036666667	-3.958420896	9.60E-23	7.58E-22	Robo2
ncbi_101791538	2.543333333	0.28	-3.183221824	1.09E-22	8.56E-22	Stk32b
ncbi_101801427	2.376666667	0.466666667	-2.348475249	1.29E-22	1.02E-21	KIAA1217
MSTRG.15967	0.326666667	2.54	2.958937343	1.56E-22	1.22E-21	ADAMTS19
ncbi_101803499	1.596666667	0.033333333	-5.581953751	1.73E-22	1.35E-21	RAPGEF4
ncbi_101805156	6.416666667	1.536666667	-2.06201979	1.94E-22	1.51E-21	THY1
ncbi_101799177	1.423333333	0.236666667	-2.58834514	1.99E-22	1.55E-21	EPHA6
ncbi_101790293	6.466666667	1.37	-2.238846353	2.13E-22	1.66E-21	RACGAP1
ncbi_101796681	5.543333333	0.646666667	-3.099659611	2.92E-22	2.27E-21	PKIG
ncbi_101797261	0.143333333	1.226666667	3.097297201	3.65E-22	2.82E-21	PI15
ncbi_101789824	0.133333333	0.993333333	2.897240426	4.03E-22	3.11E-21	CCDC141
ncbi_101793310	17.55	0.006666667	-11.36221782	4.62E-22	3.56E-21	Vcp
ncbi_101804899	0.436666667	6.8	3.960930435	7.80E-22	5.97E-21	MGP
ncbi_101802310	3.373333333	0.723333333	-2.221442342	7.89E-22	6.04E-21	SHTN1
ncbi_101796084	11.99333333	2.293333333	-2.386714717	1.42E-21	1.07E-20	CWC27
ncbi_101791767	0.856666667	0.156666667	-2.451035698	1.85E-21	1.40E-20	RAB11FIP4

ncbi_101797824	1.59	0.36	-2.142957954	1.87E-21	1.42E-20	AFF3
ncbi_101795745	2.386666667	0.45	-2.40700018	2.07E-21	1.56E-20	LNX2
ncbi_101796389	1.133333333	0.076666667	-3.88582898	2.59E-21	1.95E-20	UMODL1
ncbi_101801657	5.403333333	1.173333333	-2.203236757	2.66E-21	1.99E-20	RGS3
MSTRG.6687	3.086666667	0.67	-2.203816692	2.66E-21	1.99E-20	LBH
ncbi_106017781	2.35	0.073333333	-5.002047829	3.40E-21	2.54E-20	Lypd6
ncbi_101802542	2.083333333	0.28	-2.895394957	4.25E-21	3.16E-20	KCNK2
ncbi_101798788	2.08	0.326666667	-2.670692375	5.19E-21	3.85E-20	E2F7
ncbi_101797665	3.596666667	0.4	-3.168588554	7.09E-21	5.23E-20	ACKR2
ncbi_101794855	5.91	1.18	-2.324371271	8.48E-21	6.25E-20	CKAP2
ncbi_101802660	4.086666667	0.356666667	-3.518276277	8.55E-21	6.30E-20	Adgrg2
ncbi_101802278	0.083333333	10.10666667	6.922197848	1.29E-20	9.41E-20	Hebp2
ncbi_101800243	1.7	0.24	-2.824428435	1.63E-20	1.19E-19	MYBL1
ncbi_101796956	1.783333333	0.163333333	-3.448685237	2.22E-20	1.60E-19	tf-b
ncbi_101798064	11.48	1.176666667	-3.286345054	2.27E-20	1.64E-19	FGF2
ncbi_101805318	0.526666667	3.326666667	2.659115257	2.29E-20	1.65E-19	Gpm6b
ncbi_101803184	2.916666667	11.91333333	2.030184712	2.61E-20	1.87E-19	FKBP5
MSTRG.16592	0.706666667	8.506666667	3.589492159	3.06E-20	2.19E-19	--
ncbi_101803634	3.566666667	0.82	-2.120880576	3.48E-20	2.48E-19	INCENP
ncbi_101789953	4.04	0.936666667	-2.108747663	3.60E-20	2.57E-19	Tom1l1
ncbi_101793000	2.226666667	0.18	-3.62881679	3.91E-20	2.78E-19	MAOA
ncbi_101803894	4.286666667	1.033333333	-2.052550522	4.07E-20	2.89E-19	PTPRO
ncbi_101800393	1.106666667	0.233333333	-2.245756414	4.11E-20	2.92E-19	CIT
ncbi_101801280	8.053333333	0.001	-12.97537033	5.11E-20	3.62E-19	GAS2
ncbi_101796428	3.383333333	0.753333333	-2.16708505	6.21E-20	4.39E-19	VAT1L

ncbi_101803640	4.093333333	53.89	3.718669527	7.68E-20	5.39E-19	DERL2
ncbi_106020377	10.39666667	0.001	-13.34383343	1.11E-19	7.76E-19	Rpl37
ncbi_106020614	2.01	0.1	-4.329123596	1.17E-19	8.16E-19	HTR1A
ncbi_101803635	1.403333333	0.236666667	-2.567929304	1.21E-19	8.40E-19	Npas3
ncbi_101803834	4.36	0.81	-2.428334322	1.29E-19	8.98E-19	TMEM40
ncbi_101799600	2.576666667	0.02	-7.009362103	1.65E-19	1.14E-18	GUCY1A1
ncbi_101794469	0.726666667	0.026666667	-4.768184325	1.95E-19	1.34E-18	GCNT7
ncbi_101790524	1.586666667	0.093333333	-4.087462841	2.77E-19	1.89E-18	Mat2a
ncbi_101802093	0.936666667	0.023333333	-5.327071398	3.48E-19	2.37E-18	CDK18
ncbi_101798356	4.186666667	0.386666667	-3.436639754	5.46E-19	3.70E-18	CSRP2
ncbi_101796224	4.593333333	0.001	-12.16532577	5.89E-19	3.98E-18	CLCN4
ncbi_101803065	6.366666667	0.64	-3.314394422	5.90E-19	3.98E-18	RASL11A
ncbi_101794598	1.096666667	0.033333333	-5.040015679	7.37E-19	4.96E-18	Ankrd33b
ncbi_101791742	0.92	0.166666667	-2.464668267	9.69E-19	6.49E-18	CHRM3
ncbi_101803029	2.11	0.243333333	-3.116237131	1.82E-18	1.21E-17	Celsr1
ncbi_101791353	2.786666667	0.62	-2.168200321	1.95E-18	1.30E-17	MCM10
ncbi_113845040	2.69	0.27	-3.31657486	1.95E-18	1.30E-17	SLC38A3
ncbi_101796855	1.046666667	0.136666667	-2.937068744	2.08E-18	1.38E-17	COL19A1
ncbi_113845672	1.746666667	0.19	-3.200532987	2.12E-18	1.41E-17	PALM
ncbi_101798659	3.583333333	0.52	-2.784718726	2.16E-18	1.43E-17	Ica1
ncbi_101799927	1.266666667	0.073333333	-4.11042399	2.60E-18	1.71E-17	SLC27A6
ncbi_101798632	1.236666667	5.09	2.04120897	2.65E-18	1.74E-17	WDFY2
ncbi_101802020	1.993333333	0.076666667	-4.700439718	3.72E-18	2.43E-17	SLC2A6
ncbi_101790213	4.26	0.001	-12.05663772	3.90E-18	2.55E-17	UBE2R2
ncbi_113842275	7.13	0.001	-12.79968636	4.07E-18	2.65E-17	Golph3

ncbi_101802922	2.023333333	0.15	-3.75369961	4.65E-18	3.03E-17	GRIK2
ncbi_101798377	1.633333333	0.02	-6.351675438	4.74E-18	3.08E-17	TRPC4
ncbi_101794222	0.001	6.92	12.75655632	5.02E-18	3.25E-17	STIMATE
ncbi_101794064	12.01666667	2.096666667	-2.518867337	7.24E-18	4.67E-17	PTP4A3
ncbi_101796304	6.7	73.86666667	3.462690475	8.05E-18	5.17E-17	NEGR1
ncbi_101801320	1.666666667	0.026666667	-5.965784285	1.02E-17	6.48E-17	SLITRK6
ncbi_101799901	3.56	0.16	-4.475733431	1.21E-17	7.69E-17	HBEGF
ncbi_101804531	1.206666667	0.196666667	-2.617202838	1.24E-17	7.89E-17	Myo16
ncbi_101803492	0.883333333	0.073333333	-3.590416931	1.26E-17	7.98E-17	Fat4
ncbi_101802676	1.013333333	0.156666667	-2.693338662	1.49E-17	9.40E-17	Slc25a47
ncbi_101803663	2.75	0.426666667	-2.688250309	2.68E-17	1.68E-16	HRASLS
ncbi_101789575	3.883333333	0.196666667	-4.30347119	2.83E-17	1.77E-16	LSMEM1
ncbi_113839925	0.603333333	2.486666667	2.043185933	3.10E-17	1.93E-16	KIAA1324
ncbi_101804569	2.706666667	0.59	-2.197730367	4.74E-17	2.93E-16	LRRC4C
ncbi_101802803	10.37333333	0.013333333	-9.603626345	6.21E-17	3.82E-16	Map3k7cl
ncbi_101790444	1.256666667	0.23	-2.449896256	6.92E-17	4.25E-16	FOLH1
ncbi_101800116	4.133333333	1.006666667	-2.037719666	7.47E-17	4.58E-16	PLK1
ncbi_101792440	1.666666667	0.001	-10.70274988	1.03E-16	6.28E-16	NOX3
ncbi_101797950	3.156666667	0.173333333	-4.186780897	1.12E-16	6.78E-16	Areg
ncbi_101791936	1.296666667	0.163333333	-2.988916501	1.18E-16	7.15E-16	MARVELD2
ncbi_101791863	3.453333333	0.373333333	-3.209453366	1.25E-16	7.57E-16	Cndp1
ncbi_101796808	0.406666667	0.043333333	-3.230297619	1.30E-16	7.87E-16	LRP2
ncbi_106020685	0.001	13.48666667	13.7192462	1.32E-16	7.99E-16	Gtpbp4
ncbi_101804097	3.88	0.543333333	-2.836147189	1.56E-16	9.38E-16	RBM25
ncbi_101802738	1.456666667	0.32	-2.186526969	1.63E-16	9.84E-16	CENPF

ncbi_101799777	1.473333333	0.216666667	-2.765534746	2.00E-16	1.20E-15	MYB
ncbi_101797337	0.143333333	1.613333333	3.492598483	2.43E-16	1.45E-15	PLXND1
ncbi_101800102	2.743333333	0.453333333	-2.597285779	2.51E-16	1.49E-15	KCNV1
ncbi_113842276	5.306666667	0.001	-12.37359021	3.57E-16	2.11E-15	Golph3
ncbi_101792953	1.03	0.136666667	-2.913911023	4.59E-16	2.71E-15	KBTBD3
ncbi_101791200	8.03	1.4	-2.519973161	6.49E-16	3.80E-15	SOCS3
ncbi_101800866	2.776666667	0.11	-4.657778566	9.23E-16	5.37E-15	LUZP2
ncbi_113845585	7.723333333	1.153333333	-2.743414101	9.60E-16	5.58E-15	HSPB1
ncbi_101804661	0.226666667	2.313333333	3.351329011	1.32E-15	7.62E-15	MEOX1
ncbi_101803666	1.493333333	0.06	-4.637429921	1.35E-15	7.84E-15	ITGB3
ncbi_101790692	0.19	1.763333333	3.214233898	1.70E-15	9.79E-15	GUCY1A2
ncbi_101799314	1.663333333	0.37	-2.168480139	2.11E-15	1.22E-14	USP13
ncbi_101791511	1.223333333	0.283333333	-2.110245317	3.77E-15	2.15E-14	Phex
ncbi_101797483	1.21	0.216666667	-2.481457925	3.91E-15	2.22E-14	COBL
ncbi_101791432	2.69	0.033333333	-6.334496768	4.19E-15	2.38E-14	Krt23
ncbi_101804875	2.213333333	0.09	-4.620151929	4.45E-15	2.52E-14	Trim39
MSTRG.17022	1.153333333	0.226666667	-2.347165386	5.04E-15	2.85E-14	gag-pro-pol
ncbi_101799162	0.876666667	0.036666667	-4.579487371	5.32E-15	3.00E-14	COLEC11
ncbi_101790673	2.636666667	0.001	-11.36449948	6.56E-15	3.68E-14	--
ncbi_101796997	1.526666667	0.313333333	-2.284614936	6.92E-15	3.88E-14	GSDME
MSTRG.16558	2.313333333	0.11	-4.394397733	7.12E-15	3.99E-14	SCAMP1
ncbi_101805469	0.813333333	0.063333333	-3.682809824	7.40E-15	4.14E-14	MUC6
ncbi_101797351	3.62	0.893333333	-2.018719197	7.72E-15	4.32E-14	slc12a8
ncbi_101790734	1.833333333	0.001	-10.8402534	9.89E-15	5.51E-14	FAM219A
ncbi_101803805	0.513333333	2.173333333	2.081941614	1.18E-14	6.58E-14	Bmf

ncbi_101800956	0.84	0.076666667	-3.453717967	1.20E-14	6.68E-14	lrmp
ncbi_101797495	0.753333333	0.166666667	-2.176322773	1.48E-14	8.19E-14	PLXNB1
ncbi_101804984	1.083333333	6.036666667	2.478274923	1.50E-14	8.26E-14	BTG1
ncbi_101798663	2.31	0.26	-3.151309323	1.56E-14	8.59E-14	Rhob
ncbi_101804232	1.03	0.01	-6.686500527	1.59E-14	8.78E-14	MYBPC3
ncbi_101793169	0.82	0.08	-3.357552005	1.74E-14	9.58E-14	LAMP3
ncbi_101801371	0.69	0.006666667	-6.693486957	1.79E-14	9.81E-14	PTPRQ
ncbi_101799433	0.13	0.753333333	2.534776744	1.89E-14	1.03E-13	Scn5a
ncbi_101804395	0.001	0.826666667	9.691161905	2.15E-14	1.18E-13	Fgf13
ncbi_101797203	2.283333333	0.326666667	-2.805250334	2.17E-14	1.19E-13	KRT7
ncbi_101802869	0.4	0.01	-5.321928095	2.46E-14	1.34E-13	SHANK2
ncbi_101794252	2.203333333	13.71	2.637468895	3.04E-14	1.65E-13	GRK5
ncbi_101796106	4.94	1.183333333	-2.061654518	3.14E-14	1.71E-13	Ttc9
ncbi_101795383	1.016666667	0.1	-3.345774837	3.46E-14	1.87E-13	IL18R1
ncbi_101796489	0.296666667	0.02	-3.89077093	3.88E-14	2.10E-13	PCLO
ncbi_101802481	0.196666667	1.723333333	3.131377421	4.16E-14	2.25E-13	ALDH1A1
MSTRG.17642	0.993333333	0.196666667	-2.336525471	4.53E-14	2.44E-13	--
ncbi_101794483	1.476666667	0.033333333	-5.469234794	4.57E-14	2.46E-13	--
ncbi_101793067	1.716666667	0.066666667	-4.686500527	4.59E-14	2.47E-13	ANGPTL5
ncbi_101790595	1.063333333	0.16	-2.732450113	4.63E-14	2.49E-13	ERC2
ncbi_101791824	1.006666667	0.04	-4.653442239	5.31E-14	2.85E-13	TMEM71
ncbi_101792940	2.123333333	0.293333333	-2.855717944	5.46E-14	2.92E-13	ZPLD1
ncbi_101801932	1.753333333	0.023333333	-6.231564067	5.59E-14	2.99E-13	SLC19A3
ncbi_101804420	1.41	0.001	-10.46147945	6.53E-14	3.49E-13	CHD1
ncbi_101804775	3.296666667	0.001	-11.6867923	6.58E-14	3.52E-13	Chst5

ncbi_101795426	1.6	0.001	-10.64385619	7.92E-14	4.21E-13	ZNF131
ncbi_101799028	2.436666667	0.37	-2.71931173	8.69E-14	4.60E-13	HMGCLL1
ncbi_101803119	0.001	1.033333333	10.01309	1.01E-13	5.36E-13	RHOU
ncbi_101795208	2.04	0.016666667	-6.935459748	1.03E-13	5.43E-13	STEAP4
ncbi_101799797	0.57	0.08	-2.832890014	1.04E-13	5.46E-13	Fut9
ncbi_101802583	1.673333333	0.3	-2.479690458	1.05E-13	5.53E-13	KNL1
ncbi_101795388	7.246666667	0.666666667	-3.442280035	1.24E-13	6.52E-13	FABP3
ncbi_113844138	2.846666667	0.373333333	-2.930737338	1.25E-13	6.55E-13	--
ncbi_101796085	1.646666667	0.056666667	-4.86090439	1.25E-13	6.55E-13	TNIP3
ncbi_101798321	0.446666667	0.006666667	-6.06608919	1.31E-13	6.88E-13	IL6
ncbi_101793149	2.593333333	0.393333333	-2.720983296	1.44E-13	7.54E-13	BLVRA
ncbi_101795736	0.823333333	3.516666667	2.094660052	1.49E-13	7.81E-13	MRGPRD
ncbi_101797462	13.41	3.13	-2.099074675	1.55E-13	8.09E-13	HNRNPDL
ncbi_101802289	1.253333333	0.03	-5.38466385	1.72E-13	8.95E-13	NUAK2
ncbi_106014818	1.38	7.596666667	2.460698252	1.89E-13	9.81E-13	Lyrm2
ncbi_101798643	0.15	1.066666667	2.830074999	2.25E-13	1.17E-12	Msln
ncbi_113840922	3.593333333	0.033333333	-6.752213368	2.58E-13	1.33E-12	Ubap1
ncbi_101804086	1.736666667	0.293333333	-2.565707944	2.75E-13	1.42E-12	FRRS1
ncbi_101797407	0.5	0.023333333	-4.421463768	2.87E-13	1.47E-12	Myt1l
ncbi_101801621	1.756666667	0.373333333	-2.23430423	3.05E-13	1.57E-12	LRRK1
ncbi_101791823	1.793333333	0.296666667	-2.595728932	3.16E-13	1.62E-12	Tmem117
ncbi_101793250	1.276666667	0.003333333	-8.581200582	3.37E-13	1.72E-12	UBQLN1
ncbi_113839795	0.8	6.316666667	2.981091537	3.57E-13	1.82E-12	TMEM35B
ncbi_101798873	0.35	0.016666667	-4.392317423	3.58E-13	1.83E-12	OPN5
ncbi_101795331	0.62	0.026666667	-4.539158811	3.62E-13	1.84E-12	FLT1

ncbi_101805440	1.86	0.453333333	-2.036658471	3.68E-13	1.87E-12	Nyx
ncbi_113841346	0.001	25.89	14.66010734	3.84E-13	1.96E-12	IPO7
ncbi_101794188	3.16	0.113333333	-4.801280408	4.11E-13	2.09E-12	HECW1
ncbi_101801642	0.87	0.18	-2.273018494	4.16E-13	2.11E-12	CADM2
ncbi_101792096	0.066666667	0.346666667	2.378511623	4.70E-13	2.37E-12	PDZD2
ncbi_101790126	2.453333333	0.52	-2.238159737	5.22E-13	2.63E-12	SARG
ncbi_101798958	1.58	0.2	-2.981852653	5.90E-13	2.97E-12	MAP7
ncbi_113840231	7.936666667	0.533333333	-3.89542381	6.52E-13	3.27E-12	AVD
ncbi_101799190	1.06	0.09	-3.557995453	7.33E-13	3.66E-12	TC2N
ncbi_101792099	1.04	0.086666667	-3.584962501	7.44E-13	3.72E-12	ST8SIA5
ncbi_101790838	5.506666667	0.001	-12.42696357	7.53E-13	3.76E-12	BTF3
ncbi_101801954	1.513333333	0.001	-10.56351408	8.58E-13	4.26E-12	Lama5
ncbi_101797019	0.053333333	0.636666667	3.577428828	9.04E-13	4.49E-12	RD3
ncbi_101797566	4.78	0.383333333	-3.640339258	9.61E-13	4.76E-12	Gsta3
ncbi_101800042	0.076666667	0.843333333	3.459431619	1.09E-12	5.41E-12	IL7R
ncbi_101801463	3.09	0.566666667	-2.447034592	1.14E-12	5.64E-12	DEPDC1
ncbi_101803463	2.85	0.64	-2.154818109	1.17E-12	5.76E-12	CDCA8
ncbi_101798590	0.293333333	2.45	3.062168821	1.28E-12	6.31E-12	csgA
ncbi_101796624	1.816666667	0.12	-3.920187418	1.51E-12	7.40E-12	PRRG4
ncbi_101796779	1.313333333	0.15	-3.130198723	1.64E-12	8.01E-12	C4orf19
ncbi_101799294	1.093333333	0.05	-4.450661409	1.64E-12	8.04E-12	NSUN5
ncbi_113844992	0.686666667	3.14	2.193082722	1.77E-12	8.66E-12	FAM181B
ncbi_101802799	0.706666667	0.036666667	-4.268488836	1.94E-12	9.47E-12	FSTL5
ncbi_101790585	33.98666667	8.156666667	-2.058917272	2.00E-12	9.73E-12	PLA2G4A
ncbi_101802900	0.5	2.42	2.275007047	2.02E-12	9.84E-12	RERG

ncbi_101805096	1.176666667	0.001	-10.20048997	2.08E-12	1.01E-11	Rasa1
ncbi_101802747	0.613333333	0.026666667	-4.523561956	2.12E-12	1.03E-11	ASAP1
ncbi_101797279	0.056666667	2.75	5.600787468	2.20E-12	1.07E-11	TAC1
ncbi_101800921	12.51333333	0.03	-8.704287933	2.23E-12	1.08E-11	CCL4
ncbi_101804161	0.803333333	0.15	-2.42103624	2.24E-12	1.09E-11	SYT13
ncbi_101795221	1.173333333	0.056666667	-4.371968777	2.44E-12	1.18E-11	Fbxo41
ncbi_113844192	0.046666667	1.373333333	4.879145605	2.62E-12	1.27E-11	Ttn
ncbi_101803742	0.473333333	0.036666667	-3.690315501	2.75E-12	1.33E-11	GPR85
ncbi_101798055	1.07	0.18	-2.571541985	2.75E-12	1.33E-11	Tmem132e
MSTRG.17154	2.516666667	0.001	-11.29729843	3.03E-12	1.46E-11	--
ncbi_101797038	1.353333333	0.193333333	-2.807354922	3.56E-12	1.71E-11	Rims2
ncbi_101794591	4.416666667	0.636666667	-2.794347816	3.69E-12	1.77E-11	RHOC
ncbi_106017385	1.4	0.006666667	-7.714245518	3.74E-12	1.79E-11	Arx
ncbi_101792845	1.103333333	0.233333333	-2.24140439	3.94E-12	1.88E-11	SPAG17
ncbi_101790234	0.103333333	1.353333333	3.711139607	4.39E-12	2.09E-11	CHGA
ncbi_101798142	0.816666667	0.076666667	-3.413075983	5.20E-12	2.47E-11	MAP3K19
ncbi_101790135	2.48	0.266666667	-3.217230716	5.43E-12	2.57E-11	PTGS2
ncbi_101802016	1.136666667	0.001	-10.15059352	5.73E-12	2.71E-11	HOMER1
ncbi_101794808	2.936666667	0.033333333	-6.461070114	5.79E-12	2.74E-11	ANXA8
ncbi_101791057	0.903333333	0.176666667	-2.354228587	6.34E-12	2.99E-11	CEP350
ncbi_101789447	4.526666667	0.426666667	-3.407267764	7.57E-12	3.56E-11	RBM38
ncbi_101793417	0.073333333	0.843333333	3.523561956	8.98E-12	4.22E-11	PTPRR
ncbi_101804015	0.04	0.296666667	2.89077093	9.08E-12	4.26E-11	Kndc1
ncbi_101792647	1.34	0.246666667	-2.441598326	9.35E-12	4.38E-11	CAPN3
ncbi_101801046	1.816666667	0.436666667	-2.056689418	9.90E-12	4.64E-11	DSC2

ncbi_101800864	1.753333333	0.36	-2.284031487	1.07E-11	4.99E-11	CEP55
ncbi_101799695	2.21	0.446666667	-2.30677587	1.18E-11	5.50E-11	Rgs14
ncbi_101799102	3.106666667	0.02	-7.279223644	1.21E-11	5.64E-11	ST6GALNAC2
ncbi_101792197	1.923333333	0.001	-10.9093931	1.24E-11	5.76E-11	--
ncbi_101801480	1.033333333	0.001	-10.01309	1.31E-11	6.11E-11	Cables2
ncbi_101798948	0.803333333	0.193333333	-2.054908341	1.36E-11	6.33E-11	ABCA4
ncbi_101789714	0.54	0.016666667	-5.017921908	1.41E-11	6.52E-11	TEK
ncbi_101793958	0.98	0.013333333	-6.199672345	1.66E-11	7.67E-11	KIF6
ncbi_110353991	2.876666667	0.39	-2.88285203	1.75E-11	8.09E-11	HSPB8
ncbi_113840147	0.12	0.726666667	2.598259323	1.82E-11	8.40E-11	FGF10
ncbi_101803726	1.066666667	0.166666667	-2.678071905	1.97E-11	9.05E-11	CNK3/IPCEF1
ncbi_101793144	0.776666667	0.02	-5.279223644	2.12E-11	9.73E-11	ARMC4
ncbi_101794439	0.08	0.523333333	2.709658248	2.18E-11	1.00E-10	BMPR1B
ncbi_101796452	0.766666667	0.001	-9.582455645	2.20E-11	1.01E-10	C18orf25
ncbi_101790054	1.233333333	0.3	-2.039528364	2.25E-11	1.03E-10	Chst9
ncbi_110351193	2.366666667	0.543333333	-2.12294706	2.47E-11	1.13E-10	CXorf21
ncbi_101799734	1.55	0.02	-6.276124405	2.49E-11	1.14E-10	SH2D4B
ncbi_101800736	0.033333333	2.606666667	6.289096702	2.73E-11	1.24E-10	Slitrk4
ncbi_101791456	0.72	0.001	-9.491853096	2.74E-11	1.25E-10	CDCP1
ncbi_101798767	1.586666667	0.006666667	-7.894817763	2.95E-11	1.34E-10	SELP
ncbi_101791380	0.74	0.001	-9.531381461	3.46E-11	1.57E-10	Rasa1
ncbi_101794977	1.02	0.126666667	-3.009460329	3.49E-11	1.58E-10	ST6GALNAC5
ncbi_101790184	0.001	0.46	8.845490051	3.53E-11	1.60E-10	ENPP6
ncbi_101799346	1.63	0.396666667	-2.038872892	3.94E-11	1.78E-10	Foxm1
ncbi_101800094	0.213333333	0.001	-7.736965594	3.96E-11	1.79E-10	CMYA5

ncbi_101794402	0.65	0.04	-4.022367813	4.54E-11	2.04E-10	TINAG
ncbi_101804430	0.773333333	0.001	-9.594946589	4.66E-11	2.09E-10	4-Mar
ncbi_101790484	2.686666667	0.623333333	-2.107741569	4.83E-11	2.17E-10	CDC45
ncbi_110351393	11.35333333	2.673333333	-2.086404293	4.84E-11	2.17E-10	Srgn
ncbi_101793162	1.016666667	0.22	-2.208271313	6.02E-11	2.69E-10	Cpxm2
ncbi_101791807	0.013333333	0.896666667	6.071462363	6.16E-11	2.75E-10	GFRA2
ncbi_101802212	0.5	0.033333333	-3.906890596	6.85E-11	3.04E-10	HS6ST3
ncbi_101792097	1.416666667	0.093333333	-3.923964109	7.32E-11	3.25E-10	ARSD
ncbi_106020332	1.043333333	0.023333333	-5.482663925	7.41E-11	3.28E-10	--
MSTRG.1135	3.46	0.703333333	-2.29849154	7.46E-11	3.30E-10	HHLA2
ncbi_101798456	1.036666667	0.03	-5.110845769	7.52E-11	3.33E-10	SSX2IP
ncbi_101795454	0.786666667	0.001	-9.619608644	7.69E-11	3.40E-10	GPBP1
ncbi_101797156	14.72333333	2.736666667	-2.427612705	9.18E-11	4.04E-10	Dynlt1
ncbi_101798932	1.34	0.016666667	-6.329123596	9.28E-11	4.08E-10	SERPINB10
ncbi_101795272	0.003333333	0.363333333	6.768184325	9.72E-11	4.27E-10	RAB3C
ncbi_101795963	0.07	1.52	4.440572591	1.05E-10	4.62E-10	IL22RA2
ncbi_113843041	1.543333333	0.113333333	-3.767405542	1.07E-10	4.69E-10	--
ncbi_101799222	0.576666667	0.013333333	-5.434628228	1.28E-10	5.58E-10	CFAP43
ncbi_113839807	0.163333333	0.846666667	2.373974843	1.29E-10	5.64E-10	Foxo3
ncbi_101797874	1.886666667	0.426666667	-2.144658243	1.37E-10	5.96E-10	GREB1L
ncbi_101797356	3.053333333	0.28	-3.446886365	1.38E-10	6.00E-10	DHRS9
ncbi_101797344	1.643333333	0.376666667	-2.125264874	1.47E-10	6.37E-10	PARP8
ncbi_101799069	1.19	0.066666667	-4.157852169	1.50E-10	6.48E-10	Sfrp4
ncbi_101799544	1.523333333	0.1	-3.929159759	2.03E-10	8.72E-10	snai1
ncbi_101801785	10.51666667	2.38	-2.143644026	2.29E-10	9.77E-10	TARDBP

ncbi_110354660	0.396666667	0.001	-8.631783357	2.55E-10	1.09E-09	--
ncbi_106019552	2.266666667	0.433333333	-2.387023123	2.67E-10	1.14E-09	B3GALNT1
ncbi_101797738	1.05	0.136666667	-2.941656014	2.82E-10	1.20E-09	ZSWIM6
ncbi_101802761	0.14	0.026666667	-2.392317423	3.10E-10	1.31E-09	Obscn
ncbi_101790274	2.123333333	0.336666667	-2.65693808	3.41E-10	1.44E-09	Tmem51
ncbi_101804508	2.06	0.326666667	-2.656753184	3.52E-10	1.49E-09	AKR1B10
ncbi_101800938	4.683333333	0.001	-12.19332001	3.57E-10	1.51E-09	CCL4
ncbi_101803503	0.73	0.023333333	-4.967432138	3.74E-10	1.58E-09	DLL1
ncbi_101791523	3.43	0.296666667	-3.531293836	3.81E-10	1.61E-09	--
ncbi_101800405	0.786666667	0.001	-9.619608644	4.22E-10	1.77E-09	Dnajb5
ncbi_101795858	0.54	0.06	-3.169925001	4.69E-10	1.97E-09	NTN4
ncbi_101802913	1.41	0.153333333	-3.200951897	4.71E-10	1.97E-09	GABRD
ncbi_101798953	0.443333333	0.066666667	-2.733354341	4.73E-10	1.98E-09	RASGEF1C
ncbi_101793824	1.616666667	0.316666667	-2.351985329	4.75E-10	1.99E-09	Gm1673
ncbi_101802167	1.79	0.093333333	-4.261423356	4.95E-10	2.07E-09	ATP6V0D2
ncbi_101805329	1.34	0.001	-10.38801729	4.97E-10	2.07E-09	KCMF1
ncbi_101800041	1.123333333	0.17	-2.724179439	5.17E-10	2.15E-09	MDH1
ncbi_101797966	0.716666667	0.013333333	-5.74819285	5.17E-10	2.16E-09	Rapgef5
ncbi_101797613	0.463333333	0.043333333	-3.418501355	5.24E-10	2.18E-09	Col4a3
ncbi_101800435	2.83	0.606666667	-2.221826103	5.32E-10	2.22E-09	C1qtnf4
ncbi_101791320	1.043333333	0.16	-2.705056346	5.53E-10	2.30E-09	Kif20b
ncbi_101796773	0.746666667	3.006666667	2.009628701	5.57E-10	2.31E-09	ARRDC2
ncbi_101801352	0.1	0.48	2.263034406	5.66E-10	2.35E-09	CNR1
ncbi_101802065	0.003333333	0.676666667	7.665335917	6.34E-10	2.62E-09	ACE
ncbi_101793615	1.16	0.24	-2.273018494	6.58E-10	2.72E-09	C3

ncbi_101803704	1.563333333	0.31	-2.334285301	6.59E-10	2.72E-09	ERCC6L
ncbi_101791799	0.17	0.003333333	-5.672425342	7.08E-10	2.92E-09	Eppk1
ncbi_101797804	1.003333333	0.126666667	-2.985692163	7.13E-10	2.93E-09	PRDM1
ncbi_101800674	0.01	0.7	6.129283017	7.57E-10	3.11E-09	PLCXD3
ncbi_101794788	0.153333333	0.69	2.169925001	8.60E-10	3.52E-09	PTPRU
ncbi_101794111	1.553333333	0.196666667	-2.981543095	9.08E-10	3.71E-09	CSF2RA
ncbi_113842385	1.02	0.05	-4.350497247	9.72E-10	3.97E-09	--
ncbi_101801160	0.513333333	0.063333333	-3.018859027	1.06E-09	4.32E-09	PPBP
ncbi_101797746	0.596666667	0.001	-9.220781371	1.09E-09	4.44E-09	mtnr1aa
ncbi_101789534	0.446666667	0.033333333	-3.744161096	1.21E-09	4.89E-09	CDHR5
ncbi_101802740	0.893333333	0.03	-4.896164189	1.24E-09	5.03E-09	DDB_G0282555
ncbi_101803766	2.223333333	0.373333333	-2.574188029	1.31E-09	5.30E-09	ERI2
ncbi_101800168	0.946666667	0.2	-2.242856524	1.36E-09	5.49E-09	SLIT2
ncbi_101794326	1.036666667	0.21	-2.303490847	1.46E-09	5.86E-09	Homer2
ncbi_101802124	0.016666667	0.263333333	3.981852653	1.51E-09	6.07E-09	GPR12
ncbi_113841593	2.573333333	0.346666667	-2.892017319	1.73E-09	6.95E-09	PQLC1
ncbi_101791569	9.893333333	1.436666667	-2.783731318	1.86E-09	7.46E-09	CCL3
ncbi_101790561	0.326666667	0.046666667	-2.807354922	1.95E-09	7.82E-09	CLIC5
ncbi_106018967	0.283333333	0.01	-4.824428435	2.22E-09	8.88E-09	--
ncbi_101795741	0.006666667	1.123333333	7.396604781	2.30E-09	9.19E-09	PLIN1
ncbi_101790564	1.513333333	0.026666667	-5.826548487	2.87E-09	1.14E-08	MLKL
ncbi_101791286	1.253333333	0.001	-10.29155445	3.12E-09	1.23E-08	RWDD2B
ncbi_101803986	0.046666667	1.58	5.081388327	3.28E-09	1.29E-08	CFAP97D1
ncbi_101797512	0.776666667	0.09	-3.109298642	3.37E-09	1.33E-08	Pof1b
ncbi_101793899	4.253333333	1.01	-2.07423863	3.52E-09	1.39E-08	NFATC1

ncbi_101794857	1.44	0.243333333	-2.565062943	3.53E-09	1.39E-08	SLC46A2
ncbi_101805024	1.06	0.253333333	-2.064955442	3.55E-09	1.40E-08	Snap91
ncbi_101800588	0.856666667	0.053333333	-4.005624549	3.64E-09	1.43E-08	Gpr132
ncbi_101802261	0.52	0.003333333	-7.285402219	4.08E-09	1.60E-08	SLC19A3
ncbi_101802276	1.47	0.326666667	-2.169925001	4.19E-09	1.64E-08	GATA6
ncbi_101805048	0.2	0.016666667	-3.584962501	4.23E-09	1.66E-08	FAT2
ncbi_106016370	2.956666667	0.673333333	-2.134578812	4.70E-09	1.84E-08	Klhdc8b
ncbi_101793221	0.106666667	0.596666667	2.483815777	4.86E-09	1.90E-08	Whrn
ncbi_101803823	1.913333333	0.27	-2.825056924	5.05E-09	1.97E-08	GABRP
ncbi_101792921	0.283333333	0.046666667	-2.602036014	5.53E-09	2.15E-08	DNAH12
ncbi_101799577	2.28	0.456666667	-2.319820432	6.27E-09	2.43E-08	SHISAL1
ncbi_101805460	1.126666667	0.143333333	-2.974614682	6.63E-09	2.57E-08	Frmd4a
ncbi_101789917	0.286666667	0.02	-3.841302254	6.75E-09	2.61E-08	VGLL1
ncbi_110354509	8.693333333	2.113333333	-2.040389124	7.99E-09	3.07E-08	PDLIM7
ncbi_106017129	1.473333333	0.146666667	-3.328470941	7.99E-09	3.07E-08	TIRAP
ncbi_101793697	0.48	0.09	-2.415037499	8.10E-09	3.11E-08	GPR158
ncbi_101800635	1.563333333	0.313333333	-2.318855261	8.25E-09	3.17E-08	Sema4a
ncbi_101794581	0.696666667	0.023333333	-4.90000421	8.60E-09	3.30E-08	RIBC2
ncbi_101795572	0.94	0.103333333	-3.185355042	8.65E-09	3.32E-08	IL1RL1
MSTRG.17434	1.793333333	0.256666667	-2.804675822	8.78E-09	3.36E-08	zfr
ncbi_101790749	1.5	0.16	-3.22881869	9.13E-09	3.50E-08	Cnrip1
ncbi_101793178	0.836666667	0.001	-9.708509148	1.02E-08	3.88E-08	MIER3
ncbi_101803419	0.6	0.001	-9.22881869	1.11E-08	4.23E-08	SLC39A12
ncbi_101800738	0.99	0.196666667	-2.331676071	1.12E-08	4.26E-08	KIF5C
ncbi_101790923	4.663333333	1.073333333	-2.119263369	1.16E-08	4.42E-08	TBXA2R

ncbi_113840727	2.04	0.416666667	-2.291603558	1.17E-08	4.45E-08	SKA3
ncbi_101799805	0.94	0.086666667	-3.439111634	1.18E-08	4.47E-08	TLL2
ncbi_101794809	1.633333333	0.4	-2.029747343	1.33E-08	5.02E-08	MCOLN3
ncbi_101794409	0.79	0.103333333	-2.934546939	1.37E-08	5.18E-08	CERS3
ncbi_101791039	1.43	0.233333333	-2.615550821	1.38E-08	5.19E-08	slc22a16
ncbi_101798416	1.486666667	0.07	-4.408582477	1.44E-08	5.42E-08	ANGPT2
ncbi_101789538	0.62	0.033333333	-4.217230716	1.46E-08	5.49E-08	ESYT3
ncbi_101798515	1.003333333	0.173333333	-2.533179959	1.62E-08	6.07E-08	KIF18A
ncbi_106014252	0.001	0.636666667	9.314394422	1.96E-08	7.30E-08	OR51G2
ncbi_101791292	1.066666667	0.013333333	-6.321928095	1.98E-08	7.37E-08	TGM3
ncbi_101791678	0.703333333	0.003333333	-7.721099189	2.03E-08	7.56E-08	Il12rb2
ncbi_101789692	1.06	0.006666667	-7.312882955	2.16E-08	8.03E-08	TLR6
ncbi_113844342	0.086666667	1.49	4.103691303	2.48E-08	9.18E-08	C4bpa
ncbi_113844025	0.106666667	0.9	3.076815597	2.65E-08	9.79E-08	Ina
ncbi_101798732	1.676666667	0.263333333	-2.670633842	2.77E-08	1.02E-07	BLNK
ncbi_101798302	0.06	0.626666667	3.38466385	2.81E-08	1.04E-07	BMP3
ncbi_101805421	0.083333333	0.87	3.384049807	2.96E-08	1.09E-07	CTGF
ncbi_101790481	1.116666667	0.136666667	-3.030465281	3.08E-08	1.13E-07	FGF19
ncbi_101803209	0.206666667	0.013333333	-3.95419631	3.14E-08	1.15E-07	CACNA1H
ncbi_113840907	0.38	0.001	-8.569855608	3.32E-08	1.22E-07	Zswim6
ncbi_113842230	0.356666667	0.001	-8.478432581	3.39E-08	1.24E-07	--
ncbi_101796230	0.58	0.003333333	-7.442943496	3.41E-08	1.25E-07	CDH8
ncbi_106014253	0.663333333	0.056666667	-3.549161779	3.68E-08	1.35E-07	MYCT1
ncbi_101797743	0.68	0.001	-9.409390936	3.80E-08	1.39E-07	KIF2A
ncbi_101800865	0.836666667	0.05	-4.064652958	4.20E-08	1.53E-07	WNT2B

ncbi_101789615	26.36333333	4.65	-2.503230172	4.35E-08	1.58E-07	GMFB
ncbi_101805041	0.923333333	0.001	-9.85070776	4.67E-08	1.70E-07	TUBA4A
ncbi_101793031	0.483333333	0.036666667	-3.720477471	4.70E-08	1.70E-07	AHR
ncbi_101790863	0.33	0.033333333	-3.307428525	4.84E-08	1.75E-07	Pde6b
ncbi_101795412	0.48	0.096666667	-2.311944006	4.86E-08	1.76E-07	HORMAD2
ncbi_101799219	1.84	0.173333333	-3.408084739	5.13E-08	1.85E-07	SLC22A4
ncbi_101792808	0.94	0.026666667	-5.139551352	5.18E-08	1.87E-07	MAOB
ncbi_101801016	0.636666667	3.276666667	2.363618778	5.19E-08	1.87E-07	LYPD1
ncbi_101790373	0.001	0.44	8.781359714	5.43E-08	1.96E-07	ACP2
ncbi_101805245	0.316666667	0.001	-8.306821202	5.89E-08	2.12E-07	WNT7B
ncbi_101799655	0.183333333	0.006666667	-4.781359714	6.12E-08	2.20E-07	SLC9A4
ncbi_101790940	0.87	0.1	-3.121015401	6.28E-08	2.26E-07	Spns3
ncbi_101800228	0.02	0.413333333	4.36923381	6.33E-08	2.27E-07	--
ncbi_101793589	0.39	0.073333333	-2.410933101	6.42E-08	2.30E-07	COL25A1
ncbi_101795452	3.623333333	0.763333333	-2.246932437	6.61E-08	2.37E-07	ASF1
ncbi_101800292	0.016666667	0.353333333	4.40599236	6.77E-08	2.42E-07	C6
ncbi_101798959	1.13	0.07	-4.01282404	7.60E-08	2.71E-07	Krt75
ncbi_101805198	1.273333333	0.001	-10.31439442	7.79E-08	2.77E-07	--
ncbi_113844326	13.75333333	3.07	-2.16347076	8.35E-08	2.97E-07	CRIP1
ncbi_101797917	0.07	0.286666667	2.033947332	8.38E-08	2.98E-07	UNC5C
ncbi_101799279	0.62	0.093333333	-2.731803889	8.41E-08	2.99E-07	PAQR9
ncbi_110352107	1.06	0.216666667	-2.290515142	8.43E-08	2.99E-07	SMAD7
ncbi_106016448	0.513333333	0.11	-2.222392421	8.99E-08	3.18E-07	GJA8
ncbi_101795934	0.016666667	0.276666667	4.053111336	1.05E-07	3.71E-07	KIAA1024
ncbi_106020385	1.103333333	0.001	-10.107653	1.12E-07	3.94E-07	Stoml2

ncbi_113840033	0.043333333	0.78	4.169925001	1.23E-07	4.33E-07	--
ncbi_101801979	7.89	1.66	-2.248842059	1.33E-07	4.66E-07	PREX2
ncbi_113840919	4.386666667	1.066666667	-2.040015679	1.34E-07	4.68E-07	loxl3b
ncbi_101789620	0.036666667	0.31	3.079727192	1.38E-07	4.81E-07	--
ncbi_101796494	20.99	0.576666667	-5.185820724	1.38E-07	4.82E-07	--
ncbi_101792067	1.753333333	0.25	-2.810100299	1.39E-07	4.84E-07	slc25a30
ncbi_101801924	0.54	0.026666667	-4.339850003	1.40E-07	4.87E-07	GATA2
ncbi_101800019	0.693333333	0.02	-5.115477217	1.42E-07	4.95E-07	Adra1b
ncbi_101798073	0.243333333	0.001	-7.926790153	1.47E-07	5.13E-07	C4
ncbi_101790660	0.11	0.976666667	3.150362735	1.55E-07	5.39E-07	TFCP2L1
ncbi_101794693	3.616666667	0.836666667	-2.111935773	1.59E-07	5.54E-07	DTNBP1
ncbi_101797173	0.13	0.006666667	-4.285402219	1.64E-07	5.68E-07	DNAH5
ncbi_101800548	0.916666667	0.05	-4.196397213	1.88E-07	6.47E-07	CBLN2
ncbi_101805222	0.19	1.323333333	2.800105183	1.90E-07	6.56E-07	--
ncbi_113845478	1.7	0.163333333	-3.379643593	1.92E-07	6.61E-07	PRRT1B
ncbi_101803226	0.01	0.95	6.569855608	1.95E-07	6.73E-07	CTLA4
ncbi_101804934	0.493333333	0.113333333	-2.121990524	2.01E-07	6.90E-07	Gabrb2
ncbi_101801168	1.073333333	0.073333333	-3.871485259	2.03E-07	6.99E-07	Fah
ncbi_101798538	0.346666667	0.003333333	-6.700439718	2.04E-07	7.00E-07	AQP4
ncbi_101789623	1.413333333	0.001	-10.46488605	2.07E-07	7.12E-07	Dctn3
ncbi_113841585	1.413333333	0.001	-10.46488605	2.07E-07	7.12E-07	Dctn3
ncbi_113845717	0.001	1.086666667	10.08569375	2.13E-07	7.31E-07	TFPI2
ncbi_101794691	0.413333333	0.043333333	-3.253756592	2.18E-07	7.47E-07	BICD1
ncbi_101799998	0.586666667	0.126666667	-2.211504105	2.24E-07	7.66E-07	CENPE
ncbi_101801117	3.553333333	0.78	-2.187627003	2.41E-07	8.23E-07	MRPS6

ncbi_101790752	1.183333333	0.14	-3.079357792	2.61E-07	8.92E-07	RND2
ncbi_106020373	2.363333333	0.001	-11.20660741	2.69E-07	9.17E-07	IER3IP1
ncbi_101804717	1.63	0.34	-2.261265313	2.76E-07	9.39E-07	Sytl2
ncbi_101804825	3.586666667	0.86	-2.060235107	2.76E-07	9.40E-07	CCNB3
ncbi_101802174	0.146666667	0.001	-7.196397213	2.79E-07	9.52E-07	SCN2A
ncbi_101800366	2.123333333	0.393333333	-2.432506513	2.81E-07	9.58E-07	TENT5A
ncbi_101797759	0.456666667	0.04	-3.513069582	2.98E-07	1.01E-06	--
ncbi_101797275	0.09	0.001	-6.491853096	3.21E-07	1.09E-06	CSMD3
ncbi_101793073	1.78	0.046666667	-5.25334101	3.38E-07	1.14E-06	HSPB7
ncbi_101798589	0.446666667	0.096666667	-2.208108195	3.39E-07	1.15E-06	MYO5C
ncbi_101805051	7.083333333	1.586666667	-2.158429363	3.55E-07	1.20E-06	CHCHD7
ncbi_101793720	0.763333333	0.08	-3.254241287	3.73E-07	1.26E-06	BDNF
ncbi_101801324	0.98	0.216666667	-2.177304532	3.91E-07	1.32E-06	ccdc169
ncbi_101797791	0.32	0.026666667	-3.584962501	3.99E-07	1.34E-06	Cps1
ncbi_101793736	0.676666667	0.036666667	-4.205904299	4.02E-07	1.35E-06	SV2A
ncbi_101803806	0.766666667	0.113333333	-2.75802721	4.21E-07	1.41E-06	USP18
ncbi_101795667	1.453333333	0.273333333	-2.41063232	4.50E-07	1.50E-06	COL9A2
ncbi_101802955	0.8	0.001	-9.64385619	4.52E-07	1.51E-06	Stmn4
ncbi_101794537	0.15	0.893333333	2.574236094	4.94E-07	1.65E-06	FGL1
ncbi_101805146	1.833333333	0.42	-2.126007885	5.12E-07	1.70E-06	CCDC181
ncbi_101801955	1.483333333	0.233333333	-2.668378509	5.14E-07	1.71E-06	LIN7A
ncbi_101802686	0.06	0.766666667	3.67556505	5.22E-07	1.73E-06	ADTRP
ncbi_101798346	0.5	0.001	-8.965784285	5.35E-07	1.78E-06	Creb3l3
ncbi_113839776	0.14	0.716666667	2.355875427	5.41E-07	1.80E-06	PTPRU
ncbi_101797884	0.113333333	0.001	-6.824428435	5.50E-07	1.83E-06	Cnksr2

ncbi_101799710	0.85	0.001	-9.731319031	5.71E-07	1.89E-06	Tesk1
ncbi_101801575	0.293333333	1.583333333	2.432352085	5.80E-07	1.92E-06	Ccdc85a
ncbi_101799340	0.64	0.09	-2.830074999	5.91E-07	1.96E-06	Psd2
ncbi_101795080	0.003333333	0.49	7.199672345	6.01E-07	1.99E-06	OR51E2
ncbi_101796880	1	0.056666667	-4.141355849	6.22E-07	2.05E-06	PLCD3
ncbi_101791483	0.546666667	0.076666667	-2.833990049	6.51E-07	2.14E-06	GRM4
ncbi_101804007	0.263333333	0.02	-3.718818247	6.68E-07	2.20E-06	ASCL4
ncbi_101804361	0.54	0.006666667	-6.339850003	7.15E-07	2.35E-06	FRMD3
ncbi_101796159	0.813333333	0.093333333	-3.123382416	7.28E-07	2.39E-06	P2RY1
ncbi_101791570	0.47	0.05	-3.232660757	8.23E-07	2.68E-06	LAMA1
ncbi_101794819	0.783333333	0.126666667	-2.628589433	8.98E-07	2.92E-06	STAT4
MSTRG.17435	0.873333333	0.001	-9.770388596	9.16E-07	2.98E-06	zfr
ncbi_101803817	2.21	0.001	-11.10983065	9.32E-07	3.03E-06	CXCL8
ncbi_101801618	0.3	0.006666667	-5.491853096	9.85E-07	3.19E-06	LAMC2
ncbi_101793780	0.446666667	0.056666667	-2.978626349	1.01E-06	3.27E-06	TPH2
ncbi_101803307	0.056666667	0.286666667	2.338801913	1.02E-06	3.29E-06	CACNA1D
ncbi_101794817	1.59	0.303333333	-2.390050816	1.19E-06	3.82E-06	UGT8
ncbi_101791450	0.346666667	0.03	-3.530514717	1.23E-06	3.95E-06	CCDC180
ncbi_101798678	0.536666667	0.07	-2.938599455	1.34E-06	4.27E-06	NLGN4X
ncbi_101802722	0.106666667	0.433333333	2.022367813	1.35E-06	4.33E-06	CDH4
ncbi_110353807	0.13	0.001	-7.022367813	1.37E-06	4.37E-06	ZNF831
MSTRG.16749	0.316666667	0.003333333	-6.569855608	1.39E-06	4.43E-06	--
ncbi_101792548	0.4	0.043333333	-3.206450877	1.44E-06	4.58E-06	Sorbs2
ncbi_101799473	0.28	0.053333333	-2.392317423	1.53E-06	4.86E-06	SLC24A2
ncbi_101793408	0.163333333	0.001	-7.351675438	1.63E-06	5.17E-06	VTG2

ncbi_101803961	0.513333333	0.01	-5.68182404	1.64E-06	5.19E-06	Mlxipl
ncbi_101803410	0.336666667	50.43333333	7.226912884	1.70E-06	5.38E-06	COL26A1
ncbi_113840914	0.826666667	0.086666667	-3.253756592	1.72E-06	5.45E-06	EML6
ncbi_101790354	1.196666667	0.286666667	-2.061575279	1.73E-06	5.49E-06	VIPR1
ncbi_101802438	0.5	0.05	-3.321928095	1.78E-06	5.64E-06	GDNF
ncbi_113840301	2.43	0.16	-3.924812504	1.82E-06	5.74E-06	GLRX
ncbi_101801925	0.413333333	0.013333333	-4.95419631	1.82E-06	5.74E-06	Cd96
ncbi_101793059	0.533333333	0.006666667	-6.321928095	1.83E-06	5.77E-06	LIX1
ncbi_101802715	3.913333333	0.743333333	-2.396316793	1.95E-06	6.14E-06	IL18
ncbi_101798824	0.001	0.49	8.936637939	2.18E-06	6.85E-06	OIH
MSTRG.16559	0.573333333	0.016666667	-5.10433666	2.49E-06	7.81E-06	--
ncbi_113839922	1.083333333	0.116666667	-3.215012891	2.58E-06	8.07E-06	CD300E
ncbi_101801915	0.33	0.023333333	-3.822001698	2.58E-06	8.08E-06	TMPRSS15
ncbi_101798784	0.77	0.156666667	-2.29716019	2.69E-06	8.40E-06	--
ncbi_101789855	0.096666667	0.576666667	2.576647233	2.70E-06	8.43E-06	Gpd1
ncbi_101802271	1.76	0.423333333	-2.055709433	2.71E-06	8.45E-06	EPHX2
ncbi_101802445	1.33	0.066666667	-4.318316841	2.98E-06	9.26E-06	cmbl
ncbi_106017553	0.38	0.013333333	-4.832890014	3.04E-06	9.45E-06	Map9
ncbi_101796036	0.413333333	0.003333333	-6.95419631	3.10E-06	9.62E-06	SLC13A4
ncbi_101799262	0.526666667	0.053333333	-3.303780748	3.10E-06	9.62E-06	GNAZ
ncbi_101794388	0.34	0.03	-3.502500341	3.16E-06	9.79E-06	LIPC
ncbi_101791922	0.853333333	0.103333333	-3.04580369	3.47E-06	1.07E-05	Btn1a1
ncbi_113842608	0.263333333	0.013333333	-4.303780748	3.66E-06	1.13E-05	Tmie
ncbi_101797166	0.333333333	0.01	-5.058893689	3.69E-06	1.14E-05	PRAG1
ncbi_101796307	1.863333333	0.32	-2.541741972	3.76E-06	1.16E-05	SRGAP1

ncbi_101789957	0.001	0.56	9.129283017	4.00E-06	1.23E-05	Pcdh19
ncbi_101796665	0.396666667	0.033333333	-3.572889668	4.02E-06	1.24E-05	DCSTAMP
ncbi_101792725	0.363333333	0.003333333	-6.768184325	4.25E-06	1.30E-05	Glp1r
ncbi_106020238	0.463333333	0.016666667	-4.797012978	4.27E-06	1.31E-05	PSAP
ncbi_101804337	1.016666667	0.086666667	-3.552225714	4.32E-06	1.32E-05	STK35
ncbi_101803548	0.023333333	0.39	4.063009798	4.41E-06	1.35E-05	Tnni1
ncbi_101797775	1.136666667	0.21	-2.436348006	4.63E-06	1.41E-05	CENPN
ncbi_101797580	0.33	0.016666667	-4.307428525	4.75E-06	1.45E-05	AFP
ncbi_101795639	0.683333333	0.073333333	-3.220048481	4.94E-06	1.51E-05	WNT16
ncbi_101798211	1.346666667	0.323333333	-2.058298641	5.01E-06	1.53E-05	--
ncbi_101800871	0.66	0.03	-4.459431619	5.34E-06	1.62E-05	Dmc1
ncbi_101805104	0.81	0.033333333	-4.602884409	5.65E-06	1.71E-05	CPA2
ncbi_106020314	0.61	0.063333333	-3.267772325	5.67E-06	1.72E-05	GVQW3
ncbi_101794407	0.53	2.8	2.401362562	5.89E-06	1.78E-05	Gngt1
ncbi_101789906	0.24	0.013333333	-4.169925001	5.91E-06	1.79E-05	ATP10B
ncbi_101798541	3.913333333	0.86	-2.185989438	6.24E-06	1.88E-05	SAP30BP
ncbi_101792805	0.576666667	0.09	-2.679740725	6.26E-06	1.89E-05	NGF
ncbi_113844954	0.001	0.946666667	9.886712714	6.33E-06	1.91E-05	--
MSTRG.17381	0.49	0.063333333	-2.951744831	6.58E-06	1.98E-05	pol
ncbi_101798671	0.796666667	0.136666667	-2.543314803	6.65E-06	2.00E-05	FOXF1
ncbi_101790479	0.49	0.033333333	-3.87774425	7.03E-06	2.11E-05	Gnaq
ncbi_101801428	3.123333333	0.613333333	-2.348343282	7.18E-06	2.15E-05	CDK7
ncbi_101800961	0.393333333	0.053333333	-2.882643049	7.62E-06	2.28E-05	PHACTR1
ncbi_101803579	0.926666667	0.123333333	-2.909487707	8.35E-06	2.49E-05	CENPO
ncbi_101802460	0.506666667	0.043333333	-3.547487795	8.42E-06	2.51E-05	B3GALT2

ncbi_113843873	2.57	0.47	-2.451035698	8.47E-06	2.52E-05	C11orf96
ncbi_101800026	0.1	0.683333333	2.772589504	8.74E-06	2.60E-05	Gabrr1
ncbi_101797691	0.346666667	0.003333333	-6.700439718	8.81E-06	2.62E-05	B3GNT7
ncbi_101804073	0.12	0.023333333	-2.362570079	9.08E-06	2.70E-05	ADCY1
ncbi_101804915	0.18	0.023333333	-2.94753258	9.53E-06	2.83E-05	DSP
ncbi_106015775	0.396666667	0.016666667	-4.572889668	9.69E-06	2.87E-05	--
ncbi_101805435	0.001	0.243333333	7.926790153	9.93E-06	2.94E-05	GLRA1
ncbi_101797523	3.373333333	0.023333333	-7.175638653	9.97E-06	2.95E-05	EMB
ncbi_101792886	0.256666667	0.003333333	-6.266786541	1.01E-05	2.99E-05	DCDC2
ncbi_101800236	1.116666667	0.223333333	-2.321928095	1.04E-05	3.06E-05	RBPMS2
ncbi_101790460	0.11	0.006666667	-4.044394119	1.05E-05	3.11E-05	MUC2
ncbi_101795675	0.15	0.72	2.263034406	1.15E-05	3.36E-05	SCG2
ncbi_101804930	0.153333333	0.006666667	-4.523561956	1.16E-05	3.39E-05	Gabrr3
ncbi_101791135	0.356666667	0.04	-3.156504486	1.18E-05	3.45E-05	ANO8
ncbi_101791395	0.026666667	0.253333333	3.247927513	1.19E-05	3.48E-05	CCDC129
ncbi_101801149	0.576666667	0.12	-2.264703226	1.22E-05	3.56E-05	PPP2R2C
ncbi_101805173	0.52	0.001	-9.022367813	1.29E-05	3.76E-05	--
ncbi_101793595	0.263333333	0.006666667	-5.303780748	1.33E-05	3.87E-05	Lpar3
ncbi_101798162	0.001	0.41	8.6794801	1.36E-05	3.96E-05	OIH
ncbi_101802678	0.043333333	0.343333333	2.986060809	1.37E-05	3.98E-05	Asb2
ncbi_101798238	0.506666667	0.056666667	-3.160464672	1.39E-05	4.04E-05	clcnkb
ncbi_101805185	1.736666667	0.306666667	-2.501577606	1.43E-05	4.14E-05	CBR1
ncbi_101804841	1.156666667	0.173333333	-2.738352134	1.43E-05	4.15E-05	Dusp26
ncbi_101791423	0.146666667	0.003333333	-5.459431619	1.45E-05	4.20E-05	Slc6a5
ncbi_101792249	0.29	0.023333333	-3.635588574	1.49E-05	4.29E-05	GPR139

ncbi_113842159	0.636666667	0.001	-9.314394422	1.50E-05	4.33E-05	PIAS2
ncbi_101794229	0.673333333	0.14	-2.26589406	1.53E-05	4.40E-05	RASGRP1
ncbi_101797742	0.25	0.016666667	-3.906890596	1.53E-05	4.41E-05	Drd4
ncbi_101800531	0.11	0.643333333	2.548062918	1.59E-05	4.59E-05	nr4a1
ncbi_101793382	1.503333333	0.366666667	-2.03562391	1.63E-05	4.68E-05	ANKRD1
ncbi_101793849	0.423333333	0.023333333	-4.181329765	1.63E-05	4.68E-05	SELE
ncbi_113840114	0.566666667	0.096666667	-2.551409941	1.63E-05	4.70E-05	AFF3
ncbi_101791486	0.49	0.106666667	-2.199672345	1.66E-05	4.76E-05	ABCG4
ncbi_101802746	3.36	0.78	-2.106915204	1.67E-05	4.80E-05	GNG2
ncbi_101790027	0.21	0.023333333	-3.169925001	1.70E-05	4.86E-05	PIK3C2G
ncbi_101803898	3.64	19.56666667	2.426387647	1.78E-05	5.09E-05	PTPN4
ncbi_101804071	0.18	0.02	-3.169925001	1.92E-05	5.48E-05	MYO15A
ncbi_101802498	0.686666667	0.083333333	-3.042644337	1.96E-05	5.59E-05	SERPINB4
ncbi_101796027	0.21	0.001	-7.714245518	2.06E-05	5.86E-05	--
ncbi_110351189	0.356666667	0.001	-8.478432581	2.12E-05	6.02E-05	Btn1a1
ncbi_101793166	0.001	0.833333333	9.702749879	2.13E-05	6.05E-05	--
ncbi_101799384	0.346666667	0.043333333	-3	2.26E-05	6.40E-05	ATP8A2
ncbi_110353607	1.593333333	0.256666667	-2.634080267	2.37E-05	6.69E-05	LIF
ncbi_101791019	1.743333333	0.056666667	-4.943204295	2.44E-05	6.89E-05	NOX4
ncbi_101802682	0.463333333	0.001	-8.855906667	2.44E-05	6.90E-05	SERPINB10
ncbi_101795824	0.186666667	0.01	-4.222392421	2.56E-05	7.23E-05	PPFIA2
ncbi_101794760	0.49	0.083333333	-2.555816155	2.59E-05	7.30E-05	CHRNA5
ncbi_101794657	0.583333333	0.106666667	-2.451211112	2.85E-05	7.99E-05	PLS1
ncbi_101797234	0.803333333	0.153333333	-2.38932738	2.87E-05	8.04E-05	KIF18B
ncbi_101797439	0.236666667	0.003333333	-6.14974712	2.95E-05	8.28E-05	shroom1

ncbi_101796991	0.7	0.17	-2.041820176	3.10E-05	8.68E-05	Il21r
ncbi_113843724	0.09	0.453333333	2.332575339	3.12E-05	8.71E-05	ISM2
ncbi_101803356	1.173333333	0.206666667	-2.505235308	3.14E-05	8.77E-05	Mns1
ncbi_101797392	0.44	0.02	-4.459431619	3.17E-05	8.85E-05	KRT75
ncbi_101798261	0.066666667	0.333333333	2.321928095	3.20E-05	8.93E-05	Smoc1
ncbi_101801031	0.61	0.086666667	-2.81526012	3.28E-05	9.16E-05	SCN3B
ncbi_101799721	0.203333333	0.001	-7.667702932	3.57E-05	9.93E-05	golD
ncbi_101798928	0.903333333	0.133333333	-2.760220946	4.05E-05	0.000111957	LDLRAD1
ncbi_101802636	0.356666667	0.006666667	-5.741466986	4.12E-05	0.000113786	COL10A1
ncbi_113843270	0.023333333	0.716666667	4.940837928	4.13E-05	0.000113946	Cd24
ncbi_101800681	0.6	0.01	-5.906890596	4.17E-05	0.000115026	Fut7
ncbi_101805181	0.22	0.001	-7.781359714	4.31E-05	0.00011866	SLC7A9
ncbi_101796724	0.243333333	0.001	-7.926790153	4.32E-05	0.000119066	WDR93
ncbi_113841948	0.153333333	0.001	-7.26052755	4.38E-05	0.000120479	--
ncbi_101797468	0.606666667	0.043333333	-3.807354922	4.40E-05	0.000121145	hnf4b
ncbi_110354120	1.046666667	0.24	-2.124695747	4.49E-05	0.000123245	--
ncbi_101802850	0.046666667	0.203333333	2.123382416	4.55E-05	0.000125081	NFASC
ncbi_101802407	0.68	0.09	-2.91753784	4.60E-05	0.000126234	Bhmt
ncbi_101796896	0.36	0.03	-3.584962501	4.77E-05	0.000130551	Slco4c1
ncbi_101790004	0.436666667	0.01	-5.448460501	4.78E-05	0.000130803	TFAP2C
ncbi_101789475	0.286666667	0.033333333	-3.10433666	4.87E-05	0.000133335	DACH1
ncbi_101791813	0.483333333	0.096666667	-2.321928095	4.88E-05	0.000133481	--
ncbi_101793727	0.47	0.006666667	-6.139551352	4.88E-05	0.000133635	--
ncbi_101793032	0.216666667	0.036666667	-2.562936194	4.89E-05	0.000133804	PCDH1
ncbi_101795053	0.45	0.026666667	-4.076815597	5.06E-05	0.000138449	IL18RAP

ncbi_101802920	0.886666667	0.146666667	-2.595850817	5.17E-05	0.000141104	ALB
ncbi_101803154	0.39	0.063333333	-2.622437206	5.22E-05	0.000142338	KIAA1324L
ncbi_101795771	0.486666667	0.07	-2.797507136	5.39E-05	0.000146815	GCNT2
ncbi_101793418	0.023333333	0.223333333	3.258734268	5.48E-05	0.000149179	Cilp
ncbi_101792173	0.703333333	0.133333333	-2.399171094	5.69E-05	0.00015462	Stx11
ncbi_101794677	0.196666667	0.02	-3.297680549	5.77E-05	0.0001568	ZNF536
ncbi_101795545	0.296666667	0.001	-8.212699025	5.85E-05	0.000158771	Rnf165
ncbi_101797573	0.706666667	0.13	-2.442518236	5.93E-05	0.000160982	PGR
ncbi_101793175	0.473333333	0.001	-8.886712714	6.05E-05	0.000164039	SNX31
ncbi_101800584	1.893333333	0.346666667	-2.449307401	6.35E-05	0.000171849	KCNMB2
ncbi_113840208	0.263333333	2.576666667	3.290543856	6.40E-05	0.000173024	MOCS2
ncbi_101790482	0.12	0.003333333	-5.169925001	6.60E-05	0.000178243	BB4
ncbi_101801901	0.013333333	0.2	3.906890596	6.66E-05	0.000179849	SRL
ncbi_113839919	0.726666667	0.076666667	-3.244622369	6.82E-05	0.000183996	--
ncbi_113840613	0.043333333	0.286666667	2.725825037	7.24E-05	0.000194825	--
ncbi_101792576	0.313333333	0.036666667	-3.095157233	7.55E-05	0.00020286	mtnr1c
ncbi_101800301	0.196666667	0.006666667	-4.882643049	7.57E-05	0.000203399	HDAC9
ncbi_101796508	0.23	0.006666667	-5.108524457	7.70E-05	0.000206705	DDC
ncbi_101792387	0.143333333	0.001	-7.163230349	7.78E-05	0.000208668	EXOC3L2
ncbi_101790240	1.076666667	0.26	-2.049988136	7.81E-05	0.000209361	ANKRD13B
ncbi_101804211	1.07	0.03	-5.156504486	7.82E-05	0.00020968	GRP
ncbi_101802855	0.726666667	0.063333333	-3.520256811	7.98E-05	0.000213722	CAMK1G
ncbi_101797649	1.69	0.273333333	-2.628289932	8.00E-05	0.0002141	SPC25
ncbi_101796515	0.266666667	0.053333333	-2.321928095	8.03E-05	0.000214976	Smpd3
ncbi_101800311	0.173333333	0.001	-7.437405312	8.26E-05	0.000220609	--

ncbi_101804606	18.82666667	4.263333333	-2.142723824	8.37E-05	0.000223316	NCOA7
ncbi_101792331	0.413333333	0.066666667	-2.632268215	8.38E-05	0.000223673	COL8A2
ncbi_101796797	0.303333333	0.023333333	-3.700439718	8.43E-05	0.000225062	XKR5
ncbi_101793102	0.001	0.25	7.965784285	8.75E-05	0.000233171	FGB
ncbi_101792494	0.09	0.773333333	3.103093493	8.86E-05	0.000235957	Fzd8
ncbi_101800740	0.286666667	0.043333333	-2.725825037	8.86E-05	0.000235963	Gabbr2
ncbi_101791565	1.15	0.12	-3.26052755	9.00E-05	0.00023946	C1qtnf5
ncbi_101790301	0.753333333	0.076666667	-3.296617006	9.60E-05	0.000254549	--
ncbi_113840134	0.06	0.8	3.736965594	9.65E-05	0.000255824	FREM1
ncbi_101793753	0.68	0.026666667	-4.672425342	9.65E-05	0.0002559	Sh3yl1
ncbi_101798886	2.02	0.443333333	-2.187891548	0.000101355	0.000268093	HMGB2
ncbi_101789478	0.323333333	0.001	-8.336878436	0.000102833	0.000271674	NKX6-2
ncbi_101798358	0.19	0.013333333	-3.832890014	0.00010481	0.000276562	DNAH17
ncbi_101796115	0.123333333	0.01	-3.624490865	0.000105846	0.00027924	MYO7A
ncbi_101803835	0.16	0.003333333	-5.584962501	0.000111142	0.000292621	SLC34A2
ncbi_101795219	0.26	0.013333333	-4.285402219	0.000113664	0.00029902	ADGRG4
ncbi_101795500	0.001	0.19	7.569855608	0.000114218	0.000300357	LRRC3B
ncbi_101794422	0.043333333	0.54	3.639410285	0.00011541	0.000303428	CASQ2
ncbi_101791143	0.19	0.003333333	-5.832890014	0.000123054	0.000322814	slc32a1
ncbi_101802759	0.236666667	0.02	-3.564784619	0.000127914	0.000335225	SLC6A4
ncbi_101799598	0.366666667	0.046666667	-2.974004791	0.000129051	0.000337866	Card10
ncbi_101797553	0.186666667	0.023333333	-3	0.000129202	0.000338197	Shisa6
ncbi_101802681	0.22	0.003333333	-6.044394119	0.000130251	0.000340736	--
ncbi_101801718	0.096666667	0.001	-6.594946589	0.000133797	0.000349524	UNC80
ncbi_101801193	0.553333333	0.073333333	-2.915607813	0.00013697	0.000357242	ALDH8A1

ncbi_101801372	0.276666667	0.001	-8.112005026	0.000142023	0.000369921	NXPH1
ncbi_101794280	0.156666667	0.003333333	-5.554588852	0.000149225	0.000387812	CHRNA9
ncbi_113842837	0.433333333	0.073333333	-2.562936194	0.000152948	0.000396887	Lama1
ncbi_113845664	0.916666667	0.093333333	-3.295932886	0.000154084	0.000399647	Traf4
ncbi_113842153	0.001	0.42	8.714245518	0.000154874	0.000401615	PLAU
ncbi_101789946	0.88	0.043333333	-4.343954401	0.000158529	0.000410525	VSTM5
ncbi_101795161	0.066666667	0.566666667	3.087462841	0.000159445	0.000412652	EEF1A2
ncbi_101797163	1.286666667	0.2	-2.685566442	0.000166673	0.000430336	ALOX5AP
ncbi_101795796	0.36	0.033333333	-3.432959407	0.000168014	0.000433627	ESRP2
ncbi_101797865	0.16	0.001	-7.321928095	0.000168339	0.000434381	TACR3
ncbi_101802566	0.19	0.001	-7.569855608	0.000180343	0.0004638	GUCA1A
ncbi_101794519	0.68	0.103333333	-2.718229032	0.000182733	0.000469392	SAMSN1
ncbi_101789382	1.13	0.17	-2.732716121	0.000185537	0.000476221	SCEL
ncbi_101796043	0.253333333	0.006666667	-5.247927513	0.000193376	0.000495078	Foxa1
ncbi_101802833	0.203333333	0.001	-7.667702932	0.000194389	0.000497478	SOX3
ncbi_101792798	0.413333333	0.013333333	-4.95419631	0.000201606	0.000514739	CD3E
ncbi_101793931	0.006666667	0.093333333	3.807354922	0.00020864	0.000531661	DCC
ncbi_101789976	0.12	0.003333333	-5.169925001	0.000214027	0.000544646	TRPM1
ncbi_101791168	0.693333333	0.136666667	-2.342887714	0.000218505	0.000555067	Brsk2
ncbi_101804544	0.386666667	0.046666667	-3.050626073	0.000222791	0.000565079	--
ncbi_101798037	0.176666667	0.006666667	-4.727920455	0.000232147	0.000587127	CD200
ncbi_101795989	0.29	0.006666667	-5.442943496	0.000235364	0.000595122	Asb14
ncbi_101792901	0.123333333	0.026666667	-2.209453366	0.000239899	0.000605535	GPER1
ncbi_101790942	0.413333333	0.033333333	-3.632268215	0.000240674	0.000607256	SGIP1
ncbi_101790127	25.35	0.643333333	-5.300275495	0.000241901	0.000610118	NSDHL

ncbi_101795241	0.27	0.006666667	-5.339850003	0.000245506	0.000618374	ANO9
ncbi_101793802	0.33	0.043333333	-2.928916902	0.000256132	0.000643527	CDH17
ncbi_113845446	0.443333333	0.096666667	-2.19730144	0.000259332	0.000651443	--
ncbi_113841676	1.073333333	0.001	-10.06788247	0.00026642	0.000667837	KCMF1
ncbi_101795922	0.49	0.07	-2.807354922	0.000267553	0.000670291	OCA2
ncbi_101798680	0.096666667	0.001	-6.594946589	0.000270539	0.000677253	Scn1a
ncbi_101798633	0.206666667	1.55	2.906890596	0.000270656	0.000677416	Nipa1
ncbi_113843537	0.496666667	0.05	-3.312277925	0.0002785	0.000695585	TMPRSS11E
ncbi_101793529	0.116666667	0.003333333	-5.129283017	0.000279175	0.000697004	TNNI3K
ncbi_113841024	1.53	0.366666667	-2.06099063	0.000289365	0.000721345	--
ncbi_101800704	0.53	0.083333333	-2.669026766	0.000296587	0.000738222	SOWAHA
ncbi_101795729	0.173333333	0.001	-7.437405312	0.000297949	0.000741049	GABRA5
ncbi_101803878	0.05	0.286666667	2.519374159	0.000301054	0.000748343	PTPRT
ncbi_101798236	0.006666667	0.09	3.754887502	0.000302543	0.000751903	OPN3
ncbi_101804072	0.153333333	0.013333333	-3.523561956	0.000303644	0.000754209	KIAA1211L
ncbi_101792419	0.516666667	0.07	-2.883806982	0.000314184	0.000778466	TSPAN2
ncbi_113842241	0.343333333	0.001	-8.423466121	0.000325673	0.000804496	ZSWIM6
ncbi_101796734	0.15	0.016666667	-3.169925001	0.000327185	0.000808078	VWDE
ncbi_113842138	0.01	0.073333333	2.874469118	0.000342402	0.000844067	MKI67
ncbi_101805356	0.303333333	0.01	-4.922832139	0.000344907	0.00084992	tbpl2
ncbi_101792103	0.476666667	0.013333333	-5.159871337	0.000349216	0.000860054	--
ncbi_113845511	0.14	0.01	-3.807354922	0.000350203	0.000862322	--
MSTRG.17782	0.553333333	0.013333333	-5.375039431	0.000353699	0.000870439	VLDLR
ncbi_101797500	0.35	0.016666667	-4.392317423	0.000357977	0.000880139	Mas1
ncbi_101793415	0.03	0.001	-4.906890596	0.000362116	0.000888978	Dnah8

ncbi_101791452	0.186666667	0.03	-2.637429921	0.000364001	0.000893271	LRRC66
ncbi_101792314	0.013333333	0.246666667	4.209453366	0.000368728	0.000904023	BRINP1
ncbi_101799836	0.53	0.03	-4.142957954	0.000371928	0.000911526	KBP
ncbi_101797124	0.24	0.033333333	-2.847996907	0.000373519	0.000915083	MEIOB
ncbi_113845590	0.443333333	0.1	-2.14839184	0.00038317	0.000937499	CBARP
ncbi_101797114	0.053333333	0.001	-5.736965594	0.000394257	0.000963364	ZNF804B
ncbi_101794008	0.336666667	0.07	-2.26589406	0.000397259	0.000969806	PLPPR5
ncbi_101796858	0.626666667	0.001	-9.291554446	0.000399461	0.000974622	Bcl2l15
ncbi_101796503	0.12	0.001	-6.906890596	0.000406455	0.000990948	Avpr1a
ncbi_101794036	0.32	0.056666667	-2.497499659	0.000408975	0.000996349	DLGAP1
ncbi_101791850	0.086666667	0.473333333	2.449307401	0.000412386	0.001003723	NAT1
ncbi_101793135	0.156666667	0.003333333	-5.554588852	0.000418363	0.001017513	--
ncbi_101796550	0.533333333	0.13	-2.036525876	0.000423934	0.001030489	RUNDC3B
ncbi_101804155	0.036666667	0.001	-5.196397213	0.000447152	0.001083301	IL1RAPL1
ncbi_101789756	0.436666667	0.076666667	-2.509861045	0.00044824	0.001085737	Ss18l1
ncbi_101790702	0.25	0.046666667	-2.421463768	0.000451339	0.001092839	SKA3
ncbi_113839714	0.856666667	0.166666667	-2.361768359	0.000465417	0.001124013	AADACL4
ncbi_101804552	2.216666667	0.343333333	-2.690710003	0.000475436	0.001146304	PLAU
ncbi_101797774	0.063333333	0.001	-5.984893108	0.000483278	0.001163495	CD200R1
ncbi_101794961	0.74	0.113333333	-2.706953025	0.000483631	0.001164133	HOXC13
ncbi_101801403	0.126666667	0.016666667	-2.925999419	0.000497176	0.001194317	Kcnk10
ncbi_101794749	0.166666667	0.003333333	-5.64385619	0.000498042	0.001195959	PLA2G4E
ncbi_101792142	0.386666667	0.063333333	-2.610053482	0.000502992	0.001207182	GRIP1
ncbi_101804148	0.283333333	0.006666667	-5.409390936	0.000504369	0.001210263	ZP2
ncbi_101799338	0.343333333	0.056666667	-2.599037686	0.000505453	0.001212643	DSG2

ncbi_101791302	0.156666667	0.001	-7.291554446	0.000511533	0.001226554	Muc6
ncbi_101789730	0.21	0.003333333	-5.977279923	0.000519544	0.001244849	Cyp2j6
ncbi_101793951	0.02	0.243333333	3.604862058	0.000525325	0.00125778	SLC35F4
ncbi_101794433	0.536666667	0.073333333	-2.871485259	0.000540591	0.001292203	ZNF488
ncbi_101798540	2.39	0.413333333	-2.531632998	0.000543272	0.001298374	TK1
ncbi_101801105	0.356666667	0.006666667	-5.741466986	0.000556092	0.001326346	CLUL1
ncbi_101792199	1.626666667	0.363333333	-2.162553013	0.000561096	0.001337793	SLC17A9
ncbi_113840873	0.153333333	0.001	-7.26052755	0.000568154	0.00135388	gag-pol
ncbi_101804317	9.243333333	0.883333333	-3.387383352	0.000573234	0.001365737	CSF1
ncbi_106014640	0.18	0.001	-7.491853096	0.000575859	0.001371242	--
ncbi_101797923	0.353333333	0.001	-8.464886049	0.000588204	0.001399619	Aldh18a1
ncbi_101791392	0.086666667	0.013333333	-2.700439718	0.000589429	0.001402279	ANKFN1
ncbi_101795935	1.666666667	0.396666667	-2.070966521	0.000589801	0.00140291	AURKB
ncbi_101802974	0.53	0.04	-3.727920455	0.000607335	0.001440686	Epsti1
ncbi_101803482	0.073333333	0.006666667	-3.459431619	0.000621777	0.001471741	KBTBD12
ncbi_101790662	0.563333333	0.036666667	-3.941447818	0.000631021	0.001492542	Efcab2
ncbi_101801617	0.176666667	0.001	-7.464886049	0.00063287	0.001496373	Enkur
ncbi_113839606	0.903333333	0.096666667	-3.224168046	0.000633644	0.001497663	ctp
ncbi_101797799	0.506666667	0.073333333	-2.788495895	0.000642127	0.001516343	Hoxc5
ncbi_101803605	0.026666667	0.516666667	4.276124405	0.000645443	0.001523349	Myl2
ncbi_101800113	0.46	0.036666667	-3.649092838	0.000650604	0.001534421	Gpa33
ncbi_101802335	0.206666667	0.026666667	-2.95419631	0.000668463	0.001574837	RGS20
ncbi_101797213	0.623333333	0.046666667	-3.739539538	0.000671958	0.001582217	--
ncbi_101802757	0.243333333	0.01	-4.604862058	0.000675855	0.001590533	SLC18A2
ncbi_101791306	0.543333333	0.056666667	-3.261265313	0.000680291	0.001600108	C2cd4cC2CD4

ncbi_113840402	0.193333333	0.001	-7.594946589	0.000691194	0.001624294	RX2
ncbi_113845090	0.09	0.001	-6.491853096	0.000691194	0.001624294	Prrt3
ncbi_101789461	0.56	0.083333333	-2.748461233	0.000692263	0.001626514	Ush1c
ncbi_113841388	1.736666667	0.203333333	-3.094402225	0.000692851	0.001627603	sbf2
ncbi_101794844	0.09	0.693333333	2.945552216	0.000693542	0.001628642	--
ncbi_101798563	0.236666667	0.02	-3.564784619	0.00070983	0.001665693	Dlgap4
ncbi_101805481	0.286666667	0.001	-8.163230349	0.00072723	0.001704079	Nipal2
ncbi_101797440	0.116666667	0.006666667	-4.129283017	0.000732519	0.001715551	Col4a4
ncbi_101800214	0.14	0.03	-2.222392421	0.000741976	0.001736275	Fam19a5
ncbi_101802524	0.236666667	0.053333333	-2.14974712	0.00077548	0.001810653	Ptprn2
ncbi_101790718	0.203333333	0.001	-7.667702932	0.000794189	0.001850701	GRPR
ncbi_101792222	0.523333333	0.11	-2.25022663	0.000847162	0.001966787	SPATA17
ncbi_101800597	0.283333333	0.033333333	-3.087462841	0.000848534	0.001969273	Fam214b
ncbi_101796147	0.03	0.373333333	3.637429921	0.000863697	0.002001976	NOG
ncbi_113844283	0.353333333	0.066666667	-2.40599236	0.000867212	0.002009412	--
ncbi_101792034	0.523333333	0.043333333	-3.594181031	0.000880698	0.002038133	Slc10a4
ncbi_113843649	0.346666667	1.733333333	2.321928095	0.000897135	0.002073238	NKX3-2
ncbi_101798363	0.436666667	0.046666667	-3.226068079	0.000934987	0.002154622	SPACA9
ncbi_101790993	0.386666667	0.03	-3.688055994	0.000958791	0.002207144	Ces1d
ncbi_101799852	0.253333333	0.043333333	-2.547487795	0.000963938	0.00221821	DYTN
ncbi_106020099	0.403333333	0.053333333	-2.918863237	0.000970711	0.002233403	ERV3-1
ncbi_113842822	0.153333333	0.01	-3.938599455	0.000988805	0.002274235	TGM5
ncbi_101799891	0.213333333	0.036666667	-2.540568381	0.00100395	0.002306635	Plekha6
ncbi_101791853	0.263333333	0.04	-2.718818247	0.001018318	0.002336772	EYA1
ncbi_101795135	0.363333333	1.513333333	2.058364163	0.001019419	0.002338889	Adprh

ncbi_101799146	0.173333333	0.001	-7.437405312	0.001025444	0.002352299	KRT6C
ncbi_101805220	0.08	0.001	-6.321928095	0.001025694	0.00235246	PLCH1
ncbi_101803570	0.333333333	0.023333333	-3.836501268	0.001029099	0.002359441	gyaR
ncbi_101798870	1.363333333	0.036666667	-5.216525414	0.001063944	0.002435501	INSC
ncbi_113844993	0.143333333	0.001	-7.163230349	0.001077491	0.002462621	foxc2
ncbi_101805398	0.24	0.001	-7.906890596	0.001079197	0.00246609	Ubap1
ncbi_101804377	0.496666667	0.073333333	-2.759736902	0.001091227	0.002492709	SLCO5A1
ncbi_101794331	0.206666667	0.001	-7.691161905	0.001124021	0.002563592	--
ncbi_101798764	0.583333333	0.073333333	-2.991779493	0.001138631	0.002592844	Dnaaf1
ncbi_101802084	0.23	0.001	-7.845490051	0.001192809	0.0027082	Sstr5
ncbi_101794945	0.213333333	0.006666667	-5	0.001213276	0.002751804	ARHGAP8
ncbi_113842125	0.73	0.133333333	-2.452858965	0.001214957	0.002755138	PGR
ncbi_101790094	0.276666667	0.01	-4.790076931	0.0012474	0.002824791	CYP2J2
ncbi_101792178	3.143333333	0.736666667	-2.093211401	0.001254251	0.002839813	Cltb
ncbi_101790022	0.243333333	0.036666667	-2.73039294	0.001261927	0.002855711	Mfng
ncbi_101791376	0.196666667	0.001	-7.619608644	0.00127666	0.002886554	Upp1
ncbi_101803487	0.086666667	0.006666667	-3.700439718	0.001299991	0.002935247	Cnih3
ncbi_101801884	0.143333333	0.001	-7.163230349	0.001306855	0.002949725	FASLG
ncbi_101804947	0.11	0.01	-3.459431619	0.001315336	0.002967334	Rab38
ncbi_101805432	0.233333333	0.001	-7.866248611	0.001345312	0.003030253	CYP2C19
ncbi_101798141	41.83	0.27	-7.27543478	0.001383729	0.003111963	Rrad
ncbi_101793354	0.001	0.126666667	6.984893108	0.001399439	0.003144051	KCTD14
ncbi_101797637	0.593333333	0.143333333	-2.049468676	0.001469388	0.00329441	Mapk8ip1
ncbi_101799935	0.206666667	0.03	-2.784271309	0.001471115	0.003297717	MYLK4
ncbi_101803185	0.22	0.006666667	-5.044394119	0.001491843	0.003341891	SH2D1B

ncbi_101803831	0.096666667	0.013333333	-2.857980995	0.00149806	0.003355245	TMEM132B
ncbi_113843423	0.043333333	0.003333333	-3.700439718	0.001525883	0.003414053	Poln
ncbi_101793312	0.083333333	0.001	-6.380821784	0.001542957	0.003449893	Fbxo47
ncbi_101797984	0.036666667	0.223333333	2.606657572	0.00156136	0.003488061	Slitrk2
ncbi_101795243	0.053333333	0.001	-5.736965594	0.001580516	0.003529047	RHCG
ncbi_101801434	0.01	0.096666667	3.273018494	0.001583569	0.00353526	KCNA5
ncbi_101796299	0.156666667	0.703333333	2.166510337	0.001605274	0.003581881	Syndig1
ncbi_101790745	0.293333333	0.066666667	-2.137503524	0.001612696	0.003597215	ARHGEF26
ncbi_101798799	0.176666667	0.006666667	-4.727920455	0.001640447	0.003653519	Grin3b
ncbi_101802433	0.133333333	0.001	-7.058893689	0.001673566	0.003724097	PCSK9
ncbi_101803142	0.283333333	0.033333333	-3.087462841	0.00167648	0.003729312	PLA2G12B
ncbi_113840163	0.113333333	0.003333333	-5.087462841	0.001693534	0.003764689	IFN
ncbi_101791788	0.336666667	0.03	-3.488286481	0.00172072	0.003822523	RAPGEF3
ncbi_101797958	0.31	0.016666667	-4.217230716	0.001721379	0.003823338	C1QTNF7
ncbi_101801357	5.066666667	0.176666667	-4.841935154	0.001723526	0.003827457	ARNT2
ncbi_101791654	0.076666667	0.363333333	2.244622369	0.001735507	0.003852755	NAT1
ncbi_101791175	0.113333333	0.01	-3.502500341	0.001773414	0.003932902	Scn4a
ncbi_101804651	0.23	0.056666667	-2.021061616	0.001783991	0.003953007	Fbxo16
ncbi_101793025	0.21	0.02	-3.392317423	0.001842536	0.004075139	Arhgap22
ncbi_101794720	0.453333333	0.08	-2.502500341	0.001845932	0.00408127	AMPD1
ncbi_101795247	0.006666667	0.076666667	3.523561956	0.001853241	0.004096736	KLHL31
ncbi_101795930	0.206666667	0.006666667	-4.95419631	0.001882694	0.004160439	TMEM72
ncbi_113842968	0.813333333	0.18	-2.175849835	0.001890566	0.004175014	CSPG5
ncbi_113844749	0.546666667	0.023333333	-4.550197083	0.001896803	0.004186673	MADPRT
ncbi_101804819	0.083333333	0.336666667	2.014355293	0.001931211	0.004258302	TAF4

ncbi_101803318	0.39	0.073333333	-2.410933101	0.00196556	0.004326746	SLC1A1
ncbi_101805303	0.873333333	0.11	-2.989028882	0.001967206	0.004328184	TPD52L1
ncbi_101794025	0.093333333	0.001	-6.544320516	0.001990619	0.004377488	OVCH1
ncbi_106019854	0.153333333	0.006666667	-4.523561956	0.001993522	0.004383134	DOK2
ncbi_101793210	0.073333333	0.001	-6.196397213	0.002024719	0.004447989	Acsm4
ncbi_101802422	0.076666667	0.001	-6.26052755	0.002037868	0.004474621	lmo3
ncbi_101801038	0.286666667	0.001	-8.163230349	0.002056015	0.004513708	CHD1
ncbi_101799709	0.033333333	0.001	-5.058893689	0.002062031	0.004525397	--
ncbi_106016162	0.086666667	0.003333333	-4.700439718	0.002090248	0.004585016	FAM237A
ncbi_101796257	0.29	0.013333333	-4.442943496	0.00210999	0.004623668	GATM
ncbi_101797082	0.073333333	0.706666667	3.268488836	0.002125894	0.004654921	TMEFF2
ncbi_101794223	0.186666667	0.01	-4.222392421	0.002164374	0.004734908	CCDC170
ncbi_101796873	0.376666667	0.076666667	-2.296617006	0.002205092	0.004818342	--
ncbi_101791501	0.67	0.136666667	-2.293499687	0.002207118	0.004821964	ALPL
ncbi_101803381	0.126666667	0.01	-3.662965013	0.002239782	0.004887613	SPTB
ncbi_101799616	0.33	0.05	-2.722466024	0.002259647	0.004928495	ERICH6
ncbi_101803717	0.376666667	0.053333333	-2.820178962	0.002298192	0.005007555	Slc35f4
ncbi_101799140	0.32	0.053333333	-2.584962501	0.002366042	0.005147676	Pkd2l2
ncbi_101801051	0.086666667	0.001	-6.437405312	0.002387274	0.005192142	CCDC69
ncbi_106015823	0.16	0.006666667	-4.584962501	0.002406892	0.005230461	--
ncbi_101805271	0.336666667	0.056666667	-2.570748642	0.00241333	0.005241838	TJP3
ncbi_101802057	0.263333333	0.013333333	-4.303780748	0.002418908	0.005253082	C6orf118
ncbi_101804267	0.146666667	0.001	-7.196397213	0.002466346	0.005349885	CYP2W1
ncbi_106017693	0.003333333	0.046666667	3.807354922	0.002484731	0.005387084	C14orf132
ncbi_101797491	0.173333333	0.01	-4.115477217	0.002504055	0.005424484	--

ncbi_101799048	0.15	0.006666667	-4.491853096	0.002540281	0.005496588	Kdf1
ncbi_106015769	0.24	0.013333333	-4.169925001	0.002662451	0.005735319	klhl25
ncbi_101796391	0.001	0.236666667	7.886712714	0.002673608	0.005758404	Dnase1l3
ncbi_101798592	1.286666667	0.07	-4.200139614	0.002692695	0.005797606	Fabp6
ncbi_113840462	0.056666667	0.001	-5.824428435	0.002714908	0.005842547	Eppk1
ncbi_101796039	1.006666667	0.093333333	-3.431049817	0.002797704	0.006013799	AP4S1
ncbi_113839705	0.84	0.2	-2.070389328	0.002847739	0.006116325	SOX21
ncbi_101803082	0.04	0.226666667	2.502500341	0.002870832	0.0061639	GANAB
ncbi_101790743	0.08	0.001	-6.321928095	0.002908128	0.006238857	SLC17A6
ncbi_113845046	0.066666667	0.006666667	-3.321928095	0.003193642	0.00681227	--
ncbi_101790829	0.183333333	0.02	-3.196397213	0.003233493	0.006891655	MAP7D2
ncbi_101805361	0.446666667	0.043333333	-3.365649472	0.003266999	0.006957399	PLVAP
ncbi_101792490	0.076666667	0.003333333	-4.523561956	0.003284595	0.006990319	TRPC6
ncbi_101789673	0.363333333	0.06	-2.598259323	0.00328559	0.0069913	TRAF3IP3
ncbi_101802954	1.53	0.236666667	-2.692603224	0.003300363	0.007019308	YPTB3174
ncbi_101801276	0.123333333	0.003333333	-5.209453366	0.003303939	0.007025773	SLC17A8
ncbi_101791518	0.746666667	0.07	-3.415037499	0.003348691	0.007117465	C2orf50
ncbi_101798463	0.296666667	0.02	-3.89077093	0.003354136	0.007126721	MEP1A
ncbi_101790994	0.493333333	0.073333333	-2.750021747	0.003388062	0.007192964	kpna1
ncbi_113839601	0.193333333	0.01	-4.273018494	0.003437256	0.007288523	CNGA3
ncbi_101800069	0.26	0.03	-3.115477217	0.003438995	0.00729027	Nrip3
ncbi_101799784	0.74	0.123333333	-2.584962501	0.003499377	0.007411245	Fgfbp1
ncbi_101797887	0.001	0.103333333	6.691161905	0.003531	0.007472171	Dusp11
ncbi_101792500	0.193333333	0.006666667	-4.857980995	0.003560622	0.007529981	TMEM27
ncbi_113841640	0.19	0.883333333	2.216958535	0.003596541	0.007604714	Mki67

ncbi_101789628	0.486666667	0.076666667	-2.666262603	0.003766521	0.00794102	NEXN
ncbi_101800123	0.036666667	0.001	-5.196397213	0.003938917	0.008265848	AMER2
ncbi_101801464	1.213333333	0.293333333	-2.048363022	0.004051023	0.008481621	ZFAND5
ncbi_101803387	0.29	0.033333333	-3.121015401	0.004136014	0.008647549	CALHM6
ncbi_101793766	0.093333333	0.003333333	-4.807354922	0.004157605	0.008691301	FZD10
ncbi_101794074	0.001	0.253333333	7.984893108	0.00416672	0.008707573	PRDX3
ncbi_113839582	0.07	0.783333333	3.484199524	0.004196871	0.00876778	THRB
ncbi_101794713	0.133333333	0.001	-7.058893689	0.004248402	0.008865523	Blk
ncbi_101803179	0.01	0.103333333	3.36923381	0.004273551	0.008912316	PPP4R4
ncbi_110352706	0.243333333	0.04	-2.604862058	0.0043012	0.008961405	--
ncbi_101795253	0.08	0.003333333	-4.584962501	0.00431251	0.008982106	A2ML1
ncbi_101800770	0.223333333	0.03	-2.896164189	0.004399958	0.009158267	GALNT14
ncbi_101802619	0.406666667	0.083333333	-2.286881148	0.004428872	0.009208336	syt17
ncbi_101796874	0.036666667	0.001	-5.196397213	0.004461171	0.009269597	LIPA
ncbi_101801340	0.363333333	0.053333333	-2.768184325	0.00455973	0.009463864	PLA1A
ncbi_101792697	0.186666667	0.016666667	-3.485426827	0.00461788	0.009578477	ACE2
ncbi_101803786	0.27	0.023333333	-3.532495081	0.004647842	0.009636039	TRAPPC3L
ncbi_101796885	0.083333333	0.003333333	-4.64385619	0.004652496	0.009644159	COLQ
ncbi_101797494	0.001	0.07	6.129283017	0.00465784	0.009653707	CYP1B1
ncbi_101793630	0.413333333	0.08	-2.36923381	0.00467038	0.009676631	RHOH
ncbi_101798736	0.086666667	0.003333333	-4.700439718	0.004679456	0.009692364	Fgf14
ncbi_101801396	0.633333333	0.14	-2.177538186	0.004685372	0.009703081	Nqo2
ncbi_101789901	0.176666667	0.036666667	-2.268488836	0.004686986	0.009704889	AK9
ncbi_101791021	0.396666667	0.05	-2.987927168	0.004759916	0.00984966	ENPP5
ncbi_101791494	0.29	0.04	-2.857980995	0.004773516	0.009874677	Olfr1020

ncbi_113840184	0.08	0.003333333	-4.584962501	0.004776071	0.009878402	Onecut2
ncbi_101803583	0.69	0.163333333	-2.078777113	0.004787256	0.00989997	daw1
ncbi_101804147	0.056666667	0.003333333	-4.087462841	0.004794623	0.009913638	Lpar5
ncbi_101789465	0.266666667	0.056666667	-2.234465254	0.004842008	0.010005286	SH3BP1
ncbi_113840877	0.243333333	0.04	-2.604862058	0.004862253	0.01004236	--
ncbi_101803953	0.001	0.206666667	7.691161905	0.004925179	0.010162697	TNNT2
ncbi_101795534	0.32	0.036666667	-3.125530882	0.004984873	0.010279384	Rbm46
ncbi_101802200	0.023333333	0.001	-4.544320516	0.004993005	0.010294532	Dnah1
ncbi_101800708	0.083333333	0.001	-6.380821784	0.005195479	0.010690084	ATP12A
ncbi_113841762	0.623333333	0.09	-2.792006958	0.005233144	0.010764195	Snap91
ncbi_101791855	0.206666667	0.01	-4.36923381	0.005256657	0.010805763	--
MSTRG.17371	0.986666667	0.173333333	-2.509013647	0.005261559	0.010814142	DCAF12
ncbi_101804982	0.126666667	0.003333333	-5.247927513	0.005324053	0.010932278	ANKDD1B
ncbi_101793541	0.033333333	0.001	-5.058893689	0.005335464	0.010953989	RNF182
ncbi_101804776	0.166666667	0.023333333	-2.836501268	0.00535204	0.010981128	VWA3B
ncbi_101801563	0.001	0.073333333	6.196397213	0.005379107	0.01103147	MYZAP
ncbi_106019077	1.873333333	0.33	-2.5050697	0.005390797	0.011050247	Dnttip1
ncbi_101798059	0.106666667	0.01	-3.415037499	0.005413998	0.01108974	SLC26A9
ncbi_101796881	10.68333333	2.623333333	-2.025888816	0.005414361	0.01108974	TGFBI
ncbi_101793229	0.133333333	0.001	-7.058893689	0.005467332	0.011187845	RASL12
ncbi_101805304	0.28	0.03	-3.222392421	0.005583871	0.011403128	SLAIN1
ncbi_101794811	0.07	0.006666667	-3.392317423	0.005648594	0.011524507	PLD5
ncbi_101790414	0.596666667	0.123333333	-2.274362412	0.005654332	0.011534415	Drc3
ncbi_101804810	0.036666667	0.213333333	2.540568381	0.005701813	0.011624349	TMED11
ncbi_101800971	0.173333333	0.016666667	-3.378511623	0.005712662	0.011642508	NIM1K

ncbi_101795955	0.363333333	0.001	-8.505149919	0.005768456	0.011743409	Ctif
ncbi_101789763	0.103333333	0.016666667	-2.632268215	0.005778321	0.011759673	FHAD1
ncbi_101794922	0.39	0.001	-8.607330314	0.005858856	0.01190521	VASH1
ncbi_101803013	0.413333333	0.013333333	-4.95419631	0.006022294	0.012210821	CCL20
ncbi_101798906	0.346666667	0.02	-4.115477217	0.006025606	0.01221558	Cd274
ncbi_101798338	0.026666667	0.133333333	2.321928095	0.0061068	0.012364852	FRMPD4
ncbi_101796057	0.106666667	0.001	-6.736965594	0.006112961	0.01237541	ABRA
ncbi_101802777	1.12	0.25	-2.163498732	0.006121515	0.012390477	RICTOR
ncbi_113840712	0.383333333	0.043333333	-3.145050333	0.006200996	0.012541986	Smco3
ncbi_101802358	0.13	0.006666667	-4.285402219	0.006202666	0.012543425	CACNG4
ncbi_101800734	0.16	0.006666667	-4.584962501	0.006214473	0.012565358	ADCYAP1
ncbi_101794695	0.001	0.066666667	6.058893689	0.006339971	0.012795374	Col18a1
ncbi_101800221	0.133333333	0.001	-7.058893689	0.006503352	0.013094804	mfsd2b
ncbi_101800808	0.096666667	0.003333333	-4.857980995	0.006560946	0.013200612	ABCC2
ncbi_101804330	0.21	0.03	-2.807354922	0.0065709	0.013217148	CFAP221
ncbi_101795212	0.001	0.06	5.906890596	0.006573552	0.013219874	ENPEP
ncbi_101802176	0.006666667	0.106666667	4	0.006576443	0.013223654	CCKAR
ncbi_106014932	0.363333333	0.04	-3.183221824	0.006609051	0.013283095	CXCL8
ncbi_101790140	0.37	0.023333333	-3.987060944	0.006751004	0.013549661	--
ncbi_101790857	0.203333333	0.01	-4.345774837	0.006758493	0.013558449	METTL24
ncbi_101796480	0.323333333	0.026666667	-3.599912842	0.006813989	0.013663495	DPYS
ncbi_101801757	0.08	0.001	-6.321928095	0.006834157	0.013701835	SLC9A2
ncbi_101803132	0.12	0.006666667	-4.169925001	0.006976207	0.013969501	tfap2e
ncbi_101798815	0.146666667	0.006666667	-4.459431619	0.00701756	0.01404371	prom1a
ncbi_101799906	0.086666667	0.353333333	2.027480736	0.007070948	0.014139735	TENT5C

ncbi_101801095	6.8	0.993333333	-2.775184916	0.007113104	0.014213167	Stxbp6
ncbi_113842085	0.336666667	0.023333333	-3.850856561	0.007137336	0.014254238	EML6
ncbi_101797118	0.166666667	0.001	-7.380821784	0.007170131	0.014316183	CETP
ncbi_106015349	0.19	0.006666667	-4.832890014	0.007197468	0.014368572	--
ncbi_101802430	0.036666667	0.001	-5.196397213	0.007212453	0.014394094	ARHGEF33
ncbi_101800423	0.243333333	0.05	-2.282933963	0.007232489	0.014427475	ITGBL1
ncbi_101801848	0.293333333	0.001	-8.196397213	0.007302845	0.014554505	SH3GL2
ncbi_101794169	0.001	0.133333333	7.058893689	0.007344243	0.014630323	Krt13
ncbi_101798720	0.49	0.056666667	-3.112209504	0.007374719	0.014684325	Iqcg
ncbi_101791417	0.19	0.033333333	-2.510961919	0.007400196	0.014730568	ASIC1
ncbi_101791201	0.226666667	0.001	-7.824428435	0.007626889	0.015156443	S100Z
ncbi_101794800	0.103333333	0.001	-6.691161905	0.007998356	0.015844086	LRIT3
ncbi_101794499	0.06	0.003333333	-4.169925001	0.00805655	0.015952115	STAB2
ncbi_101799649	0.333333333	0.001	-8.380821784	0.008318612	0.01643617	NRIP3
ncbi_101801456	0.183333333	0.016666667	-3.459431619	0.008394232	0.016570297	ADGRG1
ncbi_101794234	0.24	0.01	-4.584962501	0.008560129	0.016870045	TMEM171
ncbi_110354335	0.053333333	0.001	-5.736965594	0.008589768	0.016923357	Caskin1
ncbi_101804858	0.626666667	0.11	-2.510194732	0.008771027	0.017262268	DLGAP5
ncbi_101800880	0.263333333	0.06	-2.133855747	0.008776301	0.017267452	IL9R
ncbi_101795602	0.806666667	0.17	-2.246437895	0.008795366	0.017302339	TUBA3C
ncbi_110352867	0.206666667	0.001	-7.691161905	0.009031501	0.017732218	VPS33B
ncbi_101798264	0.053333333	0.001	-5.736965594	0.009107462	0.017873309	VTG2
ncbi_101795818	0.113333333	0.001	-6.824428435	0.009168835	0.017980264	Clvs1
ncbi_113840026	0.08	0.746666667	3.222392421	0.009406246	0.018404442	Pgap3
ncbi_101792942	0.126666667	0.006666667	-4.247927513	0.009709295	0.018952035	CSF2RA

ncbi_101801009	0.02	0.16	3	0.00975423	0.019036904	CDK15

Description: Sample: id: gene id; SCP0-1_count: gene count value for SCP0-1; SCP0-4_count: gene count value for SCP0-4; log2(FC): logarithmic value of the multiplicity of difference in FPKM between samples IMP0 and IMP4, bottomed by 2; P_value: significance P-value; FDR: P-value after BH correction; Symbol: gene name; Symbol. Gene name; KEGG_A_class KEGG: first level annotation; KEGG_B_class: KEGG second level annotation; K_ID: KEGG pathway ID.
