# Supplementary material for: Identification of genes and long non-coding RNAs for intramuscular and subcutaneous fat deposition in ducks by transcriptome analysis
Source: Anim Biosci. 2025 Aug 12;39(1):250268. doi: 10.5713/ab.25.0268 (PMC12754461; doi:10.5713/ab.25.0268)
Supplement: Supplementary file 5 [file ab-25-0268-Supplementary-5.pdf]

Supplement 5. MCODE information for PPI networks of the IMP-0-vs-IMP-4 group

Betweenness	Closeness	Degree	MCODE_Cluster	MCODE_Node_Status	MCODE_Score	name
406	0.0924731	6	Cluster 4	Seed	2	ACSL5
150	0.0879346	6	Cluster 4	Clustered	1.666666667	DGAT2
150	0.0879346	6	Cluster 4	Clustered	1.666666667	PNPLA2
448	0.0968468	4		Unclustered	0.666666667	FABP7
0	0.0232558	2		Unclustered	1	AKR1D1
0	0.0232558	2		Unclustered	1	SRD5A2
43.24157207	0.1046229	18	Cluster 1	Seed	5	ANGPT2
8.936507937	0.1007026	18	Cluster 2	Clustered	3.733333333	KIT
43.46379429	0.104878	20	Cluster 1	Clustered	4.464285714	VEGFD
45.71379429	0.1056511	22	Cluster 2	Clustered	3.409090909	PDGFB
271.1191705	0.1043689	24	Cluster 1	Clustered	4.761904762	ERBB4
199.8525038	0.1041162	28	Cluster 1	Clustered	4.761904762	MET
8.936507937	0.1004673	16	Cluster 3	Clustered	2.7	FLT4
20.43592314	0.1033654	24	Cluster 2	Seed	3.888888889	KDR
528.9365079	0.1053922	18	Cluster 3	Clustered	2.7	FLT1
8.936507937	0.1004673	16	Cluster 3	Clustered	2.7	TEK
0	0.0232558	2		Unclustered	1	ANGPTL4
0	0.0232558	2		Unclustered	1	PLPPR4
0	0.0893971	2		Unclustered	1	CACNG4
70	0.0964126	4		Unclustered	0.666666667	RASGRP3
2	0.0238095	4		Unclustered	0.666666667	CHKA
0	0.0237963	2		Unclustered	1	ETNPPL
0	0.0237963	2		Unclustered	1	PCYT1B

71.14157207	0.1043689	16	Cluster 3	Clustered	2.7	CSF1
61.25	0.1021378	10		Unclustered	2.7	IL6
263	0.0834951	12		Unclustered	1.066666667	PLPP1
0	0.0912951	4	Cluster 5	Seed	2	DUSP4
266.4	0.0983982	12	Cluster 5	Clustered	2	FOS
0	0.0912951	4	Cluster 5	Clustered	2	NR4A1
0	0.0911017	2		Unclustered	1	DUSP5
71.80823873	0.1046229	18	Cluster 1	Clustered	4	FGF16
43.74157207	0.104878	20	Cluster 1	Clustered	4.285714286	IGF1
43.24157207	0.1041162	14	Cluster 3	Seed	3	NGF
216.0429137	0.1056511	22	Cluster 1	Clustered	4	PIK3R1
3.166666667	0.0975057	6		Unclustered	1.666666667	RHOB
486	0.1011765	4		Unclustered	0.666666667	FABP3
0	0.0911017	2		Unclustered	1	PLCB2
0	0.0783242	4		Unclustered	2	GPD1
1	0.0784672	6		Unclustered	1.666666667	MBOAT1
0	0.0972851	2		Unclustered	1	INPP5D
0	0.0783242	4		Unclustered	2	PLD4
3.634674923	0.1	6		Unclustered	1.666666667	RAC2
0	0.0972851	2		Unclustered	1	SYNJ2
0	0.0781818	2		Unclustered	1	UGT8

Description: Betweenness: Betweenness centrality, is a measure of the intermediary role of a node in the shortest path of the network, nodes with high betweenness centrality play an important role in the communication between different nodes. Closeness: Closeness centrality, measures the average

distance from a node to other nodes, nodes with high closeness centrality are more likely to communicate with other nodes in the network.Degree: The degree of a node. MCODE_Cluster: The Clusters column is an additional list type attribute that indicates which cluster the node belongs to. MCODE_Node_Status: The highest scoring node in the cluster is called the Seed. it is the node from which the cluster was derived. MCODE_Score: The highest scoring node in the cluster is called the Seed.
