# Supplementary material for: Identification of genes and long non-coding RNAs for intramuscular and subcutaneous fat deposition in ducks by transcriptome analysis
Source: Anim Biosci. 2025 Aug 12;39(1):250268. doi: 10.5713/ab.25.0268 (PMC12754461; doi:10.5713/ab.25.0268)
Supplement: Supplementary file 6 [file ab-25-0268-Supplementary-6.pdf]

Supplement 6. MCODE information for PPI networks of the SCP-0-vs-SCP-4 group

Betweenness	Degree	MCODE_Cluster	MCODE_Node_Status	MCODE_Score	name
0	2		Unclustered	1	ST3GAL2
0	2		Unclustered	1	PRKAA2
0	2		Unclustered	1	NFATC2
0	2		Unclustered	1	NFATC1
217.7742202	4		Unclustered	0.666666667	RHOB
0	4		Unclustered	2	RRAS2
0	4		Unclustered	2	SERPINF1
0	4		Unclustered	2	MMP7
133.7934633	8		Unclustered	3	PTGS2
318	6		Unclustered	0.5	SIRT1
0	2		Unclustered	1	HSPA2
0.5	16	Cluster 1	Clustered	6.611111111	WNT7B
0.5	16	Cluster 1	Clustered	6.611111111	WNT2B
0.75	18	Cluster 1	Seed	7.466666667	WNT16
0.75	18	Cluster 1	Clustered	7.466666667	SFRP2
0.5	16	Cluster 1	Clustered	6.611111111	SFRP1
0.5	16	Cluster 1	Clustered	6.611111111	SFRP4
0	2		Unclustered	1	ST8SIA1
0	2		Unclustered	1	FUT9
2	4		Unclustered	0.666666667	FUT7
18.41447972	14	Cluster 4	Clustered	1.928571429	PDGFD
18.41447972	10		Unclustered	0.333333333	IGF2
50.24781305	12	Cluster 4	Clustered	2	PIK3R1

18.41447972	10		Unclustered	0.333333333	NGF
224.8309196	8		Unclustered	1.666666667	TGFB2
0	2		Unclustered	1	KLF2
22.05733686	20	Cluster 6	Seed	4.036363636	IGF1
1536.085747	24	Cluster 1	Clustered	7.466666667	WNT5A
0	2		Unclustered	1	FAT2
2	4		Unclustered	0.666666667	FAT4
0	2		Unclustered	1	FAT1
0	8	Cluster 3	Seed	4	FABP7
0	2		Unclustered	1	SLC27A6
0	8	Cluster 3	Clustered	4	FABP6
37.5177599	8		Unclustered	1.2	MGLL
1.222222222	6		Unclustered	1.666666667	EPHX2
189.7280684	18	Cluster 5	Clustered	3.238095238	PLB1
888.490008	16	Cluster 5	Seed	4	PLA2G4A
71.88094936	14	Cluster 5	Clustered	4	PLA2G12B
0	6		Unclustered	3	ENPP6
5.983333333	4		Unclustered	0.666666667	FADS1
2.666666667	4		Unclustered	0.666666667	ELOVL7
117.9414863	6		Unclustered	0.5	ELOVL5
58.59264069	6	Cluster 7	Clustered	2	NSDHL
0	4	Cluster 7	Clustered	2	DHCR7
67.20307899	18	Cluster 2	Clustered	2.777777778	MBOAT2
35.33085677	16	Cluster 2	Clustered	2.777777778	MBOAT1
1.607142857	10		Unclustered	4	GPD1

760.5879412	24	Cluster 2	Clustered	2.666666667	SELENOI
13.23571429	12	Cluster 3	Clustered	4	LIPC
42.95407149	14		Unclustered	1.607142857	DGKB
254.1044822	18	Cluster 2	Clustered	2.555555556	PLPP4
486.347415	14		Unclustered	3	FADS2
36.73772414	12		Unclustered	2	CSF1
0	2		Unclustered	1	CHSY3
0	2		Unclustered	1	CSPG4
6	4		Unclustered	0.666666667	CHST11
21.08333333	4		Unclustered	0.666666667	PCYT1B
329.3902736	18	Cluster 5	Clustered	3.238095238	PISD
0	2		Unclustered	1	ETNPPL
220.7586081	8		Unclustered	0.4	CHKA
482.6256401	22	Cluster 2	Clustered	2.555555556	PLPP1
0	2		Unclustered	1	SPTLC3
242.1617647	10	Cluster 2	Seed	3	UGT8
0	6	Cluster 2	Clustered	3	SMPD3
160	8	Cluster 2	Clustered	3	CERS6
129.417838	14	Cluster 3	Clustered	4	FABP4
518.2692868	16	Cluster 3	Clustered	4	FABP3
348.6516348	16	Cluster 3	Clustered	4	PNPLA2
18.30662279	10	Cluster 3	Clustered	4	FABP5
356.3849782	16	Cluster 3	Clustered	4	DGAT2
662.1755054	12	Cluster 7	Clustered	2	FAXDC2
1318.119066	16		Unclustered	2.2	CD36

403.841145	6		Unclustered	0.5	GPX3
0	2		Unclustered	1	CAT
0	4	Cluster 8	Seed	2	CACNG3
0	4	Cluster 8	Clustered	2	CACNG4
0	4	Cluster 8	Clustered	2	CACNA1B
1440.341313	22		Unclustered	2.142857143	IL6
744.3649225	20	Cluster 4	Clustered	1.666666667	FGF9
18.41447972	12		Unclustered	1.047619048	EFNA5
687.6717593	18	Cluster 4	Clustered	1.666666667	FGF19
46.76153366	20		Unclustered	3.090909091	BDNF
180.7045083	22	Cluster 1	Clustered	6.611111111	LRP6
180.7045083	22	Cluster 1	Clustered	6.611111111	LRP5
96.88647512	4		Unclustered	0.666666667	SMAD2
25.95450829	20	Cluster 1	Clustered	7.466666667	FZD7
28.5	8		Unclustered	3	BAMBI
10	6		Unclustered	0.5	CSGALNACT1
0	2		Unclustered	1	B3GNT7
30.13181818	26	Cluster 4	Clustered	1.666666667	TEK
943.6290881	30	Cluster 4	Clustered	1.666666667	FLT1
30.13181818	26	Cluster 4	Clustered	1.666666667	FLT4
1307.318331	34		Unclustered	3.047619048	MET
261.1523159	24	Cluster 6	Clustered	3.444444444	FGF2
19.8906702	18	Cluster 6	Clustered	3.555555556	VEGFD
310.1772727	28		Unclustered	1.533333333	PDGFRA
18.41447972	14	Cluster 4	Clustered	1.928571429	ANGPT2

0	2		Unclustered	1	B3GALT1
0	2		Unclustered	1	A4GALT

Description: Betweenness: Betweenness centrality, is a measure of the intermediary role of a node in the shortest path of the network, nodes with high betweenness centrality play an important role in the communication between different nodes. Closeness: Closeness centrality, measures the average distance from a node to other nodes, nodes with high closeness centrality are more likely to communicate with other nodes in the network.Degree: The degree of a node. MCODE_Cluster: The Clusters column is an additional list type attribute that indicates which cluster the node belongs to. MCODE_Node_Status: The highest scoring node in the cluster is called the Seed. it is the node from which the cluster was derived. MCODE_Score: The highest scoring node in the cluster is called the Seed.
