# Supplementary material for: Identification of genes and long non-coding RNAs for intramuscular and subcutaneous fat deposition in ducks by transcriptome analysis
Source: Anim Biosci. 2025 Aug 12;39(1):250268. doi: 10.5713/ab.25.0268 (PMC12754461; doi:10.5713/ab.25.0268)
Supplement: Supplementary file 7 [file ab-25-0268-Supplementary-7.pdf]

Supplement 7. Table of differential lncRNAs analysis in the IMP-0-vs-IMP-4 group results

id	IMP-0_fpkm_mean	IMP-4_fpkm_mean	log2(fc)	PValue	FDR	Symbol	Description
MSTRG.11582.4	0.233333333	5.553333333	4.572889668	2.83E-188	5.35E-185	-	-
XR_003499613.1	0.4	6.9	4.108524457	3.01E-177	3.80E-174	LOC113844759	uncharacterized LOC113844759
XR_003497784.1	3.956666667	0.566666667	-2.803713284	2.70E-159	2.56E-156	LOC113843881	uncharacterized LOC113843881
XR_003495824.1	0.47	5.176666667	3.461290762	2.58E-124	1.96E-121	LOC113842912	uncharacterized LOC113842912
XR_003497399.1	2.066666667	0.15	-3.784271309	5.52E-118	3.48E-115	LOC110352933	uncharacterized LOC110352933
MSTRG.10341.20	3.346666667	0.206666667	-4.017347244	5.39E-113	2.91E-110	-	-
XR_003495823.1	0.633333333	8.266666667	3.706268797	7.94E-97	3.00E-94	LOC113842911	uncharacterized LOC113842911
MSTRG.3256.4	0.003333333	1.786666667	9.06608919	1.35E-94	4.63E-92	-	-
MSTRG.6249.1	1.936666667	0.48	-2.012469352	5.55E-90	1.75E-87	-	-
MSTRG.13036.1	1.883333333	0.176666667	-3.414186603	1.39E-87	3.51E-85	-	-
MSTRG.17438.1	1.503333333	0.173333333	-3.116543905	4.51E-77	9.49E-75	-	-
XR_003501551.1	1.836666667	10.75666667	2.550069354	1.65E-72	3.12E-70	LOC113845772	uncharacterized LOC113845772
XR_003494989.1	15.79	1.083333333	-3.865462049	1.84E-68	3.17E-66	LOC110354512	uncharacterized LOC110354512%2C transcript variant X1
XR_003496855.1	0.213333333	2.053333333	3.266786541	5.15E-68	8.47E-66	LOC113843509	uncharacterized LOC113843509
MSTRG.2341.4	0.406666667	4.8	3.561115759	6.34E-68	9.99E-66	-	-
XR_001187516.3	2.5	10.27	2.038436182	9.71E-68	1.47E-65	LOC106015458	uncharacterized LOC106015458
XR_002402756.2	1.5	0.16	-3.22881869	3.98E-66	5.57E-64	LOC110352934	uncharacterized LOC110352934
XR_003493825.1	8.066666667	0.9	-3.163975735	1.88E-63	2.37E-61	LOC113841183	uncharacterized LOC113841183
MSTRG.13804.1	0.5	2.983333333	2.576925182	9.34E-62	1.10E-59	-	-
XR_003495958.1	0.203333333	4.986666667	4.616157122	3.23E-60	3.59E-58	LOC113843062	uncharacterized LOC113843062
MSTRG.11051.1	0.466666667	2.286666667	2.292781749	4.14E-54	4.24E-52	-	-

MSTRG.10341.22	3.326666667	0.143333333	-4.536631251	4.58E-54	4.57E-52	-	-
MSTRG.6704.4	1.376666667	0.076666667	-4.166436015	1.20E-53	1.17E-51	-	-
XR_217450.4	15.79	2.723333333	-2.535565688	2.96E-49	2.55E-47	LOC101790536	uncharacterized LOC101790536
MSTRG.962.2	6.99	1.43	-2.289277309	6.77E-48	5.45E-46	-	-
MSTRG.852.1	2.48	0.313333333	-2.984569959	1.15E-47	9.05E-46	-	-
MSTRG.7242.2	3	0.35	-3.099535674	2.59E-47	2.00E-45	-	-
MSTRG.5135.3	3.28	0.113333333	-4.855051664	3.59E-47	2.72E-45	-	-
MSTRG.963.2	2.796666667	0.38	-2.879636986	6.26E-47	4.64E-45	-	-
XR_002402751.2	1.813333333	0.193333333	-3.229481846	1.42E-44	9.95E-43	LOC110352931	uncharacterized LOC110352931
MSTRG.961.1	1.82	0.3	-2.600904045	5.69E-41	3.78E-39	-	-
XR_003501238.1	1.69	0.133333333	-3.663913842	6.72E-39	4.39E-37	LOC101798301	uncharacterized LOC101798301%2C transcript variant X3
MSTRG.10341.18	1.36	0.106666667	-3.672425342	1.55E-37	9.60E-36	-	-
MSTRG.10341.19	1.613333333	0.076666667	-4.395301281	3.51E-36	2.04E-34	-	-
XR_003497284.1	2.136666667	0.513333333	-2.057394006	6.73E-34	3.54E-32	LOC106015049	uncharacterized LOC106015049%2C transcript variant X2
MSTRG.17799.1	2.153333333	0.463333333	-2.216449282	8.86E-33	4.53E-31	-	-
MSTRG.9238.5	2.316666667	0.513333333	-2.174082627	1.68E-32	8.47E-31	-	-
MSTRG.6938.4	0.13	1.843333333	3.825733451	3.76E-30	1.72E-28	-	-
XR_002401163.2	2.566666667	0.423333333	-2.600029949	1.60E-28	7.05E-27	LOC110352334	uncharacterized LOC110352334
MSTRG.7242.1	1.326666667	0.106666667	-3.636624621	2.29E-28	9.97E-27	-	-
XR_003496241.1	1.243333333	0.08	-3.95806932	6.54E-28	2.78E-26	LOC110352708	uncharacterized LOC110352708%2C transcript variant X5
XR_003500516.1	0.09	0.993333333	3.464281018	8.30E-28	3.49E-26	LOC101794131	uncharacterized LOC101794131%2C transcript variant X13

MSTRG.17302.1	0.273333333	9.163333333	5.067139187	3.64E-27	1.45E-25	-	-
XR_002402696.2	0.05	0.72	3.847996907	6.82E-26	2.61E-24	LOC106017443	uncharacterized LOC106017443
MSTRG.5363.1	3.603333333	0.103333333	-5.123954497	4.38E-25	1.63E-23	-	-
MSTRG.13035.1	2.036666667	0.113333333	-4.167565729	1.05E-24	3.83E-23	-	-
XR_003499961.1	0.97	0.22	-2.140481224	2.45E-24	8.84E-23	LOC110352806	uncharacterized LOC110352806%2C transcript variant X2
MSTRG.962.1	4.203333333	0.873333333	-2.266929559	2.58E-23	8.88E-22	-	-
MSTRG.14778.1	4.07	0.83	-2.293845553	2.23E-21	6.85E-20	-	-
MSTRG.16632.1	2.676666667	0.366666667	-2.867896464	1.40E-20	4.25E-19	-	-
XR_217889.4	0.32	0.056666667	-2.497499659	1.13E-19	3.23E-18	LOC101792789	uncharacterized LOC101792789
XR_002398922.2	1.716666667	0.08	-4.423466121	1.51E-19	4.29E-18	LOC101798301	uncharacterized LOC101798301%2C transcript variant X4
XR_003497485.1	0.743333333	3.633333333	2.28921252	7.59E-19	2.10E-17	LOC113843765	uncharacterized LOC113843765
MSTRG.15447.1	1.283333333	0.001	-10.32568023	1.39E-18	3.75E-17	-	-
XR_003492471.1	0.016666667	4.483333333	8.071462363	2.77E-18	7.38E-17	LOC113839840	uncharacterized LOC113839840
MSTRG.16415.5	24.64333333	5.08	-2.278297011	4.68E-18	1.22E-16	-	-
MSTRG.5601.1	0.83	0.206666667	-2.005805622	2.29E-17	5.82E-16	-	-
XR_003496279.1	0.046666667	0.283333333	2.602036014	2.39E-17	6.04E-16	LOC101795266	uncharacterized LOC101795266%2C transcript variant X3
XR_003499186.1	0.58	0.063333333	-3.195015982	5.83E-17	1.40E-15	LOC101797796	uncharacterized LOC101797796
XR_001187895.3	0.866666667	0.01	-6.437405312	2.02E-16	4.69E-15	LOC106015692	uncharacterized LOC106015692
XR_003494191.1	1.196666667	0.213333333	-2.487840034	2.91E-16	6.64E-15	LOC113841816	uncharacterized LOC113841816
XR_003496239.1	0.626666667	0.03	-4.38466385	3.85E-16	8.63E-15	LOC110352708	uncharacterized LOC110352708%2C transcript variant X3
MSTRG.16492.1	5.84	1.42	-2.040077439	5.53E-16	1.22E-14	-	-

MSTRG.756.3	0.656666667	0.143333333	-2.195787065	5.84E-16	1.28E-14	-	-
XR_002406604.2	0.001	1.11	10.11634396	7.30E-16	1.58E-14	LOC110354525	uncharacterized LOC110354525
MSTRG.3839.1	0.176666667	0.81	2.196892049	8.01E-16	1.72E-14	-	-
MSTRG.17302.2	0.166666667	7.396666667	5.471837762	2.03E-15	4.25E-14	-	-
MSTRG.17781.1	5.203333333	1.1	-2.241932608	5.54E-15	1.13E-13	-	-
XR_003495821.1	0.163333333	1.523333333	3.221340511	1.05E-14	2.11E-13	LOC113842909	uncharacterized LOC113842909
XR_001188103.2	0.19	0.943333333	2.311768229	1.32E-14	2.65E-13	LOC106015815	uncharacterized LOC106015815
XR_003498451.1	0.016666667	0.626666667	5.232660757	2.75E-14	5.42E-13	LOC106019170	uncharacterized LOC106019170%2C transcript variant X2
MSTRG.14722.2	4.063333333	0.001	-11.988448	3.73E-14	7.27E-13	-	-
MSTRG.17651.1	0.156666667	5.756666667	5.199463516	3.74E-14	7.27E-13	-	-
MSTRG.16636.1	2.686666667	0.62	-2.115477217	4.83E-14	9.28E-13	-	-
XR_002405956.2	0.01	0.283333333	4.824428435	5.82E-14	1.10E-12	LOC106019750	uncharacterized LOC106019750%2C transcript variant X1
XR_003500061.1	0.196666667	0.79	2.0061002	7.51E-14	1.40E-12	LOC101791976	uncharacterized LOC101791976%2C transcript variant X8
XR_003496781.1	0.043333333	0.28	2.691877705	8.13E-14	1.51E-12	LOC113843470	uncharacterized LOC113843470
XR_003500527.1	0.016666667	0.706666667	5.40599236	1.71E-13	3.09E-12	LOC101794131	uncharacterized LOC101794131%2C transcript variant X24
XR_003497955.1	1.25	0.001	-10.28771238	2.11E-13	3.79E-12	LOC113843963	uncharacterized LOC113843963
MSTRG.7331.2	4.253333333	0.89	-2.256716682	2.21E-13	3.94E-12	-	-
XR_003497206.1	0.073333333	0.403333333	2.459431619	3.68E-13	6.42E-12	LOC113843623	uncharacterized LOC113843623
XR_003499074.1	2.433333333	0.416666667	-2.545968369	3.68E-13	6.42E-12	LOC110353950	uncharacterized LOC110353950
MSTRG.8707.1	6.276666667	0.993333333	-2.659648764	9.16E-13	1.56E-11	-	-
MSTRG.16711.1	0.146666667	11.30333333	6.26806345	1.49E-12	2.51E-11	-	-

XR_002402765.2	0.65	0.03	-4.437405312	2.60E-12	4.32E-11	LOC110352941	uncharacterized LOC110352941%2C transcript variant X1
XR_003497180.1	0.001	0.756666667	9.563514081	2.98E-12	4.92E-11	LOC110351679	uncharacterized LOC110351679%2C transcript variant X3
XR_216754.4	0.6	0.066666667	-3.169925001	3.11E-12	5.11E-11	LOC101801306	uncharacterized LOC101801306%2C transcript variant X1
XR_002402646.2	0.483333333	0.073333333	-2.720477471	6.18E-12	1.00E-10	LOC101794014	uncharacterized LOC101794014%2C transcript variant X2
XR_003494086.1	0.226666667	2.94	3.697172004	8.38E-12	1.34E-10	LOC113841618	uncharacterized LOC113841618
MSTRG.10098.1	0.463333333	0.11	-2.074546953	1.56E-11	2.42E-10	-	-
MSTRG.5023.1	0.001	0.756666667	9.563514081	2.52E-11	3.84E-10	-	-
XR_002399433.2	0.713333333	0.173333333	-2.041027268	3.16E-11	4.73E-10	LOC110351689	uncharacterized LOC110351689
MSTRG.6704.2	0.95	0.006666667	-7.154818109	4.80E-11	7.13E-10	-	-
XR_001192206.3	1.196666667	0.25	-2.259021343	7.92E-11	1.16E-09	LOC106018228	uncharacterized LOC106018228
XR_003498687.1	0.52	0.001	-9.022367813	8.36E-11	1.22E-09	LOC106020406	uncharacterized LOC106020406%2C transcript variant X15
XR_003497335.1	0.001	0.43	8.74819285	1.05E-10	1.53E-09	LOC113843664	uncharacterized LOC113843664
MSTRG.3481.1	0.58	3.246666667	2.484834466	1.54E-10	2.18E-09	-	-
XR_003498679.1	0.48	0.001	-8.906890596	1.62E-10	2.27E-09	LOC106020406	uncharacterized LOC106020406%2C transcript variant X3
XR_003495808.1	0.13	0.626666667	2.269186633	1.65E-10	2.31E-09	LOC113842904	uncharacterized LOC113842904
XR_003497162.1	0.356666667	0.046666667	-2.934112064	2.49E-10	3.43E-09	LOC113843598	uncharacterized LOC113843598
XR_002401779.2	0.376666667	0.001	-8.557144557	2.51E-10	3.44E-09	LOC110352593	uncharacterized LOC110352593%2C transcript variant X1
XR_002402718.2	0.656666667	0.001	-9.359017414	2.77E-10	3.78E-09	LOC106017475	uncharacterized LOC106017475%2C transcript variant X2

XR_003498107.1	0.743333333	0.143333333	-2.374635145	4.26E-10	5.74E-09	LOC106017152	uncharacterized LOC106017152%2C transcript variant X1
XR_002406957.2	0.72	0.001	-9.491853096	4.90E-10	6.58E-09	LOC110354729	uncharacterized LOC110354729%2C transcript variant X1
MSTRG.4635.4	1.083333333	0.146666667	-2.884864289	4.96E-10	6.63E-09	-	-
MSTRG.11582.5	0.043333333	0.63	3.861802706	7.44E-10	9.88E-09	-	-
MSTRG.8484.4	1.376666667	0.316666667	-2.120142363	8.59E-10	1.13E-08	-	-
XR_003495715.1	0.006666667	1.013333333	7.247927513	1.66E-09	2.14E-08	LOC113842873	uncharacterized LOC113842873
MSTRG.759.2	0.67	0.03	-4.48112669	1.71E-09	2.20E-08	-	-
XR_001191431.3	2.15	0.086666667	-4.632715632	4.34E-09	5.46E-08	LOC106017766	uncharacterized LOC106017766
XR_002398765.2	0.266666667	1.09	2.031218731	4.59E-09	5.76E-08	LOC106014678	uncharacterized LOC106014678
XR_003498452.1	0.006666667	0.303333333	5.50779464	5.14E-09	6.40E-08	LOC106019170	uncharacterized LOC106019170%2C transcript variant X3
XR_003495273.1	0.636666667	0.113333333	-2.489965987	6.93E-09	8.60E-08	LOC113842682	uncharacterized LOC113842682%2C transcript variant X1
MSTRG.196.1	3.103333333	0.066666667	-5.540709263	7.45E-09	9.19E-08	-	-
XR_002406798.2	0.586666667	0.001	-9.196397213	7.90E-09	9.71E-08	LOC106020406	uncharacterized LOC106020406%2C transcript variant X7
XR_003500126.1	0.663333333	0.1	-2.729734025	1.13E-08	1.38E-07	LOC110352705	uncharacterized LOC110352705%2C transcript variant X4
MSTRG.11648.1	0.001	0.256666667	8.003752135	1.30E-08	1.57E-07	-	-
XR_003494770.1	0.416666667	0.013333333	-4.965784285	1.57E-08	1.88E-07	LOC106018037	uncharacterized LOC106018037%2C transcript variant X2
XR_003494582.1	0.056666667	1.15	4.34298971	1.77E-08	2.11E-07	LOC113842244	uncharacterized LOC113842244
XR_003499424.1	0.17	0.001	-7.409390936	2.03E-08	2.41E-07	LOC110351397	uncharacterized LOC110351397%2C transcript variant X1

XR_003496443.1	0.1	0.46	2.201633861	3.20E-08	3.74E-07	LOC106015994	uncharacterized LOC106015994
MSTRG.10341.21	0.806666667	0.126666667	-2.670935724	3.64E-08	4.24E-07	-	-
XR_003496660.1	0.183333333	0.983333333	2.423211431	4.16E-08	4.83E-07	LOC113843379	uncharacterized LOC113843379
MSTRG.17784.1	6.723333333	1.343333333	-2.32335934	4.24E-08	4.91E-07	-	-
XR_003492257.1	0.033333333	0.25	2.906890596	4.32E-08	4.99E-07	LOC113839689	uncharacterized LOC113839689
XR_217588.4	0.316666667	1.55	2.291231298	5.33E-08	6.09E-07	LOC101794146	uncharacterized LOC101794146
XR_003500594.1	0.306666667	0.043333333	-2.823122238	5.45E-08	6.22E-07	LOC110354183	uncharacterized LOC110354183
XR_003498785.1	0.063333333	0.28	2.144389909	7.65E-08	8.59E-07	LOC113844315	uncharacterized LOC113844315
XR_002405694.2	2.65	0.52	-2.349408831	7.93E-08	8.88E-07	LOC106019569	uncharacterized LOC106019569
MSTRG.12759.1	0.276666667	1.376666667	2.31495854	8.86E-08	9.86E-07	-	-
MSTRG.16489.2	3.32	0.563333333	-2.559122496	9.10E-08	1.01E-06	-	-
XR_003498560.1	3.416666667	0.79	-2.112664945	9.28E-08	1.02E-06	LOC113844194	uncharacterized LOC113844194
XR_003497932.1	0.253333333	0.001	-7.984893108	9.60E-08	1.06E-06	LOC101797051	uncharacterized LOC101797051%2C transcript variant X7
XR_003500112.1	0.27	0.001	-8.076815597	1.02E-07	1.12E-06	LOC106019021	uncharacterized LOC106019021%2C transcript variant X2
XR_003497745.1	0.656666667	0.026666667	-4.622051819	1.09E-07	1.19E-06	LOC110353307	uncharacterized LOC110353307%2C transcript variant X4
XR_003497091.1	0.21	0.001	-7.714245518	1.17E-07	1.27E-06	LOC106017421	uncharacterized LOC106017421%2C transcript variant X5
XR_003494705.1	0.01	0.413333333	5.36923381	3.32E-07	3.44E-06	LOC113842334	uncharacterized LOC113842334
XR_002402025.2	0.54	0.001	-9.076815597	3.52E-07	3.63E-06	LOC106017023	uncharacterized LOC106017023
MSTRG.16401.1	0.09	5.736666667	5.99414388	3.57E-07	3.67E-06	-	-
MSTRG.17303.1	0.001	2.716666667	11.40762184	4.07E-07	4.16E-06	-	-
XR_003495332.1	0.001	0.34	8.409390936	4.20E-07	4.28E-06	LOC101793821	uncharacterized LOC101793821%2C

							transcript variant X6
XR_002402427.2	0.753333333	0.143333333	-2.393914208	4.73E-07	4.77E-06	LOC110352808	uncharacterized LOC110352808
XR_003492805.1	0.16	0.726666667	2.183221824	5.45E-07	5.45E-06	LOC113840090	uncharacterized LOC113840090%2C transcript variant X2
XR_003494917.1	0.001	1.4	10.45121111	5.72E-07	5.68E-06	LOC113842413	uncharacterized LOC113842413
XR_003500005.1	0.493333333	0.033333333	-3.887525271	6.26E-07	6.17E-06	LOC113844968	uncharacterized LOC113844968
XR_001191530.3	0.123333333	0.001	-6.94641896	6.40E-07	6.29E-06	LOC106017832	uncharacterized LOC106017832
MSTRG.17145.1	0.03	2.463333333	6.359505553	6.67E-07	6.53E-06	-	-
XR_001194472.3	0.353333333	0.026666667	-3.727920455	6.71E-07	6.55E-06	LOC106019560	uncharacterized LOC106019560
XR_002400105.2	2.16	0.453333333	-2.252387162	7.16E-07	6.92E-06	LOC110351926	uncharacterized LOC110351926%2C transcript variant X1
XR_003497400.1	0.116666667	0.02	-2.544320516	8.53E-07	8.21E-06	LOC113843702	uncharacterized LOC113843702
XR_001188458.3	0.43	0.001	-8.74819285	1.02E-06	9.63E-06	LOC106016032	uncharacterized LOC106016032%2C transcript variant X2
XR_003497181.1	0.256666667	0.001	-8.003752135	1.04E-06	9.80E-06	LOC110351679	uncharacterized LOC110351679%2C transcript variant X4
XR_003495509.1	0.026666667	0.16	2.584962501	1.12E-06	1.05E-05	LOC101791071	uncharacterized LOC101791071
MSTRG.2608.1	0.363333333	0.006666667	-5.768184325	1.19E-06	1.12E-05	-	-
XR_003492955.1	0.326666667	0.003333333	-6.614709844	1.35E-06	1.25E-05	LOC113840176	uncharacterized LOC113840176%2C transcript variant X2
XR_003501132.1	0.001	0.663333333	9.373590215	1.62E-06	1.48E-05	LOC101802544	uncharacterized LOC101802544%2C transcript variant X8
XR_003494961.1	0.846666667	0.03	-4.818759685	2.34E-06	2.11E-05	LOC113842447	uncharacterized LOC113842447
XR_003499564.1	0.001	0.233333333	7.866248611	2.52E-06	2.27E-05	LOC106017396	uncharacterized LOC106017396%2C transcript variant X7

XR_003499455.1	0.11	0.47	2.095157233	2.85E-06	2.56E-05	LOC113844715	uncharacterized LOC113844715
XR_003494690.1	0.053333333	0.433333333	3.022367813	2.87E-06	2.56E-05	LOC113842327	uncharacterized LOC113842327
XR_001193187.3	0.703333333	0.06	-3.551174187	2.91E-06	2.60E-05	LOC106018810	uncharacterized LOC106018810%2C transcript variant X2
XR_003501006.1	0.4	0.001	-8.64385619	3.27E-06	2.91E-05	LOC113845445	uncharacterized LOC113845445%2C transcript variant X1
XR_003500052.1	0.446666667	0.046666667	-3.258734268	3.59E-06	3.18E-05	LOC113844972	uncharacterized LOC113844972%2C transcript variant X2
XR_003495828.1	0.126666667	0.793333333	2.64689025	3.72E-06	3.28E-05	LOC106016958	uncharacterized LOC106016958
MSTRG.3080.1	0.233333333	0.04	-2.544320516	3.90E-06	3.42E-05	-	-
XR_003492394.1	0.001	0.046666667	5.544320516	3.97E-06	3.48E-05	LOC106017370	uncharacterized LOC106017370%2C transcript variant X9
XR_003493926.1	0.02	0.476666667	4.574908836	4.44E-06	3.85E-05	LOC113841369	uncharacterized LOC113841369%2C transcript variant X6
XR_003497558.1	0.05	0.001	-5.64385619	4.91E-06	4.22E-05	LOC110354397	uncharacterized LOC110354397%2C transcript variant X10
XR_003492849.1	1.116666667	0.15	-2.896164189	5.03E-06	4.30E-05	LOC113840117	uncharacterized LOC113840117
XR_003497632.1	0.12	0.64	2.415037499	5.09E-06	4.35E-05	LOC113843806	uncharacterized LOC113843806
XR_002399473.2	0.086666667	0.003333333	-4.700439718	5.27E-06	4.49E-05	LOC110351709	uncharacterized LOC110351709%2C transcript variant X1
MSTRG.5135.1	1.023333333	0.03	-5.092169844	5.44E-06	4.62E-05	-	-
XR_001188520.3	0.236666667	0.001	-7.886712714	5.59E-06	4.73E-05	LOC101801508	uncharacterized LOC101801508%2C transcript variant X2
XR_003501239.1	0.21	0.001	-7.714245518	6.20E-06	5.23E-05	LOC101798301	uncharacterized LOC101798301%2C transcript variant X5
MSTRG.5643.4	0.503333333	2.296666667	2.189955433	1.02E-05	8.38E-05	-	-

XR_001186984.3	0.56	0.001	-9.129283017	1.04E-05	8.55E-05	LOC106015161	uncharacterized LOC106015161%2C transcript variant X2
XR_003500528.1	0.04	0.496666667	3.63420602	1.09E-05	8.94E-05	LOC101794131	uncharacterized LOC101794131%2C transcript variant X25
XR_003497454.1	2.263333333	0.463333333	-2.288326692	1.13E-05	9.17E-05	LOC113843759	uncharacterized LOC113843759%2C transcript variant X3
XR_003498683.1	3.536666667	0.103333333	-5.097012631	1.31E-05	0.000105928	LOC106020406	uncharacterized LOC106020406%2C transcript variant X10
XR_002401869.2	1.04	0.083333333	-3.641546029	1.51E-05	0.00012131	LOC106016902	uncharacterized LOC106016902
MSTRG.14131.2	0.053333333	0.526666667	3.303780748	1.60E-05	0.000128892	-	-
XR_003497459.1	0.001	0.24	7.906890596	1.83E-05	0.000145517	LOC113843759	uncharacterized LOC113843759%2C transcript variant X8
XR_002402072.2	0.323333333	0.023333333	-3.79255792	1.91E-05	0.000150664	LOC106017003	uncharacterized LOC106017003
XR_001186503.3	0.24	0.001	-7.906890596	1.92E-05	0.000151012	LOC106014868	uncharacterized LOC106014868%2C transcript variant X6
XR_003495617.1	0.593333333	0.016666667	-5.153805336	1.95E-05	0.000153151	LOC106015459	uncharacterized LOC106015459%2C transcript variant X2
XR_003494475.1	1.376666667	0.073333333	-4.230566353	2.22E-05	0.000173949	LOC113842139	uncharacterized LOC113842139
XR_002406033.2	0.18	0.723333333	2.00666373	2.22E-05	0.000173949	LOC106019797	uncharacterized LOC106019797
MSTRG.2606.1	0.59	0.001	-9.204571144	2.25E-05	0.000176152	-	-
MSTRG.16835.1	4.146666667	0.936666667	-2.14634445	2.67E-05	0.000206281	-	-
XR_001191013.3	0.223333333	0.003333333	-6.06608919	2.75E-05	0.000211943	LOC106017519	uncharacterized LOC106017519
XR_003496141.1	0.303333333	0.001	-8.244760234	2.86E-05	0.000218804	LOC113843117	uncharacterized LOC113843117
XR_003494768.1	0.406666667	0.006666667	-5.930737338	2.87E-05	0.000219097	LOC106018037	uncharacterized LOC106018037%2C transcript variant X1
MSTRG.16397.1	0.04	2.25	5.813781191	2.92E-05	0.000222453	-	-

XR_002402795.2	0.26	0.016666667	-3.963474124	3.33E-05	0.000252282	LOC106017517	uncharacterized LOC106017517
XR_003493265.1	0.78	0.053333333	-3.87036472	3.93E-05	0.000295634	LOC113840439	uncharacterized LOC113840439
XR_003493660.1	0.78	0.053333333	-3.87036472	3.93E-05	0.000295634	LOC113841054	uncharacterized LOC113841054
XR_003492617.1	0.04	0.22	2.459431619	3.97E-05	0.000297385	LOC106015201	uncharacterized LOC106015201%2C transcript variant X1
XR_002401132.2	0.266666667	0.026666667	-3.321928095	4.60E-05	0.000342856	LOC110352321	uncharacterized LOC110352321
XR_216772.4	0.31	0.046666667	-2.731803889	4.88E-05	0.000362431	LOC101802284	uncharacterized LOC101802284
XR_003500144.1	0.07	0.633333333	3.177538186	4.96E-05	0.000367393	LOC113845000	uncharacterized LOC113845000%2C transcript variant X2
XR_003498271.1	0.056666667	0.256666667	2.179323699	5.00E-05	0.000369632	LOC113844092	uncharacterized LOC113844092
XR_003499172.1	0.14	0.02	-2.807354922	5.35E-05	0.000394291	LOC106020111	uncharacterized LOC106020111%2C transcript variant X2
XR_003500730.1	0.206666667	0.03	-2.784271309	6.54E-05	0.000474351	LOC113845270	uncharacterized LOC113845270
XR_003496334.1	0.46	0.05	-3.201633861	6.71E-05	0.000484716	LOC113843176	uncharacterized LOC113843176%2C transcript variant X2
XR_003495978.1	0.023333333	0.53	4.505528033	7.00E-05	0.000503361	LOC110352611	uncharacterized LOC110352611%2C transcript variant X1
XR_002406750.2	0.08	0.003333333	-4.584962501	7.26E-05	0.000520385	LOC106020393	uncharacterized LOC106020393%2C transcript variant X1
MSTRG.4652.3	2.07	0.106666667	-4.278449458	7.35E-05	0.000526246	-	-
MSTRG.11866.1	1.476666667	0.123333333	-3.581709523	7.59E-05	0.000542068	-	-
XR_002398926.2	1.926666667	0.326666667	-2.560215838	7.71E-05	0.000547457	LOC101798301	uncharacterized LOC101798301%2C transcript variant X11
XR_003494557.1	0.001	0.42	8.714245518	7.98E-05	0.000565542	LOC113842234	uncharacterized LOC113842234
XR_002404778.2	0.116666667	0.003333333	-5.129283017	8.47E-05	0.000598023	LOC110353790	uncharacterized LOC110353790

XR_001189913.3	0.096666667	0.43	2.15324626	8.57E-05	0.000602746	LOC106016887	uncharacterized LOC106016887
MSTRG.16356.1	0.126666667	3.696666667	4.867116137	8.91E-05	0.000624686	-	-
XR_003498489.1	0.703333333	0.093333333	-2.913744267	9.35E-05	0.000651924	LOC113844166	uncharacterized LOC113844166
XR_003492182.1	0.313333333	0.016666667	-4.232660757	0.000105601	0.000727589	LOC113839646	uncharacterized LOC113839646%2C transcript variant X2
MSTRG.10914.14	1.446666667	0.31	-2.222392421	0.000105726	0.000727589	-	-
XR_003500084.1	0.006666667	0.106666667	4	0.000107377	0.000737611	LOC110352804	uncharacterized LOC110352804%2C transcript variant X1
XR_001186998.3	1.096666667	0.17	-2.689518432	0.00011266	0.000770851	LOC106015168	uncharacterized LOC106015168%2C transcript variant X2
XR_003500518.1	0.001	0.213333333	7.736965594	0.00011582	0.000788453	LOC101794131	uncharacterized LOC101794131%2C transcript variant X15
XR_003496349.1	0.31	0.001	-8.276124405	0.000133884	0.000903302	LOC110351926	uncharacterized LOC110351926%2C transcript variant X2
XR_003494417.1	0.096666667	0.001	-6.594946589	0.000139095	0.000935126	LOC113842123	uncharacterized LOC113842123%2C transcript variant X6
XR_003499823.1	0.006666667	0.133333333	4.321928095	0.000157239	0.0010478	LOC113844856	uncharacterized LOC113844856
XR_003498264.1	0.001	0.04	5.321928095	0.000157596	0.001048333	LOC110354630	uncharacterized LOC110354630%2C transcript variant X2
XR_003497005.1	0.233333333	0.05	-2.222392421	0.000158994	0.00105393	LOC113843560	uncharacterized LOC113843560
MSTRG.10801.5	0.001	0.476666667	8.896836931	0.000176926	0.001164633	-	-
XR_003499893.1	0.001	0.346666667	8.437405312	0.000190074	0.001238259	LOC113844899	uncharacterized LOC113844899
MSTRG.7703.5	0.423333333	0.001	-8.725650281	0.000190972	0.001241975	-	-
XR_003496026.1	0.366666667	0.086666667	-2.080919995	0.000202999	0.00131567	LOC110353088	uncharacterized LOC110353088%2C transcript variant X11
XR_003497435.1	0.67	0.136666667	-2.293499687	0.000208921	0.001349427	LOC106015641	uncharacterized LOC106015641

XR_003496767.1	0.11	0.001	-6.781359714	0.00021854	0.001409152	LOC113843463	uncharacterized LOC113843463
XR_002401835.2	0.106666667	0.001	-6.736965594	0.000221334	0.001424745	LOC110352617	uncharacterized LOC110352617
XR_001194083.3	0.18	0.006666667	-4.754887502	0.000224812	0.001444675	LOC106019339	uncharacterized LOC106019339%2C transcript variant X2
XR_003494226.1	0.001	0.14	7.129283017	0.000249706	0.00159246	LOC101794649	uncharacterized LOC101794649%2C transcript variant X5
XR_001195078.3	0.636666667	0.01	-5.992466327	0.000254606	0.00161692	LOC106019887	uncharacterized LOC106019887
XR_002398969.2	0.1	0.446666667	2.159198595	0.000277021	0.001747541	LOC106014835	uncharacterized LOC106014835
XR_001191984.3	0.12	0.001	-6.906890596	0.000286739	0.001798474	LOC106018106	uncharacterized LOC106018106%2C transcript variant X2
XR_003497832.1	0.1	0.001	-6.64385619	0.000286739	0.001798474	LOC110353203	uncharacterized LOC110353203%2C transcript variant X1
XR_003500051.1	0.466666667	0.06	-2.959358016	0.000286996	0.001798474	LOC113844972	uncharacterized LOC113844972%2C transcript variant X1
XR_003497172.1	0.146666667	0.001	-7.196397213	0.000291088	0.001818101	LOC110351519	uncharacterized LOC110351519%2C transcript variant X1
XR_003494925.1	0.023333333	0.366666667	3.974004791	0.000304591	0.001893064	LOC113842421	uncharacterized LOC113842421%2C transcript variant X3
MSTRG.2264.3	0.923333333	0.026666667	-5.113742166	0.000323035	0.001994593	-	-
XR_003497694.1	0.133333333	0.003333333	-5.321928095	0.000332223	0.002038029	LOC106015249	uncharacterized LOC106015249%2C transcript variant X1
XR_002404468.2	2.153333333	0.246666667	-3.125936989	0.000348422	0.002133943	LOC106018732	uncharacterized LOC106018732%2C transcript variant X2
XR_003496378.1	0.001	0.136666667	7.094517599	0.00035329	0.002160264	LOC106018734	uncharacterized LOC106018734
XR_003494959.1	0.62	0.073333333	-3.079727192	0.000358211	0.002183297	LOC101799909	uncharacterized LOC101799909%2C transcript variant X3

XR_003494823.1	0.043333333	0.001	-5.437405312	0.000365749	0.002222086	LOC101804630	uncharacterized LOC101804630%2C transcript variant X1
XR_003496831.1	0.073333333	0.001	-6.196397213	0.000372052	0.002256756	LOC106016981	uncharacterized LOC106016981%2C transcript variant X10
XR_002401371.2	0.023333333	0.136666667	2.550197083	0.000375932	0.002273007	LOC110352432	uncharacterized LOC110352432
XR_003498756.1	0.043333333	0.006666667	-2.700439718	0.000408084	0.002450625	LOC110354076	uncharacterized LOC110354076%2C transcript variant X2
XR_003499525.1	0.096666667	0.01	-3.273018494	0.000418615	0.002507053	LOC110351396	uncharacterized LOC110351396%2C transcript variant X9
XR_003497554.1	0.056666667	0.001	-5.824428435	0.000423508	0.002530017	LOC110354397	uncharacterized LOC110354397%2C transcript variant X6
XR_002400325.2	0.066666667	0.28	2.070389328	0.000444513	0.002643153	LOC110352013	uncharacterized LOC110352013
XR_003497930.1	0.09	0.001	-6.491853096	0.000444832	0.002643153	LOC101797051	uncharacterized LOC101797051%2C transcript variant X5
XR_003493079.1	0.403333333	0.036666667	-3.459431619	0.000466353	0.002762357	LOC106017632	uncharacterized LOC106017632%2C transcript variant X2
XR_003493828.1	0.376666667	2.173333333	2.528549192	0.000483868	0.002861625	LOC113841195	uncharacterized LOC113841195%2C transcript variant X1
MSTRG.9851.1	0.736666667	0.113333333	-2.700439718	0.000513884	0.003029673	-	-
XR_001194714.3	0.16	0.001	-7.321928095	0.000528831	0.003103296	LOC106019679	uncharacterized LOC106019679%2C transcript variant X1
XR_003494285.1	0.853333333	0.163333333	-2.385290156	0.000540882	0.00316455	LOC113841900	uncharacterized LOC113841900
XR_002399008.2	0.26	0.026666667	-3.285402219	0.000580445	0.00337063	LOC106014868	uncharacterized LOC106014868%2C transcript variant X3
XR_003499259.1	0.163333333	0.013333333	-3.614709844	0.000594252	0.003444476	LOC113844524	uncharacterized LOC113844524%2C transcript variant X1

XR_003496598.1	0.073333333	0.001	-6.196397213	0.000615559	0.003562524	LOC113843320	uncharacterized LOC113843320
XR_003496149.1	0.373333333	0.053333333	-2.807354922	0.000698939	0.004020493	LOC110351821	uncharacterized LOC110351821%2C transcript variant X2
XR_003500510.1	0.046666667	0.293333333	2.652076697	0.000726556	0.004160383	LOC101794131	uncharacterized LOC101794131%2C transcript variant X7
XR_003498399.1	0.05	0.001	-5.64385619	0.000748142	0.004264635	LOC101797653	uncharacterized LOC101797653%2C transcript variant X5
XR_003494957.1	0.843333333	0.11	-2.938599455	0.00075475	0.004290022	LOC101799909	uncharacterized LOC101799909%2C transcript variant X1
XR_001186512.3	0.12	0.003333333	-5.169925001	0.000754863	0.004290022	LOC106014871	uncharacterized LOC106014871
MSTRG.6465.1	0.083333333	0.596666667	2.839959587	0.000777022	0.004409337	-	-
MSTRG.3192.37	0.206666667	0.973333333	2.235628248	0.000792782	0.004478627	-	-
XR_002399744.2	0.713333333	0.156666667	-2.186878135	0.000805569	0.004530577	LOC106015446	uncharacterized LOC106015446%2C transcript variant X2
XR_001187224.3	0.403333333	0.1	-2.011972642	0.000816954	0.004580995	LOC101805445	uncharacterized LOC101805445
XR_003500852.1	0.113333333	0.001	-6.824428435	0.000854163	0.004768444	LOC101796749	uncharacterized LOC101796749%2C transcript variant X12
XR_003492431.1	0.253333333	0.001	-7.984893108	0.000891674	0.00494866	LOC113839830	uncharacterized LOC113839830
XR_003498396.1	0.001	0.046666667	5.544320516	0.000901657	0.004978399	LOC101797653	uncharacterized LOC101797653%2C transcript variant X2
XR_002403457.2	0.71	0.033333333	-4.412781525	0.000902249	0.004978399	LOC106017971	uncharacterized LOC106017971
XR_003499694.1	0.173333333	0.001	-7.437405312	0.000918268	0.005059162	LOC106019671	uncharacterized LOC106019671%2C transcript variant X12
XR_002404413.2	0.07	0.001	-6.129283017	0.000932687	0.005123687	LOC106018692	uncharacterized LOC106018692%2C transcript variant X3
XR_003493668.1	0.001	0.233333333	7.866248611	0.000939573	0.005154037	LOC101804855	uncharacterized LOC101804855%2C

							transcript variant X7
XR_003495202.1	0.133333333	0.01	-3.736965594	0.000945075	0.005176716	LOC113842645	uncharacterized LOC113842645
XR_002402400.2	0.006666667	0.123333333	4.209453366	0.000970854	0.005302573	LOC110352804	uncharacterized LOC110352804%2C transcript variant X8
XR_001189546.3	0.256666667	0.02	-3.68182404	0.001052666	0.005724626	LOC106016672	uncharacterized LOC106016672%2C transcript variant X1
XR_003500758.1	0.001	0.22	7.781359714	0.001117606	0.006035365	LOC113845296	uncharacterized LOC113845296
XR_001194010.2	0.001	0.096666667	6.594946589	0.001117778	0.006035365	LOC106019300	uncharacterized LOC106019300%2C transcript variant X1
XR_003496009.1	0.15	0.003333333	-5.491853096	0.001125208	0.006066826	LOC110352196	uncharacterized LOC110352196%2C transcript variant X1
XR_002402443.2	0.163333333	0.003333333	-5.614709844	0.001162233	0.006239788	LOC110352819	uncharacterized LOC110352819
XR_002401364.2	1.02	0.233333333	-2.128104826	0.001203215	0.006441542	LOC110352428	uncharacterized LOC110352428
MSTRG.8755.1	0.001	0.16	7.321928095	0.001264757	0.006737741	-	-
XR_002398923.2	0.266666667	0.023333333	-3.514573173	0.001265663	0.006737741	LOC101798301	uncharacterized LOC101798301%2C transcript variant X9
XR_003492351.1	0.07	0.006666667	-3.392317423	0.001275641	0.00678132	LOC113839769	uncharacterized LOC113839769
XR_001187592.3	0.363333333	0.056666667	-2.680721484	0.001280964	0.00680007	LOC106015508	uncharacterized LOC106015508%2C transcript variant X2
XR_001187508.3	0.203333333	0.001	-7.667702932	0.001355053	0.007131843	LOC106015454	uncharacterized LOC106015454%2C transcript variant X2
XR_003494462.1	0.07	0.396666667	2.502500341	0.001357739	0.007131843	LOC110351498	uncharacterized LOC110351498
XR_002398562.2	0.24	0.02	-3.584962501	0.001358536	0.007131843	LOC106014525	uncharacterized LOC106014525
XR_003500030.1	0.006666667	0.193333333	4.857980995	0.001386534	0.007268743	LOC113844969	uncharacterized LOC113844969
XR_002399965.2	0.24	0.033333333	-2.847996907	0.001406151	0.007361384	LOC110351914	uncharacterized LOC110351914%2C transcript variant X1

XR_003499148.1	0.153333333	0.001	-7.26052755	0.001409443	0.007368319	LOC113844464	uncharacterized LOC113844464
XR_003500354.1	0.116666667	0.001	-6.866248611	0.001530604	0.007946965	LOC106016023	uncharacterized LOC106016023%2C transcript variant X4
XR_003496090.1	0.12	0.001	-6.906890596	0.001556402	0.00806984	LOC101790664	uncharacterized LOC101790664%2C transcript variant X8
XR_002402340.2	0.39	0.056666667	-2.782901878	0.001648386	0.008477095	LOC110352777	uncharacterized LOC110352777
XR_003496243.1	0.333333333	0.063333333	-2.395928676	0.001703842	0.008689264	LOC110352708	uncharacterized LOC110352708%2C transcript variant X7
XR_003499171.1	0.333333333	0.023333333	-3.836501268	0.001705319	0.008689264	LOC106020111	uncharacterized LOC106020111%2C transcript variant X1
MSTRG.16396.1	0.001	1.39	10.44086917	0.001762481	0.00895435	-	-
XR_002402383.2	0.116666667	0.001	-6.866248611	0.001857886	0.00937613	LOC110352801	uncharacterized LOC110352801
XR_002400010.2	0.02	0.246666667	3.624490865	0.001896418	0.009557846	LOC101795266	uncharacterized LOC101795266%2C transcript variant X7
XR_002403174.2	0.07	0.001	-6.129283017	0.001940714	0.009768087	LOC106017743	uncharacterized LOC106017743%2C transcript variant X2
XR_002400577.2	0.603333333	0.09	-2.744958385	0.001952069	0.009799178	LOC110352126	uncharacterized LOC110352126
XR_003494295.1	1.836666667	0.36	-2.351021006	0.001977854	0.009902353	LOC113841920	uncharacterized LOC113841920%2C transcript variant X1
XR_003497020.1	0.18	0.023333333	-2.94753258	0.002009466	0.01004733	LOC113843562	uncharacterized LOC113843562
XR_003495492.1	0.003333333	0.13	5.285402219	0.002018253	0.010077953	LOC106019145	uncharacterized LOC106019145%2C transcript variant X4
XR_002402014.2	0.106666667	0.001	-6.736965594	0.002084087	0.010365665	LOC101801204	uncharacterized LOC101801204%2C transcript variant X16
XR_003495918.1	0.296666667	0.073333333	-2.016301812	0.002109126	0.010462703	LOC113843035	uncharacterized LOC113843035
XR_002404496.2	0.163333333	0.006666667	-4.614709844	0.002150021	0.010637686	LOC110353703	uncharacterized LOC110353703

XR_002402447.2	0.4	0.096666667	-2.0489096	0.002418674	0.011827752	LOC110352820	uncharacterized LOC110352820%2C transcript variant X2
XR_001186839.3	0.236666667	0.001	-7.886712714	0.002492628	0.012142339	LOC106015067	uncharacterized LOC106015067
XR_001195610.3	0.493333333	0.11	-2.165059246	0.002538633	0.012334695	LOC106020187	uncharacterized LOC106020187%2C transcript variant X2
XR_003497551.1	0.103333333	0.016666667	-2.632268215	0.002547762	0.01236318	LOC110354397	uncharacterized LOC110354397%2C transcript variant X3
XR_002398929.2	0.136666667	0.001	-7.094517599	0.002561866	0.012415701	LOC101798301	uncharacterized LOC101798301%2C transcript variant X13
MSTRG.7239.1	0.053333333	0.01	-2.415037499	0.002643797	0.012780042	-	-
MSTRG.16906.1	0.223333333	2.466666667	3.46529227	0.002671965	0.012899727	-	-
XR_003496379.1	0.03	0.193333333	2.688055994	0.002827165	0.013614274	LOC113843197	uncharacterized LOC113843197
XR_001192879.3	0.293333333	0.016666667	-4.137503524	0.002905088	0.013971739	LOC106018647	uncharacterized LOC106018647
MSTRG.1816.1	0.25	0.013333333	-4.22881869	0.003021638	0.014440531	-	-
XR_003498753.1	0.001	0.123333333	6.94641896	0.003030164	0.014463014	LOC113844290	uncharacterized LOC113844290
XR_003495434.1	0.213333333	0.023333333	-3.192645078	0.003057081	0.014573112	LOC110354348	uncharacterized LOC110354348%2C transcript variant X2
XR_002403838.2	0.26	0.02	-3.700439718	0.003153876	0.014989619	LOC110353403	uncharacterized LOC110353403%2C transcript variant X2
XR_003493662.1	0.206666667	0.001	-7.691161905	0.003162956	0.01500224	LOC101804855	uncharacterized LOC101804855%2C transcript variant X1
XR_002399153.2	0.093333333	0.001	-6.544320516	0.003263325	0.015439608	LOC106014987	uncharacterized LOC106014987%2C transcript variant X1
MSTRG.17271.1	0.09	1.17	3.700439718	0.003334707	0.015757635	-	-
XR_002406163.2	0.193333333	0.001	-7.594946589	0.003463767	0.016306416	LOC110354348	uncharacterized LOC110354348%2C transcript variant X1

XR_003498970.1	0.001	0.106666667	6.736965594	0.00363648	0.017055856	LOC113844388	uncharacterized LOC113844388
XR_001189999.3	0.076666667	0.003333333	-4.523561956	0.003701808	0.017319337	LOC106016924	uncharacterized LOC106016924%2C transcript variant X2
XR_003501003.1	0.206666667	0.036666667	-2.494764692	0.003724487	0.017361062	LOC113845443	uncharacterized LOC113845443
XR_003495374.1	0.73	0.046666667	-3.967432138	0.003729179	0.017361552	LOC113842720	uncharacterized LOC113842720
XR_003495553.1	0.113333333	0.02	-2.502500341	0.003858007	0.017917247	LOC113842799	uncharacterized LOC113842799%2C transcript variant X1
XR_002404562.2	0.27	0.001	-8.076815597	0.003916966	0.018146531	LOC110353729	uncharacterized LOC110353729
XR_003497968.1	0.003333333	0.053333333	4	0.003984614	0.018369991	LOC110351792	uncharacterized LOC110351792
XR_002400348.2	0.13	0.006666667	-4.285402219	0.004167424	0.019166101	LOC110352031	uncharacterized LOC110352031
XR_003496246.1	0.32	0.076666667	-2.061400545	0.004236575	0.019460479	LOC110352708	uncharacterized LOC110352708%2C transcript variant X10
MSTRG.17476.1	3.99	0.973333333	-2.035382878	0.004244104	0.019471436	-	-
XR_003494888.1	0.033333333	0.001	-5.058893689	0.004307235	0.019737148	LOC113842396	uncharacterized LOC113842396
XR_002398455.2	0.066666667	0.01	-2.736965594	0.004344278	0.019858809	LOC106014454	uncharacterized LOC106014454
XR_003500352.1	0.001	0.076666667	6.26052755	0.004365996	0.019934013	LOC106016023	uncharacterized LOC106016023%2C transcript variant X1
MSTRG.9062.2	0.1	0.003333333	-4.906890596	0.004461256	0.020295499	-	-
XR_003494990.1	0.453333333	0.04	-3.502500341	0.0045255	0.020513793	LOC110354512	uncharacterized LOC110354512%2C transcript variant X2
XR_216619.4	0.42	0.001	-8.714245518	0.004541955	0.020550991	LOC101794042	uncharacterized LOC101794042%2C transcript variant X1
XR_003493633.1	0.156666667	0.013333333	-3.554588852	0.004544565	0.020550991	LOC113841023	uncharacterized LOC113841023%2C transcript variant X2
XR_003493521.1	0.393333333	0.056666667	-2.795180208	0.004609902	0.020796758	LOC113840911	uncharacterized LOC113840911

XR_003498423.1	0.001	0.076666667	6.26052755	0.004640026	0.020862056	LOC106017149	uncharacterized LOC106017149%2C transcript variant X1
XR_002399439.2	0.043333333	0.01	-2.115477217	0.004640912	0.020862056	LOC110351692	uncharacterized LOC110351692%2C transcript variant X2
XR_001190983.3	0.103333333	0.003333333	-4.95419631	0.004655056	0.020884544	LOC106017488	uncharacterized LOC106017488
XR_002406329.2	0.16	0.03	-2.415037499	0.0047619	0.021279565	LOC110354414	uncharacterized LOC110354414
XR_002400249.2	0.093333333	0.016666667	-2.485426827	0.004804803	0.021445968	LOC110351990	uncharacterized LOC110351990
XR_003493046.1	0.236666667	0.001	-7.886712714	0.004831655	0.02150728	LOC106019855	uncharacterized LOC106019855%2C transcript variant X2
XR_002399969.2	0.52	0.083333333	-2.641546029	0.004835587	0.02150728	LOC106015640	uncharacterized LOC106015640%2C transcript variant X2
XR_003500530.1	0.001	0.126666667	6.984893108	0.004843809	0.021518566	LOC101794131	uncharacterized LOC101794131%2C transcript variant X27
MSTRG.13035.15	0.983333333	0.163333333	-2.5898613	0.004870474	0.021611657	-	-
XR_003496796.1	0.006666667	0.066666667	3.321928095	0.004947156	0.021926214	LOC110352340	uncharacterized LOC110352340%2C transcript variant X1
XR_002398566.2	0.06	0.001	-5.906890596	0.005014808	0.022200055	LOC110351308	uncharacterized LOC110351308
XR_003495328.1	0.336666667	0.056666667	-2.570748642	0.00506819	0.022410164	LOC101793821	uncharacterized LOC101793821%2C transcript variant X2
XR_003496347.1	0.001	0.043333333	5.437405312	0.005183833	0.022894757	LOC110351925	uncharacterized LOC110351925%2C transcript variant X10
MSTRG.16943.4	4.69	37.67666667	3.006011505	0.005221919	0.023036088	-	-
XR_003492941.1	0.093333333	0.436666667	2.226068079	0.005357485	0.023524454	LOC106017294	uncharacterized LOC106017294
XR_003498455.1	0.001	0.096666667	6.594946589	0.005402471	0.023694498	LOC101790753	uncharacterized LOC101790753%2C transcript variant X3
XR_003492958.1	0.103333333	0.003333333	-4.95419631	0.005537551	0.024230787	LOC113840177	uncharacterized LOC113840177

XR_003495054.1	0.27	0.03	-3.169925001	0.005581886	0.024368439	LOC113842529	uncharacterized LOC113842529
XR_003500508.1	0.001	0.113333333	6.824428435	0.005594455	0.024395174	LOC101794131	uncharacterized LOC101794131%2C transcript variant X5
XR_003495266.1	0.246666667	0.04	-2.624490865	0.005716022	0.024896599	LOC113842678	uncharacterized LOC113842678
XR_003499483.1	0.04	0.001	-5.321928095	0.005755299	0.02503886	LOC106016319	uncharacterized LOC106016319%2C transcript variant X8
XR_003497564.1	0.08	0.016666667	-2.263034406	0.005777415	0.025096982	LOC101802970	uncharacterized LOC101802970%2C transcript variant X2
XR_003500864.1	0.103333333	0.02	-2.36923381	0.005798892	0.025123999	LOC113845332	uncharacterized LOC113845332
XR_002402871.2	0.003333333	0.033333333	3.321928095	0.005931306	0.02556947	LOC106017577	uncharacterized LOC106017577%2C transcript variant X2
XR_003496083.1	0.12	0.001	-6.906890596	0.00602501	0.025943871	LOC101790664	uncharacterized LOC101790664%2C transcript variant X1
XR_003501186.1	0.03	1.216666667	5.341827652	0.006063115	0.026078286	LOC106015252	uncharacterized LOC106015252%2C transcript variant X2
XR_003495212.1	0.5	0.001	-8.965784285	0.006088603	0.026158187	LOC113842655	uncharacterized LOC113842655
XR_003496010.1	0.206666667	0.023333333	-3.146841388	0.006146475	0.02637688	LOC110352196	uncharacterized LOC110352196%2C transcript variant X2
XR_001185635.3	0.153333333	0.006666667	-4.523561956	0.006189119	0.026529804	LOC106014373	uncharacterized LOC106014373%2C transcript variant X2
XR_003492759.1	0.066666667	0.003333333	-4.321928095	0.006269746	0.026784412	LOC106016502	uncharacterized LOC106016502%2C transcript variant X5
XR_002402218.2	0.346666667	0.016666667	-4.378511623	0.006287597	0.026830388	LOC106017053	uncharacterized LOC106017053
XR_003493917.1	2.003333333	0.446666667	-2.16513199	0.006391789	0.027244283	LOC113841351	uncharacterized LOC113841351
XR_003492637.1	0.22	0.001	-7.781359714	0.006419682	0.027332391	LOC113839948	uncharacterized LOC113839948%2C transcript variant X3

XR_003500674.1	0.073333333	0.006666667	-3.459431619	0.00684326	0.028908192	LOC110352821	uncharacterized LOC110352821%2C transcript variant X2
XR_002401572.2	0.05	0.001	-5.64385619	0.007176235	0.030146559	LOC110352524	uncharacterized LOC110352524
XR_002402867.2	0.106666667	0.013333333	-3	0.007422618	0.031078109	LOC110352985	uncharacterized LOC110352985
XR_002403476.2	0.23	0.02	-3.523561956	0.007590616	0.031606692	LOC101799799	uncharacterized LOC101799799%2C transcript variant X6
XR_003495767.1	0.113333333	0.02	-2.502500341	0.007726685	0.032077074	LOC110352593	uncharacterized LOC110352593%2C transcript variant X3
MSTRG.17444.1	1.1	0.263333333	-2.062541466	0.007981696	0.033017181	-	-
XR_003492338.1	0.04	0.246666667	2.624490865	0.008384996	0.034534505	LOC113839747	uncharacterized LOC113839747
MSTRG.2504.2	1.256666667	0.001	-10.29538631	0.00860152	0.03534935	-	-
XR_003494855.1	0.186666667	0.033333333	-2.485426827	0.008676123	0.035549475	LOC110353218	uncharacterized LOC110353218%2C transcript variant X6
XR_003498700.1	0.023333333	0.256666667	3.459431619	0.008758138	0.035760033	LOC113844267	uncharacterized LOC113844267
XR_003500567.1	0.013333333	0.126666667	3.247927513	0.008851432	0.036102013	LOC113845181	uncharacterized LOC113845181
XR_001189264.3	0.003333333	0.03	3.169925001	0.009020646	0.03675258	LOC106016497	uncharacterized LOC106016497
XR_002404190.2	0.001	0.343333333	8.423466121	0.00907257	0.036924382	LOC106018530	uncharacterized LOC106018530%2C transcript variant X2
XR_003495297.1	0.113333333	0.013333333	-3.087462841	0.009199191	0.037359374	LOC106019987	uncharacterized LOC106019987
XR_002404352.2	0.053333333	0.001	-5.736965594	0.009239794	0.03748405	LOC106018650	uncharacterized LOC106018650%2C transcript variant X8
XR_002404953.2	0.053333333	0.003333333	-4	0.009447474	0.038244589	LOC110353849	uncharacterized LOC110353849
XR_002405347.2	0.026666667	0.17	2.672425342	0.009568277	0.03869223	LOC101791673	uncharacterized LOC101791673%2C transcript variant X3
XR_002405232.2	0.16	0.001	-7.321928095	0.009686484	0.03910152	LOC106019236	uncharacterized LOC106019236%2C transcript variant X2

XR_002400836.2	0.103333333	0.003333333	-4.95419631	0.009748115	0.039210006	LOC101804491	uncharacterized LOC101804491%2C transcript variant X2

Description: Sample: id: lncRNA id; IMP0-1_count: lncRNA count value for IMP0-1; IMP0-4_count: lncRNA count value for IMP0-4; log2(FC): logarithmic value of the multiplicity of difference in FPKM between samples IMP0 and IMP4, bottomed by 2; P_value: significance P-value; FDR: P-value after BH correction; Symbol. lncRNA name; Description: lncRNA annotation.
