# Supplementary material for: Identification of genes and long non-coding RNAs for intramuscular and subcutaneous fat deposition in ducks by transcriptome analysis
Source: Anim Biosci. 2025 Aug 12;39(1):250268. doi: 10.5713/ab.25.0268 (PMC12754461; doi:10.5713/ab.25.0268)
Supplement: Supplementary file 8 [file ab-25-0268-Supplementary-8.pdf]

Supplement 8. Table of differential lncRNAs analysis in the SCP-0-vs-SCP-4 group results

id	SCP-0_fpkm_mean	SCP-4_fpkm_mean	log2(fc)	PValue	FDR	Symbol	Description
MSTRG.13544.2	20.08333333	3.59	-2.483942991	0	0	-	-
MSTRG.2333.18	1.18	5.676666667	2.26625717	0	0	-	-
MSTRG.5135.3	14.28666667	1.083333333	-3.721120227	0	0	-	-
MSTRG.5641.4	0.193333333	2.233333333	3.53003629	0	0	-	-
MSTRG.8652.4	11.75	179.59	3.933974358	0	0	-	-
MSTRG.881.1	5.696666667	0.62	-3.199777871	0	0	-	-
MSTRG.4618.1	0.406666667	2.426666667	2.577057303	1.10E-249	5.94E-247	-	-
MSTRG.6704.4	10.95333333	0.06	-7.512191764	1.05E-216	5.03E-214	-	-
MSTRG.8536.1	1.673333333	0.263333333	-2.667762806	4.38E-204	1.90E-201	-	-
MSTRG.756.3	2.8	0.08	-5.129283017	1.66E-199	6.53E-197	-	-
MSTRG.3256.4	0.016666667	2.41	7.175923742	3.79E-189	1.37E-186	-	-
MSTRG.852.1	7.226666667	0.243333333	-4.892324482	5.58E-185	1.86E-182	-	-
MSTRG.9008.1	2.626666667	0.233333333	-3.492768803	4.91E-160	1.33E-157	-	-
MSTRG.7949.1	2.103333333	0.14	-3.909178772	9.80E-153	2.36E-150	-	-
MSTRG.6316.1	1.196666667	9.686666667	3.016978954	1.32E-151	3.00E-149	-	-
MSTRG.4704.1	15.99666667	3.52	-2.184123978	2.97E-149	6.44E-147	-	-
MSTRG.7239.1	2.883333333	0.026666667	-6.756556323	1.36E-137	2.67E-135	-	-
MSTRG.582.1	6.56	0.54	-3.602664502	2.13E-137	4.01E-135	-	-
XR_003495824.1	0.116666667	5.85	5.647972298	2.15E-134	3.87E-132	LOC113842912	uncharacterized LOC113842912
XR_003495823.1	0.226666667	9.166666667	5.337753062	3.34E-125	5.78E-123	LOC113842911	uncharacterized LOC113842911
MSTRG.7954.1	2.113333333	0.123333333	-4.098885665	2.56E-123	4.26E-121	-	-
MSTRG.3622.7	4.273333333	0.676666667	-2.658844629	7.93E-117	1.27E-114	-	-
MSTRG.885.1	4.396666667	0.32	-3.780266349	1.11E-116	1.71E-114	-	-

MSTRG.2265.1	0.823333333	5.51	2.742503778	1.61E-115	2.40E-113	-	-
MSTRG.7991.2	1.006666667	5.813333333	2.529779585	1.07E-113	1.55E-111	-	-
MSTRG.11051.1	0.95	4.38	2.204931451	1.36E-107	1.84E-105	-	-
MSTRG.8558.1	0.863333333	5.326666667	2.625243405	1.53E-104	1.95E-102	-	-
MSTRG.6424.1	2.376666667	0.253333333	-3.229830753	5.80E-99	7.17E-97	-	-
MSTRG.13036.1	2.043333333	0.203333333	-3.329005926	8.88E-99	1.07E-96	-	-
MSTRG.5997.1	4.216666667	0.523333333	-3.010300921	2.53E-97	2.96E-95	-	-
MSTRG.7248.1	1.883333333	0.106666667	-4.142107057	2.40E-94	2.73E-92	-	-
MSTRG.10341.18	4.29	0.056666667	-6.242333497	1.18E-91	1.31E-89	-	-
XR_003498491.1	3.853333333	0.286666667	-3.748660928	1.41E-89	1.53E-87	LOC113844169	uncharacterized LOC113844169
MSTRG.10341.20	15.43666667	0.09	-7.42222054	3.61E-86	3.55E-84	-	-
XR_003494957.1	7.016666667	0.43	-4.028377263	7.86E-86	7.39E-84	LOC101799909	uncharacterized LOC101799909%2C transcript variant X1
MSTRG.16118.5	31.81333333	5.506666667	-2.530380356	4.41E-82	4.06E-80	-	-
MSTRG.16051.2	13.05333333	1.94	-2.750289707	1.79E-77	1.55E-75	-	-
XR_003497566.1	0.97	0.06	-4.014950341	2.63E-77	2.23E-75	LOC101802970	uncharacterized LOC101802970%2C transcript variant X4
MSTRG.10914.13	3.78	0.52	-2.861802706	5.17E-77	4.23E-75	-	-
MSTRG.1418.1	2.313333333	0.29	-2.995848357	5.83E-74	4.68E-72	-	-
MSTRG.5644.1	0.123333333	1.256666667	3.348967348	2.53E-72	1.99E-70	-	-
MSTRG.3080.1	3.126666667	0.133333333	-4.551516018	1.28E-70	9.88E-69	-	-
XR_002406750.2	1.736666667	0.016666667	-6.703211467	2.13E-68	1.62E-66	LOC106020393	uncharacterized LOC106020393%2C transcript variant X1
MSTRG.6127.1	2.253333333	0.06	-5.230954435	2.57E-66	1.92E-64	-	-
XR_003492471.1	0.31	8.126666667	4.7123236	5.64E-65	4.01E-63	LOC113839840	uncharacterized LOC113839840

MSTRG.338.3	2.363333333	0.503333333	-2.231237078	4.03E-64	2.77E-62	-	-
MSTRG.2763.2	1.66	0.2	-3.053111336	4.43E-62	3.00E-60	-	-
MSTRG.10010.2	0.01	0.686666667	6.101538026	5.78E-62	3.85E-60	-	-
MSTRG.5881.2	1.97	0.236666667	-3.057267201	1.79E-61	1.18E-59	-	-
MSTRG.9637.1	1.166666667	6.726666667	2.527499347	2.65E-60	1.69E-58	-	-
XR_003497399.1	0.953333333	0.03	-4.989946335	3.33E-58	2.03E-56	LOC110352933	uncharacterized LOC110352933
MSTRG.13197.1	0.296666667	5.8	4.28913816	8.27E-58	4.97E-56	-	-
XR_001186427.3	2.063333333	0.32	-2.688833098	4.78E-55	2.72E-53	LOC106014827	uncharacterized LOC106014827
MSTRG.15586.1	7.653333333	36.68333333	2.260964598	9.66E-54	5.43E-52	-	-
XR_003501281.1	2.16	0.47	-2.20029865	4.46E-53	2.46E-51	LOC106015373	uncharacterized LOC106015373%2C transcript variant X2
XR_003497296.1	3.753333333	0.083333333	-5.493134922	4.49E-53	2.46E-51	LOC113843645	uncharacterized LOC113843645
XR_003497551.1	0.72	0.046666667	-3.94753258	8.64E-52	4.67E-50	LOC110354397	uncharacterized LOC110354397%2C transcript variant X3
MSTRG.582.3	1.973333333	0.123333333	-4	2.20E-51	1.18E-49	-	-
XR_003496313.1	0.18	1.176666667	2.708636871	1.92E-50	9.88E-49	LOC106015647	uncharacterized LOC106015647%2C transcript variant X33
MSTRG.2904.1	2.326666667	0.143333333	-4.020818472	5.46E-49	2.72E-47	-	-
XR_003497578.1	1.563333333	12.88	3.042435266	8.13E-49	4.00E-47	LOC113843791	uncharacterized LOC113843791
MSTRG.7345.1	0.45	2.34	2.378511623	1.07E-47	5.22E-46	-	-
XR_003494961.1	5.946666667	0.163333333	-5.186190056	5.30E-46	2.52E-44	LOC113842447	uncharacterized LOC113842447
XR_003492939.1	0.063333333	3	5.565853678	3.38E-45	1.59E-43	LOC101799584	uncharacterized LOC101799584%2C transcript variant X2
XR_003496983.1	1.063333333	0.16	-2.732450113	4.00E-44	1.82E-42	LOC113843553	uncharacterized LOC113843553
XR_003496370.1	2.216666667	0.226666667	-3.289747689	7.21E-44	3.25E-42	LOC106017302	uncharacterized LOC106017302%2C

							transcript variant X1
MSTRG.9062.2	2.626666667	0.083333333	-4.97819563	3.07E-43	1.37E-41	-	-
XR_001194132.2	2.076666667	0.12	-4.113163352	3.51E-42	1.53E-40	LOC106019368	uncharacterized LOC106019368
MSTRG.965.12	5.58	0.37	-3.914667946	4.01E-42	1.74E-40	-	-
MSTRG.8538.1	1.596666667	0.396666667	-2.009064082	3.17E-40	1.32E-38	-	-
XR_003497001.1	1.796666667	0.213333333	-3.074141463	1.23E-37	4.85E-36	LOC106017547	uncharacterized LOC106017547%2C transcript variant X1
XR_003501238.1	1.833333333	0.233333333	-2.974004791	5.14E-37	1.97E-35	LOC101798301	uncharacterized LOC101798301%2C transcript variant X3
XR_003499677.1	0.126666667	0.693333333	2.452512205	8.26E-37	3.14E-35	LOC101796143	uncharacterized LOC101796143%2C transcript variant X9
XR_003500680.1	0.35	0.03	-3.544320516	1.30E-36	4.80E-35	LOC110352551	uncharacterized LOC110352551
XR_001188103.2	0.133333333	1.576666667	3.563768278	1.36E-36	5.00E-35	LOC106015815	uncharacterized LOC106015815
MSTRG.3639.1	1.33	0.073333333	-4.180813318	2.64E-35	9.31E-34	-	-
MSTRG.10900.2	1.256666667	5.123333333	2.027480736	3.64E-35	1.27E-33	-	-
XR_002402756.2	0.71	0.03	-4.564784619	4.25E-35	1.47E-33	LOC110352934	uncharacterized LOC110352934
MSTRG.7978.1	2.14	8.986666667	2.070175294	4.82E-35	1.65E-33	-	-
MSTRG.5592.6	6.203333333	0.493333333	-3.652408974	1.39E-34	4.74E-33	-	-
MSTRG.965.13	2.416666667	0.25	-3.273018494	5.21E-34	1.75E-32	-	-
XR_003497784.1	0.673333333	0.096666667	-2.800230488	1.80E-33	5.90E-32	LOC113843881	uncharacterized LOC113843881
MSTRG.5011.1	2.48	0.576666667	-2.104530583	7.28E-33	2.37E-31	-	-
XR_003492840.1	0.113333333	0.783333333	2.789054105	1.02E-32	3.28E-31	LOC113840110	uncharacterized LOC113840110%2C transcript variant X1
MSTRG.9838.2	28.96	6.776666667	-2.095413888	1.42E-32	4.52E-31	-	-
XR_001188444.3	1.233333333	0.126666667	-3.283453947	3.56E-32	1.12E-30	LOC106016033	uncharacterized LOC106016033

MSTRG.12635.13	0.74	9.323333333	3.655248672	9.43E-32	2.90E-30	-	-
MSTRG.17409.2	0.196666667	3.933333333	4.321928095	1.07E-31	3.27E-30	-	-
XR_003499063.1	0.33	0.053333333	-2.62935662	1.13E-31	3.43E-30	LOC110353608	uncharacterized LOC110353608
MSTRG.10914.14	2.8	0.273333333	-3.356693513	2.77E-31	8.16E-30	-	-
XR_002402646.2	1.403333333	0.073333333	-4.258244804	2.84E-31	8.30E-30	LOC101794014	uncharacterized LOC101794014%2C transcript variant X2
MSTRG.10098.1	1.343333333	0.17	-2.982210687	2.94E-31	8.55E-30	-	-
XR_002400646.2	1.64	0.01	-7.357552005	2.32E-30	6.65E-29	LOC110352151	uncharacterized LOC110352151%2C transcript variant X1
XR_001191423.3	2.233333333	0.496666667	-2.168848765	5.94E-30	1.68E-28	LOC106017763	uncharacterized LOC106017763
XR_217454.4	0.503333333	0.026666667	-4.238404739	1.06E-29	2.99E-28	LOC101799933	uncharacterized LOC101799933
MSTRG.2608.1	1.773333333	0.073333333	-4.595850817	5.62E-29	1.56E-27	-	-
XR_003497661.1	2.936666667	0.65	-2.175667895	1.13E-28	3.11E-27	LOC101798800	uncharacterized LOC101798800
XR_003499966.1	1.59	0.173333333	-3.197405738	1.28E-28	3.52E-27	LOC110352806	uncharacterized LOC110352806%2C transcript variant X7
MSTRG.6704.2	5.866666667	0.01	-9.196397213	2.20E-28	5.94E-27	-	-
XR_002402751.2	1.043333333	0.03	-5.120093845	4.18E-28	1.12E-26	LOC110352931	uncharacterized LOC110352931
XR_001189917.2	19.74	0.243333333	-6.342044216	6.68E-28	1.79E-26	LOC106016889	uncharacterized LOC106016889%2C transcript variant X2
MSTRG.6704.3	8.083333333	0.016666667	-8.921840937	2.30E-27	6.07E-26	-	-
MSTRG.8113.1	1.246666667	0.096666667	-3.688913465	9.92E-27	2.60E-25	-	-
MSTRG.5135.1	4.95	0.47	-3.396695863	2.46E-26	6.34E-25	-	-
XR_001187862.3	4.026666667	0.22	-4.19401062	5.23E-26	1.32E-24	LOC106015672	uncharacterized LOC106015672%2C transcript variant X1
XR_003497767.1	0.733333333	3.12	2.089005006	8.31E-26	2.08E-24	LOC113843877	uncharacterized LOC113843877

MSTRG.17408.1	0.106666667	3.586666667	5.071462363	8.89E-26	2.20E-24	-	-
XR_003494664.1	1.896666667	0.13	-3.866882623	2.35E-25	5.74E-24	LOC113842319	uncharacterized LOC113842319
MSTRG.13538.3	1.016666667	0.226666667	-2.165202591	2.36E-25	5.74E-24	-	-
XR_002398922.2	1.66	0.223333333	-2.893912742	2.77E-25	6.69E-24	LOC101798301	uncharacterized LOC101798301%2C transcript variant X4
XR_003499961.1	2.443333333	0.186666667	-3.710314466	4.65E-24	1.08E-22	LOC110352806	uncharacterized LOC110352806%2C transcript variant X2
XR_003497547.1	0.03	0.266666667	3.152003093	5.30E-24	1.21E-22	LOC110352760	uncharacterized LOC110352760%2C transcript variant X1
XR_002402718.2	9.326666667	0.001	-13.18714584	1.06E-23	2.42E-22	LOC106017475	uncharacterized LOC106017475%2C transcript variant X2
XR_003497485.1	0.496666667	3.136666667	2.658882392	1.81E-23	4.11E-22	LOC113843765	uncharacterized LOC113843765
XR_003496241.1	1.276666667	0.116666667	-3.451917565	2.55E-23	5.74E-22	LOC110352708	uncharacterized LOC110352708%2C transcript variant X5
MSTRG.8755.1	0.1	2.236666667	4.483278361	2.70E-23	6.07E-22	-	-
XR_003494888.1	0.62	0.006666667	-6.539158811	3.43E-23	7.62E-22	LOC113842396	uncharacterized LOC113842396
XR_002405843.2	1.37	0.14	-3.290677161	5.01E-23	1.11E-21	LOC106019674	uncharacterized LOC106019674
MSTRG.10290.15	0.366666667	1.57	2.098223536	5.89E-23	1.29E-21	-	-
MSTRG.5023.2	0.27	1.48	2.454565863	8.70E-23	1.87E-21	-	-
MSTRG.10341.19	8.243333333	0.076666667	-6.748484568	1.49E-22	3.20E-21	-	-
XR_003499790.1	0.683333333	0.08	-3.094517599	1.54E-22	3.29E-21	LOC101804048	uncharacterized LOC101804048
MSTRG.16028.1	1.436666667	0.18	-2.996656557	2.30E-22	4.86E-21	-	-
XR_002401163.2	1.436666667	0.043333333	-5.051104341	2.40E-22	5.04E-21	LOC110352334	uncharacterized LOC110352334
XR_003500366.1	1.81	0.146666667	-3.625376769	3.13E-22	6.54E-21	LOC110351294	uncharacterized LOC110351294%2C transcript variant X1

XR_217889.4	0.343333333	0.07	-2.294183104	3.75E-22	7.80E-21	LOC101792789	uncharacterized LOC101792789
MSTRG.8490.1	1.766666667	0.313333333	-2.495259698	6.66E-22	1.37E-20	-	-
MSTRG.11490.9	1.343333333	5.513333333	2.037107491	8.01E-22	1.63E-20	-	-
MSTRG.4635.4	1.986666667	0.073333333	-4.759736902	1.17E-21	2.35E-20	-	-
XR_003496334.1	1.96	0.02	-6.614709844	2.32E-21	4.65E-20	LOC113843176	uncharacterized LOC113843176%2C transcript variant X2
XR_003496239.1	0.56	0.03	-4.222392421	2.52E-21	5.00E-20	LOC110352708	uncharacterized LOC110352708%2C transcript variant X3
MSTRG.13035.1	2.42	0.27	-3.163975735	2.82E-21	5.56E-20	-	-
XR_003494663.1	0.78	0.09	-3.115477217	1.35E-20	2.61E-19	LOC113842318	uncharacterized LOC113842318
XR_003499720.1	1.583333333	0.07	-4.49946628	2.73E-20	5.27E-19	LOC113844791	uncharacterized LOC113844791%2C transcript variant X1
XR_002402594.2	1.42	0.19	-2.901819606	1.64E-19	3.12E-18	LOC110352887	uncharacterized LOC110352887
MSTRG.7242.2	0.533333333	0.003333333	-7.321928095	3.96E-19	7.35E-18	-	-
XR_002401866.2	1.64	0.096666667	-4.08453351	6.86E-19	1.27E-17	LOC106016894	uncharacterized LOC106016894
XR_003493063.1	0.04	1.81	5.499845887	1.58E-18	2.89E-17	LOC113840289	uncharacterized LOC113840289
XR_001185593.3	0.08	0.94	3.554588852	1.99E-18	3.62E-17	LOC106014339	uncharacterized LOC106014339
XR_002401875.2	1.166666667	0.106666667	-3.451211112	2.03E-18	3.67E-17	LOC110352627	uncharacterized LOC110352627
MSTRG.4893.1	2.843333333	0.023333333	-6.929047009	2.05E-18	3.70E-17	-	-
MSTRG.962.2	0.696666667	0.013333333	-5.707359132	2.21E-18	3.96E-17	-	-
XR_003497032.1	0.813333333	0.063333333	-3.682809824	3.55E-18	6.25E-17	LOC106020477	uncharacterized LOC106020477%2C transcript variant X5
XR_002402719.2	0.23	0.02	-3.523561956	4.81E-18	8.42E-17	LOC106017476	uncharacterized LOC106017476
XR_003497330.1	0.07	0.88	3.652076697	8.21E-18	1.43E-16	LOC110352095	uncharacterized LOC110352095%2C transcript variant X3

XR_003497564.1	0.35	0.001	-8.451211112	1.01E-17	1.75E-16	LOC101802970	uncharacterized LOC101802970%2C transcript variant X2
XR_003497745.1	1.786666667	0.033333333	-5.744161096	1.05E-17	1.81E-16	LOC110353307	uncharacterized LOC110353307%2C transcript variant X4
XR_001189941.3	4.183333333	0.001	-12.03043724	1.18E-17	2.03E-16	LOC106016901	uncharacterized LOC106016901
MSTRG.2341.4	0.006666667	2.85	8.73978061	1.48E-17	2.52E-16	-	-
XR_003492980.1	0.166666667	2.94	4.140778656	1.58E-17	2.69E-16	LOC113840212	uncharacterized LOC113840212
XR_003496343.1	0.753333333	0.001	-9.557144557	2.18E-17	3.66E-16	LOC110351925	uncharacterized LOC110351925%2C transcript variant X6
XR_003493825.1	6.303333333	0.003333333	-10.88493365	3.73E-17	6.24E-16	LOC113841183	uncharacterized LOC113841183
XR_003500301.1	0.496666667	0.003333333	-7.21916852	4.80E-17	7.97E-16	LOC113845068	uncharacterized LOC113845068
MSTRG.13915.1	2.9	0.001	-11.50183718	5.39E-17	8.90E-16	-	-
XR_003495958.1	0.093333333	1.343333333	3.847281106	6.42E-17	1.06E-15	LOC113843062	uncharacterized LOC113843062
XR_002399173.2	1.416666667	0.001	-10.46828463	1.20E-16	1.95E-15	LOC110351568	uncharacterized LOC110351568%2C transcript variant X2
XR_003494695.1	0.066666667	0.37	2.472487771	2.23E-16	3.57E-15	LOC113842331	uncharacterized LOC113842331
XR_003496445.1	0.136666667	4.016666667	4.877265426	2.82E-16	4.48E-15	LOC113843226	uncharacterized LOC113843226
MSTRG.4591.6	0.001	0.936666667	9.871391914	4.04E-16	6.38E-15	-	-
XR_002403094.2	0.613333333	0.133333333	-2.201633861	4.73E-16	7.42E-15	LOC110353080	uncharacterized LOC110353080
XR_001187947.2	0.58	0.046666667	-3.635588574	6.50E-16	1.01E-14	LOC106015729	uncharacterized LOC106015729
XR_002406489.2	2.223333333	0.396666667	-2.486725188	9.56E-16	1.48E-14	LOC106020129	uncharacterized LOC106020129%2C transcript variant X2
XR_003495828.1	0.016666667	2.18	7.031218731	9.58E-16	1.48E-14	LOC106016958	uncharacterized LOC106016958
XR_001193187.3	0.93	0.033333333	-4.802193217	1.46E-15	2.23E-14	LOC106018810	uncharacterized LOC106018810%2C transcript variant X2

MSTRG.10100.1	2.143333333	0.306666667	-2.805112971	1.91E-15	2.90E-14	-	-
XR_003495715.1	2.586666667	0.001	-11.33687844	2.27E-15	3.42E-14	LOC113842873	uncharacterized LOC113842873
XR_003500794.1	0.173333333	1.443333333	3.057783497	2.42E-15	3.64E-14	LOC113845310	uncharacterized LOC113845310
XR_002399732.1	3.016666667	0.003333333	-9.821773982	3.60E-15	5.38E-14	LOC110351814	uncharacterized LOC110351814
MSTRG.715.1	0.96	0.001	-9.906890596	4.91E-15	7.25E-14	-	-
MSTRG.10373.2	0.206666667	1.3	2.653134003	5.72E-15	8.42E-14	-	-
XR_003501158.1	0.59	2.48	2.071553261	6.58E-15	9.62E-14	LOC113845530	uncharacterized LOC113845530
XR_003497002.1	1.066666667	0.223333333	-2.255838904	1.08E-14	1.57E-13	LOC106017547	uncharacterized LOC106017547%2C transcript variant X2
MSTRG.16568.1	1.066666667	0.21	-2.344648171	1.32E-14	1.90E-13	-	-
XR_003492182.1	0.88	0.033333333	-4.722466024	1.34E-14	1.92E-13	LOC113839646	uncharacterized LOC113839646%2C transcript variant X2
XR_003497095.1	2.196666667	0.036666667	-5.904703036	1.37E-14	1.96E-13	LOC106015748	uncharacterized LOC106015748
XR_003496440.1	2.56	0.033333333	-6.263034406	2.81E-14	4.01E-13	LOC106015283	uncharacterized LOC106015283%2C transcript variant X2
XR_002402717.2	1.47	0.001	-10.52160044	3.99E-14	5.65E-13	LOC106017475	uncharacterized LOC106017475%2C transcript variant X1
XR_002399836.2	0.293333333	0.05	-2.552541023	4.62E-14	6.51E-13	LOC106015519	uncharacterized LOC106015519
XR_003493502.1	6.656666667	0.001	-12.70058421	7.13E-14	9.96E-13	LOC113840880	uncharacterized LOC113840880
XR_001186359.3	1.093333333	0.026666667	-5.357552005	7.43E-14	1.03E-12	LOC101798301	uncharacterized LOC101798301%2C transcript variant X7
MSTRG.3249.1	4.046666667	0.446666667	-3.179463516	8.07E-14	1.12E-12	-	-
XR_003497554.1	0.27	0.003333333	-6.339850003	1.04E-13	1.43E-12	LOC110354397	uncharacterized LOC110354397%2C transcript variant X6
XR_003495207.1	1.683333333	0.263333333	-2.676358829	1.10E-13	1.51E-12	LOC113842649	uncharacterized LOC113842649

XR_003501474.1	0.97	0.05	-4.277984747	1.31E-13	1.78E-12	LOC106019132	uncharacterized LOC106019132%2C transcript variant X1
XR_002405535.2	0.52	0.001	-9.022367813	1.37E-13	1.87E-12	LOC110354079	uncharacterized LOC110354079
XR_003496449.1	0.27	2.276666667	3.075891765	1.92E-13	2.59E-12	LOC113843230	uncharacterized LOC113843230
XR_003494960.1	1.053333333	0.001	-10.04074634	2.14E-13	2.88E-12	LOC101799909	uncharacterized LOC101799909%2C transcript variant X4
XR_003492236.1	0.353333333	0.046666667	-2.920565533	2.33E-13	3.12E-12	LOC113839675	uncharacterized LOC113839675
XR_003499823.1	0.056666667	0.41	2.855051664	2.97E-13	3.97E-12	LOC113844856	uncharacterized LOC113844856
MSTRG.13544.1	1.52	0.001	-10.56985561	3.17E-13	4.19E-12	-	-
MSTRG.10010.1	0.01	0.186666667	4.222392421	4.01E-13	5.28E-12	-	-
XR_003500566.1	0.123333333	0.001	-6.94641896	4.17E-13	5.45E-12	LOC113845180	uncharacterized LOC113845180
XR_002402190.2	0.526666667	0.103333333	-2.349584438	5.68E-13	7.41E-12	LOC101798653	uncharacterized LOC101798653%2C transcript variant X2
XR_002398903.2	0.413333333	1.86	2.169925001	7.79E-13	1.01E-11	LOC110351443	uncharacterized LOC110351443
MSTRG.759.2	0.956666667	0.15	-2.67305383	8.08E-13	1.03E-11	-	-
XR_003495635.1	0.686666667	0.043333333	-3.986060809	8.87E-13	1.13E-11	LOC113842831	uncharacterized LOC113842831
XR_003493773.1	2.57	0.001	-11.32755264	1.47E-12	1.84E-11	LOC113841129	uncharacterized LOC113841129
MSTRG.2606.1	2.296666667	0.106666667	-4.428360173	1.72E-12	2.15E-11	-	-
MSTRG.1816.1	1.51	0.09	-4.068479738	1.83E-12	2.29E-11	-	-
XR_003497162.1	0.523333333	0.113333333	-2.207157908	1.95E-12	2.43E-11	LOC113843598	uncharacterized LOC113843598
XR_003492940.1	0.001	0.773333333	9.594946589	2.62E-12	3.25E-11	LOC101799584	uncharacterized LOC101799584%2C transcript variant X3
MSTRG.889.1	1.556666667	0.053333333	-4.86727874	3.10E-12	3.83E-11	-	-
XR_003493501.1	4.073333333	0.001	-11.99199416	3.17E-12	3.90E-11	LOC113840879	uncharacterized LOC113840879
MSTRG.16804.1	2.12	0.001	-11.04984855	3.40E-12	4.17E-11	-	-

XR_003493774.1	2.32	0.001	-11.17990909	4.48E-12	5.46E-11	LOC113841130	uncharacterized LOC113841130
MSTRG.4979.5	0.18	0.001	-7.491853096	5.12E-12	6.21E-11	-	-
XR_001189604.3	0.083333333	0.49	2.555816155	5.15E-12	6.22E-11	LOC106016717	uncharacterized LOC106016717
XR_002399434.2	0.22	0.036666667	-2.584962501	6.23E-12	7.41E-11	LOC110351690	uncharacterized LOC110351690
XR_002398969.2	0.05	0.84	4.070389328	8.35E-12	9.75E-11	LOC106014835	uncharacterized LOC106014835
XR_003496279.1	0.06	0.3	2.321928095	8.91E-12	1.04E-10	LOC101795266	uncharacterized LOC101795266%2C transcript variant X3
XR_002402765.2	0.523333333	0.01	-5.709658248	1.08E-11	1.24E-10	LOC110352941	uncharacterized LOC110352941%2C transcript variant X1
XR_003493103.1	0.683333333	0.063333333	-3.431552586	1.08E-11	1.24E-10	LOC113840309	uncharacterized LOC113840309
XR_216744.4	1.716666667	0.123333333	-3.798975256	1.29E-11	1.48E-10	LOC101791391	uncharacterized LOC101791391%2C transcript variant X2
XR_002402696.2	0.056666667	0.296666667	2.38827059	1.60E-11	1.81E-10	LOC106017443	uncharacterized LOC106017443
MSTRG.962.1	0.623333333	0.001	-9.283860054	1.86E-11	2.10E-10	-	-
MSTRG.3052.11	0.643333333	0.001	-9.329422631	1.94E-11	2.20E-10	-	-
MSTRG.12949.1	2.29	0.446666667	-2.358077098	2.09E-11	2.35E-10	-	-
XR_003500722.1	0.476666667	0.033333333	-3.837943242	2.20E-11	2.47E-10	LOC113845266	uncharacterized LOC113845266
XR_003495918.1	0.88	0.001	-9.781359714	2.30E-11	2.58E-10	LOC113843035	uncharacterized LOC113843035
XR_003500183.1	0.206666667	0.001	-7.691161905	2.78E-11	3.11E-10	LOC106020052	uncharacterized LOC106020052%2C transcript variant X1
XR_003494138.1	0.353333333	0.001	-8.464886049	2.80E-11	3.11E-10	LOC113841694	uncharacterized LOC113841694
XR_003493496.1	0.63	0.001	-9.299208018	2.84E-11	3.16E-10	LOC113840871	uncharacterized LOC113840871%2C transcript variant X2
XR_003497886.1	0.001	0.356666667	8.478432581	3.47E-11	3.84E-10	LOC106019172	uncharacterized LOC106019172%2C transcript variant X3

XR_002406644.2	0.263333333	0.006666667	-5.303780748	3.84E-11	4.24E-10	LOC110354539	uncharacterized LOC110354539%2C transcript variant X2
MSTRG.13937.5	5.426666667	1.04	-2.383482765	3.87E-11	4.27E-10	-	-
XR_002402359.2	0.403333333	0.001	-8.655828831	4.58E-11	5.03E-10	LOC106017212	uncharacterized LOC106017212%2C transcript variant X4
XR_003497567.1	0.08	0.001	-6.321928095	4.66E-11	5.10E-10	LOC101802970	uncharacterized LOC101802970%2C transcript variant X5
XR_003494959.1	0.803333333	0.001	-9.64985493	5.62E-11	6.09E-10	LOC101799909	uncharacterized LOC101799909%2C transcript variant X3
XR_002404947.2	0.34	0.043333333	-2.971985624	6.25E-11	6.75E-10	LOC110353846	uncharacterized LOC110353846
XR_002399172.2	0.343333333	0.001	-8.423466121	6.30E-11	6.78E-10	LOC110351568	uncharacterized LOC110351568%2C transcript variant X1
XR_001190983.3	0.673333333	0.001	-9.395177077	6.72E-11	7.21E-10	LOC106017488	uncharacterized LOC106017488
MSTRG.16489.2	0.666666667	3.24	2.280956314	6.86E-11	7.33E-10	-	-
XR_003498470.1	0.433333333	0.001	-8.759333407	7.17E-11	7.65E-10	LOC113844163	uncharacterized LOC113844163%2C transcript variant X1
XR_003501052.1	2.223333333	0.001	-11.11850855	8.24E-11	8.70E-10	LOC113845463	uncharacterized LOC113845463%2C transcript variant X3
XR_003492632.1	0.586666667	0.076666667	-2.935869663	8.71E-11	9.18E-10	LOC106018295	uncharacterized LOC106018295%2C transcript variant X1
XR_003498141.1	0.64	0.033333333	-4.263034406	1.14E-10	1.19E-09	LOC113844039	uncharacterized LOC113844039
XR_003500864.1	0.156666667	0.016666667	-3.232660757	1.80E-10	1.86E-09	LOC113845332	uncharacterized LOC113845332
XR_003500052.1	0.783333333	0.12	-2.706591945	1.95E-10	2.01E-09	LOC113844972	uncharacterized LOC113844972%2C transcript variant X2
XR_003493610.1	0.573333333	0.001	-9.163230349	1.96E-10	2.01E-09	LOC113840994	uncharacterized LOC113840994
XR_002400249.2	0.42	0.043333333	-3.276840205	2.22E-10	2.27E-09	LOC110351990	uncharacterized LOC110351990

XR_003499827.1	0.126666667	0.01	-3.662965013	2.24E-10	2.29E-09	LOC106019660	uncharacterized LOC106019660
XR_002401779.2	0.45	0.001	-8.813781191	2.44E-10	2.48E-09	LOC110352593	uncharacterized LOC110352593%2C transcript variant X1
XR_003501326.1	0.18	0.736666667	2.033015057	2.63E-10	2.65E-09	LOC113845603	uncharacterized LOC113845603
XR_003494318.1	0.673333333	0.001	-9.395177077	2.68E-10	2.70E-09	LOC113841955	uncharacterized LOC113841955
XR_003499753.1	0.33	0.01	-5.044394119	3.65E-10	3.64E-09	LOC113844812	uncharacterized LOC113844812
XR_003497529.1	3.12	0.013333333	-7.87036472	3.78E-10	3.76E-09	LOC113843776	uncharacterized LOC113843776
MSTRG.12747.4	2.356666667	0.096666667	-4.60758541	3.88E-10	3.85E-09	-	-
XR_003496222.1	0.566666667	0.001	-9.14635653	4.62E-10	4.53E-09	LOC101792916	uncharacterized LOC101792916
XR_003496598.1	0.5	0.056666667	-3.141355849	5.09E-10	4.99E-09	LOC113843320	uncharacterized LOC113843320
XR_001193468.3	0.683333333	0.016666667	-5.357552005	6.05E-10	5.90E-09	LOC106018998	uncharacterized LOC106018998
MSTRG.16636.1	0.95	0.001	-9.891783703	7.57E-10	7.33E-09	-	-
XR_003493941.1	0.516666667	0.001	-9.013089999	8.63E-10	8.32E-09	LOC113841385	uncharacterized LOC113841385
MSTRG.16632.2	0.486666667	0.001	-8.926790153	8.87E-10	8.53E-09	-	-
XR_003497951.1	1.136666667	0.001	-10.15059352	8.92E-10	8.56E-09	LOC113843960	uncharacterized LOC113843960%2C transcript variant X1
XR_001187865.3	1.21	0.001	-10.24079133	9.03E-10	8.65E-09	LOC106015672	uncharacterized LOC106015672%2C transcript variant X2
XR_003501403.1	1.3	0.001	-10.34429591	1.07E-09	1.02E-08	LOC113845679	uncharacterized LOC113845679
MSTRG.17689.1	4.723333333	0.001	-12.20558964	1.26E-09	1.19E-08	-	-
XR_003498021.1	0.123333333	0.01	-3.624490865	1.53E-09	1.45E-08	LOC101803684	uncharacterized LOC101803684%2C transcript variant X14
XR_003498718.1	0.17	0.03	-2.502500341	1.96E-09	1.84E-08	LOC113844276	uncharacterized LOC113844276%2C transcript variant X1
MSTRG.963.2	0.313333333	0.006666667	-5.554588852	2.25E-09	2.10E-08	-	-

XR_001194441.2	1.183333333	0.001	-10.20864081	3.24E-09	3.00E-08	LOC106019549	uncharacterized LOC106019549%2C transcript variant X3
XR_002401026.2	0.306666667	0.01	-4.938599455	3.25E-09	3.00E-08	LOC110352298	uncharacterized LOC110352298
XR_001189546.3	0.863333333	0.001	-9.753773882	3.52E-09	3.24E-08	LOC106016672	uncharacterized LOC106016672%2C transcript variant X1
XR_002406729.2	0.833333333	0.006666667	-6.965784285	3.72E-09	3.41E-08	LOC106020349	uncharacterized LOC106020349%2C transcript variant X2
MSTRG.961.1	0.276666667	0.006666667	-5.375039431	3.85E-09	3.52E-08	-	-
XR_002398893.2	1.396666667	0.073333333	-4.251374815	4.22E-09	3.85E-08	LOC110351436	uncharacterized LOC110351436
MSTRG.17799.1	0.426666667	0.006666667	-6	4.50E-09	4.10E-08	-	-
XR_003495767.1	2.11	0.05	-5.399171094	4.76E-09	4.32E-08	LOC110352593	uncharacterized LOC110352593%2C transcript variant X3
XR_002405956.2	0.15	0.001	-7.22881869	4.87E-09	4.41E-08	LOC106019750	uncharacterized LOC106019750%2C transcript variant X1
XR_003496294.1	0.043333333	0.276666667	2.674599713	5.61E-09	5.04E-08	LOC106015647	uncharacterized LOC106015647%2C transcript variant X14
XR_003493806.1	2.056666667	0.006666667	-8.269126679	5.70E-09	5.11E-08	LOC113841174	uncharacterized LOC113841174
MSTRG.16632.1	0.776666667	0.001	-9.601151739	5.72E-09	5.12E-08	-	-
XR_001188481.3	0.596666667	0.001	-9.220781371	6.55E-09	5.85E-08	LOC106016032	uncharacterized LOC106016032%2C transcript variant X4
XR_003494883.1	0.84	0.03	-4.807354922	6.65E-09	5.92E-08	LOC106020483	uncharacterized LOC106020483%2C transcript variant X4
XR_002398636.2	0.166666667	0.773333333	2.214124805	6.72E-09	5.97E-08	LOC110351346	uncharacterized LOC110351346%2C transcript variant X2
XR_003495124.1	0.293333333	0.023333333	-3.652076697	7.18E-09	6.37E-08	LOC113842605	uncharacterized LOC113842605
XR_001186606.3	0.263333333	0.001	-8.040746342	7.66E-09	6.77E-08	LOC106014935	uncharacterized LOC106014935

XR_003495483.1	0.323333333	0.003333333	-6.599912842	7.78E-09	6.86E-08	LOC113842770	uncharacterized LOC113842770
XR_003501475.1	0.67	0.001	-9.388017285	7.81E-09	6.87E-08	LOC106019132	uncharacterized LOC106019132%2C transcript variant X2
XR_003497568.1	0.046666667	0.001	-5.544320516	9.18E-09	8.04E-08	LOC101802970	uncharacterized LOC101802970%2C transcript variant X6
XR_003494887.1	0.756666667	0.01	-6.241585987	9.33E-09	8.16E-08	LOC113842395	uncharacterized LOC113842395
MSTRG.16558.4	1.42	0.11	-3.690315501	9.38E-09	8.18E-08	-	-
XR_217495.4	0.313333333	0.046666667	-2.74723393	1.10E-08	9.55E-08	LOC101790744	uncharacterized LOC101790744
XR_002399008.2	0.38	0.013333333	-4.832890014	1.36E-08	1.16E-07	LOC106014868	uncharacterized LOC106014868%2C transcript variant X3
XR_003494714.1	0.35	0.01	-5.129283017	1.44E-08	1.24E-07	LOC113842344	uncharacterized LOC113842344
XR_003494409.1	1.256666667	0.001	-10.29538631	1.46E-08	1.25E-07	LOC113842119	uncharacterized LOC113842119
XR_003497294.1	0.18	0.04	-2.169925001	1.49E-08	1.27E-07	LOC106019423	uncharacterized LOC106019423
MSTRG.10341.22	15.8	0.106666667	-7.210671344	1.59E-08	1.35E-07	-	-
MSTRG.887.1	0.79	0.08	-3.303780748	1.67E-08	1.42E-07	-	-
XR_002405954.2	0.533333333	0.001	-9.058893689	1.94E-08	1.64E-07	LOC110354267	uncharacterized LOC110354267%2C transcript variant X6
XR_001190624.3	0.293333333	0.02	-3.874469118	2.01E-08	1.69E-07	LOC106017263	uncharacterized LOC106017263
XR_003497400.1	0.103333333	0.01	-3.36923381	2.03E-08	1.71E-07	LOC113843702	uncharacterized LOC113843702
XR_002405511.2	2.696666667	0.616666667	-2.128614432	2.24E-08	1.88E-07	LOC101799390	uncharacterized LOC101799390%2C transcript variant X2
XR_003496360.1	0.553333333	0.033333333	-4.053111336	2.26E-08	1.89E-07	LOC106020128	uncharacterized LOC106020128%2C transcript variant X7
XR_003494837.1	1.02	0.216666667	-2.23502003	2.35E-08	1.96E-07	LOC106016770	uncharacterized LOC106016770%2C transcript variant X4

MSTRG.10341.21	4.806666667	0.086666667	-5.793415731	2.46E-08	2.04E-07	-	-
MSTRG.17824.1	0.713333333	0.001	-9.478432581	2.70E-08	2.24E-07	-	-
XR_002402026.2	0.423333333	0.05	-3.081794091	2.74E-08	2.26E-07	LOC110352675	uncharacterized LOC110352675
XR_002398923.2	0.62	0.043333333	-3.838719093	3.00E-08	2.46E-07	LOC101798301	uncharacterized LOC101798301%2C transcript variant X9
XR_001191530.3	0.1	0.001	-6.64385619	3.38E-08	2.75E-07	LOC106017832	uncharacterized LOC106017832
XR_003493495.1	0.526666667	0.001	-9.040746342	3.52E-08	2.86E-07	LOC113840871	uncharacterized LOC113840871%2C transcript variant X1
MSTRG.16156.2	0.001	0.83	9.696967526	3.88E-08	3.15E-07	-	-
XR_003494810.1	0.196666667	0.013333333	-3.882643049	4.15E-08	3.35E-07	LOC106018470	uncharacterized LOC106018470
MSTRG.14530.3	0.68	3.23	2.247927513	4.22E-08	3.40E-07	-	-
XR_003496286.1	0.001	0.133333333	7.058893689	4.91E-08	3.92E-07	LOC106015647	uncharacterized LOC106015647%2C transcript variant X6
XR_003496447.1	0.96	0.033333333	-4.847996907	6.31E-08	5.00E-07	LOC113843228	uncharacterized LOC113843228
XR_003497909.1	0.56	0.126666667	-2.144389909	7.32E-08	5.80E-07	LOC113843945	uncharacterized LOC113843945
XR_003494770.1	0.363333333	0.01	-5.183221824	7.45E-08	5.89E-07	LOC106018037	uncharacterized LOC106018037%2C transcript variant X2
XR_002405606.2	1.113333333	0.006666667	-7.383704292	7.80E-08	6.15E-07	LOC106019471	uncharacterized LOC106019471%2C transcript variant X2
MSTRG.13678.4	0.516666667	6.32	3.612618844	7.89E-08	6.21E-07	-	-
XR_002399884.2	0.323333333	0.026666667	-3.599912842	8.17E-08	6.41E-07	LOC110351873	uncharacterized LOC110351873
XR_003497665.1	0.13	0.001	-7.022367813	8.84E-08	6.91E-07	LOC110351913	uncharacterized LOC110351913%2C transcript variant X2
XR_003496331.1	0.006666667	0.4	5.906890596	8.87E-08	6.92E-07	LOC113843175	uncharacterized LOC113843175%2C transcript variant X1

XR_003499996.1	0.15	0.006666667	-4.491853096	8.98E-08	6.99E-07	LOC106014680	uncharacterized LOC106014680%2C transcript variant X1
MSTRG.963.1	0.346666667	0.003333333	-6.700439718	9.19E-08	7.14E-07	-	-
XR_003497331.1	0.1	0.696666667	2.800468536	9.96E-08	7.73E-07	LOC113843661	uncharacterized LOC113843661
XR_002401448.2	0.001	0.42	8.714245518	1.00E-07	7.75E-07	LOC101796749	uncharacterized LOC101796749%2C transcript variant X19
XR_003498910.1	0.74	0.073333333	-3.334984248	1.02E-07	7.89E-07	LOC106017955	uncharacterized LOC106017955
XR_003499078.1	0.76	0.1	-2.925999419	1.05E-07	8.09E-07	LOC113844446	uncharacterized LOC113844446
XR_003493328.1	0.84	0.103333333	-3.023083613	1.07E-07	8.28E-07	LOC106019302	uncharacterized LOC106019302%2C transcript variant X2
XR_003500051.1	0.7	0.056666667	-3.626782676	1.11E-07	8.52E-07	LOC113844972	uncharacterized LOC113844972%2C transcript variant X1
XR_003496139.1	0.21	0.001	-7.714245518	1.20E-07	9.21E-07	LOC113843116	uncharacterized LOC113843116
XR_002405612.2	0.99	0.013333333	-6.214319121	1.24E-07	9.49E-07	LOC106019471	uncharacterized LOC106019471%2C transcript variant X1
XR_002399273.2	0.04	0.27	2.754887502	1.45E-07	1.10E-06	LOC110351626	uncharacterized LOC110351626%2C transcript variant X2
XR_003498397.1	0.226666667	0.016666667	-3.765534746	1.47E-07	1.11E-06	LOC101797653	uncharacterized LOC101797653%2C transcript variant X3
XR_002401371.2	0.03	0.19	2.662965013	1.58E-07	1.19E-06	LOC110352432	uncharacterized LOC110352432
MSTRG.7242.1	0.19	0.001	-7.569855608	1.63E-07	1.22E-06	-	-
MSTRG.16693.1	2.103333333	0.096666667	-4.4435152	1.64E-07	1.23E-06	-	-
XR_003493635.1	0.001	0.14	7.129283017	2.05E-07	1.53E-06	LOC113841023	uncharacterized LOC113841023%2C transcript variant X4
XR_002406139.2	0.58	0.063333333	-3.195015982	2.09E-07	1.56E-06	LOC110354333	uncharacterized LOC110354333
XR_003499843.1	0.006666667	0.093333333	3.807354922	2.21E-07	1.65E-06	LOC113844885	uncharacterized LOC113844885

MSTRG.7331.1	0.866666667	0.001	-9.759333407	2.34E-07	1.74E-06	-	-
XR_003496332.1	0.033333333	0.393333333	3.560714954	2.61E-07	1.93E-06	LOC113843175	uncharacterized LOC113843175%2C transcript variant X2
XR_003500319.1	0.12	0.01	-3.584962501	2.85E-07	2.10E-06	LOC113845078	uncharacterized LOC113845078%2C transcript variant X3
XR_001186962.3	0.116666667	0.001	-6.866248611	2.89E-07	2.12E-06	LOC106015138	uncharacterized LOC106015138
XR_003498864.1	0.173333333	0.006666667	-4.700439718	2.89E-07	2.12E-06	LOC113844340	uncharacterized LOC113844340%2C transcript variant X2
XR_002399432.2	0.1	0.001	-6.64385619	3.03E-07	2.22E-06	LOC110351688	uncharacterized LOC110351688
XR_002399433.2	0.34	0.043333333	-2.971985624	3.14E-07	2.30E-06	LOC110351689	uncharacterized LOC110351689
MSTRG.16618.1	0.323333333	0.003333333	-6.599912842	3.20E-07	2.34E-06	-	-
XR_002402518.2	0.126666667	0.003333333	-5.247927513	3.56E-07	2.60E-06	LOC110352849	uncharacterized LOC110352849
XR_003496264.1	0.06	0.466666667	2.959358016	3.60E-07	2.62E-06	LOC113843168	uncharacterized LOC113843168%2C transcript variant X1
XR_002401835.2	0.333333333	0.003333333	-6.64385619	3.61E-07	2.62E-06	LOC110352617	uncharacterized LOC110352617
MSTRG.16889.1	4.083333333	0.043333333	-6.558126316	3.71E-07	2.70E-06	-	-
MSTRG.17825.1	0.456666667	0.003333333	-7.098032083	3.73E-07	2.70E-06	-	-
XR_003496363.1	0.253333333	0.001	-7.984893108	4.14E-07	2.99E-06	LOC106020128	uncharacterized LOC106020128%2C transcript variant X8
XR_003495345.1	0.69	0.013333333	-5.693486957	4.89E-07	3.49E-06	LOC101793386	uncharacterized LOC101793386
XR_001190631.3	0.09	0.006666667	-3.754887502	4.91E-07	3.50E-06	LOC106017268	uncharacterized LOC106017268
XR_002400653.2	0.243333333	0.01	-4.604862058	4.97E-07	3.53E-06	LOC110352151	uncharacterized LOC110352151%2C transcript variant X2
XR_001191855.3	0.593333333	0.01	-5.89077093	5.51E-07	3.89E-06	LOC106018027	uncharacterized LOC106018027
XR_003500294.1	0.036666667	0.293333333	3	5.66E-07	3.99E-06	LOC106018900	uncharacterized LOC106018900%2C

							transcript variant X4
XR_003492941.1	0.013333333	0.663333333	5.636624621	6.96E-07	4.87E-06	LOC106017294	uncharacterized LOC106017294
XR_001193442.3	0.183333333	0.003333333	-5.781359714	6.98E-07	4.88E-06	LOC106018981	uncharacterized LOC106018981
XR_003495202.1	0.31	0.03	-3.36923381	7.48E-07	5.21E-06	LOC113842645	uncharacterized LOC113842645
XR_003492820.1	0.156666667	1.053333333	2.749191896	7.73E-07	5.36E-06	LOC101796365	uncharacterized LOC101796365
MSTRG.16560.1	0.546666667	0.001	-9.094517599	8.43E-07	5.83E-06	-	-
XR_002399447.2	0.316666667	0.026666667	-3.569855608	8.45E-07	5.83E-06	LOC110351699	uncharacterized LOC110351699
XR_003495513.1	0.326666667	0.01	-5.029747343	9.45E-07	6.51E-06	LOC106018810	uncharacterized LOC106018810%2C transcript variant X1
XR_003493239.1	1.12	0.153333333	-2.868755467	9.62E-07	6.60E-06	LOC106014574	uncharacterized LOC106014574%2C transcript variant X1
XR_003497560.1	0.083333333	0.001	-6.380821784	1.02E-06	6.97E-06	LOC110354397	uncharacterized LOC110354397%2C transcript variant X12
MSTRG.3832.2	2.38	0.11	-4.435386145	1.09E-06	7.46E-06	-	-
XR_003493331.1	0.26	0.026666667	-3.285402219	1.12E-06	7.61E-06	LOC106019302	uncharacterized LOC106019302%2C transcript variant X3
XR_001195514.2	2.11	0.443333333	-2.250779254	1.22E-06	8.24E-06	LOC106020129	uncharacterized LOC106020129%2C transcript variant X1
XR_003497483.1	0.001	0.33	8.366322214	1.22E-06	8.27E-06	LOC110354152	uncharacterized LOC110354152%2C transcript variant X1
XR_003499550.1	0.14	0.001	-7.129283017	1.35E-06	9.09E-06	LOC106016478	uncharacterized LOC106016478%2C transcript variant X2
MSTRG.14400.1	0.216666667	2.086666667	3.267651034	1.51E-06	1.02E-05	-	-
XR_003499478.1	0.036666667	0.001	-5.196397213	1.61E-06	1.08E-05	LOC106016319	uncharacterized LOC106016319%2C transcript variant X3
MSTRG.4652.3	2.463333333	0.443333333	-2.474148119	1.68E-06	1.13E-05	-	-

MSTRG.9062.4	1.686666667	0.02	-6.398031074	1.73E-06	1.16E-05	-	-
XR_003496383.1	0.733333333	0.18	-2.026472211	1.81E-06	1.20E-05	LOC113839623	uncharacterized LOC113839623
XR_002404500.2	0.08	0.001	-6.321928095	1.85E-06	1.23E-05	LOC106018748	uncharacterized LOC106018748
XR_003500730.1	0.213333333	0.036666667	-2.540568381	1.86E-06	1.24E-05	LOC113845270	uncharacterized LOC113845270
MSTRG.2763.3	0.001	0.103333333	6.691161905	1.93E-06	1.28E-05	-	-
XR_002405230.2	0.06	0.363333333	2.598259323	1.95E-06	1.29E-05	LOC106019237	uncharacterized LOC106019237
MSTRG.7621.5	1.306666667	0.001	-10.35167544	2.10E-06	1.39E-05	-	-
XR_002406465.2	1.073333333	0.14	-2.938599455	2.36E-06	1.55E-05	LOC106020114	uncharacterized LOC106020114
XR_002399439.2	0.056666667	0.01	-2.502500341	2.39E-06	1.57E-05	LOC110351692	uncharacterized LOC110351692%2C transcript variant X2
XR_002400673.2	0.16	0.016666667	-3.263034406	2.47E-06	1.62E-05	LOC106016078	uncharacterized LOC106016078
MSTRG.13035.15	2.013333333	0.23	-3.129880283	2.53E-06	1.65E-05	-	-
XR_002406639.2	0.133333333	0.001	-7.058893689	2.62E-06	1.71E-05	LOC106020257	uncharacterized LOC106020257%2C transcript variant X3
XR_003494228.1	0.02	0.44	4.459431619	2.94E-06	1.90E-05	LOC101794649	uncharacterized LOC101794649%2C transcript variant X7
XR_001189916.3	3.516666667	0.16	-4.458064783	3.45E-06	2.21E-05	LOC106016889	uncharacterized LOC106016889%2C transcript variant X1
XR_003493231.1	0.16	0.001	-7.321928095	3.53E-06	2.26E-05	LOC113840405	uncharacterized LOC113840405
MSTRG.2264.2	1.613333333	0.376666667	-2.098684275	3.65E-06	2.32E-05	-	-
XR_003499027.1	0.123333333	0.006666667	-4.209453366	3.66E-06	2.32E-05	LOC113844432	uncharacterized LOC113844432
XR_002402176.2	0.4	0.006666667	-5.906890596	3.74E-06	2.37E-05	LOC110352712	uncharacterized LOC110352712
XR_003492875.1	0.263333333	0.06	-2.133855747	4.01E-06	2.54E-05	LOC101791467	uncharacterized LOC101791467%2C transcript variant X1
XR_002401580.2	0.206666667	0.001	-7.691161905	4.15E-06	2.62E-05	LOC110352526	uncharacterized LOC110352526%2C

							transcript variant X2
XR_003498560.1	1.68	0.353333333	-2.249359469	4.31E-06	2.72E-05	LOC113844194	uncharacterized LOC113844194
XR_002402383.2	0.276666667	0.001	-8.112005026	4.77E-06	2.99E-05	LOC110352801	uncharacterized LOC110352801
XR_003497816.1	0.166666667	0.001	-7.380821784	5.04E-06	3.14E-05	LOC113843897	uncharacterized LOC113843897
XR_216772.4	0.346666667	0.043333333	-3	5.30E-06	3.30E-05	LOC101802284	uncharacterized LOC101802284
MSTRG.5643.4	0.11	1.486666667	3.756505781	5.44E-06	3.38E-05	-	-
XR_003499481.1	0.176666667	0.023333333	-2.920565533	5.44E-06	3.38E-05	LOC106016319	uncharacterized LOC106016319%2C transcript variant X6
XR_003496013.1	0.306666667	0.06	-2.353636955	5.94E-06	3.67E-05	LOC110352195	uncharacterized LOC110352195
XR_003496080.1	0.58	0.11	-2.398549376	6.48E-06	3.99E-05	LOC110352444	uncharacterized LOC110352444
XR_003499813.1	0.08	0.016666667	-2.263034406	6.57E-06	4.04E-05	LOC113844847	uncharacterized LOC113844847
XR_003495930.1	0.203333333	0.033333333	-2.608809243	6.81E-06	4.19E-05	LOC113839603	uncharacterized LOC113839603
XR_003496302.1	0.001	0.096666667	6.594946589	7.20E-06	4.41E-05	LOC106015647	uncharacterized LOC106015647%2C transcript variant X22
XR_003495411.1	0.326666667	0.001	-8.351675438	7.40E-06	4.53E-05	LOC113842740	uncharacterized LOC113842740
XR_003500903.1	0.536666667	0.086666667	-2.63047716	7.74E-06	4.72E-05	LOC113845349	uncharacterized LOC113845349
XR_003496907.1	0.183333333	0.006666667	-4.781359714	8.25E-06	5.01E-05	LOC113843533	uncharacterized LOC113843533
XR_001190951.3	0.246666667	0.001	-7.94641896	8.81E-06	5.33E-05	LOC106017466	uncharacterized LOC106017466
MSTRG.17818.1	0.616666667	0.093333333	-2.724026538	9.05E-06	5.47E-05	-	-
XR_003497968.1	0.103333333	0.001	-6.691161905	9.11E-06	5.49E-05	LOC110351792	uncharacterized LOC110351792
XR_003498597.1	0.01	0.15	3.906890596	9.57E-06	5.75E-05	LOC106017337	uncharacterized LOC106017337%2C transcript variant X1
XR_003492403.1	0.15	0.001	-7.22881869	1.02E-05	6.12E-05	LOC110352880	uncharacterized LOC110352880%2C transcript variant X1
XR_002402245.2	0.12	0.006666667	-4.169925001	1.04E-05	6.20E-05	LOC110352750	uncharacterized LOC110352750

XR_003498061.1	0.18	0.006666667	-4.754887502	1.23E-05	7.31E-05	LOC106015393	uncharacterized LOC106015393%2C transcript variant X2
XR_002405024.2	0.38	0.056666667	-2.745427173	1.23E-05	7.31E-05	LOC106019089	uncharacterized LOC106019089
XR_002405449.2	0.31	0.003333333	-6.539158811	1.24E-05	7.31E-05	LOC110354050	uncharacterized LOC110354050
XR_003498605.1	0.853333333	0.063333333	-3.752072487	1.24E-05	7.31E-05	LOC113844212	uncharacterized LOC113844212%2C transcript variant X2
XR_003499477.1	0.036666667	0.001	-5.196397213	1.24E-05	7.32E-05	LOC106016319	uncharacterized LOC106016319%2C transcript variant X2
MSTRG.11490.1	0.63	2.533333333	2.007613184	1.27E-05	7.52E-05	-	-
XR_002398783.2	0.676666667	0.13	-2.379933698	1.28E-05	7.53E-05	LOC106014662	uncharacterized LOC106014662
XR_001190278.3	0.226666667	0.003333333	-6.087462841	1.35E-05	7.91E-05	LOC106017074	uncharacterized LOC106017074%2C transcript variant X2
XR_001194424.3	0.001	0.546666667	9.094517599	1.40E-05	8.16E-05	LOC106019534	uncharacterized LOC106019534%2C transcript variant X1
XR_001188458.3	0.773333333	0.09	-3.103093493	1.42E-05	8.32E-05	LOC106016032	uncharacterized LOC106016032%2C transcript variant X2
XR_001187017.3	0.49	0.001	-8.936637939	1.45E-05	8.45E-05	LOC106015179	uncharacterized LOC106015179%2C transcript variant X1
XR_003496425.1	0.67	0.07	-3.258734268	1.54E-05	8.97E-05	LOC113843216	uncharacterized LOC113843216
XR_002400941.2	0.366666667	0.001	-8.518325308	1.57E-05	9.12E-05	LOC106016276	uncharacterized LOC106016276
XR_002400440.2	0.41	0.001	-8.6794801	1.61E-05	9.32E-05	LOC106016032	uncharacterized LOC106016032%2C transcript variant X1
XR_003492946.1	1.046666667	0.066666667	-3.972692654	1.75E-05	0.000100737	LOC113840172	uncharacterized LOC113840172
XR_003498151.1	0.513333333	0.036666667	-3.807354922	1.83E-05	0.000105422	LOC110353008	uncharacterized LOC110353008%2C transcript variant X2
XR_003496431.1	0.64	0.096666667	-2.726981506	2.03E-05	0.00011644	LOC113843220	uncharacterized LOC113843220

XR_003496519.1	0.55	0.093333333	-2.558967292	2.17E-05	0.00012402	LOC113843274	uncharacterized LOC113843274
XR_003499172.1	0.17	0.016666667	-3.350497247	2.35E-05	0.000133404	LOC106020111	uncharacterized LOC106020111%2C transcript variant X2
XR_003501504.1	0.893333333	0.196666667	-2.183446141	2.37E-05	0.000134399	LOC113845747	uncharacterized LOC113845747
MSTRG.11582.4	0.21	0.046666667	-2.169925001	2.37E-05	0.00013441	-	-
XR_003497511.1	0.173333333	0.001	-7.437405312	2.41E-05	0.00013683	LOC110353153	uncharacterized LOC110353153%2C transcript variant X2
XR_003498689.1	0.346666667	0.08	-2.115477217	2.46E-05	0.000139412	LOC113844262	uncharacterized LOC113844262
XR_001190389.3	0.713333333	0.02	-5.156504486	2.47E-05	0.000139599	LOC106017139	uncharacterized LOC106017139
XR_003499109.1	0.18	0.001	-7.491853096	2.58E-05	0.000145138	LOC113844462	uncharacterized LOC113844462%2C transcript variant X6
XR_003498265.1	0.053333333	0.853333333	4	2.68E-05	0.000150274	LOC106020436	uncharacterized LOC106020436
XR_003498139.1	0.103333333	0.001	-6.691161905	2.87E-05	0.000160064	LOC106016400	uncharacterized LOC106016400%2C transcript variant X4
MSTRG.5725.1	0.096666667	2.06	4.413482033	2.93E-05	0.000162891	-	-
XR_002402795.2	0.13	0.001	-7.022367813	3.00E-05	0.000166273	LOC106017517	uncharacterized LOC106017517
XR_003495398.1	0.323333333	0.053333333	-2.599912842	3.19E-05	0.00017657	LOC113842734	uncharacterized LOC113842734
XR_002401846.2	0.35	0.01	-5.129283017	3.31E-05	0.000182838	LOC106016921	uncharacterized LOC106016921
XR_001191753.3	0.426666667	0.026666667	-4	3.35E-05	0.00018505	LOC106017960	uncharacterized LOC106017960
MSTRG.6312.1	0.146666667	1.696666667	3.532090227	3.45E-05	0.000189871	-	-
XR_003494461.1	0.15	0.003333333	-5.491853096	3.77E-05	0.000207173	LOC113842123	uncharacterized LOC113842123%2C transcript variant X50
XR_002398926.2	2.226666667	0.533333333	-2.061776198	3.83E-05	0.000210454	LOC101798301	uncharacterized LOC101798301%2C transcript variant X11
XR_002400526.2	0.2	0.001	-7.64385619	3.96E-05	0.00021655	LOC110352104	uncharacterized LOC110352104

XR_001189809.3	0.123333333	0.001	-6.94641896	4.12E-05	0.000224301	LOC106016826	uncharacterized LOC106016826%2C transcript variant X4
MSTRG.11297.8	0.54	0.073333333	-2.880418384	4.40E-05	0.000238757	-	-
XR_002401364.2	0.446666667	0.001	-8.803054785	4.49E-05	0.000243509	LOC110352428	uncharacterized LOC110352428
XR_001195078.3	0.813333333	0.016666667	-5.608809243	4.66E-05	0.000252174	LOC106019887	uncharacterized LOC106019887
XR_003497562.1	0.04	0.001	-5.321928095	4.70E-05	0.000254251	LOC110354397	uncharacterized LOC110354397%2C transcript variant X14
MSTRG.16566.1	0.37	0.001	-8.531381461	4.72E-05	0.000254603	-	-
XR_002405521.2	0.626666667	0.07	-3.162271429	4.79E-05	0.000258475	LOC106019445	uncharacterized LOC106019445
XR_003499525.1	0.006666667	0.103333333	3.95419631	4.99E-05	0.000268438	LOC110351396	uncharacterized LOC110351396%2C transcript variant X9
XR_002402799.2	0.203333333	0.001	-7.667702932	5.01E-05	0.000269201	LOC110352959	uncharacterized LOC110352959%2C transcript variant X2
XR_001186313.3	0.37	0.001	-8.531381461	5.11E-05	0.000274432	LOC101791391	uncharacterized LOC101791391%2C transcript variant X1
XR_001189244.3	0.476666667	0.01	-5.574908836	5.22E-05	0.000279753	LOC106016485	uncharacterized LOC106016485
MSTRG.256.1	4.31	0.633333333	-2.766650951	5.23E-05	0.000279785	-	-
XR_001193031.3	0.066666667	0.001	-6.058893689	5.48E-05	0.000291784	LOC101801592	uncharacterized LOC101801592%2C transcript variant X2
XR_003499791.1	0.293333333	0.023333333	-3.652076697	5.61E-05	0.000298324	LOC113844835	uncharacterized LOC113844835
MSTRG.196.1	1.173333333	0.04	-4.874469118	6.10E-05	0.000322543	-	-
XR_003495192.1	0.073333333	0.001	-6.196397213	6.37E-05	0.000335781	LOC113842640	uncharacterized LOC113842640
XR_003492300.1	0.426666667	0.046666667	-3.192645078	6.46E-05	0.000340288	LOC110354130	uncharacterized LOC110354130
XR_003494652.1	0.143333333	0.02	-2.841302254	6.77E-05	0.000356232	LOC113842315	uncharacterized LOC113842315%2C transcript variant X2

XR_003498030.1	0.113333333	0.003333333	-5.087462841	7.20E-05	0.000378474	LOC101803684	uncharacterized LOC101803684%2C transcript variant X23
XR_003499254.1	3.06	0.726666667	-2.074166019	7.49E-05	0.00039325	LOC106020392	uncharacterized LOC106020392
XR_003498693.1	1.31	0.02	-6.033423002	7.58E-05	0.000397091	LOC110354267	uncharacterized LOC110354267%2C transcript variant X2
XR_002399017.2	0.463333333	0.001	-8.855906667	7.63E-05	0.000399421	LOC110351499	uncharacterized LOC110351499
XR_001187494.3	0.296666667	0.006666667	-5.475733431	8.22E-05	0.000427687	LOC106015449	uncharacterized LOC106015449
XR_001188728.3	0.23	0.056666667	-2.021061616	8.31E-05	0.000432055	LOC106016195	uncharacterized LOC106016195
XR_003495212.1	1.286666667	0.026666667	-5.592457037	8.47E-05	0.000439709	LOC113842655	uncharacterized LOC113842655
XR_003498620.1	0.806666667	0.14	-2.526545814	8.53E-05	0.000442142	LOC113844220	uncharacterized LOC113844220%2C transcript variant X3
XR_217118.4	0.093333333	0.001	-6.544320516	8.57E-05	0.000443528	LOC101796876	uncharacterized LOC101796876
MSTRG.16827.1	2.283333333	0.413333333	-2.465763867	8.61E-05	0.000445356	-	-
XR_001193935.3	0.413333333	0.01	-5.36923381	8.75E-05	0.000452179	LOC106019263	uncharacterized LOC106019263
XR_003497545.1	0.5	0.001	-8.965784285	9.25E-05	0.00047521	LOC110352959	uncharacterized LOC110352959%2C transcript variant X3
XR_003495917.1	0.273333333	0.06	-2.187627003	9.51E-05	0.000487005	LOC106015722	uncharacterized LOC106015722
XR_003499970.1	0.286666667	0.023333333	-3.618909833	9.69E-05	0.000495059	LOC110352806	uncharacterized LOC110352806%2C transcript variant X11
XR_002404244.2	0.213333333	0.006666667	-5	9.79E-05	0.000499829	LOC106018581	uncharacterized LOC106018581%2C transcript variant X1
XR_002399231.2	0.023333333	0.296666667	3.668378509	0.000100268	0.000510658	LOC113839622	uncharacterized LOC113839622
XR_003497899.1	0.066666667	0.001	-6.058893689	0.000101505	0.000513995	LOC110353379	uncharacterized LOC110353379
XR_003495978.1	0.001	0.216666667	7.759333407	0.000103117	0.000521489	LOC110352611	uncharacterized LOC110352611%2C transcript variant X1

XR_001194486.3	0.286666667	0.001	-8.163230349	0.000103991	0.000524648	LOC106019573	uncharacterized LOC106019573
XR_002407055.2	0.296666667	0.026666667	-3.475733431	0.000104105	0.000524648	LOC106014341	uncharacterized LOC106014341
XR_002398729.2	0.31	0.001	-8.276124405	0.000106372	0.000534827	LOC106014648	uncharacterized LOC106014648%2C transcript variant X2
XR_003494775.1	0.1	0.001	-6.64385619	0.000108408	0.000543168	LOC110352361	uncharacterized LOC110352361
XR_002406555.2	0.223333333	0.001	-7.803054785	0.000118412	0.000590402	LOC110354493	uncharacterized LOC110354493
XR_003499967.1	0.35	0.04	-3.129283017	0.000124867	0.000619898	LOC110352806	uncharacterized LOC110352806%2C transcript variant X8
XR_003497207.1	0.013333333	0.073333333	2.459431619	0.000125516	0.000621693	LOC113843624	uncharacterized LOC113843624
XR_003500751.1	0.103333333	0.003333333	-4.95419631	0.000130248	0.000642923	LOC113845280	uncharacterized LOC113845280
XR_003492230.1	0.146666667	0.003333333	-5.459431619	0.000131168	0.00064599	LOC113839672	uncharacterized LOC113839672
XR_002404473.2	0.436666667	0.083333333	-2.389566812	0.000132044	0.000649523	LOC101800890	uncharacterized LOC101800890%2C transcript variant X1
MSTRG.4525.1	1.82	0.313333333	-2.538168289	0.000138085	0.000677744	-	-
MSTRG.16370.1	0.073333333	1.973333333	4.750021747	0.000140162	0.000687158	-	-
XR_003493241.1	0.57	0.03	-4.247927513	0.000144218	0.000706245	LOC106014574	uncharacterized LOC106014574%2C transcript variant X3
XR_001190677.3	0.326666667	0.006666667	-5.614709844	0.00014756	0.000720915	LOC106017297	uncharacterized LOC106017297
XR_003500184.1	0.07	0.001	-6.129283017	0.000147713	0.000720915	LOC106020052	uncharacterized LOC106020052%2C transcript variant X2
XR_003493917.1	1.173333333	0.001	-10.19639721	0.000148852	0.000725655	LOC113841351	uncharacterized LOC113841351
XR_003500293.1	0.01	0.263333333	4.718818247	0.000149526	0.000728121	LOC106018900	uncharacterized LOC106018900%2C transcript variant X3
XR_002405115.2	0.001	0.3	8.22881869	0.000150839	0.000733685	LOC110353906	uncharacterized LOC110353906
XR_002399898.2	0.22	0.001	-7.781359714	0.000152077	0.000738877	LOC110351880	uncharacterized LOC110351880%2C

							transcript variant X1
XR_003494768.1	0.283333333	0.006666667	-5.409390936	0.000153221	0.000743605	LOC106018037	uncharacterized LOC106018037%2C transcript variant X1
XR_001189913.3	0.25	0.023333333	-3.421463768	0.000154278	0.000747895	LOC106016887	uncharacterized LOC106016887
XR_003495628.1	0.343333333	0.001	-8.423466121	0.000162666	0.000786794	LOC113842828	uncharacterized LOC113842828
XR_003499014.1	0.15	0.006666667	-4.491853096	0.000164523	0.000794886	LOC106018873	uncharacterized LOC106018873%2C transcript variant X3
XR_001189247.3	0.226666667	0.001	-7.824428435	0.000169247	0.000816803	LOC106016487	uncharacterized LOC106016487
MSTRG.9851.1	0.116666667	0.593333333	2.346450414	0.000178411	0.000858159	-	-
XR_002401535.2	2.806666667	0.503333333	-2.479271684	0.000179257	0.00086127	LOC106016738	uncharacterized LOC106016738
XR_003499096.1	0.16	0.006666667	-4.584962501	0.000205257	0.000978585	LOC106020070	uncharacterized LOC106020070%2C transcript variant X2
XR_002402960.2	0.09	0.001	-6.491853096	0.00020836	0.0009912	LOC110353034	uncharacterized LOC110353034
XR_003494811.1	0.196666667	0.001	-7.619608644	0.000227548	0.001081292	LOC113842381	uncharacterized LOC113842381
XR_001186512.3	0.153333333	0.006666667	-4.523561956	0.000234321	0.001109818	LOC106014871	uncharacterized LOC106014871
XR_001193619.3	0.103333333	0.006666667	-3.95419631	0.000237989	0.001124734	LOC106019066	uncharacterized LOC106019066
XR_003494940.1	0.42	0.066666667	-2.655351829	0.00024201	0.001141242	LOC113842441	uncharacterized LOC113842441
XR_002405043.2	0.083333333	0.001	-6.380821784	0.000243072	0.001145002	LOC106019105	uncharacterized LOC106019105%2C transcript variant X1
XR_003498086.1	1.183333333	0.22	-2.427281095	0.000244326	0.00114966	LOC113844021	uncharacterized LOC113844021
XR_003498911.1	0.246666667	0.003333333	-6.209453366	0.000251718	0.001180592	LOC110352516	uncharacterized LOC110352516%2C transcript variant X1
XR_003499422.1	0.23	0.043333333	-2.408084739	0.000255361	0.001193799	LOC110351897	uncharacterized LOC110351897%2C transcript variant X3
XR_002400766.2	0.043333333	6.563333333	7.242807678	0.000255644	0.001193834	LOC110352189	uncharacterized LOC110352189%2C

							transcript variant X1
XR_003498825.1	0.053333333	0.001	-5.736965594	0.000292843	0.001356565	LOC101799512	uncharacterized LOC101799512%2C transcript variant X4
XR_002400226.2	0.213333333	0.001	-7.736965594	0.000293816	0.001358899	LOC106015733	uncharacterized LOC106015733
XR_002400594.2	0.143333333	0.01	-3.841302254	0.000298129	0.001377374	LOC110352134	uncharacterized LOC110352134
XR_003494295.1	0.76	0.001	-9.569855608	0.000304567	0.001402628	LOC113841920	uncharacterized LOC113841920%2C transcript variant X1
XR_001188086.3	0.06	0.001	-5.906890596	0.000315561	0.001448634	LOC106015802	uncharacterized LOC106015802
XR_002399969.2	0.126666667	0.006666667	-4.247927513	0.000341596	0.001558238	LOC106015640	uncharacterized LOC106015640%2C transcript variant X2
XR_002404933.2	0.12	0.006666667	-4.169925001	0.000344285	0.001568854	LOC106019030	uncharacterized LOC106019030%2C transcript variant X1
MSTRG.582.4	1.366666667	0.153333333	-3.155918143	0.000348529	0.001583192	-	-
XR_003496220.1	0.053333333	0.43	3.011227255	0.000356833	0.001619213	LOC113843156	uncharacterized LOC113843156
MSTRG.8113.3	1.386666667	0.116666667	-3.571156701	0.000359857	0.001631226	-	-
XR_003501407.1	0.096666667	0.001	-6.594946589	0.000380513	0.001717664	LOC110353611	uncharacterized LOC110353611%2C transcript variant X1
XR_001190946.3	0.29	0.001	-8.17990909	0.000412783	0.001858352	LOC106017462	uncharacterized LOC106017462%2C transcript variant X2
XR_003493072.1	0.276666667	0.026666667	-3.375039431	0.000414375	0.00186275	LOC113840294	uncharacterized LOC113840294
XR_003499444.1	0.016666667	0.436666667	4.711494907	0.000416123	0.001868669	LOC113844708	uncharacterized LOC113844708%2C transcript variant X8
XR_003495625.1	0.09	0.003333333	-4.754887502	0.000440765	0.001969114	LOC101797754	uncharacterized LOC101797754%2C transcript variant X8
XR_003499171.1	0.556666667	0.086666667	-2.683264574	0.000450671	0.002011293	LOC106020111	uncharacterized LOC106020111%2C transcript variant X1

XR_002398455.2	0.06	0.001	-5.906890596	0.000473837	0.002105996	LOC106014454	uncharacterized LOC106014454
XR_001189132.3	0.216666667	0.001	-7.759333407	0.000484426	0.002150853	LOC106016415	uncharacterized LOC106016415%2C transcript variant X1
XR_003500127.1	0.146666667	0.006666667	-4.459431619	0.000488235	0.002165543	LOC101789609	uncharacterized LOC101789609
XR_003495603.1	0.006666667	0.146666667	4.459431619	0.000496567	0.002200245	LOC106018653	uncharacterized LOC106018653%2C transcript variant X1
XR_001186561.3	0.073333333	0.001	-6.196397213	0.000517096	0.002288862	LOC106014905	uncharacterized LOC106014905%2C transcript variant X1
XR_003496756.1	0.3	0.03	-3.321928095	0.000526074	0.002326225	LOC113843454	uncharacterized LOC113843454
XR_002398728.2	0.466666667	0.016666667	-4.807354922	0.000536505	0.002369928	LOC106014648	uncharacterized LOC106014648%2C transcript variant X1
XR_003501399.1	0.56	0.013333333	-5.392317423	0.000549923	0.002426726	LOC113845676	uncharacterized LOC113845676
MSTRG.7980.2	0.206666667	0.043333333	-2.253756592	0.000555698	0.002449713	-	-
XR_001187202.3	0.18	0.001	-7.491853096	0.00058448	0.002568747	LOC106015285	uncharacterized LOC106015285
XR_001192197.3	0.203333333	0.001	-7.667702932	0.00060726	0.002663454	LOC106018218	uncharacterized LOC106018218
XR_002403638.2	0.103333333	0.001	-6.691161905	0.00060888	0.002667856	LOC110353307	uncharacterized LOC110353307%2C transcript variant X2
XR_003497509.1	0.056666667	0.003333333	-4.087462841	0.000612534	0.002681151	LOC113843772	uncharacterized LOC113843772%2C transcript variant X2
XR_002405644.2	0.353333333	0.076666667	-2.204358499	0.000621821	0.002710839	LOC110354114	uncharacterized LOC110354114
XR_003496800.1	0.106666667	0.023333333	-2.192645078	0.000626172	0.00272706	LOC110352340	uncharacterized LOC110352340%2C transcript variant X6
XR_002405450.2	0.29	0.001	-8.17990909	0.000644706	0.002799331	LOC110354051	uncharacterized LOC110354051%2C transcript variant X1
XR_003495108.1	0.033333333	0.46	3.786596362	0.000666085	0.002883483	LOC113842597	uncharacterized LOC113842597
XR_002400577.2	0.506666667	0.063333333	-3	0.000673522	0.002912762	LOC110352126	uncharacterized LOC110352126

XR_001189388.3	0.12	0.006666667	-4.169925001	0.000721535	0.003114183	LOC106016573	uncharacterized LOC106016573
XR_001193335.3	0.156666667	0.001	-7.291554446	0.000737418	0.003179564	LOC106018911	uncharacterized LOC106018911
XR_003499624.1	0.343333333	0.06	-2.516575526	0.000776313	0.003324094	LOC113844766	uncharacterized LOC113844766
XR_003494553.1	0.001	0.113333333	6.824428435	0.000780078	0.003336915	LOC101797243	uncharacterized LOC101797243%2C transcript variant X3
XR_002406597.2	0.13	0.01	-3.700439718	0.000802529	0.003419438	LOC110354516	uncharacterized LOC110354516
XR_002401018.2	0.236666667	0.001	-7.886712714	0.000810942	0.003451887	LOC110352292	uncharacterized LOC110352292
XR_003499428.1	0.013333333	0.26	4.285402219	0.000833703	0.003545286	LOC113844703	uncharacterized LOC113844703%2C transcript variant X2
XR_001189695.3	0.54	0.11	-2.295455884	0.000840345	0.003563028	LOC106016768	uncharacterized LOC106016768%2C transcript variant X2
MSTRG.16697.3	0.996666667	0.001	-9.960967268	0.000846875	0.003587202	-	-
XR_001194787.3	0.093333333	0.01	-3.222392421	0.000858371	0.003632343	LOC106019736	uncharacterized LOC106019736
XR_001188498.3	0.093333333	0.001	-6.544320516	0.000862097	0.003640991	LOC106016067	uncharacterized LOC106016067%2C transcript variant X1
XR_003498834.1	0.093333333	0.656666667	2.814696897	0.000863234	0.00364224	LOC113844330	uncharacterized LOC113844330
XR_003496232.1	0.09	0.006666667	-3.754887502	0.000866092	0.003650743	LOC110352727	uncharacterized LOC110352727%2C transcript variant X2
XR_001193695.2	0.053333333	0.003333333	-4	0.000889222	0.003737321	LOC106019125	uncharacterized LOC106019125
XR_003494588.1	0.083333333	0.001	-6.380821784	0.000902832	0.003790843	LOC113842251	uncharacterized LOC113842251
XR_001195751.3	0.156666667	0.001	-7.291554446	0.00091966	0.003854023	LOC106020304	uncharacterized LOC106020304%2C transcript variant X2
XR_003495697.1	0.4	0.03	-3.736965594	0.000936611	0.003921268	LOC113842864	uncharacterized LOC113842864%2C transcript variant X2
XR_003496459.1	0.153333333	0.01	-3.938599455	0.000945021	0.003948837	LOC106016136	uncharacterized LOC106016136%2C transcript variant X6

XR_003495354.1	0.083333333	0.403333333	2.275007047	0.000973065	0.004050383	LOC101803317	uncharacterized LOC101803317%2C transcript variant X2
XR_003496690.1	0.103333333	0.001	-6.691161905	0.000996853	0.004134063	LOC113843418	uncharacterized LOC113843418%2C transcript variant X1
XR_001187948.3	0.736666667	0.133333333	-2.465974465	0.001008757	0.00417886	LOC101789951	uncharacterized LOC101789951
XR_003495703.1	0.076666667	0.001	-6.26052755	0.001022081	0.004225968	LOC106014913	uncharacterized LOC106014913%2C transcript variant X1
XR_003497157.1	0.253333333	0.013333333	-4.247927513	0.001023323	0.004227064	LOC113843597	uncharacterized LOC113843597
XR_003501353.1	0.23	1.29	2.487665299	0.001032096	0.004257806	LOC113845622	uncharacterized LOC113845622
XR_001194939.3	0.04	0.003333333	-3.584962501	0.001032732	0.004257806	LOC106019800	uncharacterized LOC106019800
XR_002401263.2	0.14	0.001	-7.129283017	0.001040314	0.004284986	LOC106016476	uncharacterized LOC106016476%2C transcript variant X4
XR_002399473.2	0.023333333	0.001	-4.544320516	0.001044383	0.004295772	LOC110351709	uncharacterized LOC110351709%2C transcript variant X1
XR_003495352.1	0.06	0.001	-5.906890596	0.001044918	0.004295772	LOC113842713	uncharacterized LOC113842713
XR_003496243.1	0.323333333	0.05	-2.693022247	0.001117244	0.00457584	LOC110352708	uncharacterized LOC110352708%2C transcript variant X7
XR_002400870.1	0.1	0.003333333	-4.906890596	0.00111727	0.00457584	LOC110352222	uncharacterized LOC110352222
XR_002399346.2	0.096666667	0.001	-6.594946589	0.001120207	0.004583533	LOC106015170	uncharacterized LOC106015170%2C transcript variant X1
XR_003500375.1	0.096666667	0.001	-6.594946589	0.001124881	0.004593973	LOC106018420	uncharacterized LOC106018420
XR_003497023.1	0.02	0.001	-4.321928095	0.001129949	0.004605978	LOC113843564	uncharacterized LOC113843564
XR_002406820.2	0.006666667	0.28	5.392317423	0.001134482	0.004620107	LOC110354633	uncharacterized LOC110354633
XR_003501077.1	0.001	0.076666667	6.26052755	0.001141279	0.004643416	LOC113845483	uncharacterized LOC113845483%2C transcript variant X3
XR_003496550.1	0.426666667	0.046666667	-3.192645078	0.001145257	0.004655229	LOC106018878	uncharacterized LOC106018878%2C

							transcript variant X1
XR_001186984.3	0.283333333	0.001	-8.14635653	0.0011498	0.004664932	LOC106015161	uncharacterized LOC106015161%2C transcript variant X2
XR_002406957.2	0.116666667	0.001	-6.866248611	0.001150934	0.004665162	LOC110354729	uncharacterized LOC110354729%2C transcript variant X1
XR_001187991.3	0.173333333	0.013333333	-3.700439718	0.001211049	0.00490424	LOC106015755	uncharacterized LOC106015755
XR_003495853.1	0.03	0.323333333	3.429987841	0.0012124	0.00490436	LOC113842928	uncharacterized LOC113842928
XR_003495943.1	0.206666667	0.006666667	-4.95419631	0.001213345	0.00490436	LOC113843049	uncharacterized LOC113843049
XR_003496010.1	0.166666667	0.006666667	-4.64385619	0.00125404	0.005045297	LOC110352196	uncharacterized LOC110352196%2C transcript variant X2
XR_003499180.1	0.263333333	0.001	-8.040746342	0.001255737	0.005047432	LOC110351253	uncharacterized LOC110351253
XR_002398564.2	0.346666667	0.001	-8.437405312	0.001281687	0.005142191	LOC110351306	uncharacterized LOC110351306
XR_003501181.1	0.006666667	0.22	5.044394119	0.001291503	0.005176775	LOC113845539	uncharacterized LOC113845539%2C transcript variant X1
XR_001190035.3	0.203333333	0.013333333	-3.930737338	0.00136573	0.005469236	LOC106016928	uncharacterized LOC106016928%2C transcript variant X2
XR_003496382.1	0.33	0.043333333	-2.928916902	0.001408825	0.005631398	LOC113843198	uncharacterized LOC113843198
XR_003494406.1	0.18	0.016666667	-3.432959407	0.001426527	0.005691644	LOC113842111	uncharacterized LOC113842111
XR_001193197.3	0.213333333	0.001	-7.736965594	0.001449614	0.005773117	LOC106018814	uncharacterized LOC106018814%2C transcript variant X1
XR_003497474.1	0.143333333	0.703333333	2.294834434	0.001459783	0.00580294	LOC106014475	uncharacterized LOC106014475
XR_002400005.2	0.023333333	0.15	2.684498174	0.001476617	0.005859098	LOC101794992	uncharacterized LOC101794992%2C transcript variant X9
MSTRG.2348.4	0.906666667	0.043333333	-4.387023123	0.001495627	0.005929093	-	-
XR_001187167.3	0.053333333	0.003333333	-4	0.001535853	0.006082989	LOC106015263	uncharacterized LOC106015263

XR_002399062.2	0.353333333	0.013333333	-4.727920455	0.001544255	0.006110677	LOC110351520	uncharacterized LOC110351520
MSTRG.11751.2	0.243333333	1.106666667	2.185214872	0.001572725	0.006211978	-	-
XR_003492610.1	0.41	0.08	-2.357552005	0.001580582	0.00623732	LOC110351673	uncharacterized LOC110351673%2C transcript variant X2
XR_003496601.1	0.066666667	0.001	-6.058893689	0.001586668	0.00624994	LOC101791220	uncharacterized LOC101791220%2C transcript variant X3
XR_002404482.2	0.113333333	0.026666667	-2.087462841	0.001616256	0.006360701	LOC110353695	uncharacterized LOC110353695
XR_002405512.2	0.566666667	0.023333333	-4.602036014	0.001633304	0.006416129	LOC101799390	uncharacterized LOC101799390%2C transcript variant X3
XR_002404953.2	0.1	0.013333333	-2.906890596	0.001649518	0.006468085	LOC110353849	uncharacterized LOC110353849
XR_003496537.1	0.336666667	0.02	-4.073248982	0.001679724	0.006568678	LOC113843291	uncharacterized LOC113843291
XR_002400932.2	0.053333333	0.001	-5.736965594	0.001721191	0.006706602	LOC110352259	uncharacterized LOC110352259%2C transcript variant X2
XR_003498261.1	0.24	0.001	-7.906890596	0.001762759	0.00685623	LOC113844086	uncharacterized LOC113844086%2C transcript variant X2
XR_003495821.1	0.243333333	0.01	-4.604862058	0.00178201	0.006918673	LOC113842909	uncharacterized LOC113842909
XR_003497534.1	0.33	0.063333333	-2.381429107	0.001803178	0.006994587	LOC113843779	uncharacterized LOC113843779
XR_002405947.2	0.136666667	0.001	-7.094517599	0.001811989	0.007022469	LOC106019753	uncharacterized LOC106019753%2C transcript variant X1
XR_001191952.2	0.356666667	0.013333333	-4.741466986	0.001831943	0.007093453	LOC106018082	uncharacterized LOC106018082
XR_003497565.1	0.22	0.013333333	-4.044394119	0.00183693	0.007102628	LOC101802970	uncharacterized LOC101802970%2C transcript variant X3
XR_002399630.2	0.126666667	0.026666667	-2.247927513	0.001845253	0.00712587	LOC101798124	uncharacterized LOC101798124%2C transcript variant X3
XR_003495428.1	0.016666667	0.156666667	3.232660757	0.001871643	0.007214907	LOC110354321	uncharacterized LOC110354321%2C transcript variant X1

XR_002402577.2	0.001	0.176666667	7.464886049	0.001880424	0.007242308	LOC110352878	uncharacterized LOC110352878
XR_003492619.1	0.276666667	0.016666667	-4.053111336	0.001918305	0.007381639	LOC113839934	uncharacterized LOC113839934
MSTRG.16981.1	0.403333333	0.001	-8.655828831	0.00192392	0.007394451	-	-
XR_003499438.1	0.016666667	0.283333333	4.087462841	0.001925924	0.007394451	LOC113844708	uncharacterized LOC113844708%2C transcript variant X2
XR_001185755.3	0.1	0.001	-6.64385619	0.001928467	0.007394451	LOC106014431	uncharacterized LOC106014431
XR_003498805.1	0.206666667	0.001	-7.691161905	0.001928467	0.007394451	LOC106019442	uncharacterized LOC106019442%2C transcript variant X4
XR_002403213.2	0.423333333	0.013333333	-4.988684687	0.001953831	0.007485077	LOC106017699	uncharacterized LOC106017699
XR_001190039.3	0.05	0.001	-5.64385619	0.001979601	0.007577092	LOC106016930	uncharacterized LOC106016930%2C transcript variant X2
XR_001191013.3	0.153333333	0.02	-2.938599455	0.002053768	0.007840176	LOC106017519	uncharacterized LOC106017519
XR_002405598.2	0.076666667	0.001	-6.26052755	0.002076943	0.007921662	LOC106019518	uncharacterized LOC106019518%2C transcript variant X1
XR_003495535.1	0.336666667	0.033333333	-3.336283388	0.002080156	0.007923985	LOC106019086	uncharacterized LOC106019086
XR_002402841.2	0.063333333	0.001	-5.984893108	0.002165894	0.008224698	LOC110352971	uncharacterized LOC110352971
XR_003492473.1	0.083333333	0.57	2.773996325	0.002206493	0.008371523	LOC113839841	uncharacterized LOC113839841
XR_003492321.1	0.29	0.04	-2.857980995	0.002235081	0.008472563	LOC113839727	uncharacterized LOC113839727
XR_003495344.1	0.16	0.001	-7.321928095	0.002241544	0.008489629	LOC106015454	uncharacterized LOC106015454%2C transcript variant X1
XR_003499613.1	0.006666667	0.056666667	3.087462841	0.002253501	0.008527454	LOC113844759	uncharacterized LOC113844759
XR_003499807.1	0.001	0.016666667	4.058893689	0.002321961	0.008763532	LOC110351232	uncharacterized LOC110351232%2C transcript variant X2
XR_002399985.2	0.083333333	0.383333333	2.201633861	0.002350275	0.008857891	LOC106015643	uncharacterized LOC106015643
XR_003493438.1	0.85	0.123333333	-2.784900071	0.002351055	0.008857891	LOC113840706	uncharacterized LOC113840706%2C

							transcript variant X11
XR_003499515.1	0.01	0.27	4.754887502	0.002358624	0.008870966	LOC113844732	uncharacterized LOC113844732%2C transcript variant X1
XR_003498621.1	0.07	0.013333333	-2.392317423	0.002362805	0.008878977	LOC113844221	uncharacterized LOC113844221
XR_002399995.2	0.013333333	0.163333333	3.614709844	0.002391201	0.008972604	LOC101794992	uncharacterized LOC101794992%2C transcript variant X2
XR_003497952.1	0.166666667	0.001	-7.380821784	0.002413922	0.00903968	LOC113843960	uncharacterized LOC113843960%2C transcript variant X2
XR_001193302.3	0.113333333	0.003333333	-5.087462841	0.002465816	0.009218062	LOC106018881	uncharacterized LOC106018881
XR_002403533.2	0.096666667	0.006666667	-3.857980995	0.002494175	0.009316034	LOC110353257	uncharacterized LOC110353257
XR_002398777.2	0.26	0.02	-3.700439718	0.002507854	0.00935099	LOC110351389	uncharacterized LOC110351389
XR_002401479.2	0.226666667	0.001	-7.824428435	0.002513234	0.009362987	LOC106016692	uncharacterized LOC106016692%2C transcript variant X1
XR_003496236.1	0.136666667	0.013333333	-3.357552005	0.00262538	0.009763977	LOC101798653	uncharacterized LOC101798653%2C transcript variant X1
XR_001194263.3	0.043333333	0.001	-5.437405312	0.002632916	0.009775207	LOC101799512	uncharacterized LOC101799512%2C transcript variant X6
XR_003495145.1	0.356666667	0.023333333	-3.934112064	0.002643432	0.009805842	LOC106018380	uncharacterized LOC106018380%2C transcript variant X1
XR_003501377.1	0.043333333	0.001	-5.437405312	0.002648497	0.009816219	LOC113845638	uncharacterized LOC113845638%2C transcript variant X1
XR_003497570.1	0.103333333	0.001	-6.691161905	0.002657748	0.00982891	LOC106018705	uncharacterized LOC106018705%2C transcript variant X2
XR_001191910.3	0.21	0.001	-7.714245518	0.002658174	0.00982891	LOC106018057	uncharacterized LOC106018057
XR_002406125.2	0.013333333	0.056666667	2.087462841	0.002658733	0.00982891	LOC110354323	uncharacterized LOC110354323
XR_003496453.1	0.156666667	0.001	-7.291554446	0.002691825	0.009934281	LOC110352189	uncharacterized LOC110352189%2C

							transcript variant X2
XR_003492849.1	0.763333333	0.183333333	-2.057844075	0.002732033	0.010056948	LOC113840117	uncharacterized LOC113840117
XR_003500014.1	0.003333333	0.183333333	5.781359714	0.002741057	0.010078662	LOC101802362	uncharacterized LOC101802362%2C transcript variant X9
XR_002400463.2	0.063333333	0.006666667	-3.247927513	0.00276985	0.010170211	LOC106015927	uncharacterized LOC106015927%2C transcript variant X1
XR_003495766.1	0.746666667	0.02	-5.222392421	0.002787297	0.010214488	LOC110352593	uncharacterized LOC110352593%2C transcript variant X2
XR_002398851.2	0.083333333	0.001	-6.380821784	0.00285532	0.010430953	LOC106014736	uncharacterized LOC106014736%2C transcript variant X4
XR_003494971.1	0.133333333	0.001	-7.058893689	0.002892567	0.010540339	LOC113842448	uncharacterized LOC113842448
XR_002399835.2	0.196666667	0.02	-3.297680549	0.0029216	0.010632909	LOC110351843	uncharacterized LOC110351843
XR_003501157.1	0.356666667	0.086666667	-2.041027268	0.002922883	0.010632909	LOC113845529	uncharacterized LOC113845529
XR_003499736.1	0.46	0.033333333	-3.786596362	0.002970199	0.010795962	LOC113844803	uncharacterized LOC113844803%2C transcript variant X1
MSTRG.5360.1	1	0.093333333	-3.421463768	0.00298927	0.010856166	-	-
XR_003497837.1	0.173333333	0.01	-4.115477217	0.003078903	0.011162959	LOC110352928	uncharacterized LOC110352928
XR_003494623.1	0.816666667	0.153333333	-2.413075983	0.003160995	0.011422327	LOC113842292	uncharacterized LOC113842292
XR_002399526.2	0.16	0.036666667	-2.125530882	0.003196587	0.011531686	LOC106015288	uncharacterized LOC106015288
XR_001187193.3	0.11	0.001	-6.781359714	0.003262982	0.011751622	LOC106015281	uncharacterized LOC106015281
XR_001194622.3	0.026666667	0.001	-4.736965594	0.00333987	0.011998587	LOC106019644	uncharacterized LOC106019644
XR_001185921.3	0.086666667	0.003333333	-4.700439718	0.003394411	0.012157712	LOC106014541	uncharacterized LOC106014541%2C transcript variant X1
XR_001187732.3	0.176666667	0.01	-4.142957954	0.003395397	0.012157712	LOC101803363	uncharacterized LOC101803363%2C transcript variant X2

XR_002402031.2	0.026666667	0.396666667	3.894817763	0.003408332	0.012193942	LOC110352677	uncharacterized LOC110352677
XR_003492388.1	0.05	0.001	-5.64385619	0.00343868	0.012272092	LOC106017370	uncharacterized LOC106017370%2C transcript variant X2
XR_001186693.3	0.016666667	0.001	-4.058893689	0.003461609	0.012343743	LOC106014970	uncharacterized LOC106014970
XR_001190899.3	0.17	0.001	-7.409390936	0.003509512	0.012504261	LOC101794946	uncharacterized LOC101794946%2C transcript variant X1
XR_002398562.2	0.15	0.006666667	-4.491853096	0.003541368	0.012607388	LOC106014525	uncharacterized LOC106014525
XR_002403481.2	0.096666667	0.001	-6.594946589	0.003597951	0.01278779	LOC101801686	uncharacterized LOC101801686%2C transcript variant X2
XR_003500527.1	0.053333333	0.001	-5.736965594	0.003649943	0.012951313	LOC101794131	uncharacterized LOC101794131%2C transcript variant X24
XR_002402711.2	0.113333333	0.01	-3.502500341	0.003784779	0.013396816	LOC106017462	uncharacterized LOC106017462%2C transcript variant X1
XR_001186503.3	0.18	0.016666667	-3.432959407	0.003859831	0.013629046	LOC106014868	uncharacterized LOC106014868%2C transcript variant X6
XR_002401575.2	0.18	0.001	-7.491853096	0.003924053	0.013844519	LOC106016770	uncharacterized LOC106016770%2C transcript variant X2
XR_003500382.1	0.03	0.001	-4.906890596	0.003941048	0.013893158	LOC110352140	uncharacterized LOC110352140%2C transcript variant X3
XR_003496303.1	0.01	0.123333333	3.624490865	0.003956775	0.013937248	LOC106015647	uncharacterized LOC106015647%2C transcript variant X23
XR_002402236.2	0.336666667	0.063333333	-2.410283969	0.003995611	0.014051178	LOC110352746	uncharacterized LOC110352746
XR_001188942.3	0.2	0.02	-3.321928095	0.004061057	0.014258164	LOC106016322	uncharacterized LOC106016322
XR_002404498.2	0.103333333	0.001	-6.691161905	0.004270139	0.01493169	LOC110353704	uncharacterized LOC110353704
XR_001189661.3	0.006666667	0.17	4.672425342	0.004285521	0.014973382	LOC106016741	uncharacterized LOC106016741%2C transcript variant X1

XR_003496624.1	0.133333333	0.006666667	-4.321928095	0.004291005	0.014980453	LOC113843334	uncharacterized LOC113843334
XR_002403565.2	0.016666667	0.001	-4.058893689	0.004298355	0.014994021	LOC110353268	uncharacterized LOC110353268%2C transcript variant X2
XR_003499722.1	0.486666667	0.001	-8.926790153	0.004313699	0.014999198	LOC113844791	uncharacterized LOC113844791%2C transcript variant X3
XR_003498048.1	0.033333333	0.001	-5.058893689	0.004468352	0.015499598	LOC113843986	uncharacterized LOC113843986
XR_003500670.1	0.04	0.003333333	-3.584962501	0.004485135	0.015545355	LOC113845238	uncharacterized LOC113845238
XR_001187232.3	0.07	0.01	-2.807354922	0.004554148	0.015771924	LOC106015301	uncharacterized LOC106015301
XR_003498691.1	0.033333333	0.001	-5.058893689	0.004634407	0.016037048	LOC106019447	uncharacterized LOC106019447%2C transcript variant X3
XR_003496378.1	0.04	0.22	2.459431619	0.004750814	0.016400538	LOC106018734	uncharacterized LOC106018734
XR_003493939.1	0.786666667	0.113333333	-2.795180208	0.004844488	0.016700872	LOC113841382	uncharacterized LOC113841382
MSTRG.8955.3	0.47	0.001	-8.876516947	0.004845529	0.016700872	-	-
XR_003496988.1	0.023333333	0.001	-4.544320516	0.004852174	0.016710471	LOC106016054	uncharacterized LOC106016054%2C transcript variant X6
XR_003500837.1	0.053333333	0.003333333	-4	0.004866624	0.016746913	LOC113845324	uncharacterized LOC113845324%2C transcript variant X1
XR_003497420.1	0.016666667	0.086666667	2.378511623	0.004905168	0.016826047	LOC113843713	uncharacterized LOC113843713
XR_003500988.1	0.05	0.456666667	3.191141487	0.004925006	0.016867368	LOC113845435	uncharacterized LOC113845435
XR_003496035.1	0.043333333	0.001	-5.437405312	0.004930306	0.01687217	LOC110353088	uncharacterized LOC110353088%2C transcript variant X12
MSTRG.7600.1	0.17	0.006666667	-4.672425342	0.004950471	0.016927795	-	-
XR_003501168.1	0.06	0.286666667	2.256339753	0.005010804	0.017120575	LOC113845534	uncharacterized LOC113845534
XR_001187209.3	0.113333333	0.01	-3.502500341	0.005016559	0.017126724	LOC106015290	uncharacterized LOC106015290
XR_002406455.2	0.123333333	0.001	-6.94641896	0.00503782	0.017181957	LOC110354457	uncharacterized LOC110354457

XR_003496358.1	0.173333333	0.001	-7.437405312	0.005076019	0.017288818	LOC106020128	uncharacterized LOC106020128%2C transcript variant X6
XR_003496099.1	0.07	0.001	-6.129283017	0.005088879	0.01731899	LOC110352446	uncharacterized LOC110352446%2C transcript variant X3
XR_001193914.3	0.046666667	0.001	-5.544320516	0.005093408	0.017320787	LOC101799133	uncharacterized LOC101799133
XR_002400437.2	0.1	0.6	2.584962501	0.005145143	0.017469272	LOC106015915	uncharacterized LOC106015915%2C transcript variant X1
XR_002402495.2	0.753333333	0.001	-9.557144557	0.005162957	0.017516018	LOC106017298	uncharacterized LOC106017298
XR_003501021.1	0.001	0.08	6.321928095	0.005177569	0.017551838	LOC113845454	uncharacterized LOC113845454
XR_002399903.2	0.096666667	0.003333333	-4.857980995	0.005186331	0.017567783	LOC110351882	uncharacterized LOC110351882
XR_003496263.1	0.073333333	0.383333333	2.386058432	0.005204594	0.017602099	LOC101794856	uncharacterized LOC101794856%2C transcript variant X3
XR_002399146.2	0.163333333	0.01	-4.029747343	0.005216927	0.017630037	LOC106014982	uncharacterized LOC106014982
XR_003497044.1	0.72	0.026666667	-4.754887502	0.005424602	0.018260578	LOC110352979	uncharacterized LOC110352979
XR_003496246.1	0.196666667	0.026666667	-2.882643049	0.005432136	0.018271729	LOC110352708	uncharacterized LOC110352708%2C transcript variant X10
XR_002403044.2	0.001	0.32	8.321928095	0.005463948	0.018350218	LOC110353058	uncharacterized LOC110353058
XR_002399195.2	0.053333333	0.001	-5.736965594	0.00555953	0.018656748	LOC106015034	uncharacterized LOC106015034
MSTRG.9522.1	0.936666667	0.226666667	-2.046963479	0.005568124	0.018671116	-	-
XR_003498171.1	0.07	0.006666667	-3.392317423	0.005836182	0.0195246	LOC113844045	uncharacterized LOC113844045
MSTRG.15447.1	0.001	1.09	10.09011242	0.005906161	0.019713007	-	-
XR_003494886.1	1.87	0.356666667	-2.390389974	0.005913348	0.019721789	LOC113842394	uncharacterized LOC113842394
XR_002401730.2	0.15	0.001	-7.22881869	0.005933387	0.019773388	LOC101802172	uncharacterized LOC101802172%2C transcript variant X2
XR_003494372.1	0.533333333	0.001	-9.058893689	0.005992956	0.019941204	LOC113842067	uncharacterized LOC113842067%2C

							transcript variant X2
XR_003498709.1	0.246666667	0.01	-4.624490865	0.006027389	0.020040374	LOC110352747	uncharacterized LOC110352747
XR_001189136.3	0.136666667	0.001	-7.094517599	0.006113086	0.020292184	LOC106016418	uncharacterized LOC106016418
MSTRG.8206.2	0.606666667	0.001	-9.244760234	0.006114518	0.020292184	-	-
XR_002398350.2	0.001	0.23	7.845490051	0.006163968	0.020400473	LOC106014375	uncharacterized LOC106014375%2C transcript variant X4
XR_001192847.3	0.096666667	0.013333333	-2.857980995	0.006172271	0.020412347	LOC106018631	uncharacterized LOC106018631
XR_001193525.3	0.103333333	0.01	-3.36923381	0.006185265	0.020439706	LOC106019026	uncharacterized LOC106019026
XR_001187665.3	0.036666667	0.001	-5.196397213	0.006191247	0.020443867	LOC106015555	uncharacterized LOC106015555%2C transcript variant X1
XR_003499893.1	0.033333333	0.28	3.070389328	0.006227432	0.020532028	LOC113844899	uncharacterized LOC113844899
XR_002398929.2	0.3	0.033333333	-3.169925001	0.006287276	0.020713559	LOC101798301	uncharacterized LOC101798301%2C transcript variant X13
XR_002400372.2	0.053333333	0.001	-5.736965594	0.006302588	0.020748215	LOC110352044	uncharacterized LOC110352044
XR_001192311.2	0.17	0.03	-2.502500341	0.006359597	0.020906186	LOC106018298	uncharacterized LOC106018298
XR_003498156.1	0.063333333	0.001	-5.984893108	0.006360233	0.020906186	LOC106016400	uncharacterized LOC106016400%2C transcript variant X11
XR_002399524.2	0.153333333	0.01	-3.938599455	0.006527999	0.021441358	LOC106015283	uncharacterized LOC106015283%2C transcript variant X3
XR_002400639.2	0.096666667	0.001	-6.594946589	0.006565057	0.021530535	LOC110352150	uncharacterized LOC110352150
XR_003496885.1	0.263333333	0.001	-8.040746342	0.006574064	0.021543619	LOC113843526	uncharacterized LOC113843526
XR_003498700.1	0.83	0.076666667	-3.436439976	0.006607501	0.021620462	LOC113844267	uncharacterized LOC113844267
XR_003498169.1	0.046666667	0.22	2.237039197	0.006804662	0.022215221	LOC113844044	uncharacterized LOC113844044
XR_002399144.2	0.283333333	0.001	-8.14635653	0.006912357	0.022532826	LOC110351546	uncharacterized LOC110351546
XR_003494285.1	0.52	0.08	-2.700439718	0.007186218	0.023337688	LOC113841900	uncharacterized LOC113841900

XR_002405662.2	0.233333333	0.001	-7.866248611	0.007280491	0.023590754	LOC106019549	uncharacterized LOC106019549%2C transcript variant X2
XR_003494296.1	0.346666667	0.001	-8.437405312	0.007290295	0.023604853	LOC113841920	uncharacterized LOC113841920%2C transcript variant X2
MSTRG.8299.9	0.116666667	0.966666667	3.050626073	0.007355176	0.023797128	-	-
MSTRG.17302.1	0.356666667	0.001	-8.478432581	0.007372894	0.023836639	-	-
XR_002400343.2	0.093333333	0.02	-2.222392421	0.007429073	0.02400034	LOC110352022	uncharacterized LOC110352022
XR_003501341.1	0.08	0.013333333	-2.584962501	0.007532709	0.024317001	LOC113845615	uncharacterized LOC113845615
MSTRG.3201.1	3.516666667	0.233333333	-3.913744267	0.007552231	0.024361852	-	-
XR_001192642.3	0.266666667	0.056666667	-2.234465254	0.00755846	0.024363793	LOC106018502	uncharacterized LOC106018502
XR_003500062.1	0.073333333	0.001	-6.196397213	0.007618335	0.024520277	LOC101791976	uncharacterized LOC101791976%2C transcript variant X9
XR_002402410.2	0.11	0.013333333	-3.044394119	0.007651651	0.024609212	LOC101790147	uncharacterized LOC101790147%2C transcript variant X6
XR_003497494.1	0.093333333	0.001	-6.544320516	0.007666272	0.02463793	LOC101802183	uncharacterized LOC101802183
XR_001188509.3	0.22	0.053333333	-2.044394119	0.007776158	0.024972543	LOC106016076	uncharacterized LOC106016076
XR_003497481.1	0.12	0.001	-6.906890596	0.00779501	0.024996	LOC110351804	uncharacterized LOC110351804%2C transcript variant X2
XR_003500376.1	0.146666667	0.001	-7.196397213	0.007801726	0.024999016	LOC113845108	uncharacterized LOC113845108
XR_003499509.1	0.763333333	0.003333333	-7.839203788	0.007814285	0.025020739	LOC113844731	uncharacterized LOC113844731%2C transcript variant X7
XR_003500653.1	0.673333333	0.036666667	-4.198779864	0.00792827	0.025329507	LOC113845231	uncharacterized LOC113845231
XR_003495827.1	0.296666667	0.016666667	-4.153805336	0.007997914	0.025514348	LOC113842914	uncharacterized LOC113842914
XR_003494612.1	0.016666667	0.126666667	2.925999419	0.008085042	0.025735403	LOC113842286	uncharacterized LOC113842286%2C transcript variant X1

XR_002401914.2	0.18	0.016666667	-3.432959407	0.008092656	0.025740713	LOC106016928	uncharacterized LOC106016928%2C transcript variant X1
XR_003496160.1	0.416666667	0.093333333	-2.158429363	0.008170673	0.025937162	LOC113843127	uncharacterized LOC113843127
XR_003496379.1	0.026666667	0.16	2.584962501	0.008173961	0.025937162	LOC113843197	uncharacterized LOC113843197
XR_003499458.1	0.31	0.073333333	-2.079727192	0.008178384	0.025937162	LOC113844716	uncharacterized LOC113844716%2C transcript variant X3
XR_002406514.2	0.05	0.006666667	-2.906890596	0.008254344	0.026158897	LOC110354480	uncharacterized LOC110354480
XR_002399153.2	0.086666667	0.003333333	-4.700439718	0.008374231	0.026519419	LOC106014987	uncharacterized LOC106014987%2C transcript variant X1
XR_003499240.1	0.033333333	0.001	-5.058893689	0.00857176	0.027086363	LOC106019826	uncharacterized LOC106019826
XR_002403649.2	0.023333333	0.096666667	2.050626073	0.00857203	0.027086363	LOC106018095	uncharacterized LOC106018095
XR_001186046.3	0.08	0.001	-6.321928095	0.00865767	0.027337019	LOC106014612	uncharacterized LOC106014612%2C transcript variant X1
XR_002404478.2	0.67	0.05	-3.744161096	0.008706919	0.027472487	LOC101800325	uncharacterized LOC101800325%2C transcript variant X2
XR_003496968.1	0.113333333	0.013333333	-3.087462841	0.00875098	0.027566785	LOC106016071	uncharacterized LOC106016071
XR_003498066.1	0.096666667	0.02	-2.273018494	0.008783374	0.027593052	LOC110352602	uncharacterized LOC110352602%2C transcript variant X3
XR_003497206.1	0.02	0.001	-4.321928095	0.008804565	0.027639566	LOC113843623	uncharacterized LOC113843623
XR_003497895.1	0.043333333	0.003333333	-3.700439718	0.008814363	0.027650275	LOC113843937	uncharacterized LOC113843937
XR_001189442.3	0.193333333	0.006666667	-4.857980995	0.008951332	0.027972909	LOC106016617	uncharacterized LOC106016617
XR_002404557.2	0.05	0.003333333	-3.906890596	0.008979206	0.028025223	LOC106018801	uncharacterized LOC106018801%2C transcript variant X3
XR_003496299.1	0.001	0.056666667	5.824428435	0.009072015	0.028294492	LOC106015647	uncharacterized LOC106015647%2C transcript variant X19
XR_002399314.2	0.1	0.001	-6.64385619	0.009094675	0.028344742	LOC106015131	uncharacterized LOC106015131%2C

							transcript variant X3
XR_001187517.3	0.243333333	0.016666667	-3.867896464	0.009143047	0.028454529	LOC106015459	uncharacterized LOC106015459%2C transcript variant X1
XR_003494839.1	0.13	0.006666667	-4.285402219	0.009280127	0.028818988	LOC110352526	uncharacterized LOC110352526%2C transcript variant X4
XR_003497801.1	0.04	0.006666667	-2.584962501	0.009335628	0.02897056	LOC113843890	uncharacterized LOC113843890%2C transcript variant X2
XR_001187868.3	0.296666667	0.03	-3.30580843	0.009364753	0.029040127	LOC106015673	uncharacterized LOC106015673
XR_003501393.1	0.19	0.001	-7.569855608	0.0094911	0.029366298	LOC106016121	uncharacterized LOC106016121%2C transcript variant X2
XR_003496877.1	0.543333333	0.001	-9.085693748	0.00949707	0.029366298	LOC113843521	uncharacterized LOC113843521%2C transcript variant X2
XR_003497996.1	0.033333333	0.003333333	-3.321928095	0.009653763	0.029809593	LOC101800399	uncharacterized LOC101800399
XR_003499552.1	0.073333333	0.001	-6.196397213	0.009674959	0.029852386	LOC106016478	uncharacterized LOC106016478%2C transcript variant X4
XR_001186000.3	0.093333333	0.001	-6.544320516	0.00975251	0.030070238	LOC106014589	uncharacterized LOC106014589
XR_002400443.2	0.001	0.033333333	5.058893689	0.009967759	0.030646611	LOC106015916	uncharacterized LOC106015916%2C transcript variant X2

Description: Sample: id: lncRNA id; IMP0-1_count: lncRNA count value for SCP0-1; SCP0-4_count: lncRNA count value for SCP0-4; log2(FC): logarithmic value of the multiplicity of difference in FPKM between samples IMP0 and IMP4, bottomed by 2; P_value: significance P-value; FDR: P-value after BH correction; Symbol: lncRNA name; Description: lncRNA annotation.
