# Supplementary material for: Identification of genes and long non-coding RNAs for intramuscular and subcutaneous fat deposition in ducks by transcriptome analysis
Source: Anim Biosci. 2025 Aug 12;39(1):250268. doi: 10.5713/ab.25.0268 (PMC12754461; doi:10.5713/ab.25.0268)
Supplement: Supplementary file 9 [file ab-25-0268-Supplementary-9.pdf]

Supplement 9. A lncRNA-mRNA co-expression network of the IMP-0-vs-IMP-4 group

lncRNA_id	lncRNA_Symbol	GeneID	Gene_Symbol	cor	p_value	KEGG_B_class
MSTRG.11484.2	-	ncbi_101789855	Gpd1	0.953232151	0.00000163	Lipid metabolism
MSTRG.11490.3	-	ncbi_101789855	Gpd1	0.972234703	0.000000124	Lipid metabolism
MSTRG.16843.3	-	ncbi_101789855	Gpd1	0.96521909	0.000000378	Lipid metabolism
MSTRG.2341.4	-	ncbi_101789855	Gpd1	0.977912431	3.99E-08	Lipid metabolism
MSTRG.5641.4	-	ncbi_101789855	Gpd1	0.956121532	0.00000119	Lipid metabolism
XR_001192289.3	LOC106018283	ncbi_101789855	Gpd1	0.954810804	0.00000138	Lipid metabolism
XR_003494155.1	LOC113841719	ncbi_101789855	Gpd1	0.968687651	0.000000225	Lipid metabolism
XR_003495978.1	LOC110352611	ncbi_101789855	Gpd1	0.95669539	0.00000112	Lipid metabolism
XR_003498753.1	LOC113844290	ncbi_101789855	Gpd1	0.973068925	0.000000107	Lipid metabolism
MSTRG.10341.18	-	ncbi_101790851	CHKA	0.962190664	0.000000571	Global and overview maps;Lipid metabolism
MSTRG.10914.13	-	ncbi_101790851	CHKA	0.978279763	3.67E-08	Global and overview maps;Lipid metabolism
MSTRG.10914.14	-	ncbi_101790851	CHKA	0.9639485	0.000000451	Global and overview maps;Lipid metabolism
MSTRG.16028.1	-	ncbi_101790851	CHKA	0.95686719	0.00000109	Global and overview maps;Lipid metabolism
MSTRG.4635.4	-	ncbi_101790851	CHKA	0.988859661	1.33E-09	Global and overview maps;Lipid metabolism
MSTRG.852.1	-	ncbi_101790851	CHKA	0.963117276	0.000000505	Global and overview maps;Lipid metabolism
MSTRG.9008.1	-	ncbi_101790851	CHKA	0.969475725	0.000000198	Global and overview maps;Lipid

						metabolism
XR_002398923.2	LOC101798301	ncbi_101790851	CHKA	0.966319779	0.000000323	Global and overview maps;Lipid metabolism
XR_002402383.2	LOC110352801	ncbi_101790851	CHKA	0.950497471	0.00000215	Global and overview maps;Lipid metabolism
XR_002402646.2	LOC101794014	ncbi_101790851	CHKA	0.960241043	0.000000732	Global and overview maps;Lipid metabolism
XR_002404953.2	LOC110353849	ncbi_101790851	CHKA	0.960263076	0.00000073	Global and overview maps;Lipid metabolism
XR_003492632.1	LOC106018295	ncbi_101790851	CHKA	0.97512456	7.19E-08	Global and overview maps;Lipid metabolism
XR_003495202.1	LOC113842645	ncbi_101790851	CHKA	0.959579635	0.000000794	Global and overview maps;Lipid metabolism
XR_003495207.1	LOC113842649	ncbi_101790851	CHKA	0.962787139	0.000000528	Global and overview maps;Lipid metabolism
XR_003497162.1	LOC113843598	ncbi_101790851	CHKA	0.957451174	0.00000102	Global and overview maps;Lipid metabolism
XR_003497745.1	LOC110353307	ncbi_101790851	CHKA	0.97590084	6.15E-08	Global and overview maps;Lipid metabolism
XR_003499790.1	LOC101804048	ncbi_101790851	CHKA	0.964270643	0.000000432	Global and overview maps;Lipid metabolism
XR_003499961.1	LOC110352806	ncbi_101790851	CHKA	0.96923017	0.000000206	Global and overview maps;Lipid metabolism
XR_003499966.1	LOC110352806	ncbi_101790851	CHKA	0.964400455	0.000000424	Global and overview maps;Lipid metabolism
XR_003500051.1	LOC113844972	ncbi_101790851	CHKA	0.960891325	0.000000675	Global and overview maps;Lipid

						metabolism
XR_003500052.1	LOC113844972	ncbi_101790851	CHKA	0.96947389	0.000000198	Global and overview maps;Lipid metabolism
XR_003500680.1	LOC110352551	ncbi_101790851	CHKA	0.966011064	0.000000337	Global and overview maps;Lipid metabolism
XR_217495.4	LOC101790744	ncbi_101790851	CHKA	0.982451683	1.27E-08	Global and overview maps;Lipid metabolism
MSTRG.10098.1	-	ncbi_101793081	MBOAT1	0.975560909	6.59E-08	Global and overview maps;Lipid metabolism;Lipid metabolism
MSTRG.10100.1	-	ncbi_101793081	MBOAT1	0.961283086	0.000000642	Global and overview maps;Lipid metabolism;Lipid metabolism
MSTRG.10341.18	-	ncbi_101793081	MBOAT1	0.977393829	4.48E-08	Global and overview maps;Lipid metabolism;Lipid metabolism
MSTRG.10341.19	-	ncbi_101793081	MBOAT1	0.954323644	0.00000145	Global and overview maps;Lipid metabolism;Lipid metabolism
MSTRG.10341.20	-	ncbi_101793081	MBOAT1	0.962669292	0.000000536	Global and overview maps;Lipid metabolism;Lipid metabolism
MSTRG.10341.22	-	ncbi_101793081	MBOAT1	0.959527554	0.000000799	Global and overview maps;Lipid metabolism;Lipid metabolism
MSTRG.10914.13	-	ncbi_101793081	MBOAT1	0.975589933	6.55E-08	Global and overview maps;Lipid metabolism;Lipid metabolism
MSTRG.10914.14	-	ncbi_101793081	MBOAT1	0.9601532	0.00000074	Global and overview maps;Lipid metabolism;Lipid metabolism
MSTRG.16028.1	-	ncbi_101793081	MBOAT1	0.968916116	0.000000217	Global and overview maps;Lipid metabolism;Lipid metabolism
MSTRG.2606.1	-	ncbi_101793081	MBOAT1	0.959702048	0.000000782	Global and overview maps;Lipid

						metabolism;Lipid metabolism
MSTRG.4635.4	-	ncbi_101793081	MBOAT1	0.980458845	2.17E-08	Global and overview maps;Lipid metabolism;Lipid metabolism
MSTRG.5135.3	-	ncbi_101793081	MBOAT1	0.955708704	0.00000125	Global and overview maps;Lipid metabolism;Lipid metabolism
MSTRG.5601.1	-	ncbi_101793081	MBOAT1	0.96236008	0.000000559	Global and overview maps;Lipid metabolism;Lipid metabolism
MSTRG.756.3	-	ncbi_101793081	MBOAT1	0.957456952	0.00000102	Global and overview maps;Lipid metabolism;Lipid metabolism
MSTRG.852.1	-	ncbi_101793081	MBOAT1	0.980038582	2.41E-08	Global and overview maps;Lipid metabolism;Lipid metabolism
MSTRG.9008.1	-	ncbi_101793081	MBOAT1	0.984048032	7.92E-09	Global and overview maps;Lipid metabolism;Lipid metabolism
XR_002398923.2	LOC101798301	ncbi_101793081	MBOAT1	0.988791732	1.37E-09	Global and overview maps;Lipid metabolism;Lipid metabolism
XR_002402383.2	LOC110352801	ncbi_101793081	MBOAT1	0.955630256	0.00000126	Global and overview maps;Lipid metabolism;Lipid metabolism
XR_002402646.2	LOC101794014	ncbi_101793081	MBOAT1	0.978531253	3.46E-08	Global and overview maps;Lipid metabolism;Lipid metabolism
XR_002404953.2	LOC110353849	ncbi_101793081	MBOAT1	0.968752903	0.000000223	Global and overview maps;Lipid metabolism;Lipid metabolism
XR_003492182.1	LOC113839646	ncbi_101793081	MBOAT1	0.972718806	0.000000114	Global and overview maps;Lipid metabolism;Lipid metabolism
XR_003492632.1	LOC106018295	ncbi_101793081	MBOAT1	0.982350266	1.31E-08	Global and overview maps;Lipid metabolism;Lipid metabolism
XR_003495202.1	LOC113842645	ncbi_101793081	MBOAT1	0.972783999	0.000000112	Global and overview maps;Lipid

						metabolism;Lipid metabolism
XR_003495207.1	LOC113842649	ncbi_101793081	MBOAT1	0.970470358	0.000000168	Global and overview maps;Lipid metabolism;Lipid metabolism
XR_003495918.1	LOC113843035	ncbi_101793081	MBOAT1	0.958517981	0.000000902	Global and overview maps;Lipid metabolism;Lipid metabolism
XR_003496334.1	LOC113843176	ncbi_101793081	MBOAT1	0.959022338	0.000000849	Global and overview maps;Lipid metabolism;Lipid metabolism
XR_003497745.1	LOC110353307	ncbi_101793081	MBOAT1	0.992892686	1.41E-10	Global and overview maps;Lipid metabolism;Lipid metabolism
XR_003499790.1	LOC101804048	ncbi_101793081	MBOAT1	0.974177269	8.66E-08	Global and overview maps;Lipid metabolism;Lipid metabolism
XR_003499961.1	LOC110352806	ncbi_101793081	MBOAT1	0.982546498	1.24E-08	Global and overview maps;Lipid metabolism;Lipid metabolism
XR_003499966.1	LOC110352806	ncbi_101793081	MBOAT1	0.962584649	0.000000542	Global and overview maps;Lipid metabolism;Lipid metabolism
XR_003500052.1	LOC113844972	ncbi_101793081	MBOAT1	0.970807997	0.000000159	Global and overview maps;Lipid metabolism;Lipid metabolism
XR_003500183.1	LOC106020052	ncbi_101793081	MBOAT1	0.950403645	0.00000217	Global and overview maps;Lipid metabolism;Lipid metabolism
XR_217495.4	LOC101790744	ncbi_101793081	MBOAT1	0.965787816	0.000000349	Global and overview maps;Lipid metabolism;Lipid metabolism
MSTRG.14276.1	-	ncbi_101793285	FABP7	0.988375623	1.64E-09	Endocrine system
MSTRG.2671.3	-	ncbi_101793285	FABP7	0.968031918	0.000000249	Endocrine system
MSTRG.2868.1	-	ncbi_101793285	FABP7	0.989972191	7.85E-10	Endocrine system
MSTRG.338.3	-	ncbi_101793285	FABP7	0.951264751	0.00000199	Endocrine system
MSTRG.4759.1	-	ncbi_101793285	FABP7	0.959499931	0.000000802	Endocrine system

MSTRG.5775.11	-	ncbi_101793285	FABP7	-0.957332465	0.00000104	Endocrine system
MSTRG.8490.1	-	ncbi_101793285	FABP7	0.96475963	0.000000403	Endocrine system
XR_001191013.3	LOC106017519	ncbi_101793285	FABP7	0.974953643	7.44E-08	Endocrine system
XR_002398562.2	LOC106014525	ncbi_101793285	FABP7	0.957416059	0.00000103	Endocrine system
XR_002401163.2	LOC110352334	ncbi_101793285	FABP7	0.98157188	1.62E-08	Endocrine system
XR_002402751.2	LOC110352931	ncbi_101793285	FABP7	0.983169314	1.03E-08	Endocrine system
XR_002402756.2	LOC110352934	ncbi_101793285	FABP7	0.974536821	8.08E-08	Endocrine system
XR_002402765.2	LOC110352941	ncbi_101793285	FABP7	0.957519524	0.00000101	Endocrine system
XR_002403094.2	LOC110353080	ncbi_101793285	FABP7	0.959169621	0.000000834	Endocrine system
XR_003492849.1	LOC113840117	ncbi_101793285	FABP7	0.958116346	0.000000946	Endocrine system
XR_003493825.1	LOC113841183	ncbi_101793285	FABP7	0.983368094	9.75E-09	Endocrine system
XR_003496855.1	LOC113843509	ncbi_101793285	FABP7	-0.973049506	0.000000107	Endocrine system
XR_003497368.1	LOC110352930	ncbi_101793285	FABP7	0.957662502	0.000000998	Endocrine system
XR_003497399.1	LOC110352933	ncbi_101793285	FABP7	0.97503808	7.32E-08	Endocrine system
XR_003498160.1	LOC113844040	ncbi_101793285	FABP7	0.951508764	0.00000195	Endocrine system
MSTRG.10098.1	-	ncbi_101793344	etnppl	0.991584363	3.28E-10	Global and overview maps;Lipid metabolism
MSTRG.10100.1	-	ncbi_101793344	etnppl	0.991622181	3.21E-10	Global and overview maps;Lipid metabolism
MSTRG.10341.18	-	ncbi_101793344	etnppl	0.987697664	2.17E-09	Global and overview maps;Lipid metabolism
MSTRG.10341.19	-	ncbi_101793344	etnppl	0.996824225	2.53E-12	Global and overview maps;Lipid metabolism

MSTRG.10341.20	-	ncbi_101793344	etnppl	0.996837798	2.48E-12	Global and overview maps;Lipid metabolism
MSTRG.10341.21	-	ncbi_101793344	etnppl	0.996936656	2.11E-12	Global and overview maps;Lipid metabolism
MSTRG.10341.22	-	ncbi_101793344	etnppl	0.995072391	2.27E-11	Global and overview maps;Lipid metabolism
MSTRG.16028.1	-	ncbi_101793344	etnppl	0.969636677	0.000000193	Global and overview maps;Lipid metabolism
MSTRG.1816.1	-	ncbi_101793344	etnppl	0.97129871	0.000000146	Global and overview maps;Lipid metabolism
MSTRG.2606.1	-	ncbi_101793344	etnppl	0.984459068	6.96E-09	Global and overview maps;Lipid metabolism
MSTRG.2608.1	-	ncbi_101793344	etnppl	0.977125922	4.75E-08	Global and overview maps;Lipid metabolism
MSTRG.3080.1	-	ncbi_101793344	etnppl	0.98560786	4.75E-09	Global and overview maps;Lipid metabolism
MSTRG.5135.1	-	ncbi_101793344	etnppl	0.980491659	2.15E-08	Global and overview maps;Lipid metabolism
MSTRG.5135.3	-	ncbi_101793344	etnppl	0.993438956	9.47E-11	Global and overview maps;Lipid metabolism
MSTRG.5601.1	-	ncbi_101793344	etnppl	0.961233833	0.000000646	Global and overview maps;Lipid metabolism
MSTRG.5881.2	-	ncbi_101793344	etnppl	0.983743779	8.7E-09	Global and overview maps;Lipid metabolism
MSTRG.6704.2	-	ncbi_101793344	etnppl	0.986450971	3.52E-09	Global and overview maps;Lipid metabolism

MSTRG.6704.4	-	ncbi_101793344	etnppl	0.995739088	1.1E-11	Global and overview maps;Lipid metabolism
MSTRG.7239.1	-	ncbi_101793344	etnppl	0.981267492	1.76E-08	Global and overview maps;Lipid metabolism
MSTRG.7248.1	-	ncbi_101793344	etnppl	0.985734903	4.54E-09	Global and overview maps;Lipid metabolism
MSTRG.756.3	-	ncbi_101793344	etnppl	0.994912505	2.66E-11	Global and overview maps;Lipid metabolism
MSTRG.852.1	-	ncbi_101793344	etnppl	0.984129259	7.72E-09	Global and overview maps;Lipid metabolism
MSTRG.9008.1	-	ncbi_101793344	etnppl	0.987347616	2.5E-09	Global and overview maps;Lipid metabolism
MSTRG.9062.2	-	ncbi_101793344	etnppl	0.981904165	1.48E-08	Global and overview maps;Lipid metabolism
XR_001190983.3	LOC106017488	ncbi_101793344	etnppl	0.968780026	0.000000222	Global and overview maps;Lipid metabolism
XR_001195514.2	LOC106020129	ncbi_101793344	etnppl	0.95970966	0.000000781	Global and overview maps;Lipid metabolism
XR_002398923.2	LOC101798301	ncbi_101793344	etnppl	0.964362103	0.000000426	Global and overview maps;Lipid metabolism
XR_002400249.2	LOC110351990	ncbi_101793344	etnppl	0.965245645	0.000000377	Global and overview maps;Lipid metabolism
XR_002402646.2	LOC101794014	ncbi_101793344	etnppl	0.986884791	2.99E-09	Global and overview maps;Lipid metabolism
XR_002402718.2	LOC106017475	ncbi_101793344	etnppl	0.985396409	5.1E-09	Global and overview maps;Lipid metabolism

XR_002406750.2	LOC106020393	ncbi_101793344	etnppl	0.983691275	8.84E-09	Global and overview maps;Lipid metabolism
XR_003492182.1	LOC113839646	ncbi_101793344	etnppl	0.966835779	0.000000299	Global and overview maps;Lipid metabolism
XR_003492632.1	LOC106018295	ncbi_101793344	etnppl	0.968780895	0.000000222	Global and overview maps;Lipid metabolism
XR_003493079.1	LOC106017632	ncbi_101793344	etnppl	0.972181705	0.000000125	Global and overview maps;Lipid metabolism
XR_003494888.1	LOC113842396	ncbi_101793344	etnppl	0.973143041	0.000000105	Global and overview maps;Lipid metabolism
XR_003494957.1	LOC101799909	ncbi_101793344	etnppl	0.978633052	3.38E-08	Global and overview maps;Lipid metabolism
XR_003494961.1	LOC113842447	ncbi_101793344	etnppl	0.992938056	1.37E-10	Global and overview maps;Lipid metabolism
XR_003495202.1	LOC113842645	ncbi_101793344	etnppl	0.95315466	0.00000164	Global and overview maps;Lipid metabolism
XR_003495207.1	LOC113842649	ncbi_101793344	etnppl	0.970550882	0.000000166	Global and overview maps;Lipid metabolism
XR_003495767.1	LOC110352593	ncbi_101793344	etnppl	0.964495521	0.000000419	Global and overview maps;Lipid metabolism
XR_003495918.1	LOC113843035	ncbi_101793344	etnppl	0.961255174	0.000000644	Global and overview maps;Lipid metabolism
XR_003496334.1	LOC113843176	ncbi_101793344	etnppl	0.989233576	1.12E-09	Global and overview maps;Lipid metabolism
XR_003496598.1	LOC113843320	ncbi_101793344	etnppl	0.951258984	0.000002	Global and overview maps;Lipid metabolism

XR_003497001.1	LOC106017547	ncbi_101793344	etnppl	0.996752423	2.83E-12	Global and overview maps;Lipid metabolism
XR_003497002.1	LOC106017547	ncbi_101793344	etnppl	0.97208935	0.000000127	Global and overview maps;Lipid metabolism
XR_003497551.1	LOC110354397	ncbi_101793344	etnppl	0.971911546	0.000000131	Global and overview maps;Lipid metabolism
XR_003497564.1	LOC101802970	ncbi_101793344	etnppl	0.966879959	0.000000297	Global and overview maps;Lipid metabolism
XR_003497566.1	LOC101802970	ncbi_101793344	etnppl	0.996334893	5.18E-12	Global and overview maps;Lipid metabolism
XR_003497661.1	LOC101798800	ncbi_101793344	etnppl	0.968688876	0.000000225	Global and overview maps;Lipid metabolism
XR_003497745.1	LOC110353307	ncbi_101793344	etnppl	0.984290896	7.34E-09	Global and overview maps;Lipid metabolism
XR_003499790.1	LOC101804048	ncbi_101793344	etnppl	0.971831354	0.000000133	Global and overview maps;Lipid metabolism
XR_003499961.1	LOC110352806	ncbi_101793344	etnppl	0.980668413	2.06E-08	Global and overview maps;Lipid metabolism
XR_003499966.1	LOC110352806	ncbi_101793344	etnppl	0.969381907	0.000000201	Global and overview maps;Lipid metabolism
XR_003500183.1	LOC106020052	ncbi_101793344	etnppl	0.971848724	0.000000133	Global and overview maps;Lipid metabolism
XR_003500722.1	LOC113845266	ncbi_101793344	etnppl	0.979329389	2.87E-08	Global and overview maps;Lipid metabolism
MSTRG.14276.1	-	ncbi_101794817	UGT8	0.956735292	0.00000111	Global and overview maps;Lipid metabolism;Lipid metabolism

MSTRG.3256.4	-	ncbi_101794817	UGT8	-0.961439404	0.000000629	Global and overview maps;Lipid metabolism;Lipid metabolism
MSTRG.8490.1	-	ncbi_101794817	UGT8	0.970064456	0.00000018	Global and overview maps;Lipid metabolism;Lipid metabolism
XR_001191530.3	LOC106017832	ncbi_101794817	UGT8	0.964472842	0.00000042	Global and overview maps;Lipid metabolism;Lipid metabolism
XR_002399903.2	LOC110351882	ncbi_101794817	UGT8	0.956681782	0.00000112	Global and overview maps;Lipid metabolism;Lipid metabolism
XR_002402765.2	LOC110352941	ncbi_101794817	UGT8	0.954697244	0.00000139	Global and overview maps;Lipid metabolism;Lipid metabolism
XR_003493825.1	LOC113841183	ncbi_101794817	UGT8	0.958937803	0.000000858	Global and overview maps;Lipid metabolism;Lipid metabolism
XR_003496239.1	LOC110352708	ncbi_101794817	UGT8	0.960008889	0.000000753	Global and overview maps;Lipid metabolism;Lipid metabolism
XR_003496279.1	LOC101795266	ncbi_101794817	UGT8	-0.960013775	0.000000753	Global and overview maps;Lipid metabolism;Lipid metabolism
XR_003496855.1	LOC113843509	ncbi_101794817	UGT8	-0.950863235	0.00000208	Global and overview maps;Lipid metabolism;Lipid metabolism
XR_003500629.1	LOC113845225	ncbi_101794817	UGT8	-0.958138116	0.000000944	Global and overview maps;Lipid metabolism;Lipid metabolism
MSTRG.11582.4	-	ncbi_101795388	FABP3	0.966487647	0.000000315	Endocrine system
MSTRG.11582.5	-	ncbi_101795388	FABP3	0.954659219	0.0000014	Endocrine system
MSTRG.13804.1	-	ncbi_101795388	FABP3	0.983963325	8.13E-09	Endocrine system
MSTRG.13937.5	-	ncbi_101795388	FABP3	0.992578312	1.75E-10	Endocrine system
MSTRG.17145.1	-	ncbi_101795388	FABP3	0.952158115	0.00000182	Endocrine system
MSTRG.17302.1	-	ncbi_101795388	FABP3	0.961907455	0.000000592	Endocrine system

MSTRG.17651.1	-	ncbi_101795388	FABP3	0.95460597	0.00000141	Endocrine system
MSTRG.3192.37	-	ncbi_101795388	FABP3	0.965744137	0.000000351	Endocrine system
MSTRG.3481.1	-	ncbi_101795388	FABP3	0.951465312	0.00000195	Endocrine system
MSTRG.3481.2	-	ncbi_101795388	FABP3	0.997034701	1.8E-12	Endocrine system
MSTRG.6230.1	-	ncbi_101795388	FABP3	0.978039	3.88E-08	Endocrine system
MSTRG.6356.1	-	ncbi_101795388	FABP3	0.960287665	0.000000728	Endocrine system
MSTRG.6393.1	-	ncbi_101795388	FABP3	0.97259756	0.000000116	Endocrine system
MSTRG.6938.4	-	ncbi_101795388	FABP3	0.978643962	3.38E-08	Endocrine system
MSTRG.7877.3	-	ncbi_101795388	FABP3	0.989477959	9.98E-10	Endocrine system
MSTRG.7980.2	-	ncbi_101795388	FABP3	0.994888294	2.73E-11	Endocrine system
MSTRG.8665.1	-	ncbi_101795388	FABP3	0.988882124	1.31E-09	Endocrine system
XR_001187516.3	LOC106015458	ncbi_101795388	FABP3	0.984484023	6.9E-09	Endocrine system
XR_001189914.3	LOC106016888	ncbi_101795388	FABP3	0.950529859	0.00000215	Endocrine system
XR_002401934.2	LOC110352651	ncbi_101795388	FABP3	0.968099881	0.000000247	Endocrine system
XR_003492257.1	LOC113839689	ncbi_101795388	FABP3	0.960101595	0.000000745	Endocrine system
XR_003495821.1	LOC113842909	ncbi_101795388	FABP3	0.95270646	0.00000172	Endocrine system
XR_003496648.1	LOC113843366	ncbi_101795388	FABP3	0.955655131	0.00000125	Endocrine system
XR_003496781.1	LOC113843470	ncbi_101795388	FABP3	0.98663133	3.29E-09	Endocrine system
XR_003496782.1	LOC113843471	ncbi_101795388	FABP3	0.986457058	3.51E-09	Endocrine system
XR_003496937.1	LOC113843538	ncbi_101795388	FABP3	0.965730093	0.000000351	Endocrine system
XR_003497206.1	LOC113843623	ncbi_101795388	FABP3	0.988725046	1.41E-09	Endocrine system
XR_003497335.1	LOC113843664	ncbi_101795388	FABP3	0.957525377	0.00000101	Endocrine system
XR_003497476.1	LOC110353061	ncbi_101795388	FABP3	0.988837892	1.34E-09	Endocrine system
XR_003498718.1	LOC113844276	ncbi_101795388	FABP3	0.988606181	1.48E-09	Endocrine system
XR_003498785.1	LOC113844315	ncbi_101795388	FABP3	0.961432436	0.00000063	Endocrine system

XR_003498841.1	LOC113844333	ncbi_101795388	FABP3	0.987107025	2.75E-09	Endocrine system
XR_003499613.1	LOC113844759	ncbi_101795388	FABP3	0.962798223	0.000000527	Endocrine system
XR_003500528.1	LOC101794131	ncbi_101795388	FABP3	0.958162999	0.000000941	Endocrine system
XR_003500927.1	LOC113845358	ncbi_101795388	FABP3	0.976489369	5.44E-08	Endocrine system
XR_003501551.1	LOC113845772	ncbi_101795388	FABP3	0.984722613	6.39E-09	Endocrine system
MSTRG.11490.3	-	ncbi_101796497	Pnpla2	0.950496344	0.00000215	Global and overview maps;Lipid metabolism
MSTRG.2341.4	-	ncbi_101796497	Pnpla2	0.975788531	6.29E-08	Global and overview maps;Lipid metabolism
MSTRG.3461.1	-	ncbi_101796497	Pnpla2	0.962218466	0.000000569	Global and overview maps;Lipid metabolism
MSTRG.4554.1	-	ncbi_101796497	Pnpla2	-0.962161816	0.000000573	Global and overview maps;Lipid metabolism
MSTRG.5775.11	-	ncbi_101796497	Pnpla2	0.95295518	0.00000168	Global and overview maps;Lipid metabolism
MSTRG.624.1	-	ncbi_101796497	Pnpla2	0.966700117	0.000000305	Global and overview maps;Lipid metabolism
XR_001192289.3	LOC106018283	ncbi_101796497	Pnpla2	0.963931048	0.000000453	Global and overview maps;Lipid metabolism
XR_002398922.2	LOC101798301	ncbi_101796497	Pnpla2	-0.952133846	0.00000183	Global and overview maps;Lipid metabolism
XR_003493011.1	LOC113840243	ncbi_101796497	Pnpla2	-0.960703373	0.000000691	Global and overview maps;Lipid metabolism
XR_003494155.1	LOC113841719	ncbi_101796497	Pnpla2	0.986596145	3.33E-09	Global and overview maps;Lipid metabolism
XR_003497485.1	LOC113843765	ncbi_101796497	Pnpla2	0.966826004	0.000000299	Global and overview maps;Lipid

						metabolism
MSTRG.13937.5	-	ncbi_101799126	UGT8	0.981508789	1.65E-08	Global and overview maps;Lipid metabolism;Lipid metabolism
MSTRG.3481.2	-	ncbi_101799126	UGT8	0.976443482	5.49E-08	Global and overview maps;Lipid metabolism;Lipid metabolism
MSTRG.6230.1	-	ncbi_101799126	UGT8	0.976786853	5.11E-08	Global and overview maps;Lipid metabolism;Lipid metabolism
MSTRG.6356.1	-	ncbi_101799126	UGT8	0.962220244	0.000000569	Global and overview maps;Lipid metabolism;Lipid metabolism
MSTRG.6393.1	-	ncbi_101799126	UGT8	0.993990631	6.11E-11	Global and overview maps;Lipid metabolism;Lipid metabolism
MSTRG.7877.3	-	ncbi_101799126	UGT8	0.995646988	1.22E-11	Global and overview maps;Lipid metabolism;Lipid metabolism
MSTRG.7980.2	-	ncbi_101799126	UGT8	0.967680976	0.000000263	Global and overview maps;Lipid metabolism;Lipid metabolism
MSTRG.8665.1	-	ncbi_101799126	UGT8	0.990850017	4.97E-10	Global and overview maps;Lipid metabolism;Lipid metabolism
XR_001187516.3	LOC106015458	ncbi_101799126	UGT8	0.96589424	0.000000343	Global and overview maps;Lipid metabolism;Lipid metabolism
XR_003496648.1	LOC113843366	ncbi_101799126	UGT8	0.971683584	0.000000137	Global and overview maps;Lipid metabolism;Lipid metabolism
XR_003496782.1	LOC113843471	ncbi_101799126	UGT8	0.963905855	0.000000454	Global and overview maps;Lipid metabolism;Lipid metabolism
XR_003496937.1	LOC113843538	ncbi_101799126	UGT8	0.989338422	1.07E-09	Global and overview maps;Lipid metabolism;Lipid metabolism
XR_003497476.1	LOC110353061	ncbi_101799126	UGT8	0.960638357	0.000000696	Global and overview maps;Lipid

						metabolism;Lipid metabolism
XR_003498718.1	LOC113844276	ncbi_101799126	UGT8	0.966744432	0.000000303	Global and overview maps;Lipid metabolism;Lipid metabolism
XR_003498841.1	LOC113844333	ncbi_101799126	UGT8	0.968660262	0.000000226	Global and overview maps;Lipid metabolism;Lipid metabolism
XR_003500927.1	LOC113845358	ncbi_101799126	UGT8	0.966729728	0.000000304	Global and overview maps;Lipid metabolism;Lipid metabolism
MSTRG.11051.1	-	ncbi_101799557	PLPP1	0.995215128	1.96E-11	-
MSTRG.3256.4	-	ncbi_101799557	PLPP1	0.965854449	0.000000345	-
MSTRG.4618.1	-	ncbi_101799557	PLPP1	0.955955234	0.00000121	-
MSTRG.7978.1	-	ncbi_101799557	PLPP1	0.97786469	4.03E-08	-
MSTRG.8536.1	-	ncbi_101799557	PLPP1	-0.968990495	0.000000214	-
XR_001188103.2	LOC106015815	ncbi_101799557	PLPP1	0.979256513	2.92E-08	-
XR_002398969.2	LOC106014835	ncbi_101799557	PLPP1	0.965535724	0.000000361	-
XR_002400766.2	LOC110352189	ncbi_101799557	PLPP1	0.966820421	0.0000003	-
XR_002401371.2	LOC110352432	ncbi_101799557	PLPP1	0.956120696	0.00000119	-
XR_003492471.1	LOC113839840	ncbi_101799557	PLPP1	0.995993799	8.07E-12	-
XR_003492815.1	LOC113840095	ncbi_101799557	PLPP1	0.989700853	8.97E-10	-
XR_003492840.1	LOC113840110	ncbi_101799557	PLPP1	0.965331947	0.000000372	-
XR_003492841.1	LOC113840110	ncbi_101799557	PLPP1	0.954860322	0.00000137	-
XR_003492939.1	LOC101799584	ncbi_101799557	PLPP1	0.960278112	0.000000728	-
XR_003492980.1	LOC113840212	ncbi_101799557	PLPP1	0.950343175	0.00000219	-
XR_003493063.1	LOC113840289	ncbi_101799557	PLPP1	0.959743169	0.000000778	-
XR_003495828.1	LOC106016958	ncbi_101799557	PLPP1	0.966073644	0.000000334	-

XR_003499673.1	LOC101796143	ncbi_101799557	PLPP1	0.978517261	3.48E-08	-
XR_003499823.1	LOC113844856	ncbi_101799557	PLPP1	0.967660198	0.000000264	-
XR_003500629.1	LOC113845225	ncbi_101799557	PLPP1	0.963000132	0.000000513	-
MSTRG.11051.1	-	ncbi_101801215	DGAT2	0.975608628	6.53E-08	Global and overview maps;Lipid metabolism
MSTRG.12453.1	-	ncbi_101801215	DGAT2	0.964279721	0.000000431	Global and overview maps;Lipid metabolism
MSTRG.4618.1	-	ncbi_101801215	DGAT2	0.985271152	5.33E-09	Global and overview maps;Lipid metabolism
MSTRG.7978.1	-	ncbi_101801215	DGAT2	0.961803774	0.0000006	Global and overview maps;Lipid metabolism
MSTRG.8755.1	-	ncbi_101801215	DGAT2	0.966293779	0.000000324	Global and overview maps;Lipid metabolism
XR_003492815.1	LOC113840095	ncbi_101801215	DGAT2	0.969854454	0.000000186	Global and overview maps;Lipid metabolism
XR_003492939.1	LOC101799584	ncbi_101801215	DGAT2	0.976381386	5.56E-08	Global and overview maps;Lipid metabolism
XR_003492980.1	LOC113840212	ncbi_101801215	DGAT2	0.979625304	2.67E-08	Global and overview maps;Lipid metabolism
XR_003493063.1	LOC113840289	ncbi_101801215	DGAT2	0.957645636	0.000001	Global and overview maps;Lipid metabolism
XR_003499673.1	LOC101796143	ncbi_101801215	DGAT2	0.971681352	0.000000137	Global and overview maps;Lipid metabolism
XR_003499823.1	LOC113844856	ncbi_101801215	DGAT2	0.986797897	3.09E-09	Global and overview maps;Lipid metabolism
MSTRG.14722.2	-	ncbi_101801400	Pld4	0.975900123	6.15E-08	Global and overview maps;Lipid

						metabolism;Lipid metabolism
XR_001187895.3	LOC106015692	ncbi_101801400	Pld4	0.958362744	0.000000919	Global and overview maps;Lipid metabolism;Lipid metabolism
XR_001191431.3	LOC106017766	ncbi_101801400	Pld4	0.960788017	0.000000684	Global and overview maps;Lipid metabolism;Lipid metabolism
XR_002403174.2	LOC106017743	ncbi_101801400	Pld4	0.973815121	9.28E-08	Global and overview maps;Lipid metabolism;Lipid metabolism
XR_002404562.2	LOC110353729	ncbi_101801400	Pld4	0.970377105	0.000000171	Global and overview maps;Lipid metabolism;Lipid metabolism
XR_002406329.2	LOC110354414	ncbi_101801400	Pld4	0.959972713	0.000000757	Global and overview maps;Lipid metabolism;Lipid metabolism
XR_003497435.1	LOC106015641	ncbi_101801400	Pld4	0.960395576	0.000000718	Global and overview maps;Lipid metabolism;Lipid metabolism
XR_003497832.1	LOC110353203	ncbi_101801400	Pld4	0.957090034	0.00000107	Global and overview maps;Lipid metabolism;Lipid metabolism
XR_003498489.1	LOC113844166	ncbi_101801400	Pld4	0.959685937	0.000000784	Global and overview maps;Lipid metabolism;Lipid metabolism
XR_003498613.1	LOC113844216	ncbi_101801400	Pld4	0.979351671	2.86E-08	Global and overview maps;Lipid metabolism;Lipid metabolism
XR_003498687.1	LOC106020406	ncbi_101801400	Pld4	0.988897233	1.3E-09	Global and overview maps;Lipid metabolism;Lipid metabolism
MSTRG.14722.2	-	ncbi_101801834	Acsl5	0.984334595	7.24E-09	Global and overview maps;Transport and catabolism;Endocrine system;Endocrine system;Global and overview maps;Lipid metabolism;Cell growth and death;Lipid metabolism

MSTRG.17781.1	-	ncbi_101801834	Acsl5	0.95839085	0.000000916	Global and overview maps;Transport and catabolism;Endocrine system;Endocrine system;Global and overview maps;Lipid metabolism;Cell growth and death;Lipid metabolism
MSTRG.2002.1	-	ncbi_101801834	Acsl5	0.963452972	0.000000483	Global and overview maps;Transport and catabolism;Endocrine system;Endocrine system;Global and overview maps;Lipid metabolism;Cell growth and death;Lipid metabolism
MSTRG.7242.1	-	ncbi_101801834	Acsl5	0.954697747	0.00000139	Global and overview maps;Transport and catabolism;Endocrine system;Endocrine system;Global and overview maps;Lipid metabolism;Cell growth and death;Lipid metabolism
MSTRG.963.2	-	ncbi_101801834	Acsl5	0.964410242	0.000000424	Global and overview maps;Transport and catabolism;Endocrine system;Endocrine system;Global and overview maps;Lipid metabolism;Cell growth and death;Lipid metabolism
XR_001186998.3	LOC106015168	ncbi_101801834	Acsl5	0.957987691	0.00000096	Global and overview maps;Transport and catabolism;Endocrine system;Endocrine system;Global and overview maps;Lipid metabolism;Cell growth and death;Lipid metabolism
XR_001187895.3	LOC106015692	ncbi_101801834	Acsl5	0.970157941	0.000000177	Global and overview maps;Transport and

						catabolism;Endocrine system;Endocrine system;Global and overview maps;Lipid metabolism;Cell growth and death;Lipid metabolism
XR_001191431.3	LOC106017766	ncbi_101801834	Acsl5	0.980439504	2.18E-08	Global and overview maps;Transport and catabolism;Endocrine system;Endocrine system;Global and overview maps;Lipid metabolism;Cell growth and death;Lipid metabolism
XR_002403174.2	LOC106017743	ncbi_101801834	Acsl5	0.978525104	3.47E-08	Global and overview maps;Transport and catabolism;Endocrine system;Endocrine system;Global and overview maps;Lipid metabolism;Cell growth and death;Lipid metabolism
XR_002404562.2	LOC110353729	ncbi_101801834	Acsl5	0.978849475	3.22E-08	Global and overview maps;Transport and catabolism;Endocrine system;Endocrine system;Global and overview maps;Lipid metabolism;Cell growth and death;Lipid metabolism
XR_002406329.2	LOC110354414	ncbi_101801834	Acsl5	0.988363503	1.65E-09	Global and overview maps;Transport and catabolism;Endocrine system;Endocrine system;Global and overview maps;Lipid metabolism;Cell growth and death;Lipid metabolism
XR_003493047.1	LOC106019855	ncbi_101801834	Acsl5	0.964670889	0.000000408	Global and overview maps;Transport and catabolism;Endocrine system;Endocrine

						system;Global and overview maps;Lipid metabolism;Cell growth and death;Lipid metabolism
XR_003497435.1	LOC106015641	ncbi_101801834	Acsl5	0.981408921	0.000000017	Global and overview maps;Transport and catabolism;Endocrine system;Endocrine system;Global and overview maps;Lipid metabolism;Cell growth and death;Lipid metabolism
XR_003497454.1	LOC113843759	ncbi_101801834	Acsl5	0.959954544	0.000000758	Global and overview maps;Transport and catabolism;Endocrine system;Endocrine system;Global and overview maps;Lipid metabolism;Cell growth and death;Lipid metabolism
XR_003497832.1	LOC110353203	ncbi_101801834	Acsl5	0.962078684	0.000000579	Global and overview maps;Transport and catabolism;Endocrine system;Endocrine system;Global and overview maps;Lipid metabolism;Cell growth and death;Lipid metabolism
XR_003498073.1	LOC106015391	ncbi_101801834	Acsl5	0.976037591	5.98E-08	Global and overview maps;Transport and catabolism;Endocrine system;Endocrine system;Global and overview maps;Lipid metabolism;Cell growth and death;Lipid metabolism
XR_003498489.1	LOC113844166	ncbi_101801834	Acsl5	0.97087352	0.000000157	Global and overview maps;Transport and catabolism;Endocrine system;Endocrine system;Global and overview maps;Lipid

						metabolism;Cell growth and death;Lipid metabolism
XR_003498613.1	LOC113844216	ncbi_101801834	Acsl5	0.988670365	1.44E-09	Global and overview maps;Transport and catabolism;Endocrine system;Endocrine system;Global and overview maps;Lipid metabolism;Cell growth and death;Lipid metabolism
XR_003498687.1	LOC106020406	ncbi_101801834	Acsl5	0.98802735	1.9E-09	Global and overview maps;Transport and catabolism;Endocrine system;Endocrine system;Global and overview maps;Lipid metabolism;Cell growth and death;Lipid metabolism

Description: lncRNA_id: lncRNA id number; lncRNA_symbol: lncRNA symbol number; gene_id: mRNA id number; gene_symbol: mRNA symbol number; cor: mRNA-lncRNA correlation; p-value: significance of mRNA-lncRNA correlation relationship; pathway: name of the pathway in which the mRNA is located; K_ID: number of the pathway where the mRNA is located.
