# Supplementary material for: Identification of genes and long non-coding RNAs for intramuscular and subcutaneous fat deposition in ducks by transcriptome analysis
Source: Anim Biosci. 2025 Aug 12;39(1):250268. doi: 10.5713/ab.25.0268 (PMC12754461; doi:10.5713/ab.25.0268)
Supplement: Supplementary file 10 [file ab-25-0268-Supplementary-10.pdf]

Supplement 10. A lncRNA-mRNA co-expression network of the SCP-0-vs-SCP-4 group

lncRNA_id	lncRNA_Symbol	GeneID	Gene_Symbol	cor	p_value	KEGG_B_class
MSTRG.10098.1	-	ncbi_101790585	PLA2G4A	0.96131785	6.39E-07	Global and overview maps;Signal transduction;Cell growth and death;Lipid metabolism;Circulatory system;Endocrine system;Signal transduction;Lipid metabolism;Lipid metabolism;Lipid metabolism;Lipid metabolism
MSTRG.10100.1	-	ncbi_101790585	PLA2G4A	0.958631219	8.90E-07	Global and overview maps;Signal transduction;Cell growth and death;Lipid metabolism;Circulatory system;Endocrine system;Signal transduction;Lipid metabolism;Lipid metabolism;Lipid metabolism;Lipid metabolism
MSTRG.10341.19	-	ncbi_101790585	PLA2G4A	0.960155215	7.40E-07	Global and overview maps;Signal transduction;Cell growth and death;Lipid metabolism;Circulatory system;Endocrine system;Signal transduction;Lipid metabolism;Lipid metabolism;Lipid metabolism;Lipid metabolism
MSTRG.10341.20	-	ncbi_101790585	PLA2G4A	0.972795474	1.12E-07	Global and overview maps;Signal transduction;Cell growth and death;Lipid metabolism;Circulatory system;Endocrine system;Signal transduction;Lipid metabolism;Lipid metabolism;Lipid metabolism;Lipid metabolism
MSTRG.10341.21	-	ncbi_101790585	PLA2G4A	0.963376191	4.88E-07	Global and overview maps;Signal transduction;Cell growth and death;Lipid metabolism;Circulatory system;Endocrine system;Signal transduction;Lipid metabolism;Lipid metabolism;Lipid metabolism;Lipid metabolism
MSTRG.10341.22	-	ncbi_101790585	PLA2G4A	0.976746393	5.15E-08	Global and overview maps;Signal transduction;Cell growth and death;Lipid metabolism;Circulatory system;Endocrine system;Signal transduction;Lipid metabolism;Lipid

						metabolism;Lipid metabolism;Lipid metabolism
MSTRG.12747.4	-	ncbi_101790585	PLA2G4A	0.970151806	1.77E-07	Global and overview maps;Signal transduction;Cell growth and death;Lipid metabolism;Circulatory system;Endocrine system;Signal transduction;Lipid metabolism;Lipid metabolism;Lipid metabolism;Lipid metabolism
MSTRG.12949.1	-	ncbi_101790585	PLA2G4A	0.975210755	7.07E-08	Global and overview maps;Signal transduction;Cell growth and death;Lipid metabolism;Circulatory system;Endocrine system;Signal transduction;Lipid metabolism;Lipid metabolism;Lipid metabolism;Lipid metabolism
MSTRG.13538.3	-	ncbi_101790585	PLA2G4A	0.967907052	2.54E-07	Global and overview maps;Signal transduction;Cell growth and death;Lipid metabolism;Circulatory system;Endocrine system;Signal transduction;Lipid metabolism;Lipid metabolism;Lipid metabolism;Lipid metabolism
MSTRG.13544.2	-	ncbi_101790585	PLA2G4A	0.970731799	1.61E-07	Global and overview maps;Signal transduction;Cell growth and death;Lipid metabolism;Circulatory system;Endocrine system;Signal transduction;Lipid metabolism;Lipid metabolism;Lipid metabolism;Lipid metabolism
MSTRG.13915.1	-	ncbi_101790585	PLA2G4A	0.955512087	1.27E-06	Global and overview maps;Signal transduction;Cell growth and death;Lipid metabolism;Circulatory system;Endocrine system;Signal transduction;Lipid metabolism;Lipid metabolism;Lipid metabolism;Lipid metabolism
MSTRG.15965.1	-	ncbi_101790585	PLA2G4A	0.952029896	1.85E-06	Global and overview maps;Signal transduction;Cell growth and death;Lipid metabolism;Circulatory system;Endocrine system;Signal transduction;Lipid metabolism;Lipid metabolism;Lipid metabolism;Lipid metabolism
MSTRG.16889.1	-	ncbi_101790585	PLA2G4A	0.960914784	6.73E-07	Global and overview maps;Signal transduction;Cell growth

						and death;Lipid metabolism;Circulatory system;Endocrine system;Signal transduction;Lipid metabolism;Lipid metabolism;Lipid metabolism;Lipid metabolism
MSTRG.2606.1	-	ncbi_101790585	PLA2G4A	0.972517194	1.18E-07	Global and overview maps;Signal transduction;Cell growth and death;Lipid metabolism;Circulatory system;Endocrine system;Signal transduction;Lipid metabolism;Lipid metabolism;Lipid metabolism;Lipid metabolism
MSTRG.2608.1	-	ncbi_101790585	PLA2G4A	0.962715383	5.33E-07	Global and overview maps;Signal transduction;Cell growth and death;Lipid metabolism;Circulatory system;Endocrine system;Signal transduction;Lipid metabolism;Lipid metabolism;Lipid metabolism;Lipid metabolism
MSTRG.2904.1	-	ncbi_101790585	PLA2G4A	0.970348946	1.72E-07	Global and overview maps;Signal transduction;Cell growth and death;Lipid metabolism;Circulatory system;Endocrine system;Signal transduction;Lipid metabolism;Lipid metabolism;Lipid metabolism;Lipid metabolism
MSTRG.3080.1	-	ncbi_101790585	PLA2G4A	0.973886181	9.15E-08	Global and overview maps;Signal transduction;Cell growth and death;Lipid metabolism;Circulatory system;Endocrine system;Signal transduction;Lipid metabolism;Lipid metabolism;Lipid metabolism;Lipid metabolism
MSTRG.3622.7	-	ncbi_101790585	PLA2G4A	0.978924275	3.16E-08	Global and overview maps;Signal transduction;Cell growth and death;Lipid metabolism;Circulatory system;Endocrine system;Signal transduction;Lipid metabolism;Lipid metabolism;Lipid metabolism;Lipid metabolism
MSTRG.3639.1	-	ncbi_101790585	PLA2G4A	0.952866637	1.69E-06	Global and overview maps;Signal transduction;Cell growth and death;Lipid metabolism;Circulatory system;Endocrine system;Signal transduction;Lipid metabolism;Lipid

						metabolism;Lipid metabolism;Lipid metabolism
MSTRG.4704.1	-	ncbi_101790585	PLA2G4A	0.967441208	2.73E-07	Global and overview maps;Signal transduction;Cell growth and death;Lipid metabolism;Circulatory system;Endocrine system;Signal transduction;Lipid metabolism;Lipid metabolism;Lipid metabolism;Lipid metabolism
MSTRG.5011.1	-	ncbi_101790585	PLA2G4A	0.975762286	6.33E-08	Global and overview maps;Signal transduction;Cell growth and death;Lipid metabolism;Circulatory system;Endocrine system;Signal transduction;Lipid metabolism;Lipid metabolism;Lipid metabolism;Lipid metabolism
MSTRG.5135.1	-	ncbi_101790585	PLA2G4A	0.966052773	3.35E-07	Global and overview maps;Signal transduction;Cell growth and death;Lipid metabolism;Circulatory system;Endocrine system;Signal transduction;Lipid metabolism;Lipid metabolism;Lipid metabolism;Lipid metabolism
MSTRG.5135.3	-	ncbi_101790585	PLA2G4A	0.974087108	8.81E-08	Global and overview maps;Signal transduction;Cell growth and death;Lipid metabolism;Circulatory system;Endocrine system;Signal transduction;Lipid metabolism;Lipid metabolism;Lipid metabolism;Lipid metabolism
MSTRG.582.1	-	ncbi_101790585	PLA2G4A	0.966164591	3.30E-07	Global and overview maps;Signal transduction;Cell growth and death;Lipid metabolism;Circulatory system;Endocrine system;Signal transduction;Lipid metabolism;Lipid metabolism;Lipid metabolism;Lipid metabolism
MSTRG.582.3	-	ncbi_101790585	PLA2G4A	0.967080043	2.88E-07	Global and overview maps;Signal transduction;Cell growth and death;Lipid metabolism;Circulatory system;Endocrine system;Signal transduction;Lipid metabolism;Lipid metabolism;Lipid metabolism;Lipid metabolism
MSTRG.582.4	-	ncbi_101790585	PLA2G4A	0.953292366	1.62E-06	Global and overview maps;Signal transduction;Cell growth

						and death;Lipid metabolism;Circulatory system;Endocrine system;Signal transduction;Lipid metabolism;Lipid metabolism;Lipid metabolism;Lipid metabolism
MSTRG.5846.1	-	ncbi_101790585	PLA2G4A	0.966148524	3.31E-07	Global and overview maps;Signal transduction;Cell growth and death;Lipid metabolism;Circulatory system;Endocrine system;Signal transduction;Lipid metabolism;Lipid metabolism;Lipid metabolism;Lipid metabolism
MSTRG.5881.2	-	ncbi_101790585	PLA2G4A	0.961756152	6.04E-07	Global and overview maps;Signal transduction;Cell growth and death;Lipid metabolism;Circulatory system;Endocrine system;Signal transduction;Lipid metabolism;Lipid metabolism;Lipid metabolism;Lipid metabolism
MSTRG.5997.1	-	ncbi_101790585	PLA2G4A	0.970792332	1.59E-07	Global and overview maps;Signal transduction;Cell growth and death;Lipid metabolism;Circulatory system;Endocrine system;Signal transduction;Lipid metabolism;Lipid metabolism;Lipid metabolism;Lipid metabolism
MSTRG.6017.1	-	ncbi_101790585	PLA2G4A	0.967181796	2.84E-07	Global and overview maps;Signal transduction;Cell growth and death;Lipid metabolism;Circulatory system;Endocrine system;Signal transduction;Lipid metabolism;Lipid metabolism;Lipid metabolism;Lipid metabolism
MSTRG.6127.1	-	ncbi_101790585	PLA2G4A	0.969895528	1.85E-07	Global and overview maps;Signal transduction;Cell growth and death;Lipid metabolism;Circulatory system;Endocrine system;Signal transduction;Lipid metabolism;Lipid metabolism;Lipid metabolism;Lipid metabolism
MSTRG.6316.1	-	ncbi_101790585	PLA2G4A	-0.967894114	2.55E-07	Global and overview maps;Signal transduction;Cell growth and death;Lipid metabolism;Circulatory system;Endocrine system;Signal transduction;Lipid metabolism;Lipid

						metabolism;Lipid metabolism;Lipid metabolism
MSTRG.6704.2	-	ncbi_101790585	PLA2G4A	0.971116683	1.51E-07	Global and overview maps;Signal transduction;Cell growth and death;Lipid metabolism;Circulatory system;Endocrine system;Signal transduction;Lipid metabolism;Lipid metabolism;Lipid metabolism;Lipid metabolism
MSTRG.6704.3	-	ncbi_101790585	PLA2G4A	0.971768038	1.35E-07	Global and overview maps;Signal transduction;Cell growth and death;Lipid metabolism;Circulatory system;Endocrine system;Signal transduction;Lipid metabolism;Lipid metabolism;Lipid metabolism;Lipid metabolism
MSTRG.6704.4	-	ncbi_101790585	PLA2G4A	0.972353368	1.21E-07	Global and overview maps;Signal transduction;Cell growth and death;Lipid metabolism;Circulatory system;Endocrine system;Signal transduction;Lipid metabolism;Lipid metabolism;Lipid metabolism;Lipid metabolism
MSTRG.715.1	-	ncbi_101790585	PLA2G4A	0.963088453	5.07E-07	Global and overview maps;Signal transduction;Cell growth and death;Lipid metabolism;Circulatory system;Endocrine system;Signal transduction;Lipid metabolism;Lipid metabolism;Lipid metabolism;Lipid metabolism
MSTRG.7239.1	-	ncbi_101790585	PLA2G4A	0.971444602	1.43E-07	Global and overview maps;Signal transduction;Cell growth and death;Lipid metabolism;Circulatory system;Endocrine system;Signal transduction;Lipid metabolism;Lipid metabolism;Lipid metabolism;Lipid metabolism
MSTRG.7248.1	-	ncbi_101790585	PLA2G4A	0.985511665	4.91E-09	Global and overview maps;Signal transduction;Cell growth and death;Lipid metabolism;Circulatory system;Endocrine system;Signal transduction;Lipid metabolism;Lipid metabolism;Lipid metabolism;Lipid metabolism
MSTRG.756.3	-	ncbi_101790585	PLA2G4A	0.970200098	1.76E-07	Global and overview maps;Signal transduction;Cell growth

						and death;Lipid metabolism;Circulatory system;Endocrine system;Signal transduction;Lipid metabolism;Lipid metabolism;Lipid metabolism;Lipid metabolism
MSTRG.7621.5	-	ncbi_101790585	PLA2G4A	0.968430324	2.34E-07	Global and overview maps;Signal transduction;Cell growth and death;Lipid metabolism;Circulatory system;Endocrine system;Signal transduction;Lipid metabolism;Lipid metabolism;Lipid metabolism;Lipid metabolism
MSTRG.7949.1	-	ncbi_101790585	PLA2G4A	0.962571864	5.43E-07	Global and overview maps;Signal transduction;Cell growth and death;Lipid metabolism;Circulatory system;Endocrine system;Signal transduction;Lipid metabolism;Lipid metabolism;Lipid metabolism;Lipid metabolism
MSTRG.7954.1	-	ncbi_101790585	PLA2G4A	0.97007032	1.80E-07	Global and overview maps;Signal transduction;Cell growth and death;Lipid metabolism;Circulatory system;Endocrine system;Signal transduction;Lipid metabolism;Lipid metabolism;Lipid metabolism;Lipid metabolism
MSTRG.852.1	-	ncbi_101790585	PLA2G4A	0.956072216	1.20E-06	Global and overview maps;Signal transduction;Cell growth and death;Lipid metabolism;Circulatory system;Endocrine system;Signal transduction;Lipid metabolism;Lipid metabolism;Lipid metabolism;Lipid metabolism
MSTRG.885.1	-	ncbi_101790585	PLA2G4A	0.960043318	7.50E-07	Global and overview maps;Signal transduction;Cell growth and death;Lipid metabolism;Circulatory system;Endocrine system;Signal transduction;Lipid metabolism;Lipid metabolism;Lipid metabolism;Lipid metabolism
MSTRG.9062.2	-	ncbi_101790585	PLA2G4A	0.967777724	2.59E-07	Global and overview maps;Signal transduction;Cell growth and death;Lipid metabolism;Circulatory system;Endocrine system;Signal transduction;Lipid metabolism;Lipid

						metabolism;Lipid metabolism;Lipid metabolism
MSTRG.9062.4	-	ncbi_101790585	PLA2G4A	0.964452056	4.21E-07	Global and overview maps;Signal transduction;Cell growth and death;Lipid metabolism;Circulatory system;Endocrine system;Signal transduction;Lipid metabolism;Lipid metabolism;Lipid metabolism;Lipid metabolism
XR_001185755.3	LOC106014431	ncbi_101790585	PLA2G4A	0.950789654	2.09E-06	Global and overview maps;Signal transduction;Cell growth and death;Lipid metabolism;Circulatory system;Endocrine system;Signal transduction;Lipid metabolism;Lipid metabolism;Lipid metabolism;Lipid metabolism
XR_001187232.3	LOC106015301	ncbi_101790585	PLA2G4A	0.957959849	9.64E-07	Global and overview maps;Signal transduction;Cell growth and death;Lipid metabolism;Circulatory system;Endocrine system;Signal transduction;Lipid metabolism;Lipid metabolism;Lipid metabolism;Lipid metabolism
XR_001187494.3	LOC106015449	ncbi_101790585	PLA2G4A	0.958788581	8.74E-07	Global and overview maps;Signal transduction;Cell growth and death;Lipid metabolism;Circulatory system;Endocrine system;Signal transduction;Lipid metabolism;Lipid metabolism;Lipid metabolism;Lipid metabolism
XR_001187862.3	LOC106015672	ncbi_101790585	PLA2G4A	0.980955086	1.91E-08	Global and overview maps;Signal transduction;Cell growth and death;Lipid metabolism;Circulatory system;Endocrine system;Signal transduction;Lipid metabolism;Lipid metabolism;Lipid metabolism;Lipid metabolism
XR_001187865.3	LOC106015672	ncbi_101790585	PLA2G4A	0.981485977	1.66E-08	Global and overview maps;Signal transduction;Cell growth and death;Lipid metabolism;Circulatory system;Endocrine system;Signal transduction;Lipid metabolism;Lipid metabolism;Lipid metabolism;Lipid metabolism
XR_001187947.2	LOC106015729	ncbi_101790585	PLA2G4A	0.976543259	5.38E-08	Global and overview maps;Signal transduction;Cell growth

						and death;Lipid metabolism;Circulatory system;Endocrine system;Signal transduction;Lipid metabolism;Lipid metabolism;Lipid metabolism;Lipid metabolism
XR_001188444.3	LOC106016033	ncbi_101790585	PLA2G4A	0.971966755	1.30E-07	Global and overview maps;Signal transduction;Cell growth and death;Lipid metabolism;Circulatory system;Endocrine system;Signal transduction;Lipid metabolism;Lipid metabolism;Lipid metabolism;Lipid metabolism
XR_001189247.3	LOC106016487	ncbi_101790585	PLA2G4A	0.953329248	1.61E-06	Global and overview maps;Signal transduction;Cell growth and death;Lipid metabolism;Circulatory system;Endocrine system;Signal transduction;Lipid metabolism;Lipid metabolism;Lipid metabolism;Lipid metabolism
XR_001189809.3	LOC106016826	ncbi_101790585	PLA2G4A	0.95999352	7.55E-07	Global and overview maps;Signal transduction;Cell growth and death;Lipid metabolism;Circulatory system;Endocrine system;Signal transduction;Lipid metabolism;Lipid metabolism;Lipid metabolism;Lipid metabolism
XR_001189917.2	LOC106016889	ncbi_101790585	PLA2G4A	0.977075566	4.80E-08	Global and overview maps;Signal transduction;Cell growth and death;Lipid metabolism;Circulatory system;Endocrine system;Signal transduction;Lipid metabolism;Lipid metabolism;Lipid metabolism;Lipid metabolism
XR_001189941.3	LOC106016901	ncbi_101790585	PLA2G4A	0.951483087	1.95E-06	Global and overview maps;Signal transduction;Cell growth and death;Lipid metabolism;Circulatory system;Endocrine system;Signal transduction;Lipid metabolism;Lipid metabolism;Lipid metabolism;Lipid metabolism
XR_001190278.3	LOC106017074	ncbi_101790585	PLA2G4A	0.960936686	6.71E-07	Global and overview maps;Signal transduction;Cell growth and death;Lipid metabolism;Circulatory system;Endocrine system;Signal transduction;Lipid metabolism;Lipid

						metabolism;Lipid metabolism;Lipid metabolism
XR_001190631.3	LOC106017268	ncbi_101790585	PLA2G4A	0.967287762	2.79E-07	Global and overview maps;Signal transduction;Cell growth and death;Lipid metabolism;Circulatory system;Endocrine system;Signal transduction;Lipid metabolism;Lipid metabolism;Lipid metabolism;Lipid metabolism
XR_001190677.3	LOC106017297	ncbi_101790585	PLA2G4A	0.978030958	3.88E-08	Global and overview maps;Signal transduction;Cell growth and death;Lipid metabolism;Circulatory system;Endocrine system;Signal transduction;Lipid metabolism;Lipid metabolism;Lipid metabolism;Lipid metabolism
XR_001191423.3	LOC106017763	ncbi_101790585	PLA2G4A	0.960917171	6.72E-07	Global and overview maps;Signal transduction;Cell growth and death;Lipid metabolism;Circulatory system;Endocrine system;Signal transduction;Lipid metabolism;Lipid metabolism;Lipid metabolism;Lipid metabolism
XR_001191855.3	LOC106018027	ncbi_101790585	PLA2G4A	0.970139995	1.78E-07	Global and overview maps;Signal transduction;Cell growth and death;Lipid metabolism;Circulatory system;Endocrine system;Signal transduction;Lipid metabolism;Lipid metabolism;Lipid metabolism;Lipid metabolism
XR_001193197.3	LOC106018814	ncbi_101790585	PLA2G4A	0.95460588	1.41E-06	Global and overview maps;Signal transduction;Cell growth and death;Lipid metabolism;Circulatory system;Endocrine system;Signal transduction;Lipid metabolism;Lipid metabolism;Lipid metabolism;Lipid metabolism
XR_001193335.3	LOC106018911	ncbi_101790585	PLA2G4A	0.978483516	3.50E-08	Global and overview maps;Signal transduction;Cell growth and death;Lipid metabolism;Circulatory system;Endocrine system;Signal transduction;Lipid metabolism;Lipid metabolism;Lipid metabolism;Lipid metabolism
XR_001193468.3	LOC106018998	ncbi_101790585	PLA2G4A	0.96958004	1.95E-07	Global and overview maps;Signal transduction;Cell growth

						and death;Lipid metabolism;Circulatory system;Endocrine system;Signal transduction;Lipid metabolism;Lipid metabolism;Lipid metabolism;Lipid metabolism
XR_001193619.3	LOC106019066	ncbi_101790585	PLA2G4A	0.979046221	3.07E-08	Global and overview maps;Signal transduction;Cell growth and death;Lipid metabolism;Circulatory system;Endocrine system;Signal transduction;Lipid metabolism;Lipid metabolism;Lipid metabolism;Lipid metabolism
XR_001194132.2	LOC106019368	ncbi_101790585	PLA2G4A	0.980183023	2.33E-08	Global and overview maps;Signal transduction;Cell growth and death;Lipid metabolism;Circulatory system;Endocrine system;Signal transduction;Lipid metabolism;Lipid metabolism;Lipid metabolism;Lipid metabolism
XR_001194441.2	LOC106019549	ncbi_101790585	PLA2G4A	0.982980962	1.09E-08	Global and overview maps;Signal transduction;Cell growth and death;Lipid metabolism;Circulatory system;Endocrine system;Signal transduction;Lipid metabolism;Lipid metabolism;Lipid metabolism;Lipid metabolism
XR_002398728.2	LOC106014648	ncbi_101790585	PLA2G4A	0.960575659	7.02E-07	Global and overview maps;Signal transduction;Cell growth and death;Lipid metabolism;Circulatory system;Endocrine system;Signal transduction;Lipid metabolism;Lipid metabolism;Lipid metabolism;Lipid metabolism
XR_002398851.2	LOC106014736	ncbi_101790585	PLA2G4A	0.957516384	1.01E-06	Global and overview maps;Signal transduction;Cell growth and death;Lipid metabolism;Circulatory system;Endocrine system;Signal transduction;Lipid metabolism;Lipid metabolism;Lipid metabolism;Lipid metabolism
XR_002399173.2	LOC110351568	ncbi_101790585	PLA2G4A	0.980510591	2.14E-08	Global and overview maps;Signal transduction;Cell growth and death;Lipid metabolism;Circulatory system;Endocrine system;Signal transduction;Lipid metabolism;Lipid

						metabolism;Lipid metabolism;Lipid metabolism
XR_002399314.2	LOC106015131	ncbi_101790585	PLA2G4A	0.963261978	4.96E-07	Global and overview maps;Signal transduction;Cell growth and death;Lipid metabolism;Circulatory system;Endocrine system;Signal transduction;Lipid metabolism;Lipid metabolism;Lipid metabolism;Lipid metabolism
XR_002399447.2	LOC110351699	ncbi_101790585	PLA2G4A	0.95201594	1.85E-06	Global and overview maps;Signal transduction;Cell growth and death;Lipid metabolism;Circulatory system;Endocrine system;Signal transduction;Lipid metabolism;Lipid metabolism;Lipid metabolism;Lipid metabolism
XR_002399732.1	LOC110351814	ncbi_101790585	PLA2G4A	0.976131688	5.86E-08	Global and overview maps;Signal transduction;Cell growth and death;Lipid metabolism;Circulatory system;Endocrine system;Signal transduction;Lipid metabolism;Lipid metabolism;Lipid metabolism;Lipid metabolism
XR_002399836.2	LOC106015519	ncbi_101790585	PLA2G4A	0.964193162	4.37E-07	Global and overview maps;Signal transduction;Cell growth and death;Lipid metabolism;Circulatory system;Endocrine system;Signal transduction;Lipid metabolism;Lipid metabolism;Lipid metabolism;Lipid metabolism
XR_002399884.2	LOC110351873	ncbi_101790585	PLA2G4A	0.979295812	2.89E-08	Global and overview maps;Signal transduction;Cell growth and death;Lipid metabolism;Circulatory system;Endocrine system;Signal transduction;Lipid metabolism;Lipid metabolism;Lipid metabolism;Lipid metabolism
XR_002400226.2	LOC106015733	ncbi_101790585	PLA2G4A	0.964662652	4.09E-07	Global and overview maps;Signal transduction;Cell growth and death;Lipid metabolism;Circulatory system;Endocrine system;Signal transduction;Lipid metabolism;Lipid metabolism;Lipid metabolism;Lipid metabolism
XR_002400249.2	LOC110351990	ncbi_101790585	PLA2G4A	0.969110533	2.10E-07	Global and overview maps;Signal transduction;Cell growth

						and death;Lipid metabolism;Circulatory system;Endocrine system;Signal transduction;Lipid metabolism;Lipid metabolism;Lipid metabolism;Lipid metabolism
XR_002400594.2	LOC110352134	ncbi_101790585	PLA2G4A	0.96041032	7.17E-07	Global and overview maps;Signal transduction;Cell growth and death;Lipid metabolism;Circulatory system;Endocrine system;Signal transduction;Lipid metabolism;Lipid metabolism;Lipid metabolism;Lipid metabolism
XR_002400646.2	LOC110352151	ncbi_101790585	PLA2G4A	0.95869205	8.84E-07	Global and overview maps;Signal transduction;Cell growth and death;Lipid metabolism;Circulatory system;Endocrine system;Signal transduction;Lipid metabolism;Lipid metabolism;Lipid metabolism;Lipid metabolism
XR_002400941.2	LOC106016276	ncbi_101790585	PLA2G4A	0.951723154	1.90E-06	Global and overview maps;Signal transduction;Cell growth and death;Lipid metabolism;Circulatory system;Endocrine system;Signal transduction;Lipid metabolism;Lipid metabolism;Lipid metabolism;Lipid metabolism
XR_002401026.2	LOC110352298	ncbi_101790585	PLA2G4A	0.956232464	1.18E-06	Global and overview maps;Signal transduction;Cell growth and death;Lipid metabolism;Circulatory system;Endocrine system;Signal transduction;Lipid metabolism;Lipid metabolism;Lipid metabolism;Lipid metabolism
XR_002401202.2	LOC110352351	ncbi_101790585	PLA2G4A	0.968744748	2.23E-07	Global and overview maps;Signal transduction;Cell growth and death;Lipid metabolism;Circulatory system;Endocrine system;Signal transduction;Lipid metabolism;Lipid metabolism;Lipid metabolism;Lipid metabolism
XR_002401479.2	LOC106016692	ncbi_101790585	PLA2G4A	0.956260869	1.17E-06	Global and overview maps;Signal transduction;Cell growth and death;Lipid metabolism;Circulatory system;Endocrine system;Signal transduction;Lipid metabolism;Lipid

						metabolism;Lipid metabolism;Lipid metabolism
XR_002401866.2	LOC106016894	ncbi_101790585	PLA2G4A	0.97260902	1.16E-07	Global and overview maps;Signal transduction;Cell growth and death;Lipid metabolism;Circulatory system;Endocrine system;Signal transduction;Lipid metabolism;Lipid metabolism;Lipid metabolism;Lipid metabolism
XR_002401875.2	LOC110352627	ncbi_101790585	PLA2G4A	0.98069219	2.05E-08	Global and overview maps;Signal transduction;Cell growth and death;Lipid metabolism;Circulatory system;Endocrine system;Signal transduction;Lipid metabolism;Lipid metabolism;Lipid metabolism;Lipid metabolism
XR_002402176.2	LOC110352712	ncbi_101790585	PLA2G4A	0.954815532	1.37E-06	Global and overview maps;Signal transduction;Cell growth and death;Lipid metabolism;Circulatory system;Endocrine system;Signal transduction;Lipid metabolism;Lipid metabolism;Lipid metabolism;Lipid metabolism
XR_002402646.2	LOC101794014	ncbi_101790585	PLA2G4A	0.961186751	6.50E-07	Global and overview maps;Signal transduction;Cell growth and death;Lipid metabolism;Circulatory system;Endocrine system;Signal transduction;Lipid metabolism;Lipid metabolism;Lipid metabolism;Lipid metabolism
XR_002402718.2	LOC106017475	ncbi_101790585	PLA2G4A	0.964035713	4.46E-07	Global and overview maps;Signal transduction;Cell growth and death;Lipid metabolism;Circulatory system;Endocrine system;Signal transduction;Lipid metabolism;Lipid metabolism;Lipid metabolism;Lipid metabolism
XR_002402955.2	LOC106017628	ncbi_101790585	PLA2G4A	0.959130564	8.38E-07	Global and overview maps;Signal transduction;Cell growth and death;Lipid metabolism;Circulatory system;Endocrine system;Signal transduction;Lipid metabolism;Lipid metabolism;Lipid metabolism;Lipid metabolism
XR_002403184.2	LOC106017746	ncbi_101790585	PLA2G4A	0.969739872	1.90E-07	Global and overview maps;Signal transduction;Cell growth

						and death;Lipid metabolism;Circulatory system;Endocrine system;Signal transduction;Lipid metabolism;Lipid metabolism;Lipid metabolism;Lipid metabolism
XR_002403213.2	LOC106017699	ncbi_101790585	PLA2G4A	0.960273273	7.29E-07	Global and overview maps;Signal transduction;Cell growth and death;Lipid metabolism;Circulatory system;Endocrine system;Signal transduction;Lipid metabolism;Lipid metabolism;Lipid metabolism;Lipid metabolism
XR_002404244.2	LOC106018581	ncbi_101790585	PLA2G4A	0.953532744	1.58E-06	Global and overview maps;Signal transduction;Cell growth and death;Lipid metabolism;Circulatory system;Endocrine system;Signal transduction;Lipid metabolism;Lipid metabolism;Lipid metabolism;Lipid metabolism
XR_002404947.2	LOC110353846	ncbi_101790585	PLA2G4A	0.978871284	3.20E-08	Global and overview maps;Signal transduction;Cell growth and death;Lipid metabolism;Circulatory system;Endocrine system;Signal transduction;Lipid metabolism;Lipid metabolism;Lipid metabolism;Lipid metabolism
XR_002405449.2	LOC110354050	ncbi_101790585	PLA2G4A	0.961096964	6.57E-07	Global and overview maps;Signal transduction;Cell growth and death;Lipid metabolism;Circulatory system;Endocrine system;Signal transduction;Lipid metabolism;Lipid metabolism;Lipid metabolism;Lipid metabolism
XR_002405947.2	LOC106019753	ncbi_101790585	PLA2G4A	0.966795222	3.01E-07	Global and overview maps;Signal transduction;Cell growth and death;Lipid metabolism;Circulatory system;Endocrine system;Signal transduction;Lipid metabolism;Lipid metabolism;Lipid metabolism;Lipid metabolism
XR_002406465.2	LOC106020114	ncbi_101790585	PLA2G4A	0.983666058	8.91E-09	Global and overview maps;Signal transduction;Cell growth and death;Lipid metabolism;Circulatory system;Endocrine system;Signal transduction;Lipid metabolism;Lipid

						metabolism;Lipid metabolism;Lipid metabolism
XR_002406489.2	LOC106020129	ncbi_101790585	PLA2G4A	0.960159753	7.39E-07	Global and overview maps;Signal transduction;Cell growth and death;Lipid metabolism;Circulatory system;Endocrine system;Signal transduction;Lipid metabolism;Lipid metabolism;Lipid metabolism;Lipid metabolism
XR_002406729.2	LOC106020349	ncbi_101790585	PLA2G4A	0.96294594	5.17E-07	Global and overview maps;Signal transduction;Cell growth and death;Lipid metabolism;Circulatory system;Endocrine system;Signal transduction;Lipid metabolism;Lipid metabolism;Lipid metabolism;Lipid metabolism
XR_002406750.2	LOC106020393	ncbi_101790585	PLA2G4A	0.966756141	3.02E-07	Global and overview maps;Signal transduction;Cell growth and death;Lipid metabolism;Circulatory system;Endocrine system;Signal transduction;Lipid metabolism;Lipid metabolism;Lipid metabolism;Lipid metabolism
XR_003492230.1	LOC113839672	ncbi_101790585	PLA2G4A	0.963854297	4.57E-07	Global and overview maps;Signal transduction;Cell growth and death;Lipid metabolism;Circulatory system;Endocrine system;Signal transduction;Lipid metabolism;Lipid metabolism;Lipid metabolism;Lipid metabolism
XR_003493103.1	LOC113840309	ncbi_101790585	PLA2G4A	0.981926376	1.47E-08	Global and overview maps;Signal transduction;Cell growth and death;Lipid metabolism;Circulatory system;Endocrine system;Signal transduction;Lipid metabolism;Lipid metabolism;Lipid metabolism;Lipid metabolism
XR_003493806.1	LOC113841174	ncbi_101790585	PLA2G4A	0.985256586	5.35E-09	Global and overview maps;Signal transduction;Cell growth and death;Lipid metabolism;Circulatory system;Endocrine system;Signal transduction;Lipid metabolism;Lipid metabolism;Lipid metabolism;Lipid metabolism
XR_003494775.1	LOC110352361	ncbi_101790585	PLA2G4A	0.950789654	2.09E-06	Global and overview maps;Signal transduction;Cell growth

						and death;Lipid metabolism;Circulatory system;Endocrine system;Signal transduction;Lipid metabolism;Lipid metabolism;Lipid metabolism;Lipid metabolism
XR_003494883.1	LOC106020483	ncbi_101790585	PLA2G4A	0.985060563	5.72E-09	Global and overview maps;Signal transduction;Cell growth and death;Lipid metabolism;Circulatory system;Endocrine system;Signal transduction;Lipid metabolism;Lipid metabolism;Lipid metabolism;Lipid metabolism
XR_003494888.1	LOC113842396	ncbi_101790585	PLA2G4A	0.984058037	7.90E-09	Global and overview maps;Signal transduction;Cell growth and death;Lipid metabolism;Circulatory system;Endocrine system;Signal transduction;Lipid metabolism;Lipid metabolism;Lipid metabolism;Lipid metabolism
XR_003494957.1	LOC101799909	ncbi_101790585	PLA2G4A	0.986400936	3.58E-09	Global and overview maps;Signal transduction;Cell growth and death;Lipid metabolism;Circulatory system;Endocrine system;Signal transduction;Lipid metabolism;Lipid metabolism;Lipid metabolism;Lipid metabolism
XR_003494961.1	LOC113842447	ncbi_101790585	PLA2G4A	0.964313726	4.29E-07	Global and overview maps;Signal transduction;Cell growth and death;Lipid metabolism;Circulatory system;Endocrine system;Signal transduction;Lipid metabolism;Lipid metabolism;Lipid metabolism;Lipid metabolism
XR_003495345.1	LOC101793386	ncbi_101790585	PLA2G4A	0.964310648	4.29E-07	Global and overview maps;Signal transduction;Cell growth and death;Lipid metabolism;Circulatory system;Endocrine system;Signal transduction;Lipid metabolism;Lipid metabolism;Lipid metabolism;Lipid metabolism
XR_003495483.1	LOC113842770	ncbi_101790585	PLA2G4A	0.988355821	1.65E-09	Global and overview maps;Signal transduction;Cell growth and death;Lipid metabolism;Circulatory system;Endocrine system;Signal transduction;Lipid metabolism;Lipid

						metabolism;Lipid metabolism;Lipid metabolism
XR_003496139.1	LOC113843116	ncbi_101790585	PLA2G4A	0.960610905	6.99E-07	Global and overview maps;Signal transduction;Cell growth and death;Lipid metabolism;Circulatory system;Endocrine system;Signal transduction;Lipid metabolism;Lipid metabolism;Lipid metabolism;Lipid metabolism
XR_003496334.1	LOC113843176	ncbi_101790585	PLA2G4A	0.970580808	1.65E-07	Global and overview maps;Signal transduction;Cell growth and death;Lipid metabolism;Circulatory system;Endocrine system;Signal transduction;Lipid metabolism;Lipid metabolism;Lipid metabolism;Lipid metabolism
XR_003496370.1	LOC106017302	ncbi_101790585	PLA2G4A	0.974199824	8.62E-08	Global and overview maps;Signal transduction;Cell growth and death;Lipid metabolism;Circulatory system;Endocrine system;Signal transduction;Lipid metabolism;Lipid metabolism;Lipid metabolism;Lipid metabolism
XR_003496440.1	LOC106015283	ncbi_101790585	PLA2G4A	0.980664794	2.06E-08	Global and overview maps;Signal transduction;Cell growth and death;Lipid metabolism;Circulatory system;Endocrine system;Signal transduction;Lipid metabolism;Lipid metabolism;Lipid metabolism;Lipid metabolism
XR_003496447.1	LOC113843228	ncbi_101790585	PLA2G4A	0.968516588	2.31E-07	Global and overview maps;Signal transduction;Cell growth and death;Lipid metabolism;Circulatory system;Endocrine system;Signal transduction;Lipid metabolism;Lipid metabolism;Lipid metabolism;Lipid metabolism
XR_003496550.1	LOC106018878	ncbi_101790585	PLA2G4A	0.95240164	1.78E-06	Global and overview maps;Signal transduction;Cell growth and death;Lipid metabolism;Circulatory system;Endocrine system;Signal transduction;Lipid metabolism;Lipid metabolism;Lipid metabolism;Lipid metabolism
XR_003496598.1	LOC113843320	ncbi_101790585	PLA2G4A	0.967687154	2.63E-07	Global and overview maps;Signal transduction;Cell growth

						and death;Lipid metabolism;Circulatory system;Endocrine system;Signal transduction;Lipid metabolism;Lipid metabolism;Lipid metabolism;Lipid metabolism
XR_003496601.1	LOC101791220	ncbi_101790585	PLA2G4A	0.955609362	1.26E-06	Global and overview maps;Signal transduction;Cell growth and death;Lipid metabolism;Circulatory system;Endocrine system;Signal transduction;Lipid metabolism;Lipid metabolism;Lipid metabolism;Lipid metabolism
XR_003496756.1	LOC113843454	ncbi_101790585	PLA2G4A	0.950591112	2.13E-06	Global and overview maps;Signal transduction;Cell growth and death;Lipid metabolism;Circulatory system;Endocrine system;Signal transduction;Lipid metabolism;Lipid metabolism;Lipid metabolism;Lipid metabolism
XR_003497001.1	LOC106017547	ncbi_101790585	PLA2G4A	0.970447951	1.69E-07	Global and overview maps;Signal transduction;Cell growth and death;Lipid metabolism;Circulatory system;Endocrine system;Signal transduction;Lipid metabolism;Lipid metabolism;Lipid metabolism;Lipid metabolism
XR_003497002.1	LOC106017547	ncbi_101790585	PLA2G4A	0.956859782	1.09E-06	Global and overview maps;Signal transduction;Cell growth and death;Lipid metabolism;Circulatory system;Endocrine system;Signal transduction;Lipid metabolism;Lipid metabolism;Lipid metabolism;Lipid metabolism
XR_003497296.1	LOC113843645	ncbi_101790585	PLA2G4A	0.97508924	7.25E-08	Global and overview maps;Signal transduction;Cell growth and death;Lipid metabolism;Circulatory system;Endocrine system;Signal transduction;Lipid metabolism;Lipid metabolism;Lipid metabolism;Lipid metabolism
XR_003497481.1	LOC110351804	ncbi_101790585	PLA2G4A	0.952742527	1.71E-06	Global and overview maps;Signal transduction;Cell growth and death;Lipid metabolism;Circulatory system;Endocrine system;Signal transduction;Lipid metabolism;Lipid

						metabolism;Lipid metabolism;Lipid metabolism
XR_003497511.1	LOC110353153	ncbi_101790585	PLA2G4A	0.959689131	7.83E-07	Global and overview maps;Signal transduction;Cell growth and death;Lipid metabolism;Circulatory system;Endocrine system;Signal transduction;Lipid metabolism;Lipid metabolism;Lipid metabolism;Lipid metabolism
XR_003497529.1	LOC113843776	ncbi_101790585	PLA2G4A	0.963447922	4.83E-07	Global and overview maps;Signal transduction;Cell growth and death;Lipid metabolism;Circulatory system;Endocrine system;Signal transduction;Lipid metabolism;Lipid metabolism;Lipid metabolism;Lipid metabolism
XR_003497564.1	LOC101802970	ncbi_101790585	PLA2G4A	0.971345722	1.45E-07	Global and overview maps;Signal transduction;Cell growth and death;Lipid metabolism;Circulatory system;Endocrine system;Signal transduction;Lipid metabolism;Lipid metabolism;Lipid metabolism;Lipid metabolism
XR_003497566.1	LOC101802970	ncbi_101790585	PLA2G4A	0.968384144	2.36E-07	Global and overview maps;Signal transduction;Cell growth and death;Lipid metabolism;Circulatory system;Endocrine system;Signal transduction;Lipid metabolism;Lipid metabolism;Lipid metabolism;Lipid metabolism
XR_003497661.1	LOC101798800	ncbi_101790585	PLA2G4A	0.979147633	3.00E-08	Global and overview maps;Signal transduction;Cell growth and death;Lipid metabolism;Circulatory system;Endocrine system;Signal transduction;Lipid metabolism;Lipid metabolism;Lipid metabolism;Lipid metabolism
XR_003497665.1	LOC110351913	ncbi_101790585	PLA2G4A	0.970388956	1.71E-07	Global and overview maps;Signal transduction;Cell growth and death;Lipid metabolism;Circulatory system;Endocrine system;Signal transduction;Lipid metabolism;Lipid metabolism;Lipid metabolism;Lipid metabolism
XR_003497899.1	LOC110353379	ncbi_101790585	PLA2G4A	0.971084467	1.52E-07	Global and overview maps;Signal transduction;Cell growth

						and death;Lipid metabolism;Circulatory system;Endocrine system;Signal transduction;Lipid metabolism;Lipid metabolism;Lipid metabolism;Lipid metabolism
XR_003497951.1	LOC113843960	ncbi_101790585	PLA2G4A	0.956932858	1.09E-06	Global and overview maps;Signal transduction;Cell growth and death;Lipid metabolism;Circulatory system;Endocrine system;Signal transduction;Lipid metabolism;Lipid metabolism;Lipid metabolism;Lipid metabolism
XR_003498151.1	LOC110353008	ncbi_101790585	PLA2G4A	0.9753571	6.87E-08	Global and overview maps;Signal transduction;Cell growth and death;Lipid metabolism;Circulatory system;Endocrine system;Signal transduction;Lipid metabolism;Lipid metabolism;Lipid metabolism;Lipid metabolism
XR_003498491.1	LOC113844169	ncbi_101790585	PLA2G4A	0.955666473	1.25E-06	Global and overview maps;Signal transduction;Cell growth and death;Lipid metabolism;Circulatory system;Endocrine system;Signal transduction;Lipid metabolism;Lipid metabolism;Lipid metabolism;Lipid metabolism
XR_003498693.1	LOC110354267	ncbi_101790585	PLA2G4A	0.964899204	3.96E-07	Global and overview maps;Signal transduction;Cell growth and death;Lipid metabolism;Circulatory system;Endocrine system;Signal transduction;Lipid metabolism;Lipid metabolism;Lipid metabolism;Lipid metabolism
XR_003498864.1	LOC113844340	ncbi_101790585	PLA2G4A	0.966437058	3.17E-07	Global and overview maps;Signal transduction;Cell growth and death;Lipid metabolism;Circulatory system;Endocrine system;Signal transduction;Lipid metabolism;Lipid metabolism;Lipid metabolism;Lipid metabolism
XR_003499063.1	LOC110353608	ncbi_101790585	PLA2G4A	0.970549989	1.66E-07	Global and overview maps;Signal transduction;Cell growth and death;Lipid metabolism;Circulatory system;Endocrine system;Signal transduction;Lipid metabolism;Lipid

						metabolism;Lipid metabolism;Lipid metabolism
XR_003499254.1	LOC106020392	ncbi_101790585	PLA2G4A	0.960572643	7.02E-07	Global and overview maps;Signal transduction;Cell growth and death;Lipid metabolism;Circulatory system;Endocrine system;Signal transduction;Lipid metabolism;Lipid metabolism;Lipid metabolism;Lipid metabolism
XR_003499720.1	LOC113844791	ncbi_101790585	PLA2G4A	0.957505973	1.02E-06	Global and overview maps;Signal transduction;Cell growth and death;Lipid metabolism;Circulatory system;Endocrine system;Signal transduction;Lipid metabolism;Lipid metabolism;Lipid metabolism;Lipid metabolism
XR_003499790.1	LOC101804048	ncbi_101790585	PLA2G4A	0.952227918	1.81E-06	Global and overview maps;Signal transduction;Cell growth and death;Lipid metabolism;Circulatory system;Endocrine system;Signal transduction;Lipid metabolism;Lipid metabolism;Lipid metabolism;Lipid metabolism
XR_003499996.1	LOC106014680	ncbi_101790585	PLA2G4A	0.954765797	1.38E-06	Global and overview maps;Signal transduction;Cell growth and death;Lipid metabolism;Circulatory system;Endocrine system;Signal transduction;Lipid metabolism;Lipid metabolism;Lipid metabolism;Lipid metabolism
XR_003500183.1	LOC106020052	ncbi_101790585	PLA2G4A	0.958447404	9.10E-07	Global and overview maps;Signal transduction;Cell growth and death;Lipid metabolism;Circulatory system;Endocrine system;Signal transduction;Lipid metabolism;Lipid metabolism;Lipid metabolism;Lipid metabolism
XR_003500301.1	LOC113845068	ncbi_101790585	PLA2G4A	0.984158339	7.65E-09	Global and overview maps;Signal transduction;Cell growth and death;Lipid metabolism;Circulatory system;Endocrine system;Signal transduction;Lipid metabolism;Lipid metabolism;Lipid metabolism;Lipid metabolism
XR_003500366.1	LOC110351294	ncbi_101790585	PLA2G4A	0.976089288	5.91E-08	Global and overview maps;Signal transduction;Cell growth

						and death;Lipid metabolism;Circulatory system;Endocrine system;Signal transduction;Lipid metabolism;Lipid metabolism;Lipid metabolism;Lipid metabolism
XR_003500722.1	LOC113845266	ncbi_101790585	PLA2G4A	0.950208072	2.22E-06	Global and overview maps;Signal transduction;Cell growth and death;Lipid metabolism;Circulatory system;Endocrine system;Signal transduction;Lipid metabolism;Lipid metabolism;Lipid metabolism;Lipid metabolism
XR_003500862.1	LOC106016001	ncbi_101790585	PLA2G4A	0.96775228	2.60E-07	Global and overview maps;Signal transduction;Cell growth and death;Lipid metabolism;Circulatory system;Endocrine system;Signal transduction;Lipid metabolism;Lipid metabolism;Lipid metabolism;Lipid metabolism
XR_003501474.1	LOC106019132	ncbi_101790585	PLA2G4A	0.974868228	7.57E-08	Global and overview maps;Signal transduction;Cell growth and death;Lipid metabolism;Circulatory system;Endocrine system;Signal transduction;Lipid metabolism;Lipid metabolism;Lipid metabolism;Lipid metabolism
MSTRG.10341.18	-	ncbi_101790851	CHKA	0.962190664	5.71E-07	Global and overview maps;Lipid metabolism
MSTRG.10914.13	-	ncbi_101790851	CHKA	0.978279763	3.67E-08	Global and overview maps;Lipid metabolism
MSTRG.10914.14	-	ncbi_101790851	CHKA	0.9639485	4.51E-07	Global and overview maps;Lipid metabolism
MSTRG.13250.1	-	ncbi_101790851	CHKA	0.957848876	9.76E-07	Global and overview maps;Lipid metabolism
MSTRG.1418.1	-	ncbi_101790851	CHKA	0.959260817	8.25E-07	Global and overview maps;Lipid metabolism
MSTRG.15815.1	-	ncbi_101790851	CHKA	-0.979928088	2.48E-08	Global and overview maps;Lipid metabolism
MSTRG.16028.1	-	ncbi_101790851	CHKA	0.95686719	1.09E-06	Global and overview maps;Lipid metabolism
MSTRG.16827.2	-	ncbi_101790851	CHKA	0.960955489	6.69E-07	Global and overview maps;Lipid metabolism
MSTRG.17696.3	-	ncbi_101790851	CHKA	0.9509163	2.07E-06	Global and overview maps;Lipid metabolism
MSTRG.2348.4	-	ncbi_101790851	CHKA	0.963081303	5.08E-07	Global and overview maps;Lipid metabolism

MSTRG.4635.4	-	ncbi_101790851	CHKA	0.988859661	1.33E-09	Global and overview maps;Lipid metabolism
MSTRG.8130.1	-	ncbi_101790851	CHKA	0.989601048	9.41E-10	Global and overview maps;Lipid metabolism
MSTRG.852.1	-	ncbi_101790851	CHKA	0.963117276	5.05E-07	Global and overview maps;Lipid metabolism
MSTRG.881.1	-	ncbi_101790851	CHKA	0.971815549	1.34E-07	Global and overview maps;Lipid metabolism
MSTRG.9008.1	-	ncbi_101790851	CHKA	0.969475725	1.98E-07	Global and overview maps;Lipid metabolism
MSTRG.9637.1	-	ncbi_101790851	CHKA	-0.97349966	9.85E-08	Global and overview maps;Lipid metabolism
XR_001186427.3	LOC106014827	ncbi_101790851	CHKA	0.956859349	1.09E-06	Global and overview maps;Lipid metabolism
XR_001194787.3	LOC106019736	ncbi_101790851	CHKA	0.953196509	1.63E-06	Global and overview maps;Lipid metabolism
XR_002398636.2	LOC110351346	ncbi_101790851	CHKA	-0.966040334	3.36E-07	Global and overview maps;Lipid metabolism
XR_002398923.2	LOC101798301	ncbi_101790851	CHKA	0.966319779	3.23E-07	Global and overview maps;Lipid metabolism
XR_002402383.2	LOC110352801	ncbi_101790851	CHKA	0.950497471	2.15E-06	Global and overview maps;Lipid metabolism
XR_002402646.2	LOC101794014	ncbi_101790851	CHKA	0.960241043	7.32E-07	Global and overview maps;Lipid metabolism
XR_002404953.2	LOC110353849	ncbi_101790851	CHKA	0.960263076	7.30E-07	Global and overview maps;Lipid metabolism
XR_002405065.2	LOC106019134	ncbi_101790851	CHKA	-0.984537628	6.78E-09	Global and overview maps;Lipid metabolism
XR_003492209.1	LOC106016624	ncbi_101790851	CHKA	-0.96319972	5.00E-07	Global and overview maps;Lipid metabolism
XR_003492632.1	LOC106018295	ncbi_101790851	CHKA	0.97512456	7.19E-08	Global and overview maps;Lipid metabolism
XR_003494880.1	LOC106020483	ncbi_101790851	CHKA	0.954863665	1.37E-06	Global and overview maps;Lipid metabolism
XR_003495202.1	LOC113842645	ncbi_101790851	CHKA	0.959579635	7.94E-07	Global and overview maps;Lipid metabolism
XR_003495207.1	LOC113842649	ncbi_101790851	CHKA	0.962787139	5.28E-07	Global and overview maps;Lipid metabolism
XR_003497162.1	LOC113843598	ncbi_101790851	CHKA	0.957451174	1.02E-06	Global and overview maps;Lipid metabolism
XR_003497745.1	LOC110353307	ncbi_101790851	CHKA	0.97590084	6.15E-08	Global and overview maps;Lipid metabolism
XR_003499790.1	LOC101804048	ncbi_101790851	CHKA	0.964270643	4.32E-07	Global and overview maps;Lipid metabolism
XR_003499961.1	LOC110352806	ncbi_101790851	CHKA	0.96923017	2.06E-07	Global and overview maps;Lipid metabolism

XR_003499966.1	LOC110352806	ncbi_101790851	CHKA	0.964400455	4.24E-07	Global and overview maps;Lipid metabolism
XR_003500051.1	LOC113844972	ncbi_101790851	CHKA	0.960891325	6.75E-07	Global and overview maps;Lipid metabolism
XR_003500052.1	LOC113844972	ncbi_101790851	CHKA	0.96947389	1.98E-07	Global and overview maps;Lipid metabolism
XR_003500671.1	LOC106019837	ncbi_101790851	CHKA	0.967334839	2.77E-07	Global and overview maps;Lipid metabolism
XR_003500680.1	LOC110352551	ncbi_101790851	CHKA	0.966011064	3.37E-07	Global and overview maps;Lipid metabolism
XR_003501403.1	LOC113845679	ncbi_101790851	CHKA	0.961845816	5.97E-07	Global and overview maps;Lipid metabolism
XR_217495.4	LOC101790744	ncbi_101790851	CHKA	0.982451683	1.27E-08	Global and overview maps;Lipid metabolism
MSTRG.10914.13	-	ncbi_101792626	FABP4	-0.966839017	2.99E-07	Endocrine system
MSTRG.10914.14	-	ncbi_101792626	FABP4	-0.962412309	5.55E-07	Endocrine system
MSTRG.124.1	-	ncbi_101792626	FABP4	0.95784364	9.77E-07	Endocrine system
MSTRG.13250.1	-	ncbi_101792626	FABP4	-0.959446492	8.07E-07	Endocrine system
MSTRG.15815.1	-	ncbi_101792626	FABP4	0.96772241	2.61E-07	Endocrine system
MSTRG.16028.1	-	ncbi_101792626	FABP4	-0.952725988	1.72E-06	Endocrine system
MSTRG.17696.3	-	ncbi_101792626	FABP4	-0.968645144	2.26E-07	Endocrine system
MSTRG.4635.4	-	ncbi_101792626	FABP4	-0.973871241	9.18E-08	Endocrine system
MSTRG.624.1	-	ncbi_101792626	FABP4	0.955533948	1.27E-06	Endocrine system
MSTRG.759.2	-	ncbi_101792626	FABP4	-0.984552419	6.75E-09	Endocrine system
MSTRG.8130.1	-	ncbi_101792626	FABP4	-0.974717305	7.80E-08	Endocrine system

XR_001193935.3	LOC106019263	ncbi_101792626	FABP4	-0.969563336	1.95E-07	Endocrine system
XR_002398636.2	LOC110351346	ncbi_101792626	FABP4	0.980061804	2.40E-08	Endocrine system
XR_002398923.2	LOC101798301	ncbi_101792626	FABP4	-0.962134205	5.75E-07	Endocrine system
XR_002402026.2	LOC110352675	ncbi_101792626	FABP4	-0.97294279	1.09E-07	Endocrine system
XR_002404953.2	LOC110353849	ncbi_101792626	FABP4	-0.975933204	6.11E-08	Endocrine system
XR_002405065.2	LOC106019134	ncbi_101792626	FABP4	0.962391878	5.56E-07	Endocrine system
XR_003492209.1	LOC106016624	ncbi_101792626	FABP4	0.951638518	1.92E-06	Endocrine system
XR_003496853.1	LOC101805095	ncbi_101792626	FABP4	-0.97144445	1.43E-07	Endocrine system
XR_003497162.1	LOC113843598	ncbi_101792626	FABP4	-0.958862632	8.66E-07	Endocrine system
XR_003497745.1	LOC110353307	ncbi_101792626	FABP4	-0.957235784	1.05E-06	Endocrine system
XR_003500052.1	LOC113844972	ncbi_101792626	FABP4	-0.955513861	1.27E-06	Endocrine system
XR_003500680.1	LOC110352551	ncbi_101792626	FABP4	-0.951355778	1.98E-06	Endocrine system
XR_003500864.1	LOC113845332	ncbi_101792626	FABP4	-0.966709959	3.04E-07	Endocrine system
XR_216772.4	LOC101802284	ncbi_101792626	FABP4	-0.951935785	1.86E-06	Endocrine system
MSTRG.14276.1	-	ncbi_101793285	FABP7	0.988375623	1.64E-09	Endocrine system
MSTRG.2671.3	-	ncbi_101793285	FABP7	0.968031918	2.49E-07	Endocrine system
MSTRG.338.3	-	ncbi_101793285	FABP7	0.951264751	1.99E-06	Endocrine system

MSTRG.4759.1	-	ncbi_101793285	FABP7	0.959499931	8.02E-07	Endocrine system
MSTRG.8490.1	-	ncbi_101793285	FABP7	0.96475963	4.03E-07	Endocrine system
XR_001191013.3	LOC106017519	ncbi_101793285	FABP7	0.974953643	7.44E-08	Endocrine system
XR_002398562.2	LOC106014525	ncbi_101793285	FABP7	0.957416059	1.03E-06	Endocrine system
XR_002401163.2	LOC110352334	ncbi_101793285	FABP7	0.98157188	1.62E-08	Endocrine system
XR_002402751.2	LOC110352931	ncbi_101793285	FABP7	0.983169314	1.03E-08	Endocrine system
XR_002402756.2	LOC110352934	ncbi_101793285	FABP7	0.974536821	8.08E-08	Endocrine system
XR_002402765.2	LOC110352941	ncbi_101793285	FABP7	0.957519524	1.01E-06	Endocrine system
XR_002403094.2	LOC110353080	ncbi_101793285	FABP7	0.959169621	8.34E-07	Endocrine system
XR_003492849.1	LOC113840117	ncbi_101793285	FABP7	0.958116346	9.46E-07	Endocrine system
XR_003493825.1	LOC113841183	ncbi_101793285	FABP7	0.983368094	9.75E-09	Endocrine system
XR_003496855.1	LOC113843509	ncbi_101793285	FABP7	-0.973049506	1.07E-07	Endocrine system
XR_003497368.1	LOC110352930	ncbi_101793285	FABP7	0.957662502	9.98E-07	Endocrine system
XR_003497399.1	LOC110352933	ncbi_101793285	FABP7	0.97503808	7.32E-08	Endocrine system
XR_003498160.1	LOC113844040	ncbi_101793285	FABP7	0.951508764	1.95E-06	Endocrine system
MSTRG.11582.4	-	ncbi_101795388	FABP3	0.966487647	3.15E-07	Endocrine system
MSTRG.13804.1	-	ncbi_101795388	FABP3	0.983963325	8.13E-09	Endocrine system
MSTRG.13937.5	-	ncbi_101795388	FABP3	0.992578312	1.75E-10	Endocrine system
MSTRG.17302.1	-	ncbi_101795388	FABP3	0.961907455	5.92E-07	Endocrine system
MSTRG.6393.1	-	ncbi_101795388	FABP3	0.97259756	1.16E-07	Endocrine system
MSTRG.7980.2	-	ncbi_101795388	FABP3	0.994888294	2.73E-11	Endocrine system
XR_003494980.1	LOC113842453	ncbi_101795388	FABP3	0.968816178	2.20E-07	Endocrine system
XR_003495821.1	LOC113842909	ncbi_101795388	FABP3	0.95270646	1.72E-06	Endocrine system
XR_003497206.1	LOC113843623	ncbi_101795388	FABP3	0.988725046	1.41E-09	Endocrine system

XR_003498718.1	LOC113844276	ncbi_101795388	FABP3	0.988606181	1.48E-09	Endocrine system
XR_003499613.1	LOC113844759	ncbi_101795388	FABP3	0.962798223	5.27E-07	Endocrine system
XR_003501551.1	LOC113845772	ncbi_101795388	FABP3	0.984722613	6.39E-09	Endocrine system
MSTRG.10098.1	-	ncbi_101795561	Elovl5	0.993229015	1.11E-10	-
MSTRG.10100.1	-	ncbi_101795561	Elovl5	0.98184132	1.51E-08	-
MSTRG.10341.18	-	ncbi_101795561	Elovl5	0.976663807	5.24E-08	-
MSTRG.10341.19	-	ncbi_101795561	Elovl5	0.983572282	9.17E-09	-
MSTRG.10341.20	-	ncbi_101795561	Elovl5	0.991588599	3.27E-10	-
MSTRG.10341.21	-	ncbi_101795561	Elovl5	0.98116094	1.81E-08	-
MSTRG.10341.22	-	ncbi_101795561	Elovl5	0.992217875	2.22E-10	-
MSTRG.12747.4	-	ncbi_101795561	Elovl5	0.958437815	9.11E-07	-
MSTRG.13250.1	-	ncbi_101795561	Elovl5	0.965022725	3.89E-07	-
MSTRG.13544.2	-	ncbi_101795561	Elovl5	0.978673465	3.35E-08	-
MSTRG.14551.1	-	ncbi_101795561	Elovl5	-0.960028403	7.51E-07	-
MSTRG.15964.1	-	ncbi_101795561	Elovl5	0.95404539	1.49E-06	-
MSTRG.15965.1	-	ncbi_101795561	Elovl5	0.962302947	5.63E-07	-
MSTRG.16028.1	-	ncbi_101795561	Elovl5	0.971771861	1.35E-07	-
MSTRG.16827.2	-	ncbi_101795561	Elovl5	0.960808295	6.82E-07	-
MSTRG.16889.1	-	ncbi_101795561	Elovl5	0.966056169	3.35E-07	-
MSTRG.1816.1	-	ncbi_101795561	Elovl5	0.960298835	7.27E-07	-
MSTRG.2348.4	-	ncbi_101795561	Elovl5	0.975361936	6.86E-08	-
MSTRG.2606.1	-	ncbi_101795561	Elovl5	0.986049305	4.07E-09	-
MSTRG.2608.1	-	ncbi_101795561	Elovl5	0.959623639	7.90E-07	-
MSTRG.2904.1	-	ncbi_101795561	Elovl5	0.975211291	7.07E-08	-

MSTRG.3080.1	-	ncbi_101795561	Elovl5	0.971466258	1.42E-07	-
MSTRG.3622.7	-	ncbi_101795561	Elovl5	0.967285022	2.79E-07	-
MSTRG.3639.1	-	ncbi_101795561	Elovl5	0.963567554	4.75E-07	-
MSTRG.4893.1	-	ncbi_101795561	Elovl5	0.954639837	1.40E-06	-
MSTRG.5011.1	-	ncbi_101795561	Elovl5	0.968169543	2.44E-07	-
MSTRG.5066.1	-	ncbi_101795561	Elovl5	-0.958408619	9.14E-07	-
MSTRG.5135.1	-	ncbi_101795561	Elovl5	0.981968461	1.46E-08	-
MSTRG.5135.3	-	ncbi_101795561	Elovl5	0.988747908	1.39E-09	-
MSTRG.5601.1	-	ncbi_101795561	Elovl5	0.952073242	1.84E-06	-
MSTRG.582.3	-	ncbi_101795561	Elovl5	0.956930551	1.09E-06	-
MSTRG.5846.1	-	ncbi_101795561	Elovl5	0.955222607	1.31E-06	-
MSTRG.5881.2	-	ncbi_101795561	Elovl5	0.959788868	7.74E-07	-
MSTRG.5997.1	-	ncbi_101795561	Elovl5	0.95605504	1.20E-06	-
MSTRG.6127.1	-	ncbi_101795561	Elovl5	0.96277133	5.29E-07	-
MSTRG.6316.1	-	ncbi_101795561	Elovl5	-0.980109406	2.37E-08	-
MSTRG.6704.2	-	ncbi_101795561	Elovl5	0.971692567	1.37E-07	-
MSTRG.6704.3	-	ncbi_101795561	Elovl5	0.97478191	7.70E-08	-
MSTRG.6704.4	-	ncbi_101795561	Elovl5	0.982958001	1.10E-08	-
MSTRG.715.1	-	ncbi_101795561	Elovl5	0.96954335	1.96E-07	-
MSTRG.7239.1	-	ncbi_101795561	Elovl5	0.965454341	3.66E-07	-
MSTRG.7248.1	-	ncbi_101795561	Elovl5	0.979325431	2.87E-08	-
MSTRG.756.3	-	ncbi_101795561	Elovl5	0.988034697	1.89E-09	-
MSTRG.7621.5	-	ncbi_101795561	Elovl5	0.962149908	5.74E-07	-

MSTRG.7949.1	-	ncbi_101795561	Elovl5	0.954360334	1.44E-06	-
MSTRG.7954.1	-	ncbi_101795561	Elovl5	0.957779611	9.84E-07	-
MSTRG.8130.1	-	ncbi_101795561	Elovl5	0.950556709	2.14E-06	-
MSTRG.852.1	-	ncbi_101795561	Elovl5	0.984839103	6.15E-09	-
MSTRG.885.1	-	ncbi_101795561	Elovl5	0.971846352	1.33E-07	-
MSTRG.889.1	-	ncbi_101795561	Elovl5	0.97215715	1.26E-07	-
MSTRG.9008.1	-	ncbi_101795561	Elovl5	0.97945269	2.79E-08	-
MSTRG.9062.2	-	ncbi_101795561	Elovl5	0.960334554	7.23E-07	-
MSTRG.9637.1	-	ncbi_101795561	Elovl5	-0.950011472	2.26E-06	-
XR_001185755.3	LOC106014431	ncbi_101795561	Elovl5	0.95489391	1.36E-06	-
XR_001186561.3	LOC106014905	ncbi_101795561	Elovl5	0.958703375	8.82E-07	-
XR_001186606.3	LOC106014935	ncbi_101795561	Elovl5	0.960631544	6.97E-07	-
XR_001186962.3	LOC106015138	ncbi_101795561	Elovl5	0.952431239	1.77E-06	-
XR_001187862.3	LOC106015672	ncbi_101795561	Elovl5	0.956394749	1.15E-06	-
XR_001187865.3	LOC106015672	ncbi_101795561	Elovl5	0.958344556	9.21E-07	-
XR_001187947.2	LOC106015729	ncbi_101795561	Elovl5	0.956280036	1.17E-06	-
XR_001187991.3	LOC106015755	ncbi_101795561	Elovl5	0.954888853	1.36E-06	-
XR_001188444.3	LOC106016033	ncbi_101795561	Elovl5	0.967859725	2.56E-07	-
XR_001189917.2	LOC106016889	ncbi_101795561	Elovl5	0.968087617	2.47E-07	-
XR_001189941.3	LOC106016901	ncbi_101795561	Elovl5	0.953097702	1.65E-06	-
XR_001190278.3	LOC106017074	ncbi_101795561	Elovl5	0.959423792	8.09E-07	-
XR_001190677.3	LOC106017297	ncbi_101795561	Elovl5	0.958807321	8.72E-07	-
XR_001193197.3	LOC106018814	ncbi_101795561	Elovl5	0.968481552	2.32E-07	-
XR_001193335.3	LOC106018911	ncbi_101795561	Elovl5	0.966452106	3.16E-07	-

XR_001193468.3	LOC106018998	ncbi_101795561	Elovl5	0.961076532	6.59E-07	-
XR_001193619.3	LOC106019066	ncbi_101795561	Elovl5	0.965569092	3.60E-07	-
XR_001194132.2	LOC106019368	ncbi_101795561	Elovl5	0.956271415	1.17E-06	-
XR_001194441.2	LOC106019549	ncbi_101795561	Elovl5	0.958290454	9.27E-07	-
XR_001194787.3	LOC106019736	ncbi_101795561	Elovl5	0.960483549	7.10E-07	-
XR_002398851.2	LOC106014736	ncbi_101795561	Elovl5	0.951943949	1.86E-06	-
XR_002398923.2	LOC101798301	ncbi_101795561	Elovl5	0.974285142	8.48E-08	-
XR_002399173.2	LOC110351568	ncbi_101795561	Elovl5	0.958504648	9.04E-07	-
XR_002399314.2	LOC106015131	ncbi_101795561	Elovl5	0.965146451	3.82E-07	-
XR_002399432.2	LOC110351688	ncbi_101795561	Elovl5	0.953298561	1.62E-06	-
XR_002399447.2	LOC110351699	ncbi_101795561	Elovl5	0.951039349	2.04E-06	-
XR_002399732.1	LOC110351814	ncbi_101795561	Elovl5	0.961080934	6.59E-07	-
XR_002399836.2	LOC106015519	ncbi_101795561	Elovl5	0.967811599	2.58E-07	-
XR_002399884.2	LOC110351873	ncbi_101795561	Elovl5	0.961623544	6.15E-07	-
XR_002399898.2	LOC110351880	ncbi_101795561	Elovl5	0.952038339	1.84E-06	-
XR_002400226.2	LOC106015733	ncbi_101795561	Elovl5	0.952948489	1.68E-06	-
XR_002400249.2	LOC110351990	ncbi_101795561	Elovl5	0.952557887	1.75E-06	-
XR_002400594.2	LOC110352134	ncbi_101795561	Elovl5	0.980009082	2.43E-08	-
XR_002400870.1	LOC110352222	ncbi_101795561	Elovl5	0.952828006	1.70E-06	-
XR_002401026.2	LOC110352298	ncbi_101795561	Elovl5	0.957381788	1.03E-06	-
XR_002401202.2	LOC110352351	ncbi_101795561	Elovl5	0.957479943	1.02E-06	-
XR_002401479.2	LOC106016692	ncbi_101795561	Elovl5	0.951992609	1.85E-06	-
XR_002401866.2	LOC106016894	ncbi_101795561	Elovl5	0.957346542	1.03E-06	-
XR_002401875.2	LOC110352627	ncbi_101795561	Elovl5	0.956345971	1.16E-06	-
XR_002402176.2	LOC110352712	ncbi_101795561	Elovl5	0.95567263	1.25E-06	-

XR_002402383.2	LOC110352801	ncbi_101795561	Elovl5	0.95874024	8.79E-07	-
XR_002402646.2	LOC101794014	ncbi_101795561	Elovl5	0.993824916	7.00E-11	-
XR_002402718.2	LOC106017475	ncbi_101795561	Elovl5	0.960839117	6.79E-07	-
XR_002403184.2	LOC106017746	ncbi_101795561	Elovl5	0.955788873	1.23E-06	-
XR_002404947.2	LOC110353846	ncbi_101795561	Elovl5	0.982929937	1.11E-08	-
XR_002405065.2	LOC106019134	ncbi_101795561	Elovl5	-0.964849573	3.98E-07	-
XR_002405449.2	LOC110354050	ncbi_101795561	Elovl5	0.96145417	6.28E-07	-
XR_002405521.2	LOC106019445	ncbi_101795561	Elovl5	0.950130907	2.23E-06	-
XR_002405535.2	LOC110354079	ncbi_101795561	Elovl5	0.963067007	5.09E-07	-
XR_002405612.2	LOC106019471	ncbi_101795561	Elovl5	0.955886046	1.22E-06	-
XR_002405843.2	LOC106019674	ncbi_101795561	Elovl5	0.959133219	8.38E-07	-
XR_002405947.2	LOC106019753	ncbi_101795561	Elovl5	0.965904911	3.43E-07	-
XR_002406139.2	LOC110354333	ncbi_101795561	Elovl5	0.971273733	1.47E-07	-
XR_002406465.2	LOC106020114	ncbi_101795561	Elovl5	0.965871986	3.44E-07	-
XR_002406489.2	LOC106020129	ncbi_101795561	Elovl5	0.977385274	4.49E-08	-
XR_002406729.2	LOC106020349	ncbi_101795561	Elovl5	0.986262496	3.77E-09	-
XR_002406750.2	LOC106020393	ncbi_101795561	Elovl5	0.961114095	6.56E-07	-
XR_003492182.1	LOC113839646	ncbi_101795561	Elovl5	0.969024392	2.13E-07	-
XR_003492300.1	LOC110354130	ncbi_101795561	Elovl5	0.954931984	1.36E-06	-
XR_003492632.1	LOC106018295	ncbi_101795561	Elovl5	0.96903548	2.13E-07	-
XR_003492832.1	LOC113840106	ncbi_101795561	Elovl5	0.950817267	2.09E-06	-
XR_003493103.1	LOC113840309	ncbi_101795561	Elovl5	0.959328205	8.19E-07	-
XR_003493806.1	LOC113841174	ncbi_101795561	Elovl5	0.973198215	1.04E-07	-
XR_003494664.1	LOC113842319	ncbi_101795561	Elovl5	0.972508704	1.18E-07	-

XR_003494775.1	LOC110352361	ncbi_101795561	Elovl5	0.95489391	1.36E-06	-
XR_003494880.1	LOC106020483	ncbi_101795561	Elovl5	0.972091015	1.27E-07	-
XR_003494883.1	LOC106020483	ncbi_101795561	Elovl5	0.961137778	6.54E-07	-
XR_003494887.1	LOC113842395	ncbi_101795561	Elovl5	0.951408315	1.97E-06	-
XR_003494888.1	LOC113842396	ncbi_101795561	Elovl5	0.972396787	1.20E-07	-
XR_003494957.1	LOC101799909	ncbi_101795561	Elovl5	0.968769908	2.22E-07	-
XR_003494960.1	LOC101799909	ncbi_101795561	Elovl5	0.953682631	1.55E-06	-
XR_003494961.1	LOC113842447	ncbi_101795561	Elovl5	0.974237223	8.56E-08	-
XR_003495202.1	LOC113842645	ncbi_101795561	Elovl5	0.975660414	6.46E-08	-
XR_003495207.1	LOC113842649	ncbi_101795561	Elovl5	0.986549539	3.39E-09	-
XR_003495345.1	LOC101793386	ncbi_101795561	Elovl5	0.974542961	8.07E-08	-
XR_003495483.1	LOC113842770	ncbi_101795561	Elovl5	0.971816393	1.34E-07	-
XR_003495767.1	LOC110352593	ncbi_101795561	Elovl5	0.965336378	3.72E-07	-
XR_003495918.1	LOC113843035	ncbi_101795561	Elovl5	0.986752888	3.14E-09	-
XR_003495930.1	LOC113839603	ncbi_101795561	Elovl5	0.952734754	1.72E-06	-
XR_003496139.1	LOC113843116	ncbi_101795561	Elovl5	0.974176522	8.66E-08	-
XR_003496334.1	LOC113843176	ncbi_101795561	Elovl5	0.988841215	1.34E-09	-
XR_003496440.1	LOC106015283	ncbi_101795561	Elovl5	0.965719044	3.52E-07	-
XR_003496447.1	LOC113843228	ncbi_101795561	Elovl5	0.954433393	1.43E-06	-
XR_003497001.1	LOC106017547	ncbi_101795561	Elovl5	0.98827592	1.71E-09	-
XR_003497002.1	LOC106017547	ncbi_101795561	Elovl5	0.954167565	1.47E-06	-
XR_003497023.1	LOC113843564	ncbi_101795561	Elovl5	0.959383033	8.13E-07	-
XR_003497032.1	LOC106020477	ncbi_101795561	Elovl5	0.964979895	3.91E-07	-
XR_003497296.1	LOC113843645	ncbi_101795561	Elovl5	0.963347316	4.90E-07	-
XR_003497481.1	LOC110351804	ncbi_101795561	Elovl5	0.959224904	8.29E-07	-

XR_003497529.1	LOC113843776	ncbi_101795561	Elovl5	0.972274047	1.23E-07	-
XR_003497554.1	LOC110354397	ncbi_101795561	Elovl5	0.961116612	6.56E-07	-
XR_003497564.1	LOC101802970	ncbi_101795561	Elovl5	0.962507195	5.48E-07	-
XR_003497566.1	LOC101802970	ncbi_101795561	Elovl5	0.986267614	3.76E-09	-
XR_003497661.1	LOC101798800	ncbi_101795561	Elovl5	0.970302368	1.73E-07	-
XR_003497745.1	LOC110353307	ncbi_101795561	Elovl5	0.987346815	2.50E-09	-
XR_003497951.1	LOC113843960	ncbi_101795561	Elovl5	0.961350984	6.36E-07	-
XR_003498021.1	LOC101803684	ncbi_101795561	Elovl5	0.952040923	1.84E-06	-
XR_003498491.1	LOC113844169	ncbi_101795561	Elovl5	0.975340617	6.89E-08	-
XR_003498693.1	LOC110354267	ncbi_101795561	Elovl5	0.956052451	1.20E-06	-
XR_003498805.1	LOC106019442	ncbi_101795561	Elovl5	0.951184839	2.01E-06	-
XR_003498864.1	LOC113844340	ncbi_101795561	Elovl5	0.970044403	1.81E-07	-
XR_003499063.1	LOC110353608	ncbi_101795561	Elovl5	0.981998458	1.44E-08	-
XR_003499624.1	LOC113844766	ncbi_101795561	Elovl5	0.955545568	1.27E-06	-
XR_003499720.1	LOC113844791	ncbi_101795561	Elovl5	0.963602556	4.73E-07	-
XR_003499753.1	LOC113844812	ncbi_101795561	Elovl5	0.952979892	1.67E-06	-
XR_003499790.1	LOC101804048	ncbi_101795561	Elovl5	0.986000855	4.14E-09	-
XR_003499791.1	LOC113844835	ncbi_101795561	Elovl5	0.975873629	6.18E-08	-
XR_003499827.1	LOC106019660	ncbi_101795561	Elovl5	0.968405678	2.35E-07	-
XR_003499961.1	LOC110352806	ncbi_101795561	Elovl5	0.976166491	5.82E-08	-
XR_003499966.1	LOC110352806	ncbi_101795561	Elovl5	0.969032662	2.13E-07	-
XR_003500183.1	LOC106020052	ncbi_101795561	Elovl5	0.977238959	4.63E-08	-
XR_003500301.1	LOC113845068	ncbi_101795561	Elovl5	0.96569203	3.53E-07	-
XR_003500366.1	LOC110351294	ncbi_101795561	Elovl5	0.964061075	4.45E-07	-
XR_003500671.1	LOC106019837	ncbi_101795561	Elovl5	0.982601982	1.22E-08	-

XR_003500722.1	LOC113845266	ncbi_101795561	Elovl5	0.968980609	2.15E-07	-
XR_003501403.1	LOC113845679	ncbi_101795561	Elovl5	0.984093906	7.81E-09	-
XR_003501474.1	LOC106019132	ncbi_101795561	Elovl5	0.958426528	9.12E-07	-
XR_003501504.1	LOC113845747	ncbi_101795561	Elovl5	0.959601222	7.92E-07	-
MSTRG.11490.3	-	ncbi_101796497	Pnpla2	0.950496344	2.15E-06	Global and overview maps;Lipid metabolism
MSTRG.2341.4	-	ncbi_101796497	Pnpla2	0.975788531	6.29E-08	Global and overview maps;Lipid metabolism
MSTRG.3461.1	-	ncbi_101796497	Pnpla2	0.962218466	5.69E-07	Global and overview maps;Lipid metabolism
MSTRG.4554.1	-	ncbi_101796497	Pnpla2	-0.962161816	5.73E-07	Global and overview maps;Lipid metabolism
MSTRG.624.1	-	ncbi_101796497	Pnpla2	0.966700117	3.05E-07	Global and overview maps;Lipid metabolism
XR_002398922.2	LOC101798301	ncbi_101796497	Pnpla2	-0.952133846	1.83E-06	Global and overview maps;Lipid metabolism
XR_003494155.1	LOC113841719	ncbi_101796497	Pnpla2	0.986596145	3.33E-09	Global and overview maps;Lipid metabolism
XR_003497485.1	LOC113843765	ncbi_101796497	Pnpla2	0.966826004	2.99E-07	Global and overview maps;Lipid metabolism
MSTRG.14276.1	-	ncbi_101797310	ST8SIA1	0.97217924	1.25E-07	Global and overview maps;Glycan biosynthesis and metabolism;Glycan biosynthesis and metabolism;Glycan biosynthesis and metabolism
MSTRG.3256.4	-	ncbi_101797310	ST8SIA1	-0.95880497	8.72E-07	Global and overview maps;Glycan biosynthesis and metabolism;Glycan biosynthesis and metabolism;Glycan biosynthesis and metabolism
MSTRG.338.3	-	ncbi_101797310	ST8SIA1	0.953364328	1.61E-06	Global and overview maps;Glycan biosynthesis and metabolism;Glycan biosynthesis and metabolism;Glycan biosynthesis and metabolism
MSTRG.8490.1	-	ncbi_101797310	ST8SIA1	0.973014987	1.08E-07	Global and overview maps;Glycan biosynthesis and metabolism;Glycan biosynthesis and metabolism;Glycan biosynthesis and metabolism

MSTRG.8536.1	-	ncbi_101797310	ST8SIA1	0.957101683	1.06E-06	Global and overview maps;Glycan biosynthesis and metabolism;Glycan biosynthesis and metabolism;Glycan biosynthesis and metabolism
XR_001187856.3	LOC106015665	ncbi_101797310	ST8SIA1	-0.959089865	8.42E-07	Global and overview maps;Glycan biosynthesis and metabolism;Glycan biosynthesis and metabolism;Glycan biosynthesis and metabolism
XR_001191530.3	LOC106017832	ncbi_101797310	ST8SIA1	0.954906176	1.36E-06	Global and overview maps;Glycan biosynthesis and metabolism;Glycan biosynthesis and metabolism;Glycan biosynthesis and metabolism
XR_002398783.2	LOC106014662	ncbi_101797310	ST8SIA1	0.965859649	3.45E-07	Global and overview maps;Glycan biosynthesis and metabolism;Glycan biosynthesis and metabolism;Glycan biosynthesis and metabolism
XR_002399433.2	LOC110351689	ncbi_101797310	ST8SIA1	0.953171969	1.64E-06	Global and overview maps;Glycan biosynthesis and metabolism;Glycan biosynthesis and metabolism;Glycan biosynthesis and metabolism
XR_002399903.2	LOC110351882	ncbi_101797310	ST8SIA1	0.984149889	7.67E-09	Global and overview maps;Glycan biosynthesis and metabolism;Glycan biosynthesis and metabolism;Glycan biosynthesis and metabolism
XR_002401163.2	LOC110352334	ncbi_101797310	ST8SIA1	0.971686267	1.37E-07	Global and overview maps;Glycan biosynthesis and metabolism;Glycan biosynthesis and metabolism;Glycan biosynthesis and metabolism
XR_002402751.2	LOC110352931	ncbi_101797310	ST8SIA1	0.97689622	4.99E-08	Global and overview maps;Glycan biosynthesis and metabolism;Glycan biosynthesis and metabolism;Glycan biosynthesis and metabolism
XR_002402756.2	LOC110352934	ncbi_101797310	ST8SIA1	0.962681797	5.35E-07	Global and overview maps;Glycan biosynthesis and metabolism;Glycan biosynthesis and metabolism;Glycan

						biosynthesis and metabolism
XR_002403094.2	LOC110353080	ncbi_101797310	ST8SIA1	0.960473441	7.11E-07	Global and overview maps;Glycan biosynthesis and metabolism;Glycan biosynthesis and metabolism;Glycan biosynthesis and metabolism
XR_003492815.1	LOC113840095	ncbi_101797310	ST8SIA1	-0.951307589	1.99E-06	Global and overview maps;Glycan biosynthesis and metabolism;Glycan biosynthesis and metabolism;Glycan biosynthesis and metabolism
XR_003493825.1	LOC113841183	ncbi_101797310	ST8SIA1	0.976224795	5.75E-08	Global and overview maps;Glycan biosynthesis and metabolism;Glycan biosynthesis and metabolism;Glycan biosynthesis and metabolism
XR_003494768.1	LOC106018037	ncbi_101797310	ST8SIA1	0.96632777	3.22E-07	Global and overview maps;Glycan biosynthesis and metabolism;Glycan biosynthesis and metabolism;Glycan biosynthesis and metabolism
XR_003494770.1	LOC106018037	ncbi_101797310	ST8SIA1	0.951097265	2.03E-06	Global and overview maps;Glycan biosynthesis and metabolism;Glycan biosynthesis and metabolism;Glycan biosynthesis and metabolism
XR_003495957.1	LOC113843061	ncbi_101797310	ST8SIA1	0.962976253	5.15E-07	Global and overview maps;Glycan biosynthesis and metabolism;Glycan biosynthesis and metabolism;Glycan biosynthesis and metabolism
XR_003496239.1	LOC110352708	ncbi_101797310	ST8SIA1	0.953274574	1.62E-06	Global and overview maps;Glycan biosynthesis and metabolism;Glycan biosynthesis and metabolism;Glycan biosynthesis and metabolism
XR_003496855.1	LOC113843509	ncbi_101797310	ST8SIA1	-0.978599855	3.41E-08	Global and overview maps;Glycan biosynthesis and metabolism;Glycan biosynthesis and metabolism;Glycan biosynthesis and metabolism
XR_003497095.1	LOC106015748	ncbi_101797310	ST8SIA1	0.959421667	8.09E-07	Global and overview maps;Glycan biosynthesis and

						metabolism;Glycan biosynthesis and metabolism;Glycan biosynthesis and metabolism
XR_003497399.1	LOC110352933	ncbi_101797310	ST8SIA1	0.957250204	1.05E-06	Global and overview maps;Glycan biosynthesis and metabolism;Glycan biosynthesis and metabolism;Glycan biosynthesis and metabolism
XR_003498160.1	LOC113844040	ncbi_101797310	ST8SIA1	0.968106646	2.46E-07	Global and overview maps;Glycan biosynthesis and metabolism;Glycan biosynthesis and metabolism;Glycan biosynthesis and metabolism
XR_003500629.1	LOC113845225	ncbi_101797310	ST8SIA1	-0.956936412	1.08E-06	Global and overview maps;Glycan biosynthesis and metabolism;Glycan biosynthesis and metabolism;Glycan biosynthesis and metabolism
MSTRG.10098.1	-	ncbi_101798758	FADS2	0.97619224	5.79E-08	Endocrine system;Global and overview maps;Lipid metabolism;Lipid metabolism
MSTRG.10100.1	-	ncbi_101798758	FADS2	0.979478539	2.77E-08	Endocrine system;Global and overview maps;Lipid metabolism;Lipid metabolism
MSTRG.10341.18	-	ncbi_101798758	FADS2	0.975044576	7.31E-08	Endocrine system;Global and overview maps;Lipid metabolism;Lipid metabolism
MSTRG.10341.19	-	ncbi_101798758	FADS2	0.990014766	7.69E-10	Endocrine system;Global and overview maps;Lipid metabolism;Lipid metabolism
MSTRG.10341.20	-	ncbi_101798758	FADS2	0.991630866	3.19E-10	Endocrine system;Global and overview maps;Lipid metabolism;Lipid metabolism
MSTRG.10341.21	-	ncbi_101798758	FADS2	0.990316578	6.60E-10	Endocrine system;Global and overview maps;Lipid metabolism;Lipid metabolism
MSTRG.10341.22	-	ncbi_101798758	FADS2	0.992382213	1.99E-10	Endocrine system;Global and overview maps;Lipid metabolism;Lipid metabolism
MSTRG.12747.4	-	ncbi_101798758	FADS2	0.975674588	6.44E-08	Endocrine system;Global and overview maps;Lipid

						metabolism;Lipid metabolism
MSTRG.12949.1	-	ncbi_101798758	FADS2	0.959491491	8.02E-07	Endocrine system;Global and overview maps;Lipid metabolism;Lipid metabolism
MSTRG.13538.3	-	ncbi_101798758	FADS2	0.968467592	2.33E-07	Endocrine system;Global and overview maps;Lipid metabolism;Lipid metabolism
MSTRG.13544.2	-	ncbi_101798758	FADS2	0.988591216	1.49E-09	Endocrine system;Global and overview maps;Lipid metabolism;Lipid metabolism
MSTRG.13915.1	-	ncbi_101798758	FADS2	0.976732909	5.16E-08	Endocrine system;Global and overview maps;Lipid metabolism;Lipid metabolism
MSTRG.14551.1	-	ncbi_101798758	FADS2	-0.964323251	4.29E-07	Endocrine system;Global and overview maps;Lipid metabolism;Lipid metabolism
MSTRG.15964.1	-	ncbi_101798758	FADS2	0.969424658	2.00E-07	Endocrine system;Global and overview maps;Lipid metabolism;Lipid metabolism
MSTRG.15965.1	-	ncbi_101798758	FADS2	0.992459885	1.90E-10	Endocrine system;Global and overview maps;Lipid metabolism;Lipid metabolism
MSTRG.16028.1	-	ncbi_101798758	FADS2	0.969581328	1.95E-07	Endocrine system;Global and overview maps;Lipid metabolism;Lipid metabolism
MSTRG.16889.1	-	ncbi_101798758	FADS2	0.982198682	1.37E-08	Endocrine system;Global and overview maps;Lipid metabolism;Lipid metabolism
MSTRG.1816.1	-	ncbi_101798758	FADS2	0.955970729	1.21E-06	Endocrine system;Global and overview maps;Lipid metabolism;Lipid metabolism
MSTRG.2348.4	-	ncbi_101798758	FADS2	0.95690927	1.09E-06	Endocrine system;Global and overview maps;Lipid metabolism;Lipid metabolism
MSTRG.2606.1	-	ncbi_101798758	FADS2	0.981696113	1.57E-08	Endocrine system;Global and overview maps;Lipid metabolism;Lipid metabolism
MSTRG.2608.1	-	ncbi_101798758	FADS2	0.968801636	2.21E-07	Endocrine system;Global and overview maps;Lipid

						metabolism;Lipid metabolism
MSTRG.2763.2	-	ncbi_101798758	FADS2	0.960736868	6.88E-07	Endocrine system;Global and overview maps;Lipid metabolism;Lipid metabolism
MSTRG.2904.1	-	ncbi_101798758	FADS2	0.984867093	6.09E-09	Endocrine system;Global and overview maps;Lipid metabolism;Lipid metabolism
MSTRG.3080.1	-	ncbi_101798758	FADS2	0.986453302	3.51E-09	Endocrine system;Global and overview maps;Lipid metabolism;Lipid metabolism
MSTRG.3622.7	-	ncbi_101798758	FADS2	0.986104963	3.99E-09	Endocrine system;Global and overview maps;Lipid metabolism;Lipid metabolism
MSTRG.3639.1	-	ncbi_101798758	FADS2	0.987039669	2.82E-09	Endocrine system;Global and overview maps;Lipid metabolism;Lipid metabolism
MSTRG.3832.2	-	ncbi_101798758	FADS2	0.96322273	4.98E-07	Endocrine system;Global and overview maps;Lipid metabolism;Lipid metabolism
MSTRG.4704.1	-	ncbi_101798758	FADS2	0.954021902	1.50E-06	Endocrine system;Global and overview maps;Lipid metabolism;Lipid metabolism
MSTRG.4893.1	-	ncbi_101798758	FADS2	0.97494749	7.45E-08	Endocrine system;Global and overview maps;Lipid metabolism;Lipid metabolism
MSTRG.5011.1	-	ncbi_101798758	FADS2	0.973558467	9.74E-08	Endocrine system;Global and overview maps;Lipid metabolism;Lipid metabolism
MSTRG.5066.1	-	ncbi_101798758	FADS2	-0.963605319	4.73E-07	Endocrine system;Global and overview maps;Lipid metabolism;Lipid metabolism
MSTRG.5135.1	-	ncbi_101798758	FADS2	0.97471522	7.80E-08	Endocrine system;Global and overview maps;Lipid metabolism;Lipid metabolism
MSTRG.5135.3	-	ncbi_101798758	FADS2	0.98346562	9.47E-09	Endocrine system;Global and overview maps;Lipid metabolism;Lipid metabolism
MSTRG.582.1	-	ncbi_101798758	FADS2	0.974040848	8.89E-08	Endocrine system;Global and overview maps;Lipid

						metabolism;Lipid metabolism
MSTRG.582.3	-	ncbi_101798758	FADS2	0.985132252	5.58E-09	Endocrine system;Global and overview maps;Lipid metabolism;Lipid metabolism
MSTRG.582.4	-	ncbi_101798758	FADS2	0.958900567	8.62E-07	Endocrine system;Global and overview maps;Lipid metabolism;Lipid metabolism
MSTRG.5846.1	-	ncbi_101798758	FADS2	0.960101618	7.45E-07	Endocrine system;Global and overview maps;Lipid metabolism;Lipid metabolism
MSTRG.5881.2	-	ncbi_101798758	FADS2	0.980308013	2.26E-08	Endocrine system;Global and overview maps;Lipid metabolism;Lipid metabolism
MSTRG.5997.1	-	ncbi_101798758	FADS2	0.97275828	1.13E-07	Endocrine system;Global and overview maps;Lipid metabolism;Lipid metabolism
MSTRG.6127.1	-	ncbi_101798758	FADS2	0.98421576	7.51E-09	Endocrine system;Global and overview maps;Lipid metabolism;Lipid metabolism
MSTRG.6316.1	-	ncbi_101798758	FADS2	-0.97814925	3.78E-08	Endocrine system;Global and overview maps;Lipid metabolism;Lipid metabolism
MSTRG.6704.2	-	ncbi_101798758	FADS2	0.981315159	1.74E-08	Endocrine system;Global and overview maps;Lipid metabolism;Lipid metabolism
MSTRG.6704.3	-	ncbi_101798758	FADS2	0.988374877	1.64E-09	Endocrine system;Global and overview maps;Lipid metabolism;Lipid metabolism
MSTRG.6704.4	-	ncbi_101798758	FADS2	0.992907574	1.40E-10	Endocrine system;Global and overview maps;Lipid metabolism;Lipid metabolism
MSTRG.715.1	-	ncbi_101798758	FADS2	0.985093816	5.65E-09	Endocrine system;Global and overview maps;Lipid metabolism;Lipid metabolism
MSTRG.7239.1	-	ncbi_101798758	FADS2	0.984987284	5.86E-09	Endocrine system;Global and overview maps;Lipid metabolism;Lipid metabolism
MSTRG.7248.1	-	ncbi_101798758	FADS2	0.987672113	2.20E-09	Endocrine system;Global and overview maps;Lipid

						metabolism;Lipid metabolism
MSTRG.7548.1	-	ncbi_101798758	FADS2	-0.95264616	1.73E-06	Endocrine system;Global and overview maps;Lipid metabolism;Lipid metabolism
MSTRG.756.3	-	ncbi_101798758	FADS2	0.994051868	5.81E-11	Endocrine system;Global and overview maps;Lipid metabolism;Lipid metabolism
MSTRG.7621.5	-	ncbi_101798758	FADS2	0.983469857	9.45E-09	Endocrine system;Global and overview maps;Lipid metabolism;Lipid metabolism
MSTRG.7949.1	-	ncbi_101798758	FADS2	0.982116452	1.40E-08	Endocrine system;Global and overview maps;Lipid metabolism;Lipid metabolism
MSTRG.7954.1	-	ncbi_101798758	FADS2	0.982393092	1.29E-08	Endocrine system;Global and overview maps;Lipid metabolism;Lipid metabolism
MSTRG.8113.1	-	ncbi_101798758	FADS2	0.955883725	1.22E-06	Endocrine system;Global and overview maps;Lipid metabolism;Lipid metabolism
MSTRG.852.1	-	ncbi_101798758	FADS2	0.974315123	8.43E-08	Endocrine system;Global and overview maps;Lipid metabolism;Lipid metabolism
MSTRG.881.1	-	ncbi_101798758	FADS2	0.953998336	1.50E-06	Endocrine system;Global and overview maps;Lipid metabolism;Lipid metabolism
MSTRG.885.1	-	ncbi_101798758	FADS2	0.989146923	1.16E-09	Endocrine system;Global and overview maps;Lipid metabolism;Lipid metabolism
MSTRG.889.1	-	ncbi_101798758	FADS2	0.97025921	1.74E-07	Endocrine system;Global and overview maps;Lipid metabolism;Lipid metabolism
MSTRG.9008.1	-	ncbi_101798758	FADS2	0.971315696	1.46E-07	Endocrine system;Global and overview maps;Lipid metabolism;Lipid metabolism
MSTRG.9025.1	-	ncbi_101798758	FADS2	0.967235293	2.81E-07	Endocrine system;Global and overview maps;Lipid metabolism;Lipid metabolism
MSTRG.9062.2	-	ncbi_101798758	FADS2	0.981219946	1.78E-08	Endocrine system;Global and overview maps;Lipid

						metabolism;Lipid metabolism
XR_001185755.3	LOC106014431	ncbi_101798758	FADS2	0.976904346	4.98E-08	Endocrine system;Global and overview maps;Lipid metabolism;Lipid metabolism
XR_001186359.3	LOC101798301	ncbi_101798758	FADS2	0.963559139	4.76E-07	Endocrine system;Global and overview maps;Lipid metabolism;Lipid metabolism
XR_001186606.3	LOC106014935	ncbi_101798758	FADS2	0.976351136	5.60E-08	Endocrine system;Global and overview maps;Lipid metabolism;Lipid metabolism
XR_001186962.3	LOC106015138	ncbi_101798758	FADS2	0.976811577	5.08E-08	Endocrine system;Global and overview maps;Lipid metabolism;Lipid metabolism
XR_001187494.3	LOC106015449	ncbi_101798758	FADS2	0.968689313	2.25E-07	Endocrine system;Global and overview maps;Lipid metabolism;Lipid metabolism
XR_001187862.3	LOC106015672	ncbi_101798758	FADS2	0.973723038	9.44E-08	Endocrine system;Global and overview maps;Lipid metabolism;Lipid metabolism
XR_001187865.3	LOC106015672	ncbi_101798758	FADS2	0.978663948	3.36E-08	Endocrine system;Global and overview maps;Lipid metabolism;Lipid metabolism
XR_001187947.2	LOC106015729	ncbi_101798758	FADS2	0.974654012	7.90E-08	Endocrine system;Global and overview maps;Lipid metabolism;Lipid metabolism
XR_001187991.3	LOC106015755	ncbi_101798758	FADS2	0.969925937	1.84E-07	Endocrine system;Global and overview maps;Lipid metabolism;Lipid metabolism
XR_001188444.3	LOC106016033	ncbi_101798758	FADS2	0.977084525	4.79E-08	Endocrine system;Global and overview maps;Lipid metabolism;Lipid metabolism
XR_001189244.3	LOC106016485	ncbi_101798758	FADS2	0.976667694	5.24E-08	Endocrine system;Global and overview maps;Lipid metabolism;Lipid metabolism
XR_001189247.3	LOC106016487	ncbi_101798758	FADS2	0.965856631	3.45E-07	Endocrine system;Global and overview maps;Lipid metabolism;Lipid metabolism
XR_001189916.3	LOC106016889	ncbi_101798758	FADS2	0.96328576	4.94E-07	Endocrine system;Global and overview maps;Lipid

						metabolism;Lipid metabolism
XR_001189917.2	LOC106016889	ncbi_101798758	FADS2	0.988975835	1.26E-09	Endocrine system;Global and overview maps;Lipid metabolism;Lipid metabolism
XR_001189941.3	LOC106016901	ncbi_101798758	FADS2	0.978439826	3.54E-08	Endocrine system;Global and overview maps;Lipid metabolism;Lipid metabolism
XR_001190278.3	LOC106017074	ncbi_101798758	FADS2	0.96048195	7.10E-07	Endocrine system;Global and overview maps;Lipid metabolism;Lipid metabolism
XR_001190389.3	LOC106017139	ncbi_101798758	FADS2	0.956661771	1.12E-06	Endocrine system;Global and overview maps;Lipid metabolism;Lipid metabolism
XR_001190631.3	LOC106017268	ncbi_101798758	FADS2	0.970661155	1.63E-07	Endocrine system;Global and overview maps;Lipid metabolism;Lipid metabolism
XR_001190677.3	LOC106017297	ncbi_101798758	FADS2	0.979554542	2.72E-08	Endocrine system;Global and overview maps;Lipid metabolism;Lipid metabolism
XR_001190983.3	LOC106017488	ncbi_101798758	FADS2	0.956409024	1.15E-06	Endocrine system;Global and overview maps;Lipid metabolism;Lipid metabolism
XR_001191855.3	LOC106018027	ncbi_101798758	FADS2	0.96772666	2.61E-07	Endocrine system;Global and overview maps;Lipid metabolism;Lipid metabolism
XR_001193197.3	LOC106018814	ncbi_101798758	FADS2	0.980621187	2.08E-08	Endocrine system;Global and overview maps;Lipid metabolism;Lipid metabolism
XR_001193302.3	LOC106018881	ncbi_101798758	FADS2	0.955541111	1.27E-06	Endocrine system;Global and overview maps;Lipid metabolism;Lipid metabolism
XR_001193335.3	LOC106018911	ncbi_101798758	FADS2	0.9866355	3.28E-09	Endocrine system;Global and overview maps;Lipid metabolism;Lipid metabolism
XR_001193468.3	LOC106018998	ncbi_101798758	FADS2	0.977403736	4.47E-08	Endocrine system;Global and overview maps;Lipid metabolism;Lipid metabolism
XR_001193619.3	LOC106019066	ncbi_101798758	FADS2	0.964770804	4.03E-07	Endocrine system;Global and overview maps;Lipid

						metabolism;Lipid metabolism
XR_001194132.2	LOC106019368	ncbi_101798758	FADS2	0.977894112	4.01E-08	Endocrine system;Global and overview maps;Lipid metabolism;Lipid metabolism
XR_001194441.2	LOC106019549	ncbi_101798758	FADS2	0.974118982	8.76E-08	Endocrine system;Global and overview maps;Lipid metabolism;Lipid metabolism
XR_001194622.3	LOC106019644	ncbi_101798758	FADS2	0.955516025	1.27E-06	Endocrine system;Global and overview maps;Lipid metabolism;Lipid metabolism
XR_002398893.2	LOC110351436	ncbi_101798758	FADS2	0.96197954	5.87E-07	Endocrine system;Global and overview maps;Lipid metabolism;Lipid metabolism
XR_002399173.2	LOC110351568	ncbi_101798758	FADS2	0.979823042	2.55E-08	Endocrine system;Global and overview maps;Lipid metabolism;Lipid metabolism
XR_002399314.2	LOC106015131	ncbi_101798758	FADS2	0.952998472	1.67E-06	Endocrine system;Global and overview maps;Lipid metabolism;Lipid metabolism
XR_002399432.2	LOC110351688	ncbi_101798758	FADS2	0.951713466	1.91E-06	Endocrine system;Global and overview maps;Lipid metabolism;Lipid metabolism
XR_002399732.1	LOC110351814	ncbi_101798758	FADS2	0.984441434	7.00E-09	Endocrine system;Global and overview maps;Lipid metabolism;Lipid metabolism
XR_002399836.2	LOC106015519	ncbi_101798758	FADS2	0.971392805	1.44E-07	Endocrine system;Global and overview maps;Lipid metabolism;Lipid metabolism
XR_002399884.2	LOC110351873	ncbi_101798758	FADS2	0.972704838	1.14E-07	Endocrine system;Global and overview maps;Lipid metabolism;Lipid metabolism
XR_002399898.2	LOC110351880	ncbi_101798758	FADS2	0.957224673	1.05E-06	Endocrine system;Global and overview maps;Lipid metabolism;Lipid metabolism
XR_002400226.2	LOC106015733	ncbi_101798758	FADS2	0.960114146	7.43E-07	Endocrine system;Global and overview maps;Lipid metabolism;Lipid metabolism
XR_002400249.2	LOC110351990	ncbi_101798758	FADS2	0.964398099	4.24E-07	Endocrine system;Global and overview maps;Lipid

						metabolism;Lipid metabolism
XR_002400594.2	LOC110352134	ncbi_101798758	FADS2	0.978312364	3.64E-08	Endocrine system;Global and overview maps;Lipid metabolism;Lipid metabolism
XR_002400639.2	LOC110352150	ncbi_101798758	FADS2	0.957226178	1.05E-06	Endocrine system;Global and overview maps;Lipid metabolism;Lipid metabolism
XR_002400646.2	LOC110352151	ncbi_101798758	FADS2	0.970157283	1.77E-07	Endocrine system;Global and overview maps;Lipid metabolism;Lipid metabolism
XR_002400673.2	LOC106016078	ncbi_101798758	FADS2	0.95107817	2.03E-06	Endocrine system;Global and overview maps;Lipid metabolism;Lipid metabolism
XR_002400870.1	LOC110352222	ncbi_101798758	FADS2	0.963444156	4.83E-07	Endocrine system;Global and overview maps;Lipid metabolism;Lipid metabolism
XR_002400941.2	LOC106016276	ncbi_101798758	FADS2	0.976540924	5.38E-08	Endocrine system;Global and overview maps;Lipid metabolism;Lipid metabolism
XR_002401026.2	LOC110352298	ncbi_101798758	FADS2	0.976836546	5.05E-08	Endocrine system;Global and overview maps;Lipid metabolism;Lipid metabolism
XR_002401202.2	LOC110352351	ncbi_101798758	FADS2	0.967965178	2.52E-07	Endocrine system;Global and overview maps;Lipid metabolism;Lipid metabolism
XR_002401479.2	LOC106016692	ncbi_101798758	FADS2	0.978121162	3.81E-08	Endocrine system;Global and overview maps;Lipid metabolism;Lipid metabolism
XR_002401846.2	LOC106016921	ncbi_101798758	FADS2	0.954042267	1.49E-06	Endocrine system;Global and overview maps;Lipid metabolism;Lipid metabolism
XR_002401866.2	LOC106016894	ncbi_101798758	FADS2	0.979518306	2.74E-08	Endocrine system;Global and overview maps;Lipid metabolism;Lipid metabolism
XR_002401875.2	LOC110352627	ncbi_101798758	FADS2	0.974660795	7.88E-08	Endocrine system;Global and overview maps;Lipid metabolism;Lipid metabolism
XR_002402176.2	LOC110352712	ncbi_101798758	FADS2	0.977752196	4.14E-08	Endocrine system;Global and overview maps;Lipid

						metabolism;Lipid metabolism
XR_002402646.2	LOC101794014	ncbi_101798758	FADS2	0.978617359	3.40E-08	Endocrine system;Global and overview maps;Lipid metabolism;Lipid metabolism
XR_002402718.2	LOC106017475	ncbi_101798758	FADS2	0.982423352	1.28E-08	Endocrine system;Global and overview maps;Lipid metabolism;Lipid metabolism
XR_002402719.2	LOC106017476	ncbi_101798758	FADS2	0.977617984	4.26E-08	Endocrine system;Global and overview maps;Lipid metabolism;Lipid metabolism
XR_002403184.2	LOC106017746	ncbi_101798758	FADS2	0.977221422	4.65E-08	Endocrine system;Global and overview maps;Lipid metabolism;Lipid metabolism
XR_002404244.2	LOC106018581	ncbi_101798758	FADS2	0.95446499	1.43E-06	Endocrine system;Global and overview maps;Lipid metabolism;Lipid metabolism
XR_002404947.2	LOC110353846	ncbi_101798758	FADS2	0.980737702	2.02E-08	Endocrine system;Global and overview maps;Lipid metabolism;Lipid metabolism
XR_002405449.2	LOC110354050	ncbi_101798758	FADS2	0.964034081	4.46E-07	Endocrine system;Global and overview maps;Lipid metabolism;Lipid metabolism
XR_002405521.2	LOC106019445	ncbi_101798758	FADS2	0.962646551	5.38E-07	Endocrine system;Global and overview maps;Lipid metabolism;Lipid metabolism
XR_002405535.2	LOC110354079	ncbi_101798758	FADS2	0.978913851	3.17E-08	Endocrine system;Global and overview maps;Lipid metabolism;Lipid metabolism
XR_002405612.2	LOC106019471	ncbi_101798758	FADS2	0.956064408	1.20E-06	Endocrine system;Global and overview maps;Lipid metabolism;Lipid metabolism
XR_002405843.2	LOC106019674	ncbi_101798758	FADS2	0.977252924	4.62E-08	Endocrine system;Global and overview maps;Lipid metabolism;Lipid metabolism
XR_002405947.2	LOC106019753	ncbi_101798758	FADS2	0.981291645	1.75E-08	Endocrine system;Global and overview maps;Lipid metabolism;Lipid metabolism
XR_002406139.2	LOC110354333	ncbi_101798758	FADS2	0.96973383	1.90E-07	Endocrine system;Global and overview maps;Lipid

						metabolism;Lipid metabolism
XR_002406465.2	LOC106020114	ncbi_101798758	FADS2	0.969581844	1.95E-07	Endocrine system;Global and overview maps;Lipid metabolism;Lipid metabolism
XR_002406489.2	LOC106020129	ncbi_101798758	FADS2	0.967190452	2.83E-07	Endocrine system;Global and overview maps;Lipid metabolism;Lipid metabolism
XR_002406729.2	LOC106020349	ncbi_101798758	FADS2	0.989043184	1.22E-09	Endocrine system;Global and overview maps;Lipid metabolism;Lipid metabolism
XR_002406750.2	LOC106020393	ncbi_101798758	FADS2	0.98399619	8.05E-09	Endocrine system;Global and overview maps;Lipid metabolism;Lipid metabolism
XR_003492182.1	LOC113839646	ncbi_101798758	FADS2	0.955144445	1.33E-06	Endocrine system;Global and overview maps;Lipid metabolism;Lipid metabolism
XR_003492230.1	LOC113839672	ncbi_101798758	FADS2	0.981558654	1.63E-08	Endocrine system;Global and overview maps;Lipid metabolism;Lipid metabolism
XR_003492236.1	LOC113839675	ncbi_101798758	FADS2	0.959493006	8.02E-07	Endocrine system;Global and overview maps;Lipid metabolism;Lipid metabolism
XR_003492300.1	LOC110354130	ncbi_101798758	FADS2	0.964760872	4.03E-07	Endocrine system;Global and overview maps;Lipid metabolism;Lipid metabolism
XR_003492318.1	LOC113839721	ncbi_101798758	FADS2	0.969792057	1.88E-07	Endocrine system;Global and overview maps;Lipid metabolism;Lipid metabolism
XR_003492832.1	LOC113840106	ncbi_101798758	FADS2	0.977847215	4.05E-08	Endocrine system;Global and overview maps;Lipid metabolism;Lipid metabolism
XR_003493103.1	LOC113840309	ncbi_101798758	FADS2	0.98193633	1.47E-08	Endocrine system;Global and overview maps;Lipid metabolism;Lipid metabolism
XR_003493806.1	LOC113841174	ncbi_101798758	FADS2	0.983580994	9.14E-09	Endocrine system;Global and overview maps;Lipid metabolism;Lipid metabolism
XR_003494318.1	LOC113841955	ncbi_101798758	FADS2	0.965880056	3.44E-07	Endocrine system;Global and overview maps;Lipid

						metabolism;Lipid metabolism
XR_003494664.1	LOC113842319	ncbi_101798758	FADS2	0.987160962	2.69E-09	Endocrine system;Global and overview maps;Lipid metabolism;Lipid metabolism
XR_003494775.1	LOC110352361	ncbi_101798758	FADS2	0.976904346	4.98E-08	Endocrine system;Global and overview maps;Lipid metabolism;Lipid metabolism
XR_003494880.1	LOC106020483	ncbi_101798758	FADS2	0.95905409	8.46E-07	Endocrine system;Global and overview maps;Lipid metabolism;Lipid metabolism
XR_003494883.1	LOC106020483	ncbi_101798758	FADS2	0.981245004	1.77E-08	Endocrine system;Global and overview maps;Lipid metabolism;Lipid metabolism
XR_003494887.1	LOC113842395	ncbi_101798758	FADS2	0.975276339	6.98E-08	Endocrine system;Global and overview maps;Lipid metabolism;Lipid metabolism
XR_003494888.1	LOC113842396	ncbi_101798758	FADS2	0.985067764	5.70E-09	Endocrine system;Global and overview maps;Lipid metabolism;Lipid metabolism
XR_003494919.1	LOC113842414	ncbi_101798758	FADS2	-0.968921069	2.17E-07	Endocrine system;Global and overview maps;Lipid metabolism;Lipid metabolism
XR_003494957.1	LOC101799909	ncbi_101798758	FADS2	0.982751083	1.17E-08	Endocrine system;Global and overview maps;Lipid metabolism;Lipid metabolism
XR_003494960.1	LOC101799909	ncbi_101798758	FADS2	0.974656623	7.89E-08	Endocrine system;Global and overview maps;Lipid metabolism;Lipid metabolism
XR_003494961.1	LOC113842447	ncbi_101798758	FADS2	0.988190252	1.77E-09	Endocrine system;Global and overview maps;Lipid metabolism;Lipid metabolism
XR_003495207.1	LOC113842649	ncbi_101798758	FADS2	0.967847152	2.56E-07	Endocrine system;Global and overview maps;Lipid metabolism;Lipid metabolism
XR_003495345.1	LOC101793386	ncbi_101798758	FADS2	0.985824569	4.40E-09	Endocrine system;Global and overview maps;Lipid metabolism;Lipid metabolism
XR_003495483.1	LOC113842770	ncbi_101798758	FADS2	0.982634657	1.21E-08	Endocrine system;Global and overview maps;Lipid

						metabolism;Lipid metabolism
XR_003495635.1	LOC113842831	ncbi_101798758	FADS2	0.962906984	5.20E-07	Endocrine system;Global and overview maps;Lipid metabolism;Lipid metabolism
XR_003495767.1	LOC110352593	ncbi_101798758	FADS2	0.969372131	2.02E-07	Endocrine system;Global and overview maps;Lipid metabolism;Lipid metabolism
XR_003495918.1	LOC113843035	ncbi_101798758	FADS2	0.969041336	2.13E-07	Endocrine system;Global and overview maps;Lipid metabolism;Lipid metabolism
XR_003495930.1	LOC113839603	ncbi_101798758	FADS2	0.975589704	6.55E-08	Endocrine system;Global and overview maps;Lipid metabolism;Lipid metabolism
XR_003496139.1	LOC113843116	ncbi_101798758	FADS2	0.967639087	2.65E-07	Endocrine system;Global and overview maps;Lipid metabolism;Lipid metabolism
XR_003496222.1	LOC101792916	ncbi_101798758	FADS2	0.96600445	3.38E-07	Endocrine system;Global and overview maps;Lipid metabolism;Lipid metabolism
XR_003496334.1	LOC113843176	ncbi_101798758	FADS2	0.99168585	3.09E-10	Endocrine system;Global and overview maps;Lipid metabolism;Lipid metabolism
XR_003496440.1	LOC106015283	ncbi_101798758	FADS2	0.980356362	2.23E-08	Endocrine system;Global and overview maps;Lipid metabolism;Lipid metabolism
XR_003496447.1	LOC113843228	ncbi_101798758	FADS2	0.982252694	1.35E-08	Endocrine system;Global and overview maps;Lipid metabolism;Lipid metabolism
XR_003496885.1	LOC113843526	ncbi_101798758	FADS2	0.964910766	3.95E-07	Endocrine system;Global and overview maps;Lipid metabolism;Lipid metabolism
XR_003496983.1	LOC113843553	ncbi_101798758	FADS2	0.953842963	1.53E-06	Endocrine system;Global and overview maps;Lipid metabolism;Lipid metabolism
XR_003497001.1	LOC106017547	ncbi_101798758	FADS2	0.988655326	1.45E-09	Endocrine system;Global and overview maps;Lipid metabolism;Lipid metabolism
XR_003497002.1	LOC106017547	ncbi_101798758	FADS2	0.959769434	7.76E-07	Endocrine system;Global and overview maps;Lipid

						metabolism;Lipid metabolism
XR_003497032.1	LOC106020477	ncbi_101798758	FADS2	0.956460434	1.15E-06	Endocrine system;Global and overview maps;Lipid metabolism;Lipid metabolism
XR_003497296.1	LOC113843645	ncbi_101798758	FADS2	0.984730225	6.37E-09	Endocrine system;Global and overview maps;Lipid metabolism;Lipid metabolism
XR_003497330.1	LOC110352095	ncbi_101798758	FADS2	-0.95056309	2.14E-06	Endocrine system;Global and overview maps;Lipid metabolism;Lipid metabolism
XR_003497481.1	LOC110351804	ncbi_101798758	FADS2	0.973145495	1.05E-07	Endocrine system;Global and overview maps;Lipid metabolism;Lipid metabolism
XR_003497511.1	LOC110353153	ncbi_101798758	FADS2	0.954500275	1.42E-06	Endocrine system;Global and overview maps;Lipid metabolism;Lipid metabolism
XR_003497529.1	LOC113843776	ncbi_101798758	FADS2	0.989393185	1.04E-09	Endocrine system;Global and overview maps;Lipid metabolism;Lipid metabolism
XR_003497551.1	LOC110354397	ncbi_101798758	FADS2	0.965253011	3.76E-07	Endocrine system;Global and overview maps;Lipid metabolism;Lipid metabolism
XR_003497554.1	LOC110354397	ncbi_101798758	FADS2	0.950980168	2.05E-06	Endocrine system;Global and overview maps;Lipid metabolism;Lipid metabolism
XR_003497564.1	LOC101802970	ncbi_101798758	FADS2	0.974561132	8.04E-08	Endocrine system;Global and overview maps;Lipid metabolism;Lipid metabolism
XR_003497566.1	LOC101802970	ncbi_101798758	FADS2	0.991121269	4.28E-10	Endocrine system;Global and overview maps;Lipid metabolism;Lipid metabolism
XR_003497568.1	LOC101802970	ncbi_101798758	FADS2	0.957395533	1.03E-06	Endocrine system;Global and overview maps;Lipid metabolism;Lipid metabolism
XR_003497661.1	LOC101798800	ncbi_101798758	FADS2	0.967370991	2.76E-07	Endocrine system;Global and overview maps;Lipid metabolism;Lipid metabolism
XR_003497665.1	LOC110351913	ncbi_101798758	FADS2	0.974558693	8.04E-08	Endocrine system;Global and overview maps;Lipid

						metabolism;Lipid metabolism
XR_003497745.1	LOC110353307	ncbi_101798758	FADS2	0.964298475	4.30E-07	Endocrine system;Global and overview maps;Lipid metabolism;Lipid metabolism
XR_003497899.1	LOC110353379	ncbi_101798758	FADS2	0.976505552	5.42E-08	Endocrine system;Global and overview maps;Lipid metabolism;Lipid metabolism
XR_003497951.1	LOC113843960	ncbi_101798758	FADS2	0.978937021	3.15E-08	Endocrine system;Global and overview maps;Lipid metabolism;Lipid metabolism
XR_003498021.1	LOC101803684	ncbi_101798758	FADS2	0.967008829	2.91E-07	Endocrine system;Global and overview maps;Lipid metabolism;Lipid metabolism
XR_003498030.1	LOC101803684	ncbi_101798758	FADS2	0.958982933	8.53E-07	Endocrine system;Global and overview maps;Lipid metabolism;Lipid metabolism
XR_003498061.1	LOC106015393	ncbi_101798758	FADS2	0.962412264	5.55E-07	Endocrine system;Global and overview maps;Lipid metabolism;Lipid metabolism
XR_003498141.1	LOC113844039	ncbi_101798758	FADS2	0.959240714	8.27E-07	Endocrine system;Global and overview maps;Lipid metabolism;Lipid metabolism
XR_003498397.1	LOC101797653	ncbi_101798758	FADS2	0.951102948	2.03E-06	Endocrine system;Global and overview maps;Lipid metabolism;Lipid metabolism
XR_003498491.1	LOC113844169	ncbi_101798758	FADS2	0.993740183	7.49E-11	Endocrine system;Global and overview maps;Lipid metabolism;Lipid metabolism
XR_003498621.1	LOC113844221	ncbi_101798758	FADS2	0.953178379	1.64E-06	Endocrine system;Global and overview maps;Lipid metabolism;Lipid metabolism
XR_003498691.1	LOC106019447	ncbi_101798758	FADS2	0.963028501	5.11E-07	Endocrine system;Global and overview maps;Lipid metabolism;Lipid metabolism
XR_003498693.1	LOC110354267	ncbi_101798758	FADS2	0.977639038	4.24E-08	Endocrine system;Global and overview maps;Lipid metabolism;Lipid metabolism
XR_003498805.1	LOC106019442	ncbi_101798758	FADS2	0.973894484	9.14E-08	Endocrine system;Global and overview maps;Lipid

						metabolism;Lipid metabolism
XR_003498864.1	LOC113844340	ncbi_101798758	FADS2	0.980173052	2.33E-08	Endocrine system;Global and overview maps;Lipid metabolism;Lipid metabolism
XR_003498910.1	LOC106017955	ncbi_101798758	FADS2	0.962522371	5.47E-07	Endocrine system;Global and overview maps;Lipid metabolism;Lipid metabolism
XR_003499063.1	LOC110353608	ncbi_101798758	FADS2	0.990645166	5.55E-10	Endocrine system;Global and overview maps;Lipid metabolism;Lipid metabolism
XR_003499720.1	LOC113844791	ncbi_101798758	FADS2	0.98017485	2.33E-08	Endocrine system;Global and overview maps;Lipid metabolism;Lipid metabolism
XR_003499753.1	LOC113844812	ncbi_101798758	FADS2	0.972901601	1.10E-07	Endocrine system;Global and overview maps;Lipid metabolism;Lipid metabolism
XR_003499790.1	LOC101804048	ncbi_101798758	FADS2	0.976269386	5.70E-08	Endocrine system;Global and overview maps;Lipid metabolism;Lipid metabolism
XR_003499827.1	LOC106019660	ncbi_101798758	FADS2	0.961974595	5.87E-07	Endocrine system;Global and overview maps;Lipid metabolism;Lipid metabolism
XR_003499961.1	LOC110352806	ncbi_101798758	FADS2	0.958303082	9.25E-07	Endocrine system;Global and overview maps;Lipid metabolism;Lipid metabolism
XR_003499966.1	LOC110352806	ncbi_101798758	FADS2	0.971688195	1.37E-07	Endocrine system;Global and overview maps;Lipid metabolism;Lipid metabolism
XR_003500183.1	LOC106020052	ncbi_101798758	FADS2	0.962138725	5.75E-07	Endocrine system;Global and overview maps;Lipid metabolism;Lipid metabolism
XR_003500301.1	LOC113845068	ncbi_101798758	FADS2	0.981442269	1.68E-08	Endocrine system;Global and overview maps;Lipid metabolism;Lipid metabolism
XR_003500366.1	LOC110351294	ncbi_101798758	FADS2	0.976388561	5.56E-08	Endocrine system;Global and overview maps;Lipid metabolism;Lipid metabolism
XR_003500566.1	LOC113845180	ncbi_101798758	FADS2	0.961577923	6.18E-07	Endocrine system;Global and overview maps;Lipid

						metabolism;Lipid metabolism
XR_003500671.1	LOC106019837	ncbi_101798758	FADS2	0.972669798	1.15E-07	Endocrine system;Global and overview maps;Lipid metabolism;Lipid metabolism
XR_003500722.1	LOC113845266	ncbi_101798758	FADS2	0.96779174	2.59E-07	Endocrine system;Global and overview maps;Lipid metabolism;Lipid metabolism
XR_003500751.1	LOC113845280	ncbi_101798758	FADS2	0.966026995	3.37E-07	Endocrine system;Global and overview maps;Lipid metabolism;Lipid metabolism
XR_003500934.1	LOC113845362	ncbi_101798758	FADS2	0.953988761	1.50E-06	Endocrine system;Global and overview maps;Lipid metabolism;Lipid metabolism
XR_003501281.1	LOC106015373	ncbi_101798758	FADS2	0.972351589	1.21E-07	Endocrine system;Global and overview maps;Lipid metabolism;Lipid metabolism
XR_003501403.1	LOC113845679	ncbi_101798758	FADS2	0.961639788	6.13E-07	Endocrine system;Global and overview maps;Lipid metabolism;Lipid metabolism
XR_003501474.1	LOC106019132	ncbi_101798758	FADS2	0.972558567	1.17E-07	Endocrine system;Global and overview maps;Lipid metabolism;Lipid metabolism
XR_217454.4	LOC101799933	ncbi_101798758	FADS2	0.972481079	1.19E-07	Endocrine system;Global and overview maps;Lipid metabolism;Lipid metabolism
MSTRG.10098.1	-	ncbi_101799007	FADS1	0.986140348	3.94E-09	Global and overview maps;Lipid metabolism
MSTRG.10100.1	-	ncbi_101799007	FADS1	0.990631939	5.59E-10	Global and overview maps;Lipid metabolism
MSTRG.10341.18	-	ncbi_101799007	FADS1	0.98012984	2.36E-08	Global and overview maps;Lipid metabolism
MSTRG.10341.19	-	ncbi_101799007	FADS1	0.995941619	8.61E-12	Global and overview maps;Lipid metabolism
MSTRG.10341.20	-	ncbi_101799007	FADS1	0.995083922	2.24E-11	Global and overview maps;Lipid metabolism
MSTRG.10341.21	-	ncbi_101799007	FADS1	0.994208899	5.08E-11	Global and overview maps;Lipid metabolism
MSTRG.10341.22	-	ncbi_101799007	FADS1	0.996396241	4.76E-12	Global and overview maps;Lipid metabolism
MSTRG.12747.4	-	ncbi_101799007	FADS1	0.982550746	1.24E-08	Global and overview maps;Lipid metabolism
MSTRG.12949.1	-	ncbi_101799007	FADS1	0.971398726	1.44E-07	Global and overview maps;Lipid metabolism

MSTRG.13250.1	-	ncbi_101799007	FADS1	0.961513965	6.23E-07	Global and overview maps;Lipid metabolism
MSTRG.13538.3	-	ncbi_101799007	FADS1	0.975791419	6.29E-08	Global and overview maps;Lipid metabolism
MSTRG.13544.2	-	ncbi_101799007	FADS1	0.996032542	7.69E-12	Global and overview maps;Lipid metabolism
MSTRG.13915.1	-	ncbi_101799007	FADS1	0.98057396	2.11E-08	Global and overview maps;Lipid metabolism
MSTRG.14551.1	-	ncbi_101799007	FADS1	-0.963169041	5.02E-07	Global and overview maps;Lipid metabolism
MSTRG.15964.1	-	ncbi_101799007	FADS1	0.959709549	7.81E-07	Global and overview maps;Lipid metabolism
MSTRG.15965.1	-	ncbi_101799007	FADS1	0.986984434	2.88E-09	Global and overview maps;Lipid metabolism
MSTRG.16028.1	-	ncbi_101799007	FADS1	0.962481873	5.50E-07	Global and overview maps;Lipid metabolism
MSTRG.16118.5	-	ncbi_101799007	FADS1	0.963190552	5.00E-07	Global and overview maps;Lipid metabolism
MSTRG.16827.2	-	ncbi_101799007	FADS1	0.959511584	8.01E-07	Global and overview maps;Lipid metabolism
MSTRG.16889.1	-	ncbi_101799007	FADS1	0.984545335	6.77E-09	Global and overview maps;Lipid metabolism
MSTRG.1816.1	-	ncbi_101799007	FADS1	0.968827126	2.20E-07	Global and overview maps;Lipid metabolism
MSTRG.2348.4	-	ncbi_101799007	FADS1	0.960287887	7.28E-07	Global and overview maps;Lipid metabolism
MSTRG.2606.1	-	ncbi_101799007	FADS1	0.990565785	5.79E-10	Global and overview maps;Lipid metabolism
MSTRG.2608.1	-	ncbi_101799007	FADS1	0.975360388	6.86E-08	Global and overview maps;Lipid metabolism
MSTRG.2763.2	-	ncbi_101799007	FADS1	0.974243804	8.55E-08	Global and overview maps;Lipid metabolism
MSTRG.2904.1	-	ncbi_101799007	FADS1	0.990155385	7.16E-10	Global and overview maps;Lipid metabolism
MSTRG.3080.1	-	ncbi_101799007	FADS1	0.992085294	2.41E-10	Global and overview maps;Lipid metabolism
MSTRG.3201.1	-	ncbi_101799007	FADS1	0.9523947	1.78E-06	Global and overview maps;Lipid metabolism
MSTRG.3622.7	-	ncbi_101799007	FADS1	0.989992554	7.77E-10	Global and overview maps;Lipid metabolism
MSTRG.3639.1	-	ncbi_101799007	FADS1	0.976301972	5.66E-08	Global and overview maps;Lipid metabolism
MSTRG.3832.2	-	ncbi_101799007	FADS1	0.969510779	1.97E-07	Global and overview maps;Lipid metabolism
MSTRG.4704.1	-	ncbi_101799007	FADS1	0.971593228	1.39E-07	Global and overview maps;Lipid metabolism
MSTRG.4893.1	-	ncbi_101799007	FADS1	0.980416776	2.20E-08	Global and overview maps;Lipid metabolism

MSTRG.5011.1	-	ncbi_101799007	FADS1	0.983704368	8.81E-09	Global and overview maps;Lipid metabolism
MSTRG.5066.1	-	ncbi_101799007	FADS1	-0.972179136	1.25E-07	Global and overview maps;Lipid metabolism
MSTRG.5135.1	-	ncbi_101799007	FADS1	0.990456417	6.14E-10	Global and overview maps;Lipid metabolism
MSTRG.5135.3	-	ncbi_101799007	FADS1	0.996228572	5.97E-12	Global and overview maps;Lipid metabolism
MSTRG.5601.1	-	ncbi_101799007	FADS1	0.954200361	1.47E-06	Global and overview maps;Lipid metabolism
MSTRG.582.1	-	ncbi_101799007	FADS1	0.975145729	7.16E-08	Global and overview maps;Lipid metabolism
MSTRG.582.3	-	ncbi_101799007	FADS1	0.984260046	7.41E-09	Global and overview maps;Lipid metabolism
MSTRG.582.4	-	ncbi_101799007	FADS1	0.972780012	1.12E-07	Global and overview maps;Lipid metabolism
MSTRG.5846.1	-	ncbi_101799007	FADS1	0.977111936	4.76E-08	Global and overview maps;Lipid metabolism
MSTRG.5881.2	-	ncbi_101799007	FADS1	0.989170872	1.15E-09	Global and overview maps;Lipid metabolism
MSTRG.5997.1	-	ncbi_101799007	FADS1	0.982992611	1.09E-08	Global and overview maps;Lipid metabolism
MSTRG.6017.1	-	ncbi_101799007	FADS1	0.961837913	5.98E-07	Global and overview maps;Lipid metabolism
MSTRG.6127.1	-	ncbi_101799007	FADS1	0.989480622	9.97E-10	Global and overview maps;Lipid metabolism
MSTRG.6316.1	-	ncbi_101799007	FADS1	-0.990965712	4.67E-10	Global and overview maps;Lipid metabolism
MSTRG.6704.2	-	ncbi_101799007	FADS1	0.985104404	5.63E-09	Global and overview maps;Lipid metabolism
MSTRG.6704.3	-	ncbi_101799007	FADS1	0.990983156	4.62E-10	Global and overview maps;Lipid metabolism
MSTRG.6704.4	-	ncbi_101799007	FADS1	0.996881827	2.31E-12	Global and overview maps;Lipid metabolism
MSTRG.715.1	-	ncbi_101799007	FADS1	0.989097797	1.19E-09	Global and overview maps;Lipid metabolism
MSTRG.7239.1	-	ncbi_101799007	FADS1	0.988526998	1.54E-09	Global and overview maps;Lipid metabolism
MSTRG.7248.1	-	ncbi_101799007	FADS1	0.991482699	3.48E-10	Global and overview maps;Lipid metabolism
MSTRG.756.3	-	ncbi_101799007	FADS1	0.995682807	1.17E-11	Global and overview maps;Lipid metabolism
MSTRG.7621.5	-	ncbi_101799007	FADS1	0.986431624	3.54E-09	Global and overview maps;Lipid metabolism
MSTRG.7949.1	-	ncbi_101799007	FADS1	0.982841523	1.14E-08	Global and overview maps;Lipid metabolism

MSTRG.7954.1	-	ncbi_101799007	FADS1	0.986113128	3.97E-09	Global and overview maps;Lipid metabolism
MSTRG.8113.1	-	ncbi_101799007	FADS1	0.9658266	3.47E-07	Global and overview maps;Lipid metabolism
MSTRG.8113.3	-	ncbi_101799007	FADS1	0.950868589	2.08E-06	Global and overview maps;Lipid metabolism
MSTRG.852.1	-	ncbi_101799007	FADS1	0.97821321	3.73E-08	Global and overview maps;Lipid metabolism
MSTRG.8538.1	-	ncbi_101799007	FADS1	0.951359427	1.98E-06	Global and overview maps;Lipid metabolism
MSTRG.885.1	-	ncbi_101799007	FADS1	0.980250783	2.29E-08	Global and overview maps;Lipid metabolism
MSTRG.889.1	-	ncbi_101799007	FADS1	0.958866215	8.65E-07	Global and overview maps;Lipid metabolism
MSTRG.9008.1	-	ncbi_101799007	FADS1	0.974495084	8.14E-08	Global and overview maps;Lipid metabolism
MSTRG.9062.2	-	ncbi_101799007	FADS1	0.987149793	2.70E-09	Global and overview maps;Lipid metabolism
XR_001185755.3	LOC106014431	ncbi_101799007	FADS1	0.98130559	1.74E-08	Global and overview maps;Lipid metabolism
XR_001186359.3	LOC101798301	ncbi_101799007	FADS1	0.962000729	5.85E-07	Global and overview maps;Lipid metabolism
XR_001186606.3	LOC106014935	ncbi_101799007	FADS1	0.980060277	2.40E-08	Global and overview maps;Lipid metabolism
XR_001186962.3	LOC106015138	ncbi_101799007	FADS1	0.980536203	2.13E-08	Global and overview maps;Lipid metabolism
XR_001187494.3	LOC106015449	ncbi_101799007	FADS1	0.970519298	1.67E-07	Global and overview maps;Lipid metabolism
XR_001187862.3	LOC106015672	ncbi_101799007	FADS1	0.980480497	2.16E-08	Global and overview maps;Lipid metabolism
XR_001187865.3	LOC106015672	ncbi_101799007	FADS1	0.979288202	2.90E-08	Global and overview maps;Lipid metabolism
XR_001187947.2	LOC106015729	ncbi_101799007	FADS1	0.981763103	1.54E-08	Global and overview maps;Lipid metabolism
XR_001187991.3	LOC106015755	ncbi_101799007	FADS1	0.973098293	1.06E-07	Global and overview maps;Lipid metabolism
XR_001188444.3	LOC106016033	ncbi_101799007	FADS1	0.986175927	3.89E-09	Global and overview maps;Lipid metabolism
XR_001189244.3	LOC106016485	ncbi_101799007	FADS1	0.974787904	7.69E-08	Global and overview maps;Lipid metabolism
XR_001189247.3	LOC106016487	ncbi_101799007	FADS1	0.962092088	5.78E-07	Global and overview maps;Lipid metabolism
XR_001189916.3	LOC106016889	ncbi_101799007	FADS1	0.966068513	3.35E-07	Global and overview maps;Lipid metabolism
XR_001189917.2	LOC106016889	ncbi_101799007	FADS1	0.990061859	7.51E-10	Global and overview maps;Lipid metabolism
XR_001189941.3	LOC106016901	ncbi_101799007	FADS1	0.981966507	1.46E-08	Global and overview maps;Lipid metabolism
XR_001190278.3	LOC106017074	ncbi_101799007	FADS1	0.957219218	1.05E-06	Global and overview maps;Lipid metabolism

XR_001190389.3	LOC106017139	ncbi_101799007	FADS1	0.953004922	1.67E-06	Global and overview maps;Lipid metabolism
XR_001190631.3	LOC106017268	ncbi_101799007	FADS1	0.967191499	2.83E-07	Global and overview maps;Lipid metabolism
XR_001190677.3	LOC106017297	ncbi_101799007	FADS1	0.9835817	9.14E-09	Global and overview maps;Lipid metabolism
XR_001190983.3	LOC106017488	ncbi_101799007	FADS1	0.960300076	7.27E-07	Global and overview maps;Lipid metabolism
XR_001191423.3	LOC106017763	ncbi_101799007	FADS1	0.960225632	7.33E-07	Global and overview maps;Lipid metabolism
XR_001191855.3	LOC106018027	ncbi_101799007	FADS1	0.969413993	2.00E-07	Global and overview maps;Lipid metabolism
XR_001191910.3	LOC106018057	ncbi_101799007	FADS1	0.951512589	1.95E-06	Global and overview maps;Lipid metabolism
XR_001192311.2	LOC106018298	ncbi_101799007	FADS1	0.959783217	7.74E-07	Global and overview maps;Lipid metabolism
XR_001193197.3	LOC106018814	ncbi_101799007	FADS1	0.989092747	1.19E-09	Global and overview maps;Lipid metabolism
XR_001193335.3	LOC106018911	ncbi_101799007	FADS1	0.987079944	2.77E-09	Global and overview maps;Lipid metabolism
XR_001193468.3	LOC106018998	ncbi_101799007	FADS1	0.98110739	1.84E-08	Global and overview maps;Lipid metabolism
XR_001193619.3	LOC106019066	ncbi_101799007	FADS1	0.973314419	1.02E-07	Global and overview maps;Lipid metabolism
XR_001194132.2	LOC106019368	ncbi_101799007	FADS1	0.980426269	2.19E-08	Global and overview maps;Lipid metabolism
XR_001194441.2	LOC106019549	ncbi_101799007	FADS1	0.972407636	1.20E-07	Global and overview maps;Lipid metabolism
XR_001194622.3	LOC106019644	ncbi_101799007	FADS1	0.961696236	6.09E-07	Global and overview maps;Lipid metabolism
XR_001195514.2	LOC106020129	ncbi_101799007	FADS1	0.952788006	1.71E-06	Global and overview maps;Lipid metabolism
XR_002398893.2	LOC110351436	ncbi_101799007	FADS1	0.963704703	4.67E-07	Global and overview maps;Lipid metabolism
XR_002399173.2	LOC110351568	ncbi_101799007	FADS1	0.980989346	1.89E-08	Global and overview maps;Lipid metabolism
XR_002399314.2	LOC106015131	ncbi_101799007	FADS1	0.953754006	1.54E-06	Global and overview maps;Lipid metabolism
XR_002399432.2	LOC110351688	ncbi_101799007	FADS1	0.953200987	1.63E-06	Global and overview maps;Lipid metabolism
XR_002399732.1	LOC110351814	ncbi_101799007	FADS1	0.986181948	3.88E-09	Global and overview maps;Lipid metabolism
XR_002399836.2	LOC106015519	ncbi_101799007	FADS1	0.984601323	6.64E-09	Global and overview maps;Lipid metabolism
XR_002399884.2	LOC110351873	ncbi_101799007	FADS1	0.974938943	7.46E-08	Global and overview maps;Lipid metabolism
XR_002399898.2	LOC110351880	ncbi_101799007	FADS1	0.955559949	1.27E-06	Global and overview maps;Lipid metabolism
XR_002400226.2	LOC106015733	ncbi_101799007	FADS1	0.95801872	9.57E-07	Global and overview maps;Lipid metabolism

XR_002400249.2	LOC110351990	ncbi_101799007	FADS1	0.974207551	8.61E-08	Global and overview maps;Lipid metabolism
XR_002400594.2	LOC110352134	ncbi_101799007	FADS1	0.981149869	1.82E-08	Global and overview maps;Lipid metabolism
XR_002400639.2	LOC110352150	ncbi_101799007	FADS1	0.958320555	9.24E-07	Global and overview maps;Lipid metabolism
XR_002400646.2	LOC110352151	ncbi_101799007	FADS1	0.973723287	9.44E-08	Global and overview maps;Lipid metabolism
XR_002400673.2	LOC106016078	ncbi_101799007	FADS1	0.958966473	8.55E-07	Global and overview maps;Lipid metabolism
XR_002400870.1	LOC110352222	ncbi_101799007	FADS1	0.970365657	1.71E-07	Global and overview maps;Lipid metabolism
XR_002400941.2	LOC106016276	ncbi_101799007	FADS1	0.977633002	4.25E-08	Global and overview maps;Lipid metabolism
XR_002401026.2	LOC110352298	ncbi_101799007	FADS1	0.980274829	2.28E-08	Global and overview maps;Lipid metabolism
XR_002401202.2	LOC110352351	ncbi_101799007	FADS1	0.980475214	2.16E-08	Global and overview maps;Lipid metabolism
XR_002401263.2	LOC106016476	ncbi_101799007	FADS1	0.956511876	1.14E-06	Global and overview maps;Lipid metabolism
XR_002401479.2	LOC106016692	ncbi_101799007	FADS1	0.981964417	1.46E-08	Global and overview maps;Lipid metabolism
XR_002401846.2	LOC106016921	ncbi_101799007	FADS1	0.961022392	6.64E-07	Global and overview maps;Lipid metabolism
XR_002401866.2	LOC106016894	ncbi_101799007	FADS1	0.982697799	1.19E-08	Global and overview maps;Lipid metabolism
XR_002401875.2	LOC110352627	ncbi_101799007	FADS1	0.981986111	1.45E-08	Global and overview maps;Lipid metabolism
XR_002402176.2	LOC110352712	ncbi_101799007	FADS1	0.977287057	4.58E-08	Global and overview maps;Lipid metabolism
XR_002402646.2	LOC101794014	ncbi_101799007	FADS1	0.982100864	1.40E-08	Global and overview maps;Lipid metabolism
XR_002402717.2	LOC106017475	ncbi_101799007	FADS1	0.955914669	1.22E-06	Global and overview maps;Lipid metabolism
XR_002402718.2	LOC106017475	ncbi_101799007	FADS1	0.98585773	4.35E-09	Global and overview maps;Lipid metabolism
XR_002402719.2	LOC106017476	ncbi_101799007	FADS1	0.960258857	7.30E-07	Global and overview maps;Lipid metabolism
XR_002403184.2	LOC106017746	ncbi_101799007	FADS1	0.984098555	7.80E-09	Global and overview maps;Lipid metabolism
XR_002404947.2	LOC110353846	ncbi_101799007	FADS1	0.987484762	2.37E-09	Global and overview maps;Lipid metabolism
XR_002405449.2	LOC110354050	ncbi_101799007	FADS1	0.96368023	4.68E-07	Global and overview maps;Lipid metabolism
XR_002405521.2	LOC106019445	ncbi_101799007	FADS1	0.977030371	4.85E-08	Global and overview maps;Lipid metabolism
XR_002405535.2	LOC110354079	ncbi_101799007	FADS1	0.983410521	9.62E-09	Global and overview maps;Lipid metabolism
XR_002405598.2	LOC106019518	ncbi_101799007	FADS1	0.951952765	1.86E-06	Global and overview maps;Lipid metabolism

XR_002405612.2	LOC106019471	ncbi_101799007	FADS1	0.963887969	4.55E-07	Global and overview maps;Lipid metabolism
XR_002405662.2	LOC106019549	ncbi_101799007	FADS1	0.952161249	1.82E-06	Global and overview maps;Lipid metabolism
XR_002405843.2	LOC106019674	ncbi_101799007	FADS1	0.983638849	8.98E-09	Global and overview maps;Lipid metabolism
XR_002405947.2	LOC106019753	ncbi_101799007	FADS1	0.984185199	7.59E-09	Global and overview maps;Lipid metabolism
XR_002406139.2	LOC110354333	ncbi_101799007	FADS1	0.983353527	9.79E-09	Global and overview maps;Lipid metabolism
XR_002406465.2	LOC106020114	ncbi_101799007	FADS1	0.983025448	1.08E-08	Global and overview maps;Lipid metabolism
XR_002406489.2	LOC106020129	ncbi_101799007	FADS1	0.965267868	3.75E-07	Global and overview maps;Lipid metabolism
XR_002406729.2	LOC106020349	ncbi_101799007	FADS1	0.993350065	1.01E-10	Global and overview maps;Lipid metabolism
XR_002406750.2	LOC106020393	ncbi_101799007	FADS1	0.987888872	2.01E-09	Global and overview maps;Lipid metabolism
XR_003492182.1	LOC113839646	ncbi_101799007	FADS1	0.959801275	7.73E-07	Global and overview maps;Lipid metabolism
XR_003492230.1	LOC113839672	ncbi_101799007	FADS1	0.980032196	2.42E-08	Global and overview maps;Lipid metabolism
XR_003492300.1	LOC110354130	ncbi_101799007	FADS1	0.958691549	8.84E-07	Global and overview maps;Lipid metabolism
XR_003492318.1	LOC113839721	ncbi_101799007	FADS1	0.960897434	6.74E-07	Global and overview maps;Lipid metabolism
XR_003492632.1	LOC106018295	ncbi_101799007	FADS1	0.953208632	1.63E-06	Global and overview maps;Lipid metabolism
XR_003492832.1	LOC113840106	ncbi_101799007	FADS1	0.971559864	1.40E-07	Global and overview maps;Lipid metabolism
XR_003492946.1	LOC113840172	ncbi_101799007	FADS1	0.95692996	1.09E-06	Global and overview maps;Lipid metabolism
XR_003493103.1	LOC113840309	ncbi_101799007	FADS1	0.981458545	1.67E-08	Global and overview maps;Lipid metabolism
XR_003493806.1	LOC113841174	ncbi_101799007	FADS1	0.985801068	4.44E-09	Global and overview maps;Lipid metabolism
XR_003493939.1	LOC113841382	ncbi_101799007	FADS1	0.952239538	1.81E-06	Global and overview maps;Lipid metabolism
XR_003494318.1	LOC113841955	ncbi_101799007	FADS1	0.958853038	8.67E-07	Global and overview maps;Lipid metabolism
XR_003494664.1	LOC113842319	ncbi_101799007	FADS1	0.977005865	4.87E-08	Global and overview maps;Lipid metabolism
XR_003494775.1	LOC110352361	ncbi_101799007	FADS1	0.98130559	1.74E-08	Global and overview maps;Lipid metabolism
XR_003494880.1	LOC106020483	ncbi_101799007	FADS1	0.955503228	1.27E-06	Global and overview maps;Lipid metabolism
XR_003494883.1	LOC106020483	ncbi_101799007	FADS1	0.976988019	4.89E-08	Global and overview maps;Lipid metabolism
XR_003494887.1	LOC113842395	ncbi_101799007	FADS1	0.980160477	2.34E-08	Global and overview maps;Lipid metabolism

XR_003494888.1	LOC113842396	ncbi_101799007	FADS1	0.98779356	2.09E-09	Global and overview maps;Lipid metabolism
XR_003494919.1	LOC113842414	ncbi_101799007	FADS1	-0.951411339	1.97E-06	Global and overview maps;Lipid metabolism
XR_003494957.1	LOC101799909	ncbi_101799007	FADS1	0.98803139	1.90E-09	Global and overview maps;Lipid metabolism
XR_003494960.1	LOC101799909	ncbi_101799007	FADS1	0.979339249	2.86E-08	Global and overview maps;Lipid metabolism
XR_003494961.1	LOC113842447	ncbi_101799007	FADS1	0.995726203	1.11E-11	Global and overview maps;Lipid metabolism
XR_003495202.1	LOC113842645	ncbi_101799007	FADS1	0.954364506	1.44E-06	Global and overview maps;Lipid metabolism
XR_003495207.1	LOC113842649	ncbi_101799007	FADS1	0.972833334	1.11E-07	Global and overview maps;Lipid metabolism
XR_003495345.1	LOC101793386	ncbi_101799007	FADS1	0.992746776	1.56E-10	Global and overview maps;Lipid metabolism
XR_003495483.1	LOC113842770	ncbi_101799007	FADS1	0.982730212	1.18E-08	Global and overview maps;Lipid metabolism
XR_003495635.1	LOC113842831	ncbi_101799007	FADS1	0.968003771	2.50E-07	Global and overview maps;Lipid metabolism
XR_003495767.1	LOC110352593	ncbi_101799007	FADS1	0.973720222	9.45E-08	Global and overview maps;Lipid metabolism
XR_003495918.1	LOC113843035	ncbi_101799007	FADS1	0.964572803	4.14E-07	Global and overview maps;Lipid metabolism
XR_003495930.1	LOC113839603	ncbi_101799007	FADS1	0.978617211	3.40E-08	Global and overview maps;Lipid metabolism
XR_003496139.1	LOC113843116	ncbi_101799007	FADS1	0.964770611	4.03E-07	Global and overview maps;Lipid metabolism
XR_003496222.1	LOC101792916	ncbi_101799007	FADS1	0.96827922	2.40E-07	Global and overview maps;Lipid metabolism
XR_003496334.1	LOC113843176	ncbi_101799007	FADS1	0.995406821	1.60E-11	Global and overview maps;Lipid metabolism
XR_003496440.1	LOC106015283	ncbi_101799007	FADS1	0.97747116	4.40E-08	Global and overview maps;Lipid metabolism
XR_003496447.1	LOC113843228	ncbi_101799007	FADS1	0.983091081	1.06E-08	Global and overview maps;Lipid metabolism
XR_003496598.1	LOC113843320	ncbi_101799007	FADS1	0.95880646	8.72E-07	Global and overview maps;Lipid metabolism
XR_003496885.1	LOC113843526	ncbi_101799007	FADS1	0.963900774	4.54E-07	Global and overview maps;Lipid metabolism
XR_003497001.1	LOC106017547	ncbi_101799007	FADS1	0.996780702	2.71E-12	Global and overview maps;Lipid metabolism
XR_003497002.1	LOC106017547	ncbi_101799007	FADS1	0.975334657	6.90E-08	Global and overview maps;Lipid metabolism
XR_003497032.1	LOC106020477	ncbi_101799007	FADS1	0.967750415	2.60E-07	Global and overview maps;Lipid metabolism
XR_003497296.1	LOC113843645	ncbi_101799007	FADS1	0.987649219	2.22E-09	Global and overview maps;Lipid metabolism

XR_003497330.1	LOC110352095	ncbi_101799007	FADS1	-0.965240283	3.77E-07	Global and overview maps;Lipid metabolism
XR_003497481.1	LOC110351804	ncbi_101799007	FADS1	0.977192467	4.68E-08	Global and overview maps;Lipid metabolism
XR_003497529.1	LOC113843776	ncbi_101799007	FADS1	0.98553042	4.88E-09	Global and overview maps;Lipid metabolism
XR_003497551.1	LOC110354397	ncbi_101799007	FADS1	0.971222047	1.48E-07	Global and overview maps;Lipid metabolism
XR_003497554.1	LOC110354397	ncbi_101799007	FADS1	0.951231372	2.00E-06	Global and overview maps;Lipid metabolism
XR_003497564.1	LOC101802970	ncbi_101799007	FADS1	0.970866461	1.57E-07	Global and overview maps;Lipid metabolism
XR_003497566.1	LOC101802970	ncbi_101799007	FADS1	0.996126749	6.82E-12	Global and overview maps;Lipid metabolism
XR_003497568.1	LOC101802970	ncbi_101799007	FADS1	0.961577564	6.18E-07	Global and overview maps;Lipid metabolism
XR_003497661.1	LOC101798800	ncbi_101799007	FADS1	0.985593858	4.77E-09	Global and overview maps;Lipid metabolism
XR_003497665.1	LOC110351913	ncbi_101799007	FADS1	0.976085102	5.92E-08	Global and overview maps;Lipid metabolism
XR_003497745.1	LOC110353307	ncbi_101799007	FADS1	0.971056869	1.52E-07	Global and overview maps;Lipid metabolism
XR_003497899.1	LOC110353379	ncbi_101799007	FADS1	0.972009855	1.29E-07	Global and overview maps;Lipid metabolism
XR_003497951.1	LOC113843960	ncbi_101799007	FADS1	0.98280709	1.15E-08	Global and overview maps;Lipid metabolism
XR_003498021.1	LOC101803684	ncbi_101799007	FADS1	0.968665015	2.26E-07	Global and overview maps;Lipid metabolism
XR_003498030.1	LOC101803684	ncbi_101799007	FADS1	0.960796694	6.83E-07	Global and overview maps;Lipid metabolism
XR_003498061.1	LOC106015393	ncbi_101799007	FADS1	0.961696614	6.09E-07	Global and overview maps;Lipid metabolism
XR_003498141.1	LOC113844039	ncbi_101799007	FADS1	0.966098814	3.33E-07	Global and overview maps;Lipid metabolism
XR_003498491.1	LOC113844169	ncbi_101799007	FADS1	0.989269814	1.10E-09	Global and overview maps;Lipid metabolism
XR_003498621.1	LOC113844221	ncbi_101799007	FADS1	0.963948674	4.51E-07	Global and overview maps;Lipid metabolism
XR_003498691.1	LOC106019447	ncbi_101799007	FADS1	0.966813641	3.00E-07	Global and overview maps;Lipid metabolism
XR_003498693.1	LOC110354267	ncbi_101799007	FADS1	0.978815278	3.24E-08	Global and overview maps;Lipid metabolism
XR_003498805.1	LOC106019442	ncbi_101799007	FADS1	0.978672672	3.35E-08	Global and overview maps;Lipid metabolism
XR_003498864.1	LOC113844340	ncbi_101799007	FADS1	0.987781222	2.10E-09	Global and overview maps;Lipid metabolism
XR_003498910.1	LOC106017955	ncbi_101799007	FADS1	0.975587009	6.56E-08	Global and overview maps;Lipid metabolism

XR_003499063.1	LOC110353608	ncbi_101799007	FADS1	0.991826002	2.83E-10	Global and overview maps;Lipid metabolism
XR_003499254.1	LOC106020392	ncbi_101799007	FADS1	0.954659995	1.40E-06	Global and overview maps;Lipid metabolism
XR_003499624.1	LOC113844766	ncbi_101799007	FADS1	0.951130472	2.02E-06	Global and overview maps;Lipid metabolism
XR_003499720.1	LOC113844791	ncbi_101799007	FADS1	0.984416112	7.05E-09	Global and overview maps;Lipid metabolism
XR_003499753.1	LOC113844812	ncbi_101799007	FADS1	0.976895853	4.99E-08	Global and overview maps;Lipid metabolism
XR_003499790.1	LOC101804048	ncbi_101799007	FADS1	0.974087138	8.81E-08	Global and overview maps;Lipid metabolism
XR_003499791.1	LOC113844835	ncbi_101799007	FADS1	0.954759729	1.38E-06	Global and overview maps;Lipid metabolism
XR_003499827.1	LOC106019660	ncbi_101799007	FADS1	0.959700111	7.82E-07	Global and overview maps;Lipid metabolism
XR_003499961.1	LOC110352806	ncbi_101799007	FADS1	0.96577821	3.49E-07	Global and overview maps;Lipid metabolism
XR_003499966.1	LOC110352806	ncbi_101799007	FADS1	0.971705675	1.36E-07	Global and overview maps;Lipid metabolism
XR_003500116.1	LOC113844988	ncbi_101799007	FADS1	0.956336512	1.16E-06	Global and overview maps;Lipid metabolism
XR_003500183.1	LOC106020052	ncbi_101799007	FADS1	0.969440204	1.99E-07	Global and overview maps;Lipid metabolism
XR_003500301.1	LOC113845068	ncbi_101799007	FADS1	0.982016067	1.44E-08	Global and overview maps;Lipid metabolism
XR_003500366.1	LOC110351294	ncbi_101799007	FADS1	0.97990539	2.49E-08	Global and overview maps;Lipid metabolism
XR_003500566.1	LOC113845180	ncbi_101799007	FADS1	0.963408738	4.86E-07	Global and overview maps;Lipid metabolism
XR_003500671.1	LOC106019837	ncbi_101799007	FADS1	0.974200791	8.62E-08	Global and overview maps;Lipid metabolism
XR_003500722.1	LOC113845266	ncbi_101799007	FADS1	0.981346886	1.72E-08	Global and overview maps;Lipid metabolism
XR_003500751.1	LOC113845280	ncbi_101799007	FADS1	0.97115775	1.50E-07	Global and overview maps;Lipid metabolism
XR_003501281.1	LOC106015373	ncbi_101799007	FADS1	0.958128848	9.45E-07	Global and overview maps;Lipid metabolism
XR_003501403.1	LOC113845679	ncbi_101799007	FADS1	0.957530824	1.01E-06	Global and overview maps;Lipid metabolism
XR_003501474.1	LOC106019132	ncbi_101799007	FADS1	0.978000251	3.91E-08	Global and overview maps;Lipid metabolism
XR_216744.4	LOC101791391	ncbi_101799007	FADS1	0.961670557	6.11E-07	Global and overview maps;Lipid metabolism
XR_217454.4	LOC101799933	ncbi_101799007	FADS1	0.952565209	1.75E-06	Global and overview maps;Lipid metabolism
MSTRG.11051.1	-	ncbi_101799557	PLPP1	0.995215128	1.96E-11	-
MSTRG.3256.4	-	ncbi_101799557	PLPP1	0.965854449	3.45E-07	-

MSTRG.4618.1	-	ncbi_101799557	PLPP1	0.955955234	1.21E-06	-
MSTRG.7978.1	-	ncbi_101799557	PLPP1	0.97786469	4.03E-08	-
MSTRG.8085.1	-	ncbi_101799557	PLPP1	0.968264444	2.40E-07	-
MSTRG.8536.1	-	ncbi_101799557	PLPP1	-0.968990495	2.14E-07	-
MSTRG.9342.1	-	ncbi_101799557	PLPP1	0.952816157	1.70E-06	-
XR_001187856.3	LOC106015665	ncbi_101799557	PLPP1	0.961481618	6.26E-07	-
XR_001188103.2	LOC106015815	ncbi_101799557	PLPP1	0.979256513	2.92E-08	-
XR_002398969.2	LOC106014835	ncbi_101799557	PLPP1	0.965535724	3.61E-07	-
XR_002399833.1	LOC106015481	ncbi_101799557	PLPP1	0.965168982	3.81E-07	-
XR_002400766.2	LOC110352189	ncbi_101799557	PLPP1	0.966820421	3.00E-07	-
XR_002401371.2	LOC110352432	ncbi_101799557	PLPP1	0.956120696	1.19E-06	-
XR_003492176.1	LOC113839643	ncbi_101799557	PLPP1	0.970669422	1.63E-07	-
XR_003492471.1	LOC113839840	ncbi_101799557	PLPP1	0.995993799	8.07E-12	-
XR_003492815.1	LOC113840095	ncbi_101799557	PLPP1	0.989700853	8.97E-10	-
XR_003492840.1	LOC113840110	ncbi_101799557	PLPP1	0.965331947	3.72E-07	-
XR_003492841.1	LOC113840110	ncbi_101799557	PLPP1	0.954860322	1.37E-06	-
XR_003492939.1	LOC101799584	ncbi_101799557	PLPP1	0.960278112	7.28E-07	-
XR_003492980.1	LOC113840212	ncbi_101799557	PLPP1	0.950343175	2.19E-06	-
XR_003493063.1	LOC113840289	ncbi_101799557	PLPP1	0.959743169	7.78E-07	-
XR_003493886.1	LOC113841287	ncbi_101799557	PLPP1	0.960617206	6.98E-07	-
XR_003494706.1	LOC113842335	ncbi_101799557	PLPP1	-0.970710128	1.62E-07	-
XR_003495828.1	LOC106016958	ncbi_101799557	PLPP1	0.966073644	3.34E-07	-
XR_003499181.1	LOC113844485	ncbi_101799557	PLPP1	0.959589688	7.93E-07	-

XR_003499673.1	LOC101796143	ncbi_101799557	PLPP1	0.978517261	3.48E-08	-
XR_003499823.1	LOC113844856	ncbi_101799557	PLPP1	0.967660198	2.64E-07	-
XR_003500120.1	LOC113844989	ncbi_101799557	PLPP1	0.953943661	1.51E-06	-
XR_003500629.1	LOC113845225	ncbi_101799557	PLPP1	0.963000132	5.13E-07	-
XR_003501390.1	LOC113845667	ncbi_101799557	PLPP1	0.9657809	3.49E-07	-
MSTRG.11051.1	-	ncbi_101800297	mboat2	-0.976340604	5.61E-08	Global and overview maps;Lipid metabolism;Lipid metabolism
MSTRG.12453.1	-	ncbi_101800297	mboat2	-0.966971563	2.93E-07	Global and overview maps;Lipid metabolism;Lipid metabolism
MSTRG.4618.1	-	ncbi_101800297	mboat2	-0.965492973	3.64E-07	Global and overview maps;Lipid metabolism;Lipid metabolism
MSTRG.5077.2	-	ncbi_101800297	mboat2	-0.963533148	4.78E-07	Global and overview maps;Lipid metabolism;Lipid metabolism
MSTRG.5077.3	-	ncbi_101800297	mboat2	-0.963533148	4.78E-07	Global and overview maps;Lipid metabolism;Lipid metabolism
MSTRG.965.12	-	ncbi_101800297	mboat2	0.951367237	1.97E-06	Global and overview maps;Lipid metabolism;Lipid metabolism
XR_002399833.1	LOC106015481	ncbi_101800297	mboat2	-0.967989137	2.51E-07	Global and overview maps;Lipid metabolism;Lipid metabolism
XR_003492176.1	LOC113839643	ncbi_101800297	mboat2	-0.963989808	4.49E-07	Global and overview maps;Lipid metabolism;Lipid metabolism
XR_003492815.1	LOC113840095	ncbi_101800297	mboat2	-0.964801699	4.01E-07	Global and overview maps;Lipid metabolism;Lipid metabolism
XR_003492837.1	LOC113840108	ncbi_101800297	mboat2	-0.950051635	2.25E-06	Global and overview maps;Lipid metabolism;Lipid metabolism
XR_003499673.1	LOC101796143	ncbi_101800297	mboat2	-	2.86E-07	Global and overview maps;Lipid metabolism;Lipid

				0.967130225		metabolism
XR_003499823.1	LOC113844856	ncbi_101800297	mboat2	-0.967146033	2.85E-07	Global and overview maps;Lipid metabolism;Lipid metabolism
XR_003500120.1	LOC113844989	ncbi_101800297	mboat2	-0.979306337	2.89E-08	Global and overview maps;Lipid metabolism;Lipid metabolism
MSTRG.10098.1	-	ncbi_101801145	FAT1	0.989932546	8.01E-10	-
MSTRG.10100.1	-	ncbi_101801145	FAT1	0.986021585	4.11E-09	-
MSTRG.10341.18	-	ncbi_101801145	FAT1	0.98968315	9.05E-10	-
MSTRG.10341.19	-	ncbi_101801145	FAT1	0.992926642	1.38E-10	-
MSTRG.10341.20	-	ncbi_101801145	FAT1	0.995744628	1.09E-11	-
MSTRG.10341.21	-	ncbi_101801145	FAT1	0.990919021	4.79E-10	-
MSTRG.10341.22	-	ncbi_101801145	FAT1	0.9946332	3.47E-11	-
MSTRG.10914.13	-	ncbi_101801145	FAT1	0.960036899	7.51E-07	-
MSTRG.12747.4	-	ncbi_101801145	FAT1	0.964030237	4.46E-07	-
MSTRG.12949.1	-	ncbi_101801145	FAT1	0.954478965	1.43E-06	-
MSTRG.13250.1	-	ncbi_101801145	FAT1	0.979244068	2.93E-08	-
MSTRG.13538.3	-	ncbi_101801145	FAT1	0.966800611	3.00E-07	-
MSTRG.13544.2	-	ncbi_101801145	FAT1	0.987652293	2.21E-09	-
MSTRG.13915.1	-	ncbi_101801145	FAT1	0.968096743	2.47E-07	-
MSTRG.14551.1	-	ncbi_101801145	FAT1	-0.982807918	1.15E-08	-
MSTRG.15965.1	-	ncbi_101801145	FAT1	0.970341004	1.72E-07	-
MSTRG.16028.1	-	ncbi_101801145	FAT1	0.975597885	6.54E-08	-
MSTRG.16827.2	-	ncbi_101801145	FAT1	0.961929811	5.91E-07	-
MSTRG.16889.1	-	ncbi_101801145	FAT1	0.970751973	1.60E-07	-

MSTRG.1816.1	-	ncbi_101801145	FAT1	0.955258195	1.31E-06	-
MSTRG.2348.4	-	ncbi_101801145	FAT1	0.974941184	7.46E-08	-
MSTRG.2606.1	-	ncbi_101801145	FAT1	0.987750566	2.13E-09	-
MSTRG.2608.1	-	ncbi_101801145	FAT1	0.982050908	1.42E-08	-
MSTRG.2763.2	-	ncbi_101801145	FAT1	0.95962916	7.89E-07	-
MSTRG.2904.1	-	ncbi_101801145	FAT1	0.981710125	1.56E-08	-
MSTRG.3080.1	-	ncbi_101801145	FAT1	0.980510421	2.14E-08	-
MSTRG.3622.7	-	ncbi_101801145	FAT1	0.980995122	1.89E-08	-
MSTRG.3639.1	-	ncbi_101801145	FAT1	0.968339399	2.38E-07	-
MSTRG.3832.2	-	ncbi_101801145	FAT1	0.970821767	1.59E-07	-
MSTRG.4704.1	-	ncbi_101801145	FAT1	0.95527682	1.31E-06	-
MSTRG.4893.1	-	ncbi_101801145	FAT1	0.971010851	1.54E-07	-
MSTRG.5011.1	-	ncbi_101801145	FAT1	0.972327301	1.22E-07	-
MSTRG.5066.1	-	ncbi_101801145	FAT1	-0.976790381	5.10E-08	-
MSTRG.5135.1	-	ncbi_101801145	FAT1	0.975226973	7.05E-08	-
MSTRG.5135.3	-	ncbi_101801145	FAT1	0.989241936	1.11E-09	-
MSTRG.5601.1	-	ncbi_101801145	FAT1	0.968405691	2.35E-07	-
MSTRG.582.1	-	ncbi_101801145	FAT1	0.96039562	7.18E-07	-
MSTRG.582.3	-	ncbi_101801145	FAT1	0.973875423	9.17E-08	-
MSTRG.582.4	-	ncbi_101801145	FAT1	0.956724373	1.11E-06	-
MSTRG.5846.1	-	ncbi_101801145	FAT1	0.974592715	7.99E-08	-
MSTRG.5881.2	-	ncbi_101801145	FAT1	0.982496157	1.26E-08	-
MSTRG.5997.1	-	ncbi_101801145	FAT1	0.965953388	3.40E-07	-
MSTRG.6017.1	-	ncbi_101801145	FAT1	0.950333725	2.19E-06	-

MSTRG.6127.1	-	ncbi_101801145	FAT1	0.974655476	7.89E-08	-
MSTRG.6316.1	-	ncbi_101801145	FAT1	-0.988705693	1.42E-09	-
MSTRG.6704.2	-	ncbi_101801145	FAT1	0.984007461	8.02E-09	-
MSTRG.6704.3	-	ncbi_101801145	FAT1	0.976708519	5.19E-08	-
MSTRG.6704.4	-	ncbi_101801145	FAT1	0.990430785	6.22E-10	-
MSTRG.715.1	-	ncbi_101801145	FAT1	0.974793754	7.68E-08	-
MSTRG.7239.1	-	ncbi_101801145	FAT1	0.973296098	1.02E-07	-
MSTRG.7248.1	-	ncbi_101801145	FAT1	0.986353094	3.64E-09	-
MSTRG.756.3	-	ncbi_101801145	FAT1	0.996843117	2.46E-12	-
MSTRG.7621.5	-	ncbi_101801145	FAT1	0.970777069	1.60E-07	-
MSTRG.7949.1	-	ncbi_101801145	FAT1	0.972622392	1.16E-07	-
MSTRG.7954.1	-	ncbi_101801145	FAT1	0.97455841	8.04E-08	-
MSTRG.8130.1	-	ncbi_101801145	FAT1	0.951370488	1.97E-06	-
MSTRG.852.1	-	ncbi_101801145	FAT1	0.987435057	2.42E-09	-
MSTRG.881.1	-	ncbi_101801145	FAT1	0.95192568	1.87E-06	-
MSTRG.885.1	-	ncbi_101801145	FAT1	0.979337232	2.87E-08	-
MSTRG.889.1	-	ncbi_101801145	FAT1	0.957078879	1.07E-06	-
MSTRG.9008.1	-	ncbi_101801145	FAT1	0.985207395	5.44E-09	-
MSTRG.9062.2	-	ncbi_101801145	FAT1	0.975443695	6.75E-08	-
XR_001185755.3	LOC106014431	ncbi_101801145	FAT1	0.965974994	3.39E-07	-
XR_001186606.3	LOC106014935	ncbi_101801145	FAT1	0.966377911	3.20E-07	-
XR_001186962.3	LOC106015138	ncbi_101801145	FAT1	0.973344671	1.01E-07	-
XR_001187494.3	LOC106015449	ncbi_101801145	FAT1	0.977887249	4.01E-08	-
XR_001187862.3	LOC106015672	ncbi_101801145	FAT1	0.96222018	5.69E-07	-

XR_001187865.3	LOC106015672	ncbi_101801145	FAT1	0.963565674	4.76E-07	-
XR_001187947.2	LOC106015729	ncbi_101801145	FAT1	0.968814803	2.20E-07	-
XR_001187991.3	LOC106015755	ncbi_101801145	FAT1	0.968988383	2.14E-07	-
XR_001188444.3	LOC106016033	ncbi_101801145	FAT1	0.972846158	1.11E-07	-
XR_001189244.3	LOC106016485	ncbi_101801145	FAT1	0.956013196	1.20E-06	-
XR_001189247.3	LOC106016487	ncbi_101801145	FAT1	0.960506366	7.08E-07	-
XR_001189916.3	LOC106016889	ncbi_101801145	FAT1	0.958778952	8.75E-07	-
XR_001189917.2	LOC106016889	ncbi_101801145	FAT1	0.977445102	4.43E-08	-
XR_001189941.3	LOC106016901	ncbi_101801145	FAT1	0.968799359	2.21E-07	-
XR_001190389.3	LOC106017139	ncbi_101801145	FAT1	0.953184942	1.64E-06	-
XR_001190631.3	LOC106017268	ncbi_101801145	FAT1	0.960379047	7.19E-07	-
XR_001190677.3	LOC106017297	ncbi_101801145	FAT1	0.968042681	2.49E-07	-
XR_001190951.3	LOC106017466	ncbi_101801145	FAT1	0.952207733	1.81E-06	-
XR_001190983.3	LOC106017488	ncbi_101801145	FAT1	0.968907516	2.17E-07	-
XR_001191855.3	LOC106018027	ncbi_101801145	FAT1	0.960104937	7.44E-07	-
XR_001192311.2	LOC106018298	ncbi_101801145	FAT1	0.962334439	5.60E-07	-
XR_001192492.3	LOC106018413	ncbi_101801145	FAT1	0.956555083	1.13E-06	-
XR_001193197.3	LOC106018814	ncbi_101801145	FAT1	0.965232057	3.77E-07	-
XR_001193335.3	LOC106018911	ncbi_101801145	FAT1	0.978847653	3.22E-08	-
XR_001193468.3	LOC106018998	ncbi_101801145	FAT1	0.960443554	7.14E-07	-
XR_001193619.3	LOC106019066	ncbi_101801145	FAT1	0.955414349	1.29E-06	-
XR_001194132.2	LOC106019368	ncbi_101801145	FAT1	0.968544673	2.30E-07	-
XR_001194441.2	LOC106019549	ncbi_101801145	FAT1	0.961912355	5.92E-07	-
XR_001195514.2	LOC106020129	ncbi_101801145	FAT1	0.96216878	5.73E-07	-
XR_002398893.2	LOC110351436	ncbi_101801145	FAT1	0.957545559	1.01E-06	-

XR_002398923.2	LOC101798301	ncbi_101801145	FAT1	0.967164615	2.84E-07	-
XR_002399173.2	LOC110351568	ncbi_101801145	FAT1	0.967892839	2.55E-07	-
XR_002399314.2	LOC106015131	ncbi_101801145	FAT1	0.952756586	1.71E-06	-
XR_002399732.1	LOC110351814	ncbi_101801145	FAT1	0.974589568	8.00E-08	-
XR_002399836.2	LOC106015519	ncbi_101801145	FAT1	0.97916679	2.98E-08	-
XR_002399884.2	LOC110351873	ncbi_101801145	FAT1	0.978979914	3.12E-08	-
XR_002400249.2	LOC110351990	ncbi_101801145	FAT1	0.973246415	1.03E-07	-
XR_002400594.2	LOC110352134	ncbi_101801145	FAT1	0.988217859	1.75E-09	-
XR_002400639.2	LOC110352150	ncbi_101801145	FAT1	0.962865628	5.22E-07	-
XR_002400646.2	LOC110352151	ncbi_101801145	FAT1	0.962497272	5.49E-07	-
XR_002400673.2	LOC106016078	ncbi_101801145	FAT1	0.954623515	1.40E-06	-
XR_002400870.1	LOC110352222	ncbi_101801145	FAT1	0.966596134	3.10E-07	-
XR_002400941.2	LOC106016276	ncbi_101801145	FAT1	0.971573481	1.39E-07	-
XR_002401026.2	LOC110352298	ncbi_101801145	FAT1	0.976590332	5.32E-08	-
XR_002401202.2	LOC110352351	ncbi_101801145	FAT1	0.956443898	1.15E-06	-
XR_002401479.2	LOC106016692	ncbi_101801145	FAT1	0.968635121	2.27E-07	-
XR_002401866.2	LOC106016894	ncbi_101801145	FAT1	0.973390029	1.00E-07	-
XR_002401875.2	LOC110352627	ncbi_101801145	FAT1	0.97262438	1.16E-07	-
XR_002402176.2	LOC110352712	ncbi_101801145	FAT1	0.955868016	1.22E-06	-
XR_002402646.2	LOC101794014	ncbi_101801145	FAT1	0.991180263	4.14E-10	-
XR_002402718.2	LOC106017475	ncbi_101801145	FAT1	0.980365336	2.22E-08	-
XR_002403184.2	LOC106017746	ncbi_101801145	FAT1	0.97462783	7.94E-08	-
XR_002404947.2	LOC110353846	ncbi_101801145	FAT1	0.982726999	1.18E-08	-
XR_002405065.2	LOC106019134	ncbi_101801145	FAT1	- 0.965596475	3.58E-07	-

XR_002405521.2	LOC106019445	ncbi_101801145	FAT1	0.950127117	2.23E-06	-
XR_002405535.2	LOC110354079	ncbi_101801145	FAT1	0.97099399	1.54E-07	-
XR_002405843.2	LOC106019674	ncbi_101801145	FAT1	0.976748619	5.15E-08	-
XR_002405947.2	LOC106019753	ncbi_101801145	FAT1	0.965764578	3.50E-07	-
XR_002406139.2	LOC110354333	ncbi_101801145	FAT1	0.98091987	1.93E-08	-
XR_002406465.2	LOC106020114	ncbi_101801145	FAT1	0.973123723	1.06E-07	-
XR_002406489.2	LOC106020129	ncbi_101801145	FAT1	0.955625725	1.26E-06	-
XR_002406729.2	LOC106020349	ncbi_101801145	FAT1	0.980147513	2.35E-08	-
XR_002406750.2	LOC106020393	ncbi_101801145	FAT1	0.978475104	3.51E-08	-
XR_003492182.1	LOC113839646	ncbi_101801145	FAT1	0.978660088	3.36E-08	-
XR_003492230.1	LOC113839672	ncbi_101801145	FAT1	0.959655243	7.87E-07	-
XR_003492632.1	LOC106018295	ncbi_101801145	FAT1	0.956319983	1.16E-06	-
XR_003492829.1	LOC113840104	ncbi_101801145	FAT1	0.954621132	1.40E-06	-
XR_003492832.1	LOC113840106	ncbi_101801145	FAT1	0.9610722	6.59E-07	-
XR_003493103.1	LOC113840309	ncbi_101801145	FAT1	0.965309422	3.73E-07	-
XR_003493806.1	LOC113841174	ncbi_101801145	FAT1	0.975597409	6.54E-08	-
XR_003494318.1	LOC113841955	ncbi_101801145	FAT1	0.957724606	9.90E-07	-
XR_003494664.1	LOC113842319	ncbi_101801145	FAT1	0.983263292	1.01E-08	-
XR_003494775.1	LOC110352361	ncbi_101801145	FAT1	0.965974994	3.39E-07	-
XR_003494880.1	LOC106020483	ncbi_101801145	FAT1	0.963003616	5.13E-07	-
XR_003494883.1	LOC106020483	ncbi_101801145	FAT1	0.958320796	9.24E-07	-
XR_003494884.1	LOC106020483	ncbi_101801145	FAT1	0.950687	2.11E-06	-
XR_003494887.1	LOC113842395	ncbi_101801145	FAT1	0.974210578	8.60E-08	-
XR_003494888.1	LOC113842396	ncbi_101801145	FAT1	0.971063459	1.52E-07	-
XR_003494957.1	LOC101799909	ncbi_101801145	FAT1	0.98248442	1.26E-08	-

XR_003494960.1	LOC101799909	ncbi_101801145	FAT1	0.963538056	4.77E-07	-
XR_003494961.1	LOC113842447	ncbi_101801145	FAT1	0.98792338	1.98E-09	-
XR_003495202.1	LOC113842645	ncbi_101801145	FAT1	0.965854021	3.45E-07	-
XR_003495207.1	LOC113842649	ncbi_101801145	FAT1	0.973505636	9.83E-08	-
XR_003495345.1	LOC101793386	ncbi_101801145	FAT1	0.985490888	4.94E-09	-
XR_003495483.1	LOC113842770	ncbi_101801145	FAT1	0.977612671	4.27E-08	-
XR_003495635.1	LOC113842831	ncbi_101801145	FAT1	0.955162235	1.32E-06	-
XR_003495767.1	LOC110352593	ncbi_101801145	FAT1	0.955989635	1.21E-06	-
XR_003495918.1	LOC113843035	ncbi_101801145	FAT1	0.962223362	5.69E-07	-
XR_003495930.1	LOC113839603	ncbi_101801145	FAT1	0.976446281	5.49E-08	-
XR_003496139.1	LOC113843116	ncbi_101801145	FAT1	0.954317037	1.45E-06	-
XR_003496222.1	LOC101792916	ncbi_101801145	FAT1	0.956082218	1.20E-06	-
XR_003496334.1	LOC113843176	ncbi_101801145	FAT1	0.990864897	4.93E-10	-
XR_003496440.1	LOC106015283	ncbi_101801145	FAT1	0.970068371	1.80E-07	-
XR_003496447.1	LOC113843228	ncbi_101801145	FAT1	0.964404462	4.24E-07	-
XR_003496598.1	LOC113843320	ncbi_101801145	FAT1	0.954252163	1.46E-06	-
XR_003496624.1	LOC113843334	ncbi_101801145	FAT1	0.970382326	1.71E-07	-
XR_003496885.1	LOC113843526	ncbi_101801145	FAT1	0.951880222	1.87E-06	-
XR_003496907.1	LOC113843533	ncbi_101801145	FAT1	0.973380565	1.01E-07	-
XR_003497001.1	LOC106017547	ncbi_101801145	FAT1	0.99321068	1.12E-10	-
XR_003497002.1	LOC106017547	ncbi_101801145	FAT1	0.985515975	4.90E-09	-
XR_003497023.1	LOC113843564	ncbi_101801145	FAT1	0.952424974	1.77E-06	-
XR_003497032.1	LOC106020477	ncbi_101801145	FAT1	0.959912004	7.62E-07	-
XR_003497296.1	LOC113843645	ncbi_101801145	FAT1	0.972539634	1.17E-07	-
XR_003497481.1	LOC110351804	ncbi_101801145	FAT1	0.984028839	7.97E-09	-

XR_003497511.1	LOC110353153	ncbi_101801145	FAT1	0.95974729	7.78E-07	-
XR_003497529.1	LOC113843776	ncbi_101801145	FAT1	0.983595779	9.10E-09	-
XR_003497551.1	LOC110354397	ncbi_101801145	FAT1	0.977278259	4.59E-08	-
XR_003497554.1	LOC110354397	ncbi_101801145	FAT1	0.960112606	7.44E-07	-
XR_003497564.1	LOC101802970	ncbi_101801145	FAT1	0.975477802	6.70E-08	-
XR_003497566.1	LOC101802970	ncbi_101801145	FAT1	0.993167889	1.16E-10	-
XR_003497661.1	LOC101798800	ncbi_101801145	FAT1	0.967906179	2.54E-07	-
XR_003497665.1	LOC110351913	ncbi_101801145	FAT1	0.964331061	4.28E-07	-
XR_003497745.1	LOC110353307	ncbi_101801145	FAT1	0.98236272	1.30E-08	-
XR_003497816.1	LOC113843897	ncbi_101801145	FAT1	0.957968513	9.63E-07	-
XR_003497899.1	LOC110353379	ncbi_101801145	FAT1	0.957507423	1.02E-06	-
XR_003497951.1	LOC113843960	ncbi_101801145	FAT1	0.965377699	3.70E-07	-
XR_003498021.1	LOC101803684	ncbi_101801145	FAT1	0.97859093	3.42E-08	-
XR_003498030.1	LOC101803684	ncbi_101801145	FAT1	0.955185633	1.32E-06	-
XR_003498141.1	LOC113844039	ncbi_101801145	FAT1	0.971739451	1.35E-07	-
XR_003498491.1	LOC113844169	ncbi_101801145	FAT1	0.986076319	4.03E-09	-
XR_003498621.1	LOC113844221	ncbi_101801145	FAT1	0.951993347	1.85E-06	-
XR_003498691.1	LOC106019447	ncbi_101801145	FAT1	0.957919674	9.68E-07	-
XR_003498693.1	LOC110354267	ncbi_101801145	FAT1	0.975996011	6.03E-08	-
XR_003498805.1	LOC106019442	ncbi_101801145	FAT1	0.963707658	4.67E-07	-
XR_003498857.1	LOC106018813	ncbi_101801145	FAT1	-0.958821051	8.70E-07	-
XR_003498864.1	LOC113844340	ncbi_101801145	FAT1	0.971772741	1.35E-07	-
XR_003498910.1	LOC106017955	ncbi_101801145	FAT1	0.964065117	4.44E-07	-
XR_003499063.1	LOC110353608	ncbi_101801145	FAT1	0.985979483	4.17E-09	-

XR_003499624.1	LOC113844766	ncbi_101801145	FAT1	0.951074663	2.03E-06	-
XR_003499720.1	LOC113844791	ncbi_101801145	FAT1	0.979025579	3.09E-08	-
XR_003499753.1	LOC113844812	ncbi_101801145	FAT1	0.973867125	9.19E-08	-
XR_003499790.1	LOC101804048	ncbi_101801145	FAT1	0.981454337	1.67E-08	-
XR_003499791.1	LOC113844835	ncbi_101801145	FAT1	0.954971326	1.35E-06	-
XR_003499827.1	LOC106019660	ncbi_101801145	FAT1	0.962511714	5.48E-07	-
XR_003499961.1	LOC110352806	ncbi_101801145	FAT1	0.97489949	7.52E-08	-
XR_003499966.1	LOC110352806	ncbi_101801145	FAT1	0.967207423	2.83E-07	-
XR_003500183.1	LOC106020052	ncbi_101801145	FAT1	0.958251049	9.31E-07	-
XR_003500301.1	LOC113845068	ncbi_101801145	FAT1	0.972383506	1.21E-07	-
XR_003500366.1	LOC110351294	ncbi_101801145	FAT1	0.963916072	4.53E-07	-
XR_003500566.1	LOC113845180	ncbi_101801145	FAT1	0.960132955	7.42E-07	-
XR_003500671.1	LOC106019837	ncbi_101801145	FAT1	0.985240906	5.38E-09	-
XR_003500722.1	LOC113845266	ncbi_101801145	FAT1	0.964136146	4.40E-07	-
XR_003500751.1	LOC113845280	ncbi_101801145	FAT1	0.958805817	8.72E-07	-
XR_003500777.1	LOC113845300	ncbi_101801145	FAT1	-0.961801571	6.01E-07	-
XR_003501281.1	LOC106015373	ncbi_101801145	FAT1	0.958234389	9.33E-07	-
XR_003501403.1	LOC113845679	ncbi_101801145	FAT1	0.953749277	1.54E-06	-
XR_003501474.1	LOC106019132	ncbi_101801145	FAT1	0.956876315	1.09E-06	-
MSTRG.10900.2	-	ncbi_101802708	PNPLA2	-0.956796266	1.10E-06	-
MSTRG.10098.1	-	ncbi_101802983	MGLL	0.990118056	7.30E-10	Global and overview maps;Lipid metabolism
MSTRG.10100.1	-	ncbi_101802983	MGLL	0.991240423	4.00E-10	Global and overview maps;Lipid metabolism
MSTRG.10341.18	-	ncbi_101802983	MGLL	0.989275266	1.10E-09	Global and overview maps;Lipid metabolism

MSTRG.10341.19	-	ncbi_101802983	MGLL	0.996514467	4.03E-12	Global and overview maps;Lipid metabolism
MSTRG.10341.20	-	ncbi_101802983	MGLL	0.997942636	2.89E-13	Global and overview maps;Lipid metabolism
MSTRG.10341.21	-	ncbi_101802983	MGLL	0.997864883	3.48E-13	Global and overview maps;Lipid metabolism
MSTRG.10341.22	-	ncbi_101802983	MGLL	0.995686463	1.17E-11	Global and overview maps;Lipid metabolism
MSTRG.10914.13	-	ncbi_101802983	MGLL	0.953285382	1.62E-06	Global and overview maps;Lipid metabolism
MSTRG.12747.4	-	ncbi_101802983	MGLL	0.971707223	1.36E-07	Global and overview maps;Lipid metabolism
MSTRG.12949.1	-	ncbi_101802983	MGLL	0.951733632	1.90E-06	Global and overview maps;Lipid metabolism
MSTRG.13250.1	-	ncbi_101802983	MGLL	0.968004006	2.50E-07	Global and overview maps;Lipid metabolism
MSTRG.13538.3	-	ncbi_101802983	MGLL	0.97304358	1.07E-07	Global and overview maps;Lipid metabolism
MSTRG.13544.2	-	ncbi_101802983	MGLL	0.994122757	5.47E-11	Global and overview maps;Lipid metabolism
MSTRG.13915.1	-	ncbi_101802983	MGLL	0.979335991	2.87E-08	Global and overview maps;Lipid metabolism
MSTRG.14551.1	-	ncbi_101802983	MGLL	-0.970780496	1.60E-07	Global and overview maps;Lipid metabolism
MSTRG.15964.1	-	ncbi_101802983	MGLL	0.975652943	6.47E-08	Global and overview maps;Lipid metabolism
MSTRG.15965.1	-	ncbi_101802983	MGLL	0.980021468	2.42E-08	Global and overview maps;Lipid metabolism
MSTRG.16028.1	-	ncbi_101802983	MGLL	0.973568955	9.72E-08	Global and overview maps;Lipid metabolism
MSTRG.16118.5	-	ncbi_101802983	MGLL	0.950546953	2.14E-06	Global and overview maps;Lipid metabolism
MSTRG.16827.2	-	ncbi_101802983	MGLL	0.95033508	2.19E-06	Global and overview maps;Lipid metabolism
MSTRG.16889.1	-	ncbi_101802983	MGLL	0.984595104	6.66E-09	Global and overview maps;Lipid metabolism
MSTRG.1816.1	-	ncbi_101802983	MGLL	0.964098949	4.42E-07	Global and overview maps;Lipid metabolism
MSTRG.2348.4	-	ncbi_101802983	MGLL	0.975534285	6.63E-08	Global and overview maps;Lipid metabolism
MSTRG.2606.1	-	ncbi_101802983	MGLL	0.984711287	6.41E-09	Global and overview maps;Lipid metabolism
MSTRG.2608.1	-	ncbi_101802983	MGLL	0.981396977	1.70E-08	Global and overview maps;Lipid metabolism
MSTRG.2763.2	-	ncbi_101802983	MGLL	0.953745739	1.54E-06	Global and overview maps;Lipid metabolism
MSTRG.2904.1	-	ncbi_101802983	MGLL	0.99000654	7.72E-10	Global and overview maps;Lipid metabolism

MSTRG.3080.1	-	ncbi_101802983	MGLL	0.986620596	3.30E-09	Global and overview maps;Lipid metabolism
MSTRG.3622.7	-	ncbi_101802983	MGLL	0.984192231	7.57E-09	Global and overview maps;Lipid metabolism
MSTRG.3639.1	-	ncbi_101802983	MGLL	0.986138623	3.94E-09	Global and overview maps;Lipid metabolism
MSTRG.3832.2	-	ncbi_101802983	MGLL	0.979363602	2.85E-08	Global and overview maps;Lipid metabolism
MSTRG.4704.1	-	ncbi_101802983	MGLL	0.956687784	1.12E-06	Global and overview maps;Lipid metabolism
MSTRG.4893.1	-	ncbi_101802983	MGLL	0.982225009	1.36E-08	Global and overview maps;Lipid metabolism
MSTRG.5011.1	-	ncbi_101802983	MGLL	0.983328029	9.86E-09	Global and overview maps;Lipid metabolism
MSTRG.5066.1	-	ncbi_101802983	MGLL	-0.97152903	1.40E-07	Global and overview maps;Lipid metabolism
MSTRG.5135.1	-	ncbi_101802983	MGLL	0.973827857	9.26E-08	Global and overview maps;Lipid metabolism
MSTRG.5135.3	-	ncbi_101802983	MGLL	0.990515301	5.95E-10	Global and overview maps;Lipid metabolism
MSTRG.5601.1	-	ncbi_101802983	MGLL	0.956939529	1.08E-06	Global and overview maps;Lipid metabolism
MSTRG.582.1	-	ncbi_101802983	MGLL	0.971061685	1.52E-07	Global and overview maps;Lipid metabolism
MSTRG.582.3	-	ncbi_101802983	MGLL	0.98175305	1.55E-08	Global and overview maps;Lipid metabolism
MSTRG.582.4	-	ncbi_101802983	MGLL	0.965504027	3.63E-07	Global and overview maps;Lipid metabolism
MSTRG.5846.1	-	ncbi_101802983	MGLL	0.973307075	1.02E-07	Global and overview maps;Lipid metabolism
MSTRG.5881.2	-	ncbi_101802983	MGLL	0.985951447	4.21E-09	Global and overview maps;Lipid metabolism
MSTRG.5997.1	-	ncbi_101802983	MGLL	0.970394243	1.70E-07	Global and overview maps;Lipid metabolism
MSTRG.6127.1	-	ncbi_101802983	MGLL	0.9835816	9.14E-09	Global and overview maps;Lipid metabolism
MSTRG.6316.1	-	ncbi_101802983	MGLL	-0.987225234	2.62E-09	Global and overview maps;Lipid metabolism
MSTRG.6704.2	-	ncbi_101802983	MGLL	0.989579559	9.51E-10	Global and overview maps;Lipid metabolism
MSTRG.6704.3	-	ncbi_101802983	MGLL	0.979963277	2.46E-08	Global and overview maps;Lipid metabolism
MSTRG.6704.4	-	ncbi_101802983	MGLL	0.996246956	5.83E-12	Global and overview maps;Lipid metabolism
MSTRG.715.1	-	ncbi_101802983	MGLL	0.984776867	6.28E-09	Global and overview maps;Lipid metabolism
MSTRG.7239.1	-	ncbi_101802983	MGLL	0.981871235	1.50E-08	Global and overview maps;Lipid metabolism

MSTRG.7248.1	-	ncbi_101802983	MGLL	0.988113594	1.83E-09	Global and overview maps;Lipid metabolism
MSTRG.756.3	-	ncbi_101802983	MGLL	0.996732382	2.92E-12	Global and overview maps;Lipid metabolism
MSTRG.7621.5	-	ncbi_101802983	MGLL	0.980069709	2.40E-08	Global and overview maps;Lipid metabolism
MSTRG.7949.1	-	ncbi_101802983	MGLL	0.983615093	9.05E-09	Global and overview maps;Lipid metabolism
MSTRG.7954.1	-	ncbi_101802983	MGLL	0.982655682	1.20E-08	Global and overview maps;Lipid metabolism
MSTRG.8113.1	-	ncbi_101802983	MGLL	0.956971114	1.08E-06	Global and overview maps;Lipid metabolism
MSTRG.852.1	-	ncbi_101802983	MGLL	0.985861214	4.35E-09	Global and overview maps;Lipid metabolism
MSTRG.881.1	-	ncbi_101802983	MGLL	0.957522045	1.01E-06	Global and overview maps;Lipid metabolism
MSTRG.885.1	-	ncbi_101802983	MGLL	0.987521554	2.33E-09	Global and overview maps;Lipid metabolism
MSTRG.889.1	-	ncbi_101802983	MGLL	0.972549177	1.17E-07	Global and overview maps;Lipid metabolism
MSTRG.9008.1	-	ncbi_101802983	MGLL	0.988550763	1.52E-09	Global and overview maps;Lipid metabolism
MSTRG.9025.1	-	ncbi_101802983	MGLL	0.958870491	8.65E-07	Global and overview maps;Lipid metabolism
MSTRG.9062.2	-	ncbi_101802983	MGLL	0.983318946	9.89E-09	Global and overview maps;Lipid metabolism
MSTRG.9637.1	-	ncbi_101802983	MGLL	-0.950331937	2.19E-06	Global and overview maps;Lipid metabolism
XR_001185755.3	LOC106014431	ncbi_101802983	MGLL	0.977416229	4.45E-08	Global and overview maps;Lipid metabolism
XR_001186359.3	LOC101798301	ncbi_101802983	MGLL	0.9583461	9.21E-07	Global and overview maps;Lipid metabolism
XR_001186606.3	LOC106014935	ncbi_101802983	MGLL	0.97356013	9.73E-08	Global and overview maps;Lipid metabolism
XR_001186962.3	LOC106015138	ncbi_101802983	MGLL	0.984700021	6.44E-09	Global and overview maps;Lipid metabolism
XR_001187167.3	LOC106015263	ncbi_101802983	MGLL	0.959931957	7.60E-07	Global and overview maps;Lipid metabolism
XR_001187494.3	LOC106015449	ncbi_101802983	MGLL	0.967729279	2.61E-07	Global and overview maps;Lipid metabolism
XR_001187862.3	LOC106015672	ncbi_101802983	MGLL	0.967698443	2.62E-07	Global and overview maps;Lipid metabolism
XR_001187865.3	LOC106015672	ncbi_101802983	MGLL	0.969346787	2.02E-07	Global and overview maps;Lipid metabolism
XR_001187947.2	LOC106015729	ncbi_101802983	MGLL	0.977150263	4.72E-08	Global and overview maps;Lipid metabolism
XR_001187991.3	LOC106015755	ncbi_101802983	MGLL	0.966773691	3.02E-07	Global and overview maps;Lipid metabolism

XR_001188444.3	LOC106016033	ncbi_101802983	MGLL	0.97896641	3.13E-08	Global and overview maps;Lipid metabolism
XR_001189244.3	LOC106016485	ncbi_101802983	MGLL	0.970330364	1.72E-07	Global and overview maps;Lipid metabolism
XR_001189247.3	LOC106016487	ncbi_101802983	MGLL	0.97116761	1.50E-07	Global and overview maps;Lipid metabolism
XR_001189916.3	LOC106016889	ncbi_101802983	MGLL	0.968230806	2.42E-07	Global and overview maps;Lipid metabolism
XR_001189917.2	LOC106016889	ncbi_101802983	MGLL	0.985131125	5.58E-09	Global and overview maps;Lipid metabolism
XR_001189941.3	LOC106016901	ncbi_101802983	MGLL	0.979769969	2.58E-08	Global and overview maps;Lipid metabolism
XR_001190389.3	LOC106017139	ncbi_101802983	MGLL	0.961633473	6.14E-07	Global and overview maps;Lipid metabolism
XR_001190631.3	LOC106017268	ncbi_101802983	MGLL	0.977002431	4.87E-08	Global and overview maps;Lipid metabolism
XR_001190677.3	LOC106017297	ncbi_101802983	MGLL	0.972339725	1.22E-07	Global and overview maps;Lipid metabolism
XR_001190983.3	LOC106017488	ncbi_101802983	MGLL	0.973970566	9.01E-08	Global and overview maps;Lipid metabolism
XR_001191855.3	LOC106018027	ncbi_101802983	MGLL	0.96443391	4.22E-07	Global and overview maps;Lipid metabolism
XR_001192311.2	LOC106018298	ncbi_101802983	MGLL	0.954385276	1.44E-06	Global and overview maps;Lipid metabolism
XR_001193197.3	LOC106018814	ncbi_101802983	MGLL	0.975058268	7.29E-08	Global and overview maps;Lipid metabolism
XR_001193335.3	LOC106018911	ncbi_101802983	MGLL	0.980153096	2.35E-08	Global and overview maps;Lipid metabolism
XR_001193468.3	LOC106018998	ncbi_101802983	MGLL	0.968426508	2.34E-07	Global and overview maps;Lipid metabolism
XR_001193619.3	LOC106019066	ncbi_101802983	MGLL	0.963536402	4.77E-07	Global and overview maps;Lipid metabolism
XR_001194132.2	LOC106019368	ncbi_101802983	MGLL	0.973345473	1.01E-07	Global and overview maps;Lipid metabolism
XR_001194441.2	LOC106019549	ncbi_101802983	MGLL	0.961007143	6.65E-07	Global and overview maps;Lipid metabolism
XR_001194622.3	LOC106019644	ncbi_101802983	MGLL	0.964321764	4.29E-07	Global and overview maps;Lipid metabolism
XR_001195514.2	LOC106020129	ncbi_101802983	MGLL	0.958507226	9.03E-07	Global and overview maps;Lipid metabolism
XR_002398893.2	LOC110351436	ncbi_101802983	MGLL	0.973080171	1.06E-07	Global and overview maps;Lipid metabolism
XR_002398923.2	LOC101798301	ncbi_101802983	MGLL	0.958742793	8.78E-07	Global and overview maps;Lipid metabolism
XR_002399173.2	LOC110351568	ncbi_101802983	MGLL	0.974359573	8.36E-08	Global and overview maps;Lipid metabolism
XR_002399314.2	LOC106015131	ncbi_101802983	MGLL	0.961907971	5.92E-07	Global and overview maps;Lipid metabolism
XR_002399432.2	LOC110351688	ncbi_101802983	MGLL	0.952024328	1.85E-06	Global and overview maps;Lipid metabolism

XR_002399732.1	LOC110351814	ncbi_101802983	MGLL	0.9812261	1.78E-08	Global and overview maps;Lipid metabolism
XR_002399836.2	LOC106015519	ncbi_101802983	MGLL	0.982969328	1.10E-08	Global and overview maps;Lipid metabolism
XR_002399884.2	LOC110351873	ncbi_101802983	MGLL	0.968081383	2.47E-07	Global and overview maps;Lipid metabolism
XR_002400249.2	LOC110351990	ncbi_101802983	MGLL	0.968039685	2.49E-07	Global and overview maps;Lipid metabolism
XR_002400594.2	LOC110352134	ncbi_101802983	MGLL	0.979512154	2.75E-08	Global and overview maps;Lipid metabolism
XR_002400639.2	LOC110352150	ncbi_101802983	MGLL	0.964457618	4.21E-07	Global and overview maps;Lipid metabolism
XR_002400646.2	LOC110352151	ncbi_101802983	MGLL	0.972767618	1.13E-07	Global and overview maps;Lipid metabolism
XR_002400673.2	LOC106016078	ncbi_101802983	MGLL	0.964421625	4.23E-07	Global and overview maps;Lipid metabolism
XR_002400870.1	LOC110352222	ncbi_101802983	MGLL	0.979490166	2.76E-08	Global and overview maps;Lipid metabolism
XR_002400941.2	LOC106016276	ncbi_101802983	MGLL	0.978660499	3.36E-08	Global and overview maps;Lipid metabolism
XR_002401026.2	LOC110352298	ncbi_101802983	MGLL	0.987878787	2.02E-09	Global and overview maps;Lipid metabolism
XR_002401202.2	LOC110352351	ncbi_101802983	MGLL	0.965229866	3.78E-07	Global and overview maps;Lipid metabolism
XR_002401479.2	LOC106016692	ncbi_101802983	MGLL	0.979782363	2.57E-08	Global and overview maps;Lipid metabolism
XR_002401846.2	LOC106016921	ncbi_101802983	MGLL	0.953658446	1.56E-06	Global and overview maps;Lipid metabolism
XR_002401866.2	LOC106016894	ncbi_101802983	MGLL	0.980118828	2.37E-08	Global and overview maps;Lipid metabolism
XR_002401875.2	LOC110352627	ncbi_101802983	MGLL	0.973311777	1.02E-07	Global and overview maps;Lipid metabolism
XR_002402176.2	LOC110352712	ncbi_101802983	MGLL	0.96990952	1.85E-07	Global and overview maps;Lipid metabolism
XR_002402359.2	LOC106017212	ncbi_101802983	MGLL	0.95025845	2.21E-06	Global and overview maps;Lipid metabolism
XR_002402646.2	LOC101794014	ncbi_101802983	MGLL	0.98730309	2.54E-09	Global and overview maps;Lipid metabolism
XR_002402717.2	LOC106017475	ncbi_101802983	MGLL	0.960020793	7.52E-07	Global and overview maps;Lipid metabolism
XR_002402718.2	LOC106017475	ncbi_101802983	MGLL	0.988463793	1.58E-09	Global and overview maps;Lipid metabolism
XR_002402719.2	LOC106017476	ncbi_101802983	MGLL	0.96528603	3.75E-07	Global and overview maps;Lipid metabolism
XR_002403184.2	LOC106017746	ncbi_101802983	MGLL	0.980639153	2.07E-08	Global and overview maps;Lipid metabolism
XR_002404947.2	LOC110353846	ncbi_101802983	MGLL	0.978642238	3.38E-08	Global and overview maps;Lipid metabolism
XR_002405065.2	LOC106019134	ncbi_101802983	MGLL	-	8.14E-07	Global and overview maps;Lipid metabolism

				0.959377762		
XR_002405521.2	LOC106019445	ncbi_101802983	MGLL	0.964715088	4.06E-07	Global and overview maps;Lipid metabolism
XR_002405535.2	LOC110354079	ncbi_101802983	MGLL	0.983197346	1.03E-08	Global and overview maps;Lipid metabolism
XR_002405612.2	LOC106019471	ncbi_101802983	MGLL	0.9620447	5.82E-07	Global and overview maps;Lipid metabolism
XR_002405662.2	LOC106019549	ncbi_101802983	MGLL	0.952171412	1.82E-06	Global and overview maps;Lipid metabolism
XR_002405843.2	LOC106019674	ncbi_101802983	MGLL	0.985470901	4.98E-09	Global and overview maps;Lipid metabolism
XR_002405947.2	LOC106019753	ncbi_101802983	MGLL	0.97422007	8.59E-08	Global and overview maps;Lipid metabolism
XR_002406139.2	LOC110354333	ncbi_101802983	MGLL	0.978485702	3.50E-08	Global and overview maps;Lipid metabolism
XR_002406465.2	LOC106020114	ncbi_101802983	MGLL	0.974381695	8.32E-08	Global and overview maps;Lipid metabolism
XR_002406489.2	LOC106020129	ncbi_101802983	MGLL	0.960890105	6.75E-07	Global and overview maps;Lipid metabolism
XR_002406644.2	LOC110354539	ncbi_101802983	MGLL	0.952073704	1.84E-06	Global and overview maps;Lipid metabolism
XR_002406729.2	LOC106020349	ncbi_101802983	MGLL	0.986156795	3.91E-09	Global and overview maps;Lipid metabolism
XR_002406750.2	LOC106020393	ncbi_101802983	MGLL	0.986058266	4.05E-09	Global and overview maps;Lipid metabolism
XR_003492182.1	LOC113839646	ncbi_101802983	MGLL	0.971144367	1.50E-07	Global and overview maps;Lipid metabolism
XR_003492230.1	LOC113839672	ncbi_101802983	MGLL	0.974564195	8.03E-08	Global and overview maps;Lipid metabolism
XR_003492236.1	LOC113839675	ncbi_101802983	MGLL	0.953863761	1.52E-06	Global and overview maps;Lipid metabolism
XR_003492318.1	LOC113839721	ncbi_101802983	MGLL	0.963197024	5.00E-07	Global and overview maps;Lipid metabolism
XR_003492632.1	LOC106018295	ncbi_101802983	MGLL	0.961558022	6.20E-07	Global and overview maps;Lipid metabolism
XR_003492832.1	LOC113840106	ncbi_101802983	MGLL	0.976435769	5.50E-08	Global and overview maps;Lipid metabolism
XR_003493103.1	LOC113840309	ncbi_101802983	MGLL	0.97442746	8.25E-08	Global and overview maps;Lipid metabolism
XR_003493806.1	LOC113841174	ncbi_101802983	MGLL	0.975353321	6.87E-08	Global and overview maps;Lipid metabolism
XR_003494318.1	LOC113841955	ncbi_101802983	MGLL	0.970891873	1.57E-07	Global and overview maps;Lipid metabolism
XR_003494664.1	LOC113842319	ncbi_101802983	MGLL	0.992618685	1.70E-10	Global and overview maps;Lipid metabolism
XR_003494714.1	LOC113842344	ncbi_101802983	MGLL	0.955005875	1.35E-06	Global and overview maps;Lipid metabolism
XR_003494775.1	LOC110352361	ncbi_101802983	MGLL	0.977416229	4.45E-08	Global and overview maps;Lipid metabolism

XR_003494880.1	LOC106020483	ncbi_101802983	MGLL	0.96541445	3.68E-07	Global and overview maps;Lipid metabolism
XR_003494883.1	LOC106020483	ncbi_101802983	MGLL	0.971573265	1.39E-07	Global and overview maps;Lipid metabolism
XR_003494887.1	LOC113842395	ncbi_101802983	MGLL	0.981792001	1.53E-08	Global and overview maps;Lipid metabolism
XR_003494888.1	LOC113842396	ncbi_101802983	MGLL	0.974214676	8.60E-08	Global and overview maps;Lipid metabolism
XR_003494919.1	LOC113842414	ncbi_101802983	MGLL	-0.950633746	2.12E-06	Global and overview maps;Lipid metabolism
XR_003494957.1	LOC101799909	ncbi_101802983	MGLL	0.982102355	1.40E-08	Global and overview maps;Lipid metabolism
XR_003494960.1	LOC101799909	ncbi_101802983	MGLL	0.975252166	7.01E-08	Global and overview maps;Lipid metabolism
XR_003494961.1	LOC113842447	ncbi_101802983	MGLL	0.9929375	1.37E-10	Global and overview maps;Lipid metabolism
XR_003495202.1	LOC113842645	ncbi_101802983	MGLL	0.951005015	2.05E-06	Global and overview maps;Lipid metabolism
XR_003495207.1	LOC113842649	ncbi_101802983	MGLL	0.968158594	2.44E-07	Global and overview maps;Lipid metabolism
XR_003495345.1	LOC101793386	ncbi_101802983	MGLL	0.99160033	3.25E-10	Global and overview maps;Lipid metabolism
XR_003495483.1	LOC113842770	ncbi_101802983	MGLL	0.979089188	3.04E-08	Global and overview maps;Lipid metabolism
XR_003495635.1	LOC113842831	ncbi_101802983	MGLL	0.969521794	1.97E-07	Global and overview maps;Lipid metabolism
XR_003495767.1	LOC110352593	ncbi_101802983	MGLL	0.961251715	6.45E-07	Global and overview maps;Lipid metabolism
XR_003495918.1	LOC113843035	ncbi_101802983	MGLL	0.960194637	7.36E-07	Global and overview maps;Lipid metabolism
XR_003495930.1	LOC113839603	ncbi_101802983	MGLL	0.983076866	1.06E-08	Global and overview maps;Lipid metabolism
XR_003496222.1	LOC101792916	ncbi_101802983	MGLL	0.964801239	4.01E-07	Global and overview maps;Lipid metabolism
XR_003496334.1	LOC113843176	ncbi_101802983	MGLL	0.989923592	8.04E-10	Global and overview maps;Lipid metabolism
XR_003496440.1	LOC106015283	ncbi_101802983	MGLL	0.973022617	1.08E-07	Global and overview maps;Lipid metabolism
XR_003496447.1	LOC113843228	ncbi_101802983	MGLL	0.974163003	8.68E-08	Global and overview maps;Lipid metabolism
XR_003496598.1	LOC113843320	ncbi_101802983	MGLL	0.955762859	1.24E-06	Global and overview maps;Lipid metabolism
XR_003496624.1	LOC113843334	ncbi_101802983	MGLL	0.950054483	2.25E-06	Global and overview maps;Lipid metabolism
XR_003496885.1	LOC113843526	ncbi_101802983	MGLL	0.956820713	1.10E-06	Global and overview maps;Lipid metabolism
XR_003496907.1	LOC113843533	ncbi_101802983	MGLL	0.961705398	6.08E-07	Global and overview maps;Lipid metabolism

XR_003497001.1	LOC106017547	ncbi_101802983	MGLL	0.993989239	6.12E-11	Global and overview maps;Lipid metabolism
XR_003497002.1	LOC106017547	ncbi_101802983	MGLL	0.973412595	1.00E-07	Global and overview maps;Lipid metabolism
XR_003497023.1	LOC113843564	ncbi_101802983	MGLL	0.967150439	2.85E-07	Global and overview maps;Lipid metabolism
XR_003497032.1	LOC106020477	ncbi_101802983	MGLL	0.970509774	1.67E-07	Global and overview maps;Lipid metabolism
XR_003497296.1	LOC113843645	ncbi_101802983	MGLL	0.980983542	1.90E-08	Global and overview maps;Lipid metabolism
XR_003497481.1	LOC110351804	ncbi_101802983	MGLL	0.983681376	8.87E-09	Global and overview maps;Lipid metabolism
XR_003497511.1	LOC110353153	ncbi_101802983	MGLL	0.950735743	2.10E-06	Global and overview maps;Lipid metabolism
XR_003497529.1	LOC113843776	ncbi_101802983	MGLL	0.990922712	4.78E-10	Global and overview maps;Lipid metabolism
XR_003497551.1	LOC110354397	ncbi_101802983	MGLL	0.977841481	4.05E-08	Global and overview maps;Lipid metabolism
XR_003497564.1	LOC101802970	ncbi_101802983	MGLL	0.974229862	8.57E-08	Global and overview maps;Lipid metabolism
XR_003497566.1	LOC101802970	ncbi_101802983	MGLL	0.995825115	9.92E-12	Global and overview maps;Lipid metabolism
XR_003497661.1	LOC101798800	ncbi_101802983	MGLL	0.965261426	3.76E-07	Global and overview maps;Lipid metabolism
XR_003497665.1	LOC110351913	ncbi_101802983	MGLL	0.972434332	1.20E-07	Global and overview maps;Lipid metabolism
XR_003497745.1	LOC110353307	ncbi_101802983	MGLL	0.982333965	1.32E-08	Global and overview maps;Lipid metabolism
XR_003497899.1	LOC110353379	ncbi_101802983	MGLL	0.969807849	1.88E-07	Global and overview maps;Lipid metabolism
XR_003497951.1	LOC113843960	ncbi_101802983	MGLL	0.975509779	6.66E-08	Global and overview maps;Lipid metabolism
XR_003498021.1	LOC101803684	ncbi_101802983	MGLL	0.972853656	1.11E-07	Global and overview maps;Lipid metabolism
XR_003498030.1	LOC101803684	ncbi_101802983	MGLL	0.974647238	7.91E-08	Global and overview maps;Lipid metabolism
XR_003498141.1	LOC113844039	ncbi_101802983	MGLL	0.97789719	4.00E-08	Global and overview maps;Lipid metabolism
XR_003498397.1	LOC101797653	ncbi_101802983	MGLL	0.955586206	1.26E-06	Global and overview maps;Lipid metabolism
XR_003498491.1	LOC113844169	ncbi_101802983	MGLL	0.994950199	2.56E-11	Global and overview maps;Lipid metabolism
XR_003498621.1	LOC113844221	ncbi_101802983	MGLL	0.955165384	1.32E-06	Global and overview maps;Lipid metabolism
XR_003498691.1	LOC106019447	ncbi_101802983	MGLL	0.969992414	1.82E-07	Global and overview maps;Lipid metabolism
XR_003498693.1	LOC110354267	ncbi_101802983	MGLL	0.98088134	1.95E-08	Global and overview maps;Lipid metabolism
XR_003498805.1	LOC106019442	ncbi_101802983	MGLL	0.975772479	6.31E-08	Global and overview maps;Lipid metabolism

XR_003498857.1	LOC106018813	ncbi_101802983	MGLL	-0.953880041	1.52E-06	Global and overview maps;Lipid metabolism
XR_003498864.1	LOC113844340	ncbi_101802983	MGLL	0.987545893	2.31E-09	Global and overview maps;Lipid metabolism
XR_003498910.1	LOC106017955	ncbi_101802983	MGLL	0.965933945	3.41E-07	Global and overview maps;Lipid metabolism
XR_003499063.1	LOC110353608	ncbi_101802983	MGLL	0.994226287	5.00E-11	Global and overview maps;Lipid metabolism
XR_003499254.1	LOC106020392	ncbi_101802983	MGLL	0.9544554	1.43E-06	Global and overview maps;Lipid metabolism
XR_003499624.1	LOC113844766	ncbi_101802983	MGLL	0.966278134	3.25E-07	Global and overview maps;Lipid metabolism
XR_003499720.1	LOC113844791	ncbi_101802983	MGLL	0.989162804	1.16E-09	Global and overview maps;Lipid metabolism
XR_003499753.1	LOC113844812	ncbi_101802983	MGLL	0.984678002	6.48E-09	Global and overview maps;Lipid metabolism
XR_003499790.1	LOC101804048	ncbi_101802983	MGLL	0.974850058	7.60E-08	Global and overview maps;Lipid metabolism
XR_003499791.1	LOC113844835	ncbi_101802983	MGLL	0.955163644	1.32E-06	Global and overview maps;Lipid metabolism
XR_003499827.1	LOC106019660	ncbi_101802983	MGLL	0.970675427	1.63E-07	Global and overview maps;Lipid metabolism
XR_003499961.1	LOC110352806	ncbi_101802983	MGLL	0.975442487	6.75E-08	Global and overview maps;Lipid metabolism
XR_003499966.1	LOC110352806	ncbi_101802983	MGLL	0.970059988	1.80E-07	Global and overview maps;Lipid metabolism
XR_003500116.1	LOC113844988	ncbi_101802983	MGLL	0.958178629	9.39E-07	Global and overview maps;Lipid metabolism
XR_003500183.1	LOC106020052	ncbi_101802983	MGLL	0.968063117	2.48E-07	Global and overview maps;Lipid metabolism
XR_003500301.1	LOC113845068	ncbi_101802983	MGLL	0.974084814	8.81E-08	Global and overview maps;Lipid metabolism
XR_003500366.1	LOC110351294	ncbi_101802983	MGLL	0.970153803	1.77E-07	Global and overview maps;Lipid metabolism
XR_003500375.1	LOC106018420	ncbi_101802983	MGLL	0.951420761	1.96E-06	Global and overview maps;Lipid metabolism
XR_003500566.1	LOC113845180	ncbi_101802983	MGLL	0.966886013	2.97E-07	Global and overview maps;Lipid metabolism
XR_003500671.1	LOC106019837	ncbi_101802983	MGLL	0.989274443	1.10E-09	Global and overview maps;Lipid metabolism
XR_003500722.1	LOC113845266	ncbi_101802983	MGLL	0.971406475	1.43E-07	Global and overview maps;Lipid metabolism
XR_003500751.1	LOC113845280	ncbi_101802983	MGLL	0.97363616	9.60E-08	Global and overview maps;Lipid metabolism
XR_003500777.1	LOC113845300	ncbi_101802983	MGLL	-0.960929892	6.71E-07	Global and overview maps;Lipid metabolism

XR_003501281.1	LOC106015373	ncbi_101802983	MGLL	0.973188115	1.04E-07	Global and overview maps;Lipid metabolism
XR_003501403.1	LOC113845679	ncbi_101802983	MGLL	0.962354809	5.59E-07	Global and overview maps;Lipid metabolism
XR_003501474.1	LOC106019132	ncbi_101802983	MGLL	0.963375981	4.88E-07	Global and overview maps;Lipid metabolism
XR_216744.4	LOC101791391	ncbi_101802983	MGLL	0.959195336	8.32E-07	Global and overview maps;Lipid metabolism
XR_217454.4	LOC101799933	ncbi_101802983	MGLL	0.965441535	3.66E-07	Global and overview maps;Lipid metabolism

Description: lncRNA_id: lncRNA id number; lncRNA_symbol: lncRNA symbol number; gene_id: mRNA id number; gene_symbol: mRNA symbol number; cor: mRNA-lncRNA correlation; p-value: significance of mRNA-lncRNA correlation relationship; pathway: name of the pathway in which the mRNA is located; K_ID: number of the pathway where the mRNA is located.
